# Supplementary material for: Revealing the chirality origin and homochirality crystallization of Ag14 nanocluster at the molecular level
Source: Nat Commun. 2021 Aug 17;12:4966. doi: 10.1038/s41467-021-25275-2 (PMC8371133; doi:10.1038/s41467-021-25275-2)
Supplement: Supplementary file 1 — Supplementary Information [file 41467_2021_25275_MOESM1_ESM.pdf]

Supplementary Information

Revealing the Chirality Origin and Homochirality Crystallization of Ag_{14} Nanocluster at the Molecular Level

Xiao-Qian Liang,[1] Ying-Zhou Li,[2] Zhi Wang,[1] Shan-Shan Zhang,[1] Yi-Cheng Liu,[1] Zhao-Zhen Cao,[1] Lei Feng,[1] Zhi-Yong Gao,[3] Qing-Wang Xue,[4] Chen-Ho Tung,[1] and Di Sun[1*]

[1]School of Chemistry and Chemical Engineering, State Key Laboratory of Crystal Materials, Shandong University, Ji'nan, 250100, People's Republic of China.

[2]Shandong Provincial Key Laboratory of Molecular Engineering, Qilu University of Technology (Shandong Academy of Science), Ji'nan, 250353, People's Republic of China.

[3]School of Chemistry and Chemical Engineering, Collaborative Innovation Center of Henan Province for Green Manufacturing of Fine Chemicals, Key Laboratory of Green Chemical Media and Reactions, Ministry of Education, Henan Normal University, Henan Xinxiang 453007, People's Republic of China.

[4]Shandong Provincial Key Laboratory of Chemical Energy Storage and Novel Cell Technology, and School of Chemistry and Chemical Engineering, Liaocheng University, Liaocheng 252000, China.

[*]To whom correspondence should be addressed. E-mail: dsun@sdu.edu.cn.

Supplementary Methods.

Materials and reagents

Solvents and reagents (Adamas-Beta®) were purchased from Shanghai Titan Scientific Co., Ltd. In particular, *p*-nitrothiophenol and 1,6-bis(diphenylphosphino)hexane were obtained from a commercial source. Unless otherwise noted, all of the chemicals were reagent grade and used without any further purification.

Elemental mapping images

Morphology of the samples and elemental composition analyses were measured using a Gemini300 field emission scanning electron microscope (FESEM; Zeiss, Germany) equipped with a Bruker Quantax 200 XFlash 6|100 energy dispersive X-ray spectroscopy (EDS) attachment.

Nuclear magnetic resonance (NMR)

^{1}H NMR and ^{31}P NMR spectra were recorded at room temperature on a Bruker AM-400 spectrometer (400 MHz ^{1}H NMR and ^{31}P NMR, 298 K, δ/ppm), the chemical shifts are reported in ppm.

Mass Spectrometry

Mass spectrum (MS) was recorded on a Bruker impact II high definition mass spectrometer, quadrupole and time-of-flight (Q/TOF) modules in the positive ion mode. Typical measurement conditions are as follows: end plate offset = -400 V; dry gas = 3 L min^{-1}, nebulizer = 0.3 bar, capillary voltage = 3500 V, sample flow rate = 4 L min^{-1}. The data analysis of mass spectrum was performed based on the isotope distribution patterns using Compass Data Analysis software (Version 4.4).

Circular Dichroism Spectrometry

Dispersing one crystal into 100 mg KBr by grinding could effectively reduce the macroscopic anisotropic arrangement, and the mixtures were tableted for testing. CD and CPL were collected with an Applied Photophysics ChirascanV100 model.

Photoluminescence spectroscopy

Temperature-dependent photoluminescence measurements were carried out in an

Edinburgh spectrofluorimeter (F920S) coupled with an Optistat DN cryostat (Oxford Instruments), and the ITC temperature controller and a pressure gauge were used to realize the variable-temperature measurement in the range of 83-293 K. Spectra were collected at different temperatures after a 2 min homoiothermy.

Photoluminescence lifetime

Time-resolved photoluminescence lifetime measurements were measured on Edinburgh spectrofluorimeter (F920S) using a time-correlated single-photon counting technique.

UV-Vis spectroscopy

The diffuse-reflectance spectra were recorded on a UV-Vis spectrophotometer (Evolution 220, ISA-220 accessory, Thermo Scientific).

Powder X-ray diffraction (PXRD)

Powder X-ray diffraction (PXRD) analyses were carried out on a microcrystalline powder using a Rigaku Oxford Diffraction XtaLAB Synergy diffractometer using Cu radiation (λ = 1.54184 Å). The PXRD patterns were processed with the *CrysAlis^{Pro}* software suite[1] using the Powder function.

Fourier transform infrared spectroscopy (FTIR)

IR spectra were recorded on a Bruker Tensor II spectrophotometer (Bruker Optics GmbH, Ettlingen, Germany) utilizing a single attenuated total reflectance (ATR) accessory covering a wavenumber range from 400 to 4000 cm^{-1}. The final spectrum was the average of 32 scans accumulated using Bruker's Opus software 8.1, taken at 4 cm^{-1} resolution. The samples were measured under the same mechanical force pushing the samples in contact with the diamond window.

Supplementary Note 1.

Single crystals of **SD/*rac*-Ag14a**, **SD/*L*-Ag14, SD/*R*-Ag14** and **SD/*rac*-Ag14b** with appropriate dimensions were chosen under an optical microscope and quickly coated with high vacuum grease (Dow Corning Corporation) to prevent decomposition. Crystals were mounted on CryoLoop™ loop and the cell parameters and intensity data were recorded on a Rigaku Oxford Diffraction XtaLAB Synergy diffractometer equipped with a HyPix-6000HE Hybrid Photon Counting (HPC) detector operating in shutterless mode and an Oxford Cryosystems Cryostream 800 Plus using Cu $K\alpha$ (λ = 1.54184 Å) for **SD/*rac*-Ag14a**, **SD/*rac*-Ag14b**, **SD/*L*-Ag14** and **SD/*R*-Ag14** from PhotonJet micro-focus X-ray Source at 100 K and 173 K, respectively. Data were processed using the *CrystAlis*^{Pro} software suite.[1] These structures were solved using the charge-flipping algorithm, as implemented in the program *SUPERFLIP*[2] and refined by full-matrix least-squares techniques against F_o^2 using the SHELXL program[3] through the OLEX2 interface.[4] Hydrogen atoms at carbon were placed in calculated positions and refined isotropically by using a riding model. Appropriate restraints or constraints were applied to the geometry and the atomic displacement parameters of the atoms in the cluster. The structures were examined using the Addsym subroutine of PLATON[5] to ensure that no additional symmetry could be applied to the models. Pertinent crystallographic data collection and refinement parameters are collated in Supplementary Table 1. Selected bond lengths and angles are collated in Supplementary Table 2.

Supplementary Note 2.

DFT calculations were performed with the Gaussian 09W suite of programs.[6] For the optimizations of the cluster **SD/*L*-Ag14**, the gradient-corrected MPW1PW91 exchange correlation functional, based on the generalized gradient approximation (GGA) was utilized.[7] For structural optimization and TD-DFT calculations, LanL2DZ (Los Alamos effective core potential double-ζ) basis set was employed for Ag atoms, augmented with d- or f-type polarization functions and 6-31G(d) basis set was used for other atoms. The MPW1PW91 functional is expected to be more suitable for the second and third row transition metal systems.[8] Spin-restricted calculations were used for geometry optimization. The TD-DFT calculations were performed to get the most probable transitions and the orbitals corresponding to the main peaks in the calculated electronic spectrum. A total of 900 singlet states were chosen in the calculations. The root is set as 1 in the TD-DFT calculations. Data for orbital composition analysis with Mulliken partition are from Gaussian 09 calculations and processed with Multiwfn software.[9] The most probable transitions were determined based on the oscillator strength values and weights. The electronic plot of the intensity *vs*. energy (nm) spectrum was fitted with Gaussian with a half-width at half-height of 0.25 eV. The CD spectra were convoluted with a Gaussian line shape with a half-width at half-height of 0.25 eV.

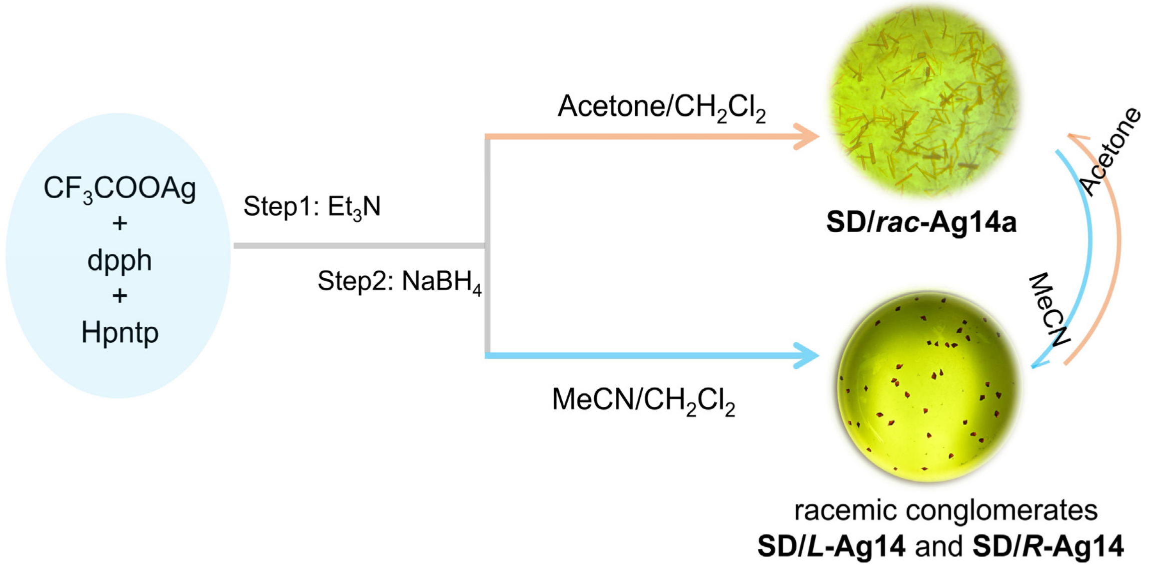

Supplementary Figure 1. Synthetic route for SD/Ag14. The detailed synthetic route to **SD/*rac*-Ag14a**, **SD/*L*-Ag14** and **SD/*R*-Ag14** (Hpntp = *p*-nitrothiophenol and dpph = 1,6-bis(diphenylphosphino)hexane).

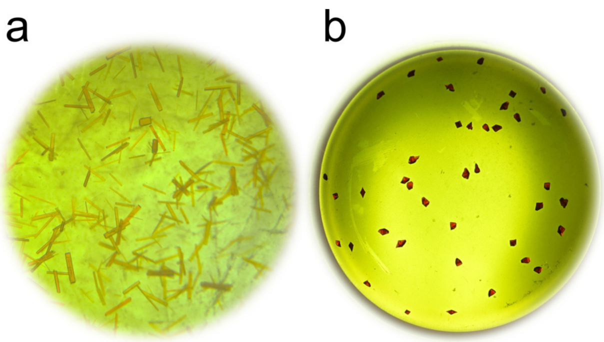

Supplementary Figure 2: Photographic images of SD/Ag14. a The photo of the crystals of **SD/*rac*-Ag14a**. **b** The photo of the crystals of the racemic conglomerates **SD/*L*-Ag14** and **SD/*R*-Ag14**.

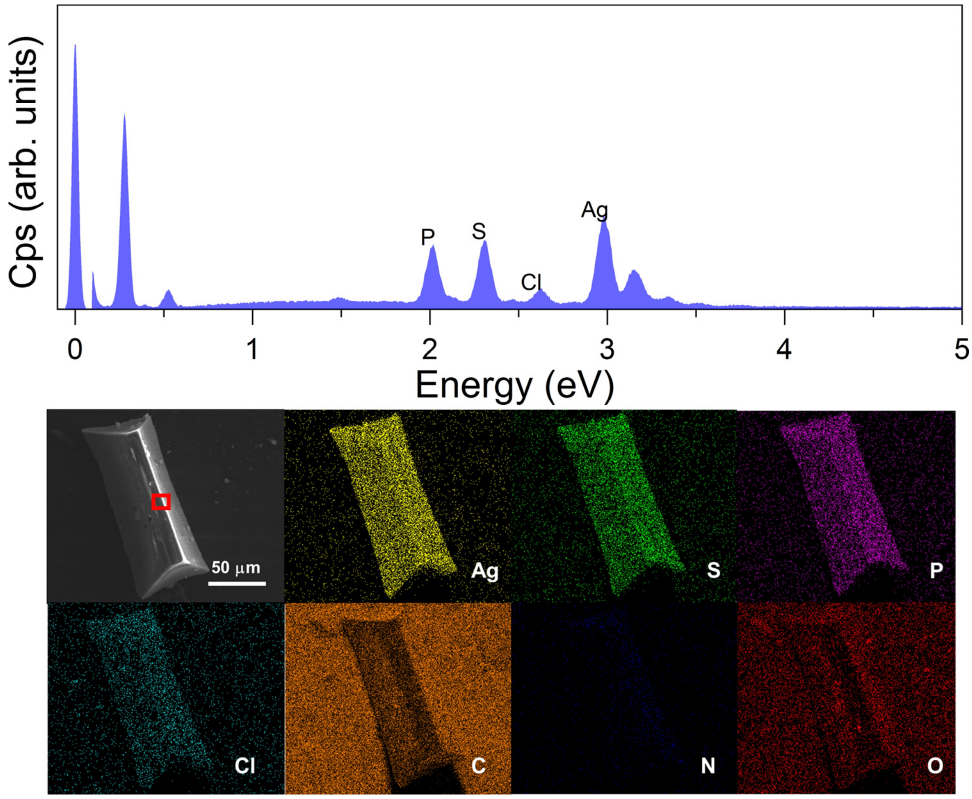

Supplementary Figure 3: Energy dispersive spectroscopic analysis of SD/*L*-Ag14 and SD/*R*-Ag14. Energy dispersive spectroscopy (EDS) spectrum and mapping figures of racemic conglomerates **SD/*L*-Ag14** and **SD/*R*-Ag14**.

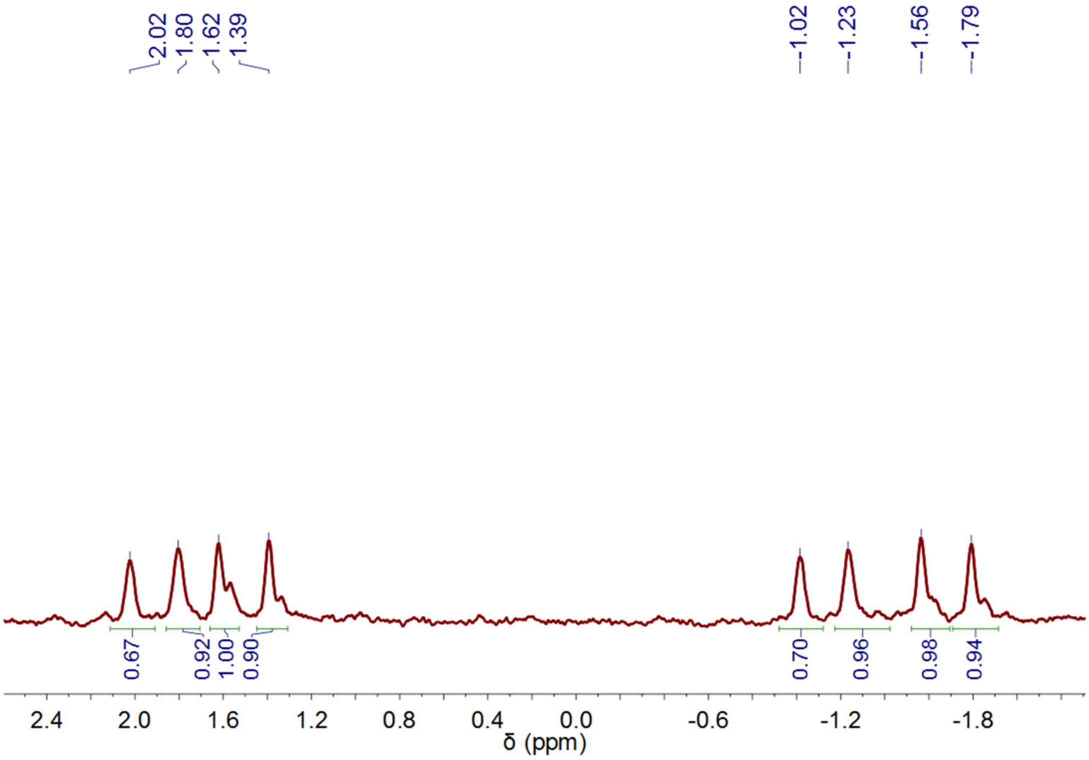

Supplementary Figure 4: The ^{31}P nuclear magnetic resonance spectrometry study of SD/*L*-Ag14 and SD/*R*-Ag14. The ^{31}P nuclear magnetic resonance (NMR) spectrum of racemic conglomerates **SD/*L*-Ag14** and **SD/*R*-Ag14** collected in one batch dissolved in CD_2Cl_2.

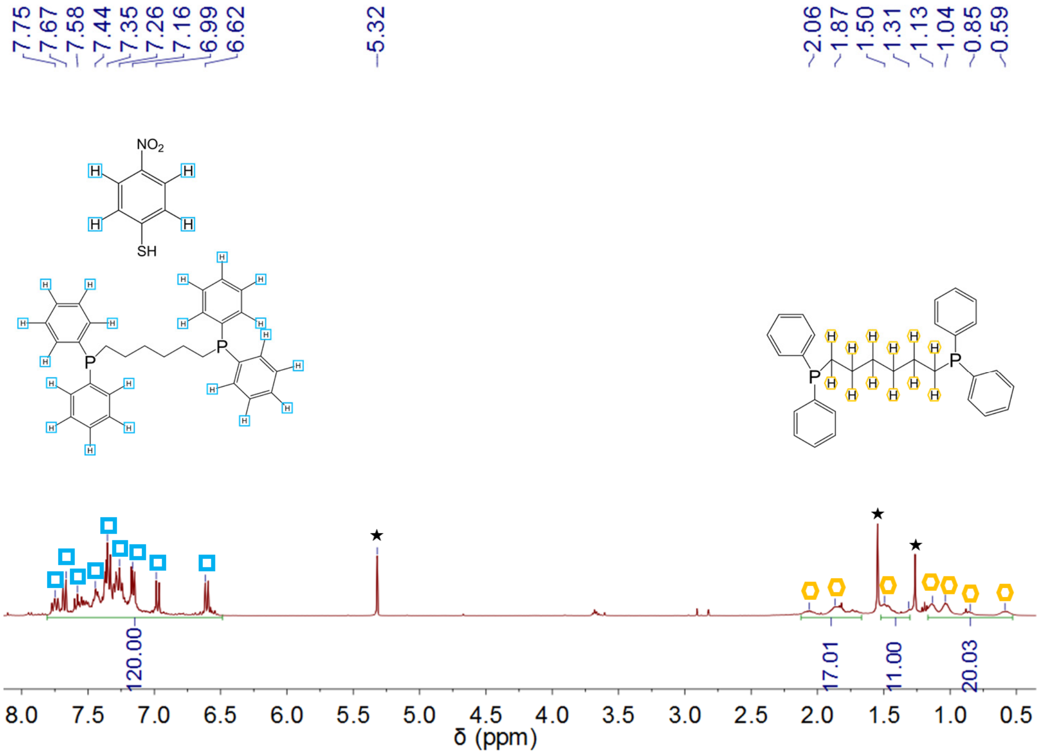

Supplementary Figure 5: The ^{1}H nuclear magnetic resonance spectrometry study of SD/*L*-Ag14 and SD/*R*-Ag14. The ^{1}H nuclear magnetic resonance (NMR) spectrum of racemic conglomerates **SD/*L*-Ag14** and **SD/*R*-Ag14** collected in one batch dissolved in CD_2Cl_2.

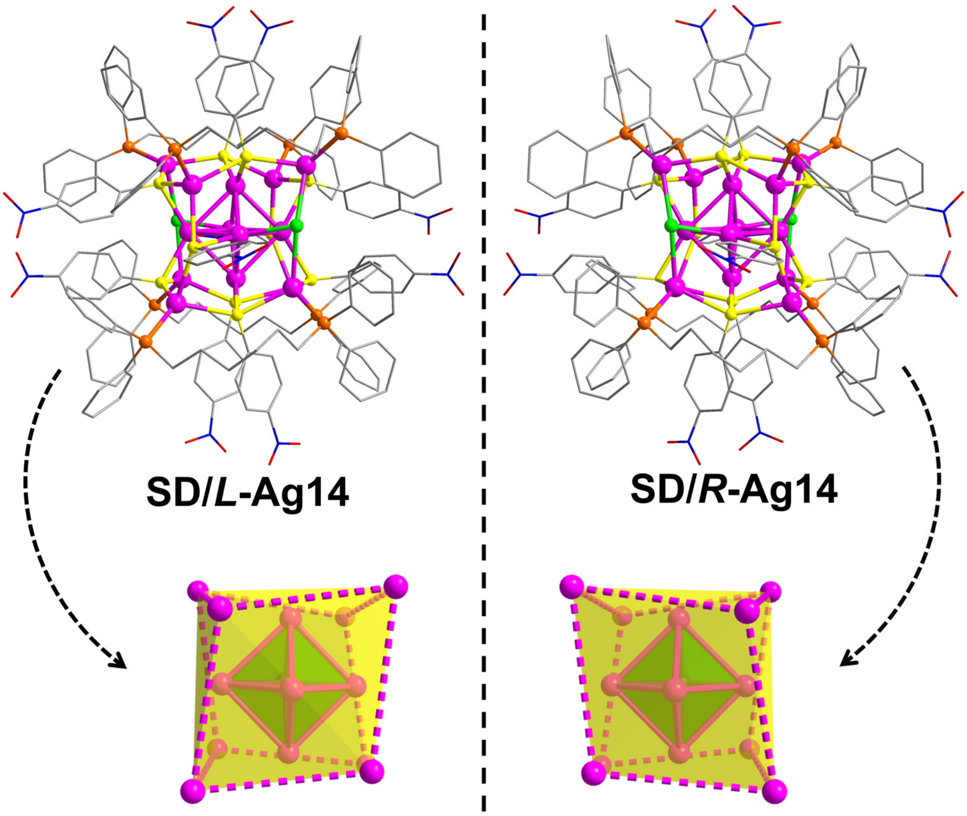

Supplementary Figure 6: Structures of enantiomers of SD/*rac*-Ag14a. The crystal structure (top) and face-rotating (bottom) of the **SD/*L*-Ag14** and **SD/*R*-Ag14**. Color labels: pink, Ag; yellow, S; orange, P; green, Cl; gray, C; blue, N; red, O. All H atoms are omitted for clarity.

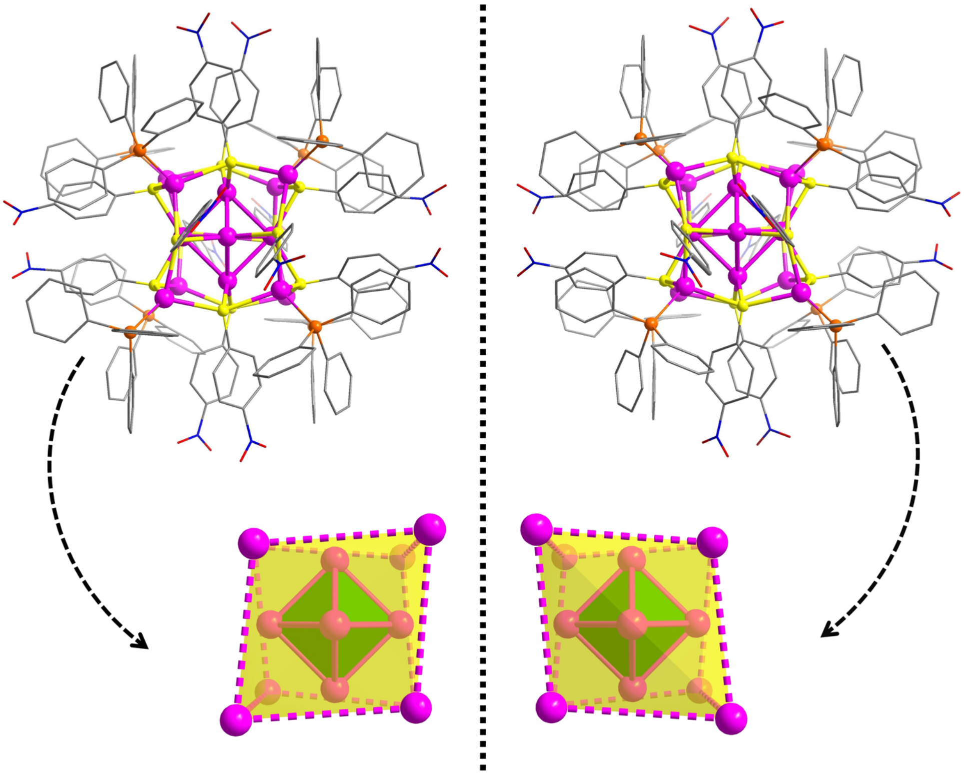

Supplementary Figure 7: Structures of enantiomers of SD/*rac*-Ag14b.The crystal structure (top) and face-rotating (bottom) of enantiomers in the unit cell of the **SD/*rac*-Ag14b**. Color labels: pink, Ag; yellow, S; orange, P; gray, C; blue, N; red, O. All H atoms are omitted for clarity.

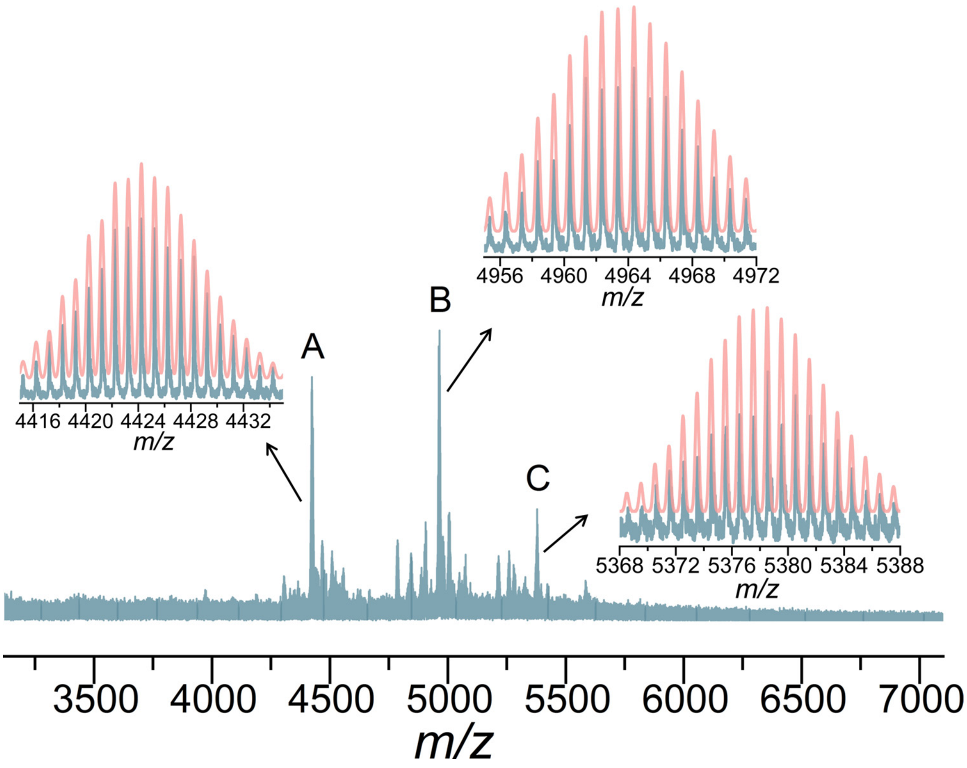

Supplementary Figure 8: Electrospray ionization mass spectrometry study of SD/*L*-Ag14 and SD/*R*-Ag14. Positive-ion mode electrospray ionization mass spectrometry (ESI-MS) of racemic conglomerates **SD/*L*-Ag14** and **SD/*R*-Ag14** dissolved in MeOH/CH_2Cl_2. Insets: Zoom-in ESI-MS of experimental (blue line) and simulated (red line) for each labeled species.

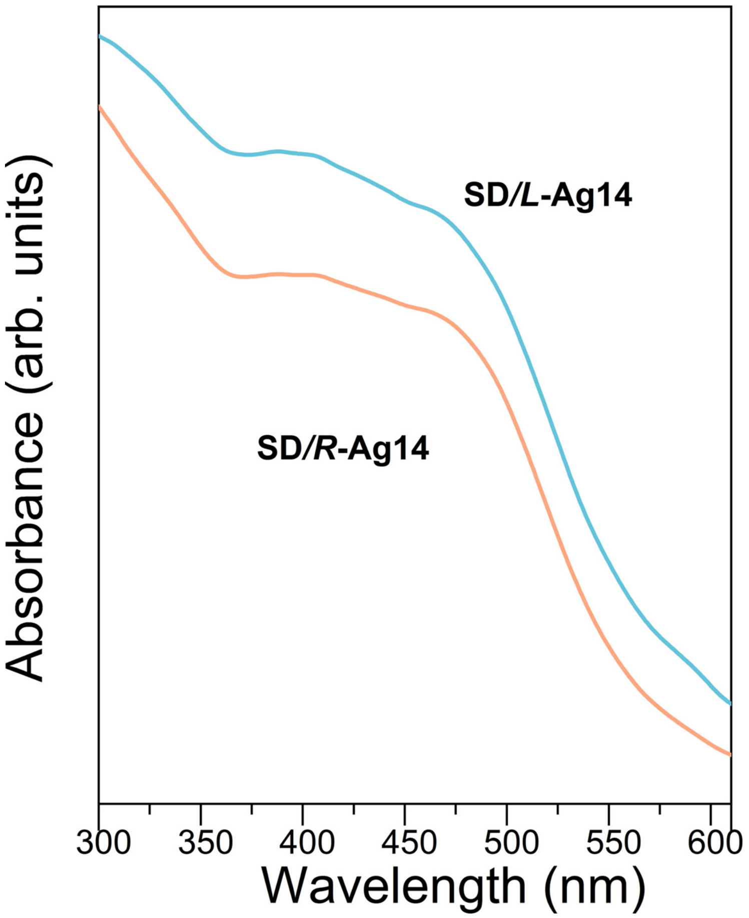

Supplementary Figure 9: The absorption spectra of SD/*L*-Ag14 and SD/*R*-Ag14. The absorption spectra of **SD/*L*-Ag14** (blue solid line) and **SD/*R*-Ag14** (orange solid line) collected along with the circular dichroism (CD) spectra measurement in the solid state.

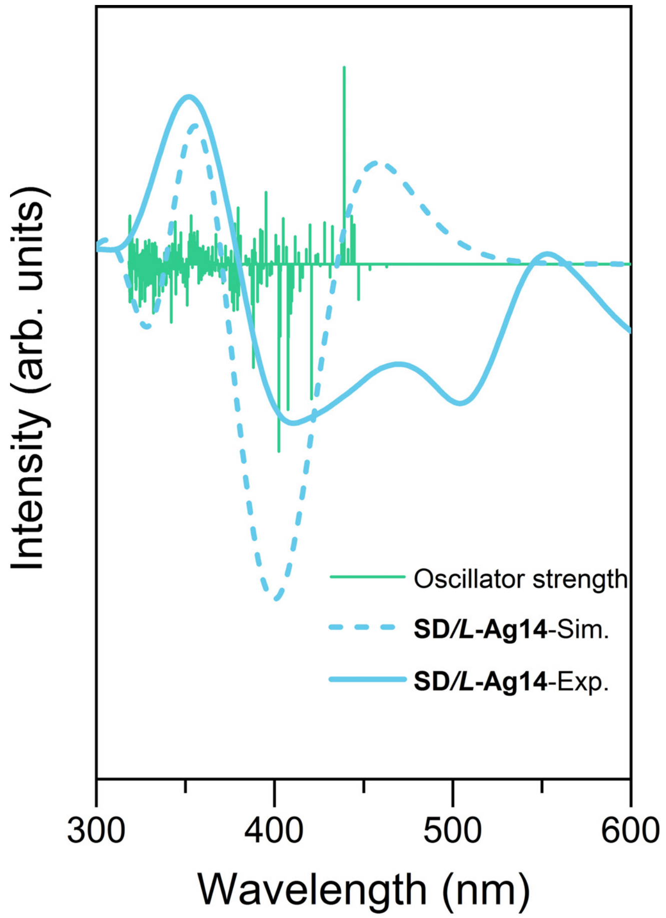

Supplementary Figure 10: The simulated and experimental circular dichroism spectra of SD/*L*-Ag14 nanocluster. Simulated (dashed line) and experimental (solid lines) circular dichroism spectra of **SD/*L*-Ag14** nanocluster. The calculated pattern was red-shift offset by 40 nm for better comparison.

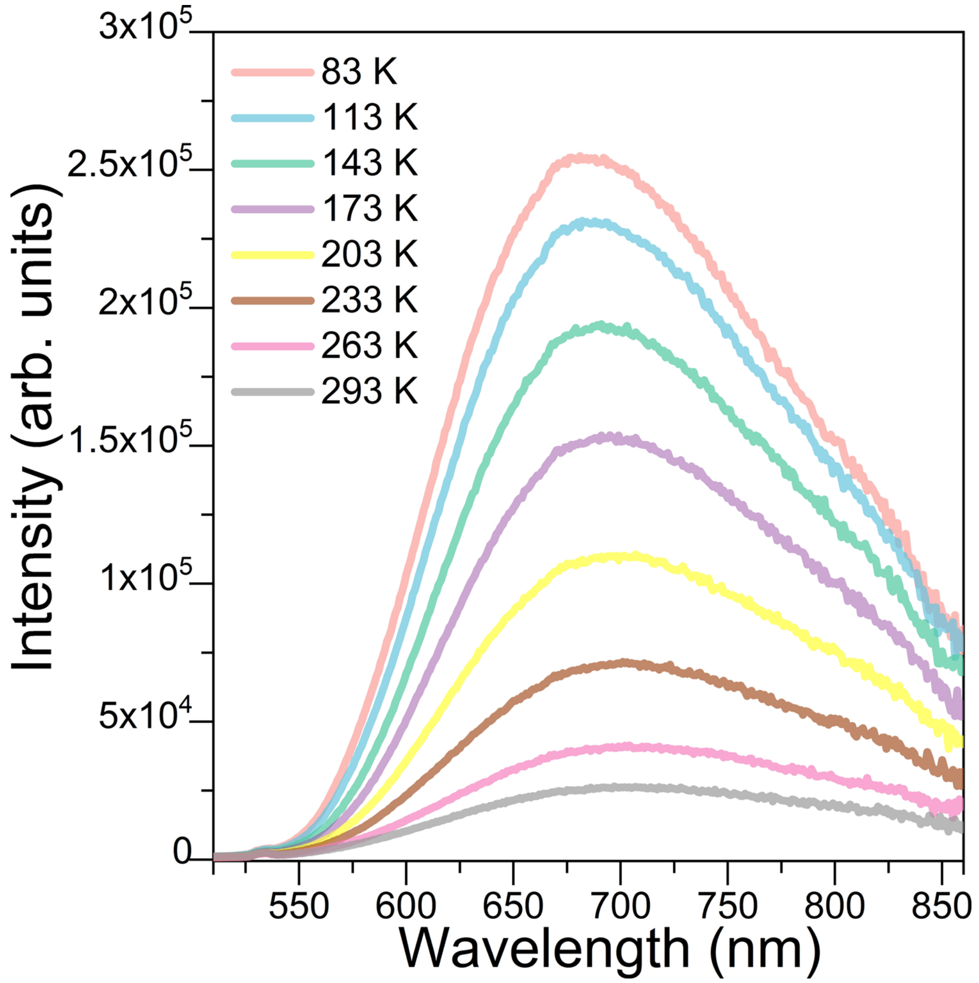

Supplementary Figure 11: Luminescence spectra of SD/*rac*-Ag14a. Variable-temperature emission spectra of **SD/*rac*-Ag14a** in the solid state from 83 to 293 K (λ_{ex}=365 nm).

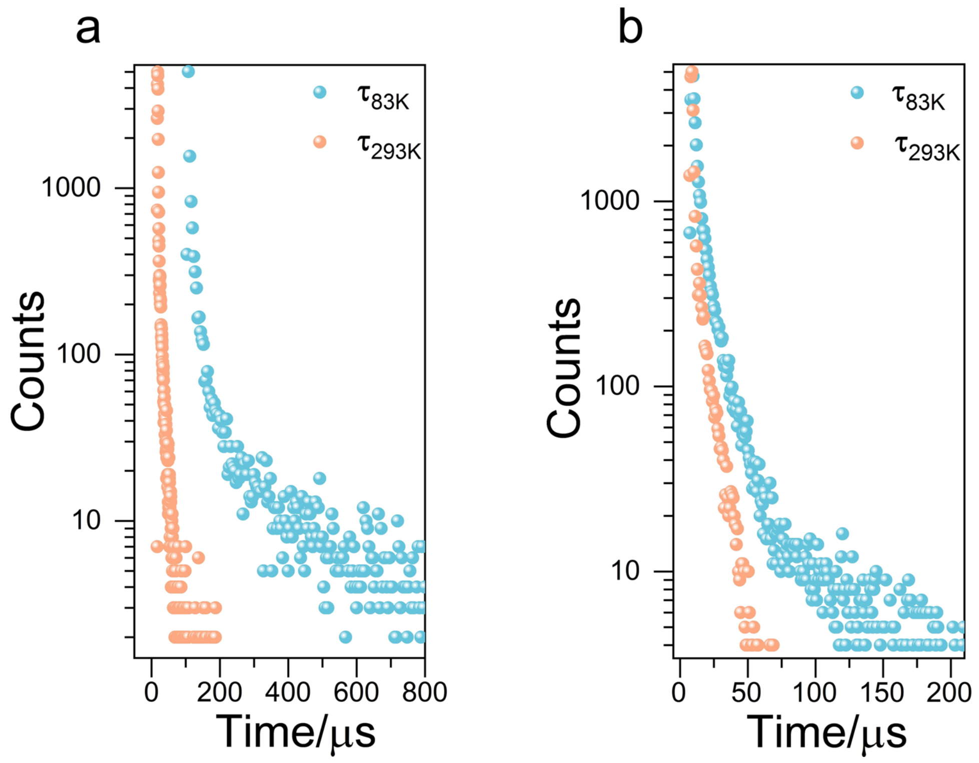

Supplementary Figure 12: Luminescence lifetimes of SD/Ag14. a Luminescence lifetime of **SD/*rac*-Ag14a** at 83 K and 293K. **b** Luminescence lifetime of racemic conglomerates **SD/*L*-Ag14** and **SD/*R*-Ag14** at 83 K and 293K.

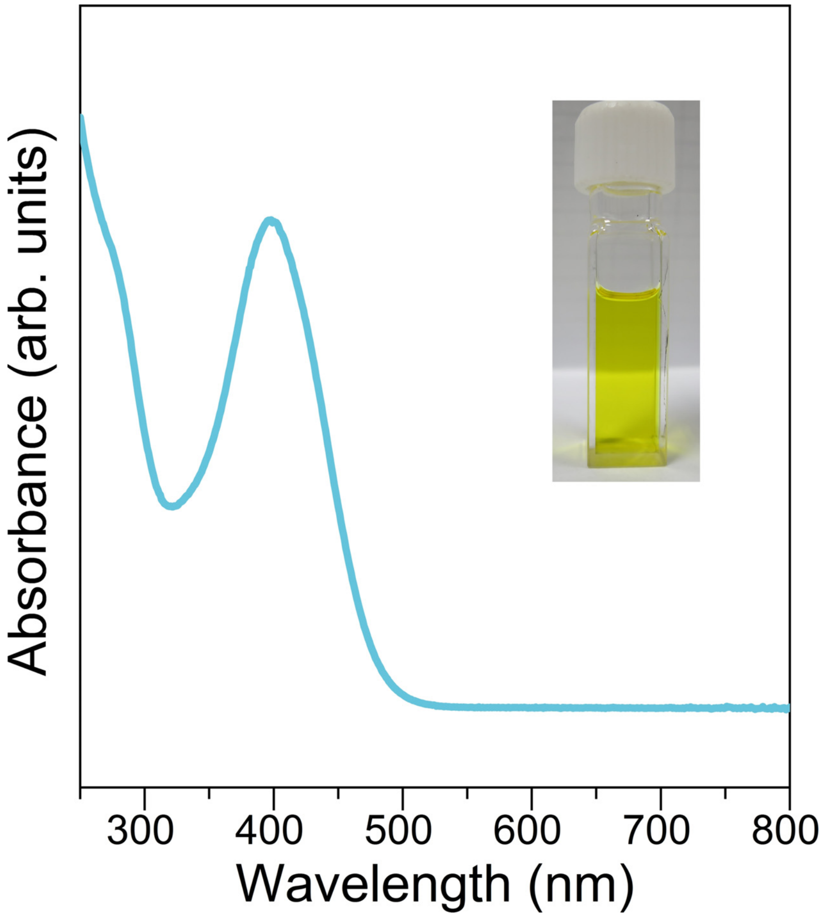

Supplementary Figure 13: The absorption spectrum of SD/Ag14. The absorption spectrum of **SD/*rac*-Ag14a**, **SD/*L*-Ag14** and **SD/*R*-Ag14** dissolved in CH_2Cl_2, respectively. Insets: the photograph of the solution of **SD/*rac*-Ag14a**, **SD/*L*-Ag14** and **SD/*R*-Ag14** dissolved in CH_2Cl_2, respectively.

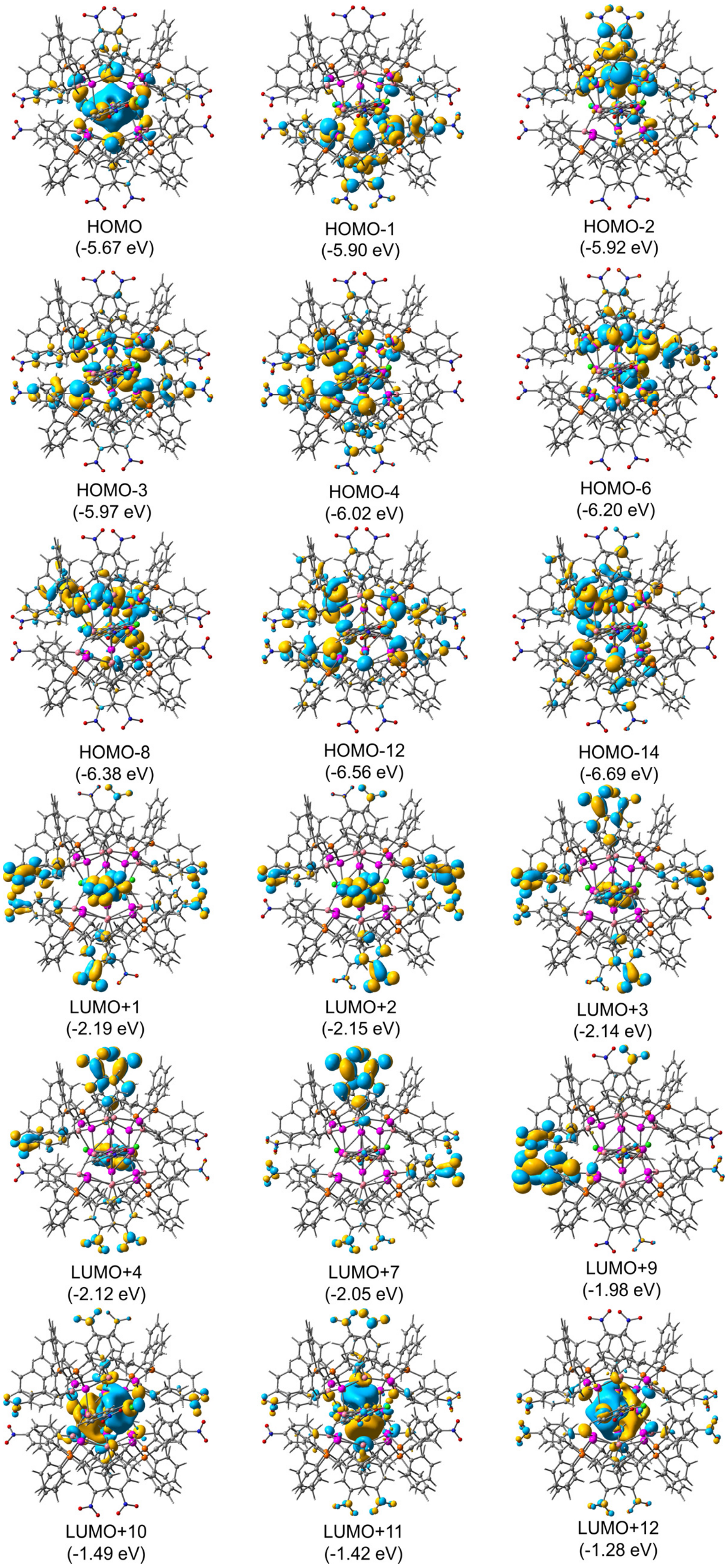

Supplementary Figure 14: Selected calculated orbitals of SD/*L*-Ag14. The selected calculated HOMO and LUMO orbitals of **SD/*L*-Ag14**.

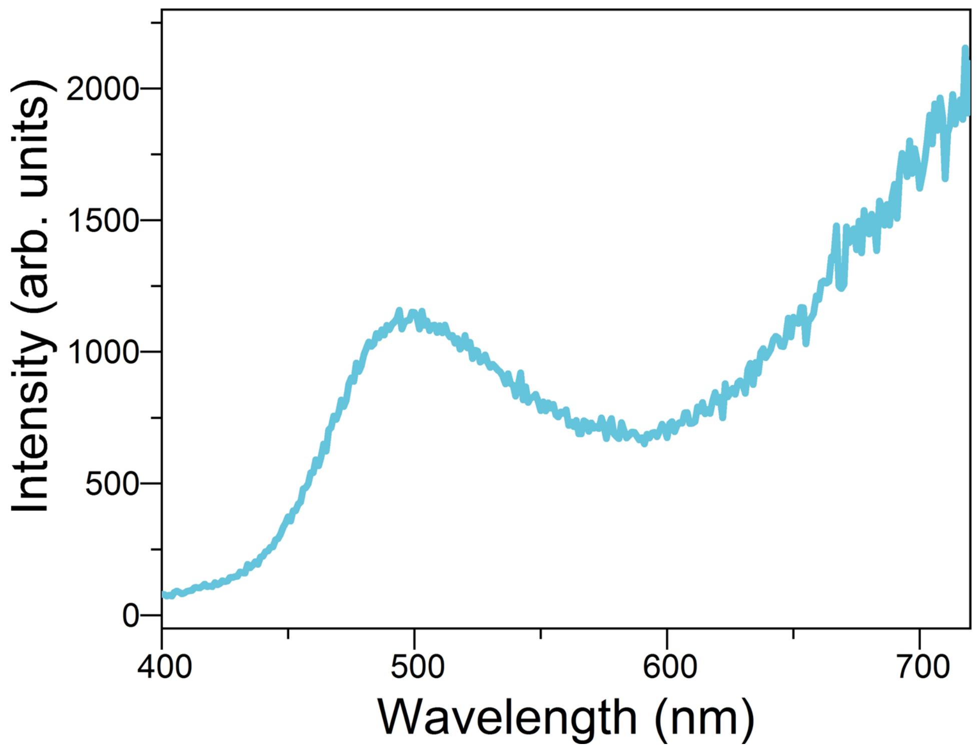

Supplementary Figure 15: Emission spectrum of SD/Ag14. Emission spectrum of **SD/*rac*-Ag14a**, **SD/*L*-Ag14** and **SD/*R*-Ag14** dissolved in CH_2Cl_2 in excitation of 365 nm at 293K, respectively.

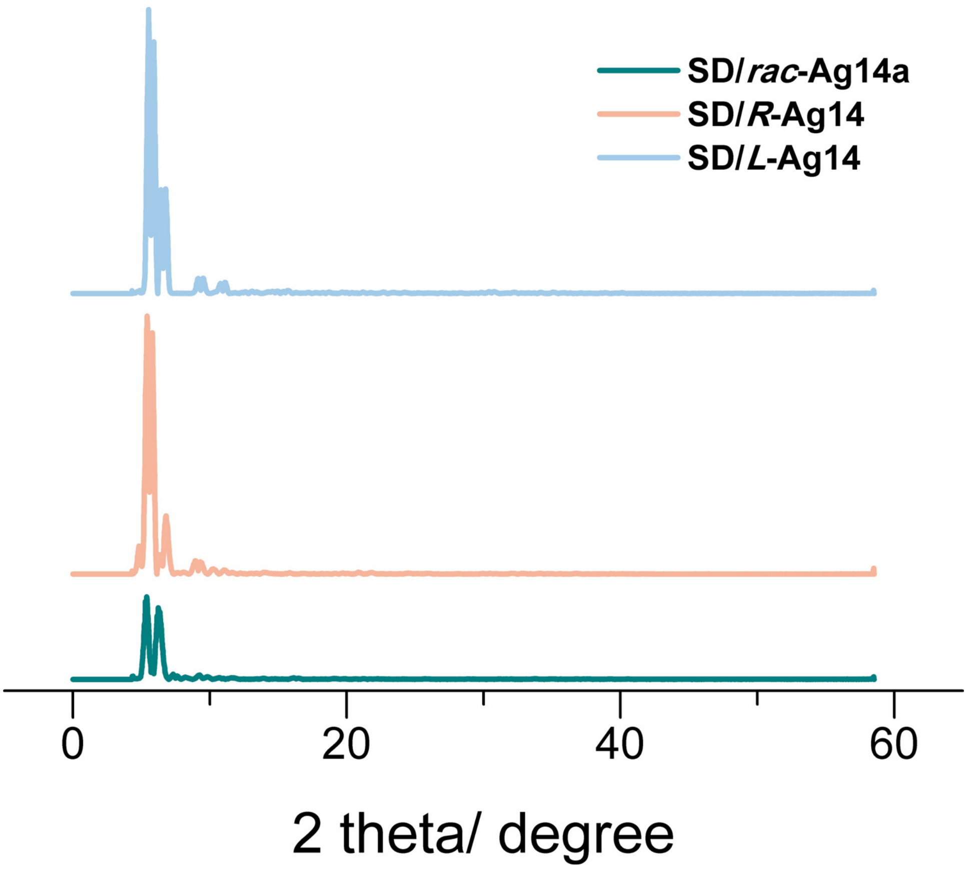

Supplementary Figure 16: The powder X-ray diffraction patterns of SD/Ag14. The powder X-ray diffraction patterns of **SD/*rac*-Ag14a** (green solid line), **SD/*R*-Ag14** (orange solid line) and **SD/*L*-Ag14** (blue solid line).

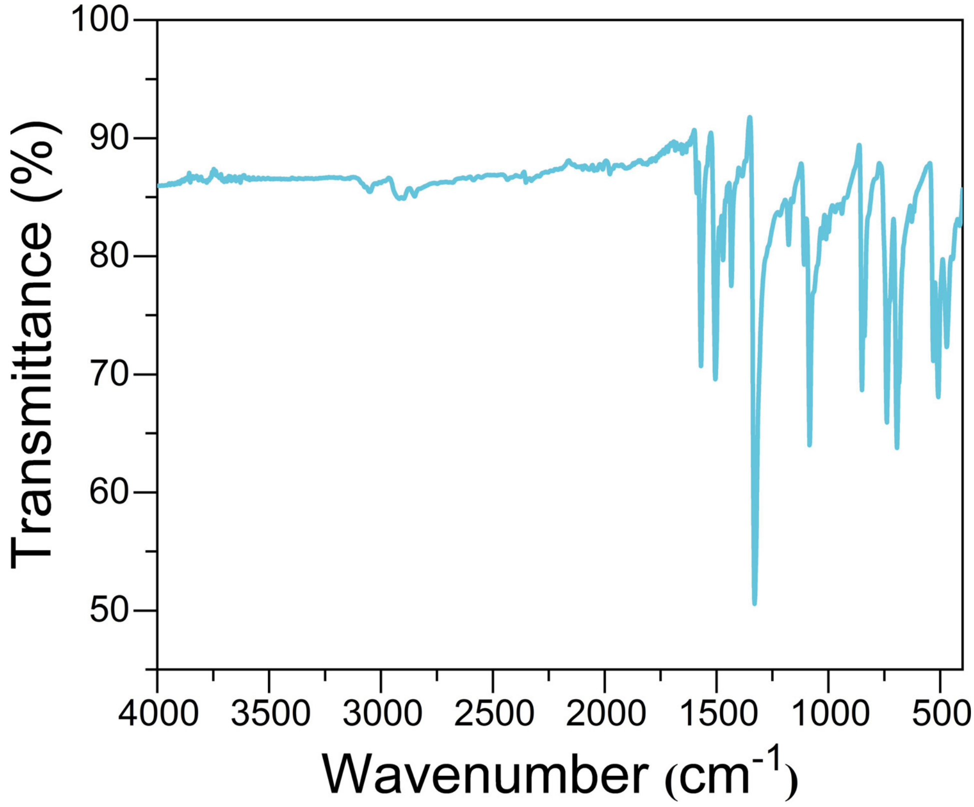

Supplementary Figure 17: The infrared spectroscopy (IR) spectrum of SD/Ag14.

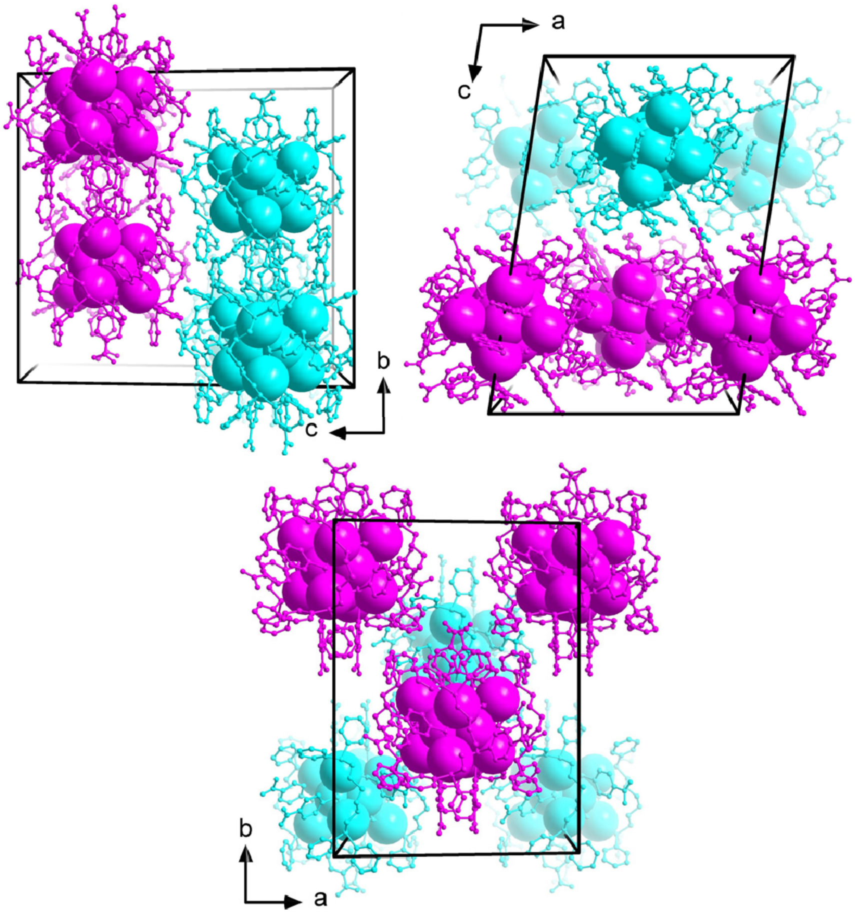

Supplementary Figure 18: Packing structure of SD/*rac*-Ag14a. The molecular packing of **SD/*rac*-Ag14a** in the unit cell viewed along *a*, *b* and *c* axis.

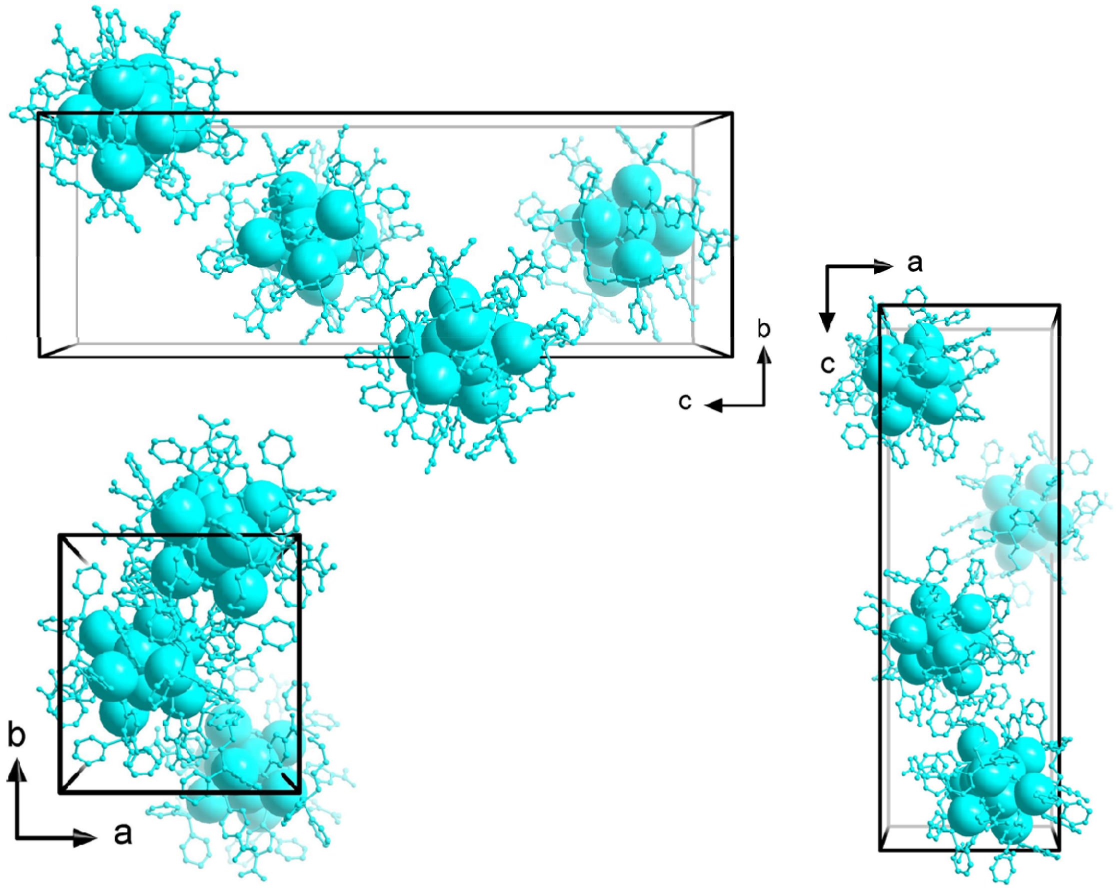

Supplementary Figure 19: Packing structure of SD/*L*-Ag14. The molecular packing of **SD/*L*-Ag14** in the unit cell viewed along *a*, *b* and *c* axis.

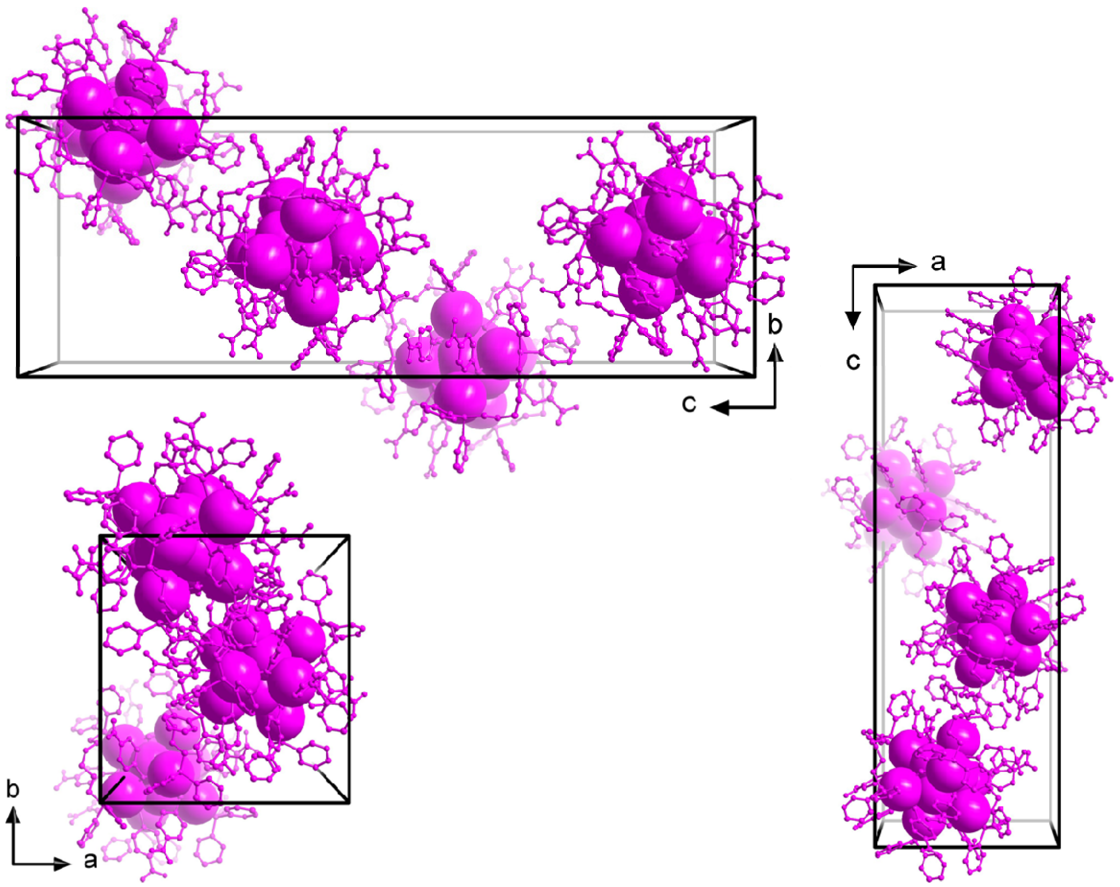

Supplementary Figure 20: Packing structure of SD/*R*-Ag14. The molecular packing of **SD/*R*-Ag14** in the unit cell viewed along *a*, *b* and *c* axis.

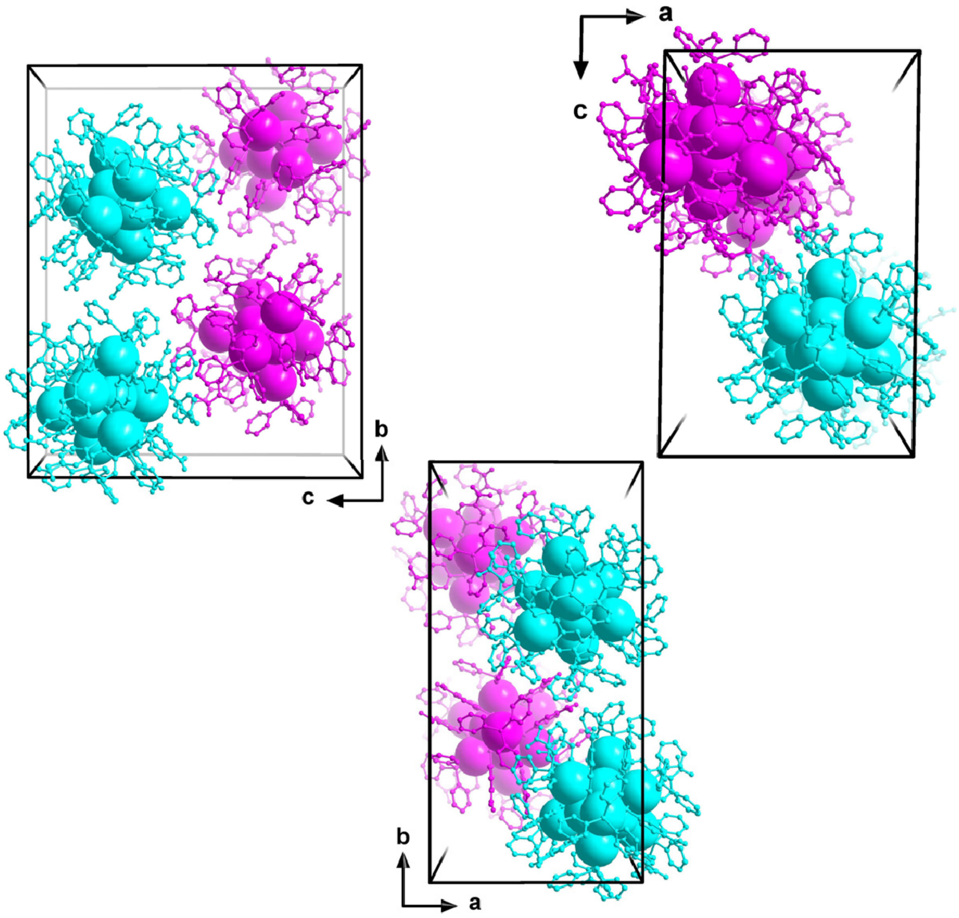

Supplementary Figure 21: Packing structure of SD/*rac*-Ag14b. The molecular packing of **SD/*rac*-Ag14b** in the unit cell viewed along *a*, *b* and *c* axis.

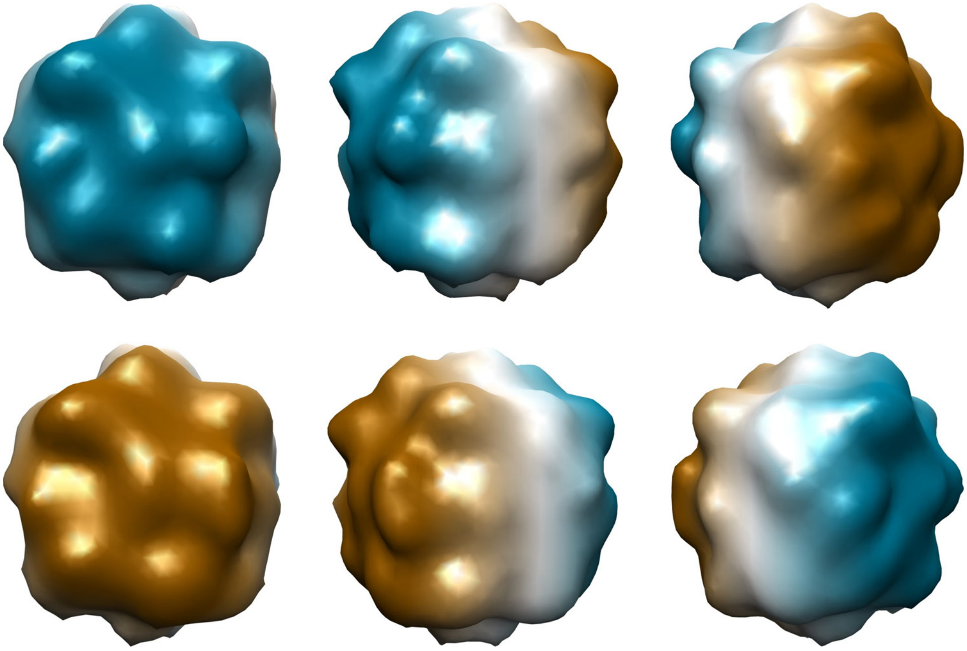

Supplementary Figure 22: The surface calculation of SD/*rac*-Ag14a. The surface of **SD/*rac*-Ag14a** calculated via 3V Volume Assessor program[10] by rolling a virtual probe (1 Å) on the surface viewed along six different orientations.

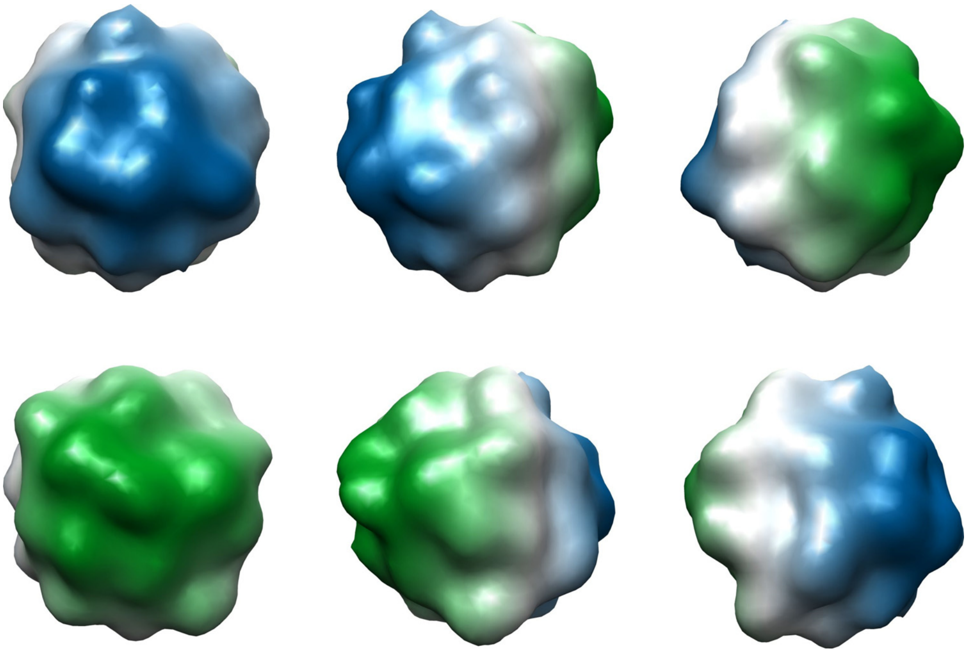

Supplementary Figure 23: The surface calculation of SD/*L*-Ag14. The surface of **SD/*L*-Ag14** calculated via 3V Volume Assessor program[10] by rolling a virtual probe (1 Å) on the surface viewed along six different orientations.

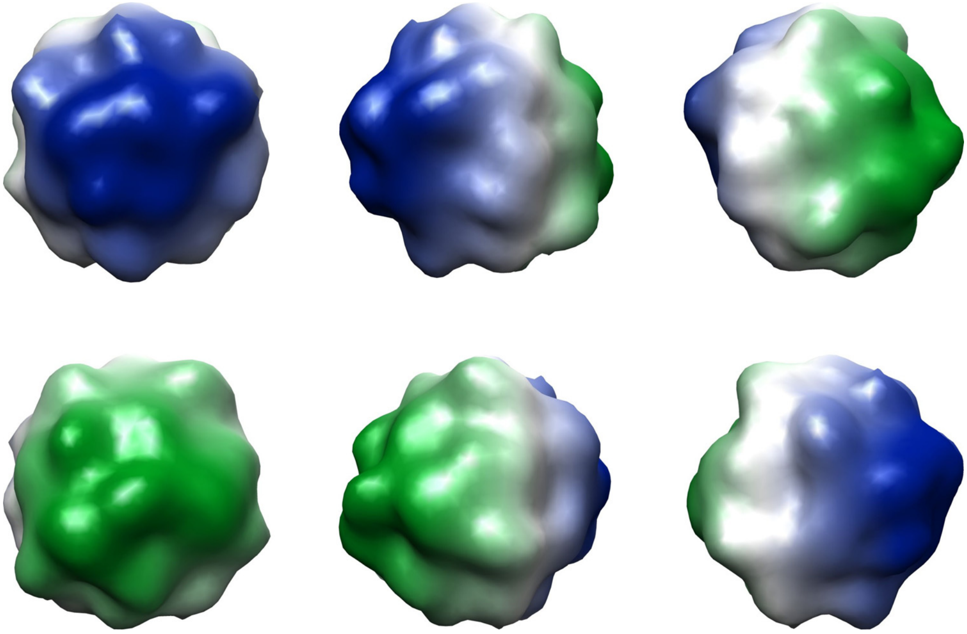

Supplementary Figure 24: The surface calculation of SD/*R*-Ag14. The surface of **SD/*R*-Ag14** calculated via 3V Volume Assessor program[10] by rolling a virtual probe (1 Å) on the surface viewed along six different orientations.

Supplementary Table 1: Crystal data collection and structure refinement for **SD/*rac*-Ag14a**, **SD/*rac*-Ag14b**, **SD/*L*-Ag14** and **SD/*R*-Ag14**.

Compound	**SD/*rac*-Ag14a**	**SD/*rac*-Ag14b**
Empirical formula	$C_{180}H_{168}Ag_{14}Cl_2N_{10}O_{20}P_8S_{10}$	$C_{216}H_{168}Ag_{14}N_{12}O_{24}P_8S_{12}$
Formula weight	4940.68	5458.27
Temperature/K	100.01(13)	173.00(10)
Crystal system	monoclinic	monoclinic
Space group	$C2/c$	$P2_1/n$
a/Å	22.4673(7)	19.8860(5)
b/Å	30.1820(9)	39.3231(11)
c/Å	32.6375(8)	31.7829(8)
α/°	90.00	90
β/°	98.804(3)	90.837(2)
γ/°	90.00	90
Volume/Å^3	21871.0(11)	24850.9(11)
Z	4	4
$\rho_{calc}g/cm^3$	1.500	1.459
μ/mm^{-1}	11.927	10.645
F(000)	9800.0	10840.0
Crystal size/mm^3	0.06 × 0.05 × 0.04	0.06 × 0.05 × 0.05
Radiation	Cu $K\alpha$ (λ = 1.54184)	Cu $K\alpha$ (λ = 1.54184)
2Θ range for data collection/°	5.34 to 134.16	5.562 to 134.16
Index ranges	$-26 \le h \le 26$, $-36 \le k \le 31$, $-38 \le l \le 38$	$-23 \le h \le 22$, $-46 \le k \le 46$, $-37 \le l \le 19$
Reflections collected	67936	153057
Independent reflections	19263 [R_{int} = 0.0623, R_{sigma} = 0.0490]	43097 [R_{int} = 0.1063, R_{sigma} = 0.0899]
Data/restraints/parameters	19263/192/1192	43097/1546/2179
Goodness-of-fit on F^2	1.116	1.530
Final R indexes [I>=2σ (I)]	R_1 = 0.0904, wR_2 = 0.2526	R_1 = 0.1503, wR_2 = 0.4167
Final R indexes [all data]	R_1 = 0.1091, wR_2 = 0.2672	R_1 = 0.1831, wR_2 = 0.4343
Largest diff. peak/hole / e Å^{-3}	2.03/-1.57	3.13/-2.13

Compound	**SD/*L*-Ag14**	**SD/*R*-Ag14**
Empirical formula	$C_{182}H_{171}Ag_{14}Cl_2N_{11}O_{20}P_8S_{10}$	$C_{182}H_{171}Ag_{14}Cl_2N_{11}O_{20}P_8S_{10}$
Formula weight	4981.73	4981.73
Temperature/K	173.00(10)	173.00(10)
Crystal system	orthorhombic	orthorhombic
Space group	$P2_12_12_1$	$P2_12_12_1$
a/Å	18.6072(3)	18.5908(4)
b/Å	19.9937(3)	19.9791(3)
c/Å	56.7004(6)	56.7024(11)
α/°	90	90
β/°	90	90
γ/°	90	90
Volume/Å^3	21094.1(5)	21060.8(7)
Z	4	4
$\rho_{calc} g/cm^3$	1.569	1.571
μ/mm^{-1}	12.373	12.392
F(000)	9888.0	9888.0
Crystal size/mm^3	0.4 × 0.4 × 0.3	0.44 × 0.42 × 0.3
Radiation	Cu $K\alpha$ (λ = 1.54184)	Cu $K\alpha$ (λ = 1.54184)
2Θ range for data collection/°	4.998 to 154.154	5.412 to 153.844
Index ranges	$-23 \le h \le 16$, $-25 \le k \le 25$, $-71 \le l \le 51$	$-19 \le h \le 23$, $-25 \le k \le 24$, $-70 \le l \le 52$
Reflections collected	104498	119196
Independent reflections	40764 [R_{int} = 0.0900, R_{sigma} = 0.0884]	41855 [R_{int} = 0.1685, R_{sigma} = 0.1264]
Data/restraints/parameters	40764/1142/2261	41855/1294/2245
Goodness-of-fit on F^2	1.059	1.154
Final R indexes [I>=2σ (I)]	R_1 = 0.0903, wR_2 = 0.2385	R_1 = 0.1123, wR_2 = 0.2751
Final R indexes [all data]	R_1 = 0.0955, wR_2 = 0.2430	R_1 = 0.1229, wR_2 = 0.2969
Largest diff. peak/hole / e Å^{-3}	2.36/-2.70	2.60/-3.01
Flack parameter	0.013(6)	0.002(10)

Supplementary Table 2: Selected bond lengths (Å) and angles (º) for **SD/*rac*-Ag14a**, **SD/*rac*-Ag14b**, **SD/*L*-Ag14** and **SD/*R*-Ag14**.

**SD/*rac*-Ag14a**			
Ag1—Ag3^{1}	2.8213(12)	Ag4—Ag6^{1}	3.3677(14)
Ag1—Ag3	2.8213(12)	Ag4—S4	2.515(3)
Ag1—Ag4	2.8394(11)	Ag4—S2	2.516(3)
Ag1—Ag4^{1}	2.8394(11)	Ag5—Cl1	2.673(3)
Ag1—Ag5^{1}	3.2417(9)	Ag5—S5	2.635(3)
Ag1—Ag5	3.2417(9)	Ag5—S2	2.657(4)
Ag1—S5	2.522(3)	Ag5—P1	2.54(4)
Ag1—S5^{1}	2.522(3)	Ag6—Ag4^{1}	3.3677(13)
Ag2—Ag3^{1}	2.8070(13)	Ag6—S4^{1}	2.679(3)
Ag2—Ag3	2.8070(13)	Ag6—S1	2.647(3)
Ag2—Ag4^{1}	2.8521(12)	Ag6—S3^{1}	2.656(4)
Ag2—Ag4	2.8521(12)	Ag6—P5	2.448(4)
Ag2—Ag6^{1}	3.3671(11)	Ag7—S1	2.830(3)
Ag2—Ag6	3.3671(11)	Ag7—S3	2.542(4)
Ag2—S3	2.522(3)	Ag7—S2	2.602(4)
Ag2—S3^{1}	2.522(3)	Ag7—P4	2.422(5)
Ag3—Ag4^{1}	2.8002(11)	Ag8—S4	2.613(3)
Ag3—Ag4	2.8054(12)	Ag8—Cl1^{1}	2.748(3)
Ag3—Ag5	3.1082(12)	Ag8—S5	2.554(3)
Ag3—Ag6	3.1212(13)	Ag8—P3	2.404(4)
Ag3—S1	2.565(3)	S4—Ag6^{1}	2.679(3)
Ag3—Cl1	2.566(3)	Cl1—Ag8^{1}	2.748(3)
Ag4—Ag3^{1}	2.8001(11)	S3—Ag6^{1}	2.656(4)
Ag4—Ag5	3.2390(14)	P5—Ag6—S1	117.20(12)
S5—Ag1—S5^{1}	118.27(15)	P5—Ag6—S3^{1}	117.08(12)
S3—Ag2—S3^{1}	120.94(16)	S3—Ag7—S1	97.25(10)
S1—Ag3—Cl1	118.60(11)	S3—Ag7—S2	101.50(11)
S4—Ag4—S2	116.72(11)	S2—Ag7—S1	87.18(10)
S5—Ag5—Cl1	106.42(10)	P4—Ag7—S1	108.28(15)
S5—Ag5—S2	106.94(11)	P4—Ag7—S3	131.33(16)
S2—Ag5—Cl1	116.98(10)	P4—Ag7—S2	120.03(18)
P1—Ag5—Cl1	114.0(8)	S4—Ag8—Cl1^{1}	87.33(9)
P1—Ag5—S5	113.8(11)	S5—Ag8—S4	99.68(11)
P1—Ag5—S2	98.6(7)	S5—Ag8—Cl1^{1}	95.01(10)
S1—Ag6—S4^{1}	116.94(10)	P3—Ag8—S4	121.86(14)

S1—Ag6—S3[1]	104.61(11)	P3—Ag8—Cl1[1]	113.95(13)
S3[1]—Ag6—S4[1]	102.78(11)	P3—Ag8—S5	128.79(13)
P5—Ag6—S4[1]	97.79(12)		
Symmetry code: (1) -*x*,+*y*,1/2-*z*			

**SD/*rac*-Ag14b**			
Ag1—Ag7	2.8246(18)	Ag8—Ag10	3.290(2)
Ag1—Ag8	2.8265(19)	Ag8—Ag11	2.8182(19)
Ag1—Ag9	2.8264(17)	Ag8—Ag13	3.2830(19)
Ag1—Ag12	2.8344(18)	Ag8—S4	2.532(5)
Ag1—Ag13	3.2718(18)	Ag8—S12	2.524(5)
Ag1—Ag14	3.3032(19)	Ag9—Ag11	2.8118(19)
Ag1—S1	2.527(5)	Ag9—Ag12	2.8344(18)
Ag1—S9	2.522(5)	Ag9—Ag13	3.2597(19)
Ag2—S5	2.606(5)	Ag9—S8	2.524(5)
Ag2—S6	2.626(5)	Ag9—S10	2.524(6)
Ag2—S11	2.693(6)	Ag10—Ag11	3.304(2)
Ag2—P5	2.425(5)	Ag10—S3	2.642(5)
Ag3—S3	2.647(5)	Ag10—S4	2.663(6)
Ag3—S9	2.661(6)	Ag10—S5	2.656(5)
Ag3—S12	2.657(5)	Ag10—P7	2.503(6)
Ag3—P6	2.436(6)	Ag11—Ag12	2.8255(19)
Ag4—Ag9	3.233(2)	Ag11—S2	2.533(5)
Ag4—Ag11	3.241(2)	Ag11—S5	2.519(5)
Ag4—Ag12	3.2607(19)	Ag12—Ag14	3.3255(19)
Ag4—S2	2.686(6)	Ag12—S7	2.531(5)
Ag4—S10	2.696(5)	Ag12—S11	2.524(5)
Ag4—S11	2.677(5)	Ag13—S1	2.652(5)
Ag4—P8	2.529(6)	Ag13—S8	2.685(5)
Ag5—S1	2.659(5)	Ag13—S12	2.694(5)
Ag5—S7	2.607(5)	Ag13-P1	2.519(5)
Ag5—S10	2.691(5)	Ag14—S6	2.661(5)
Ag5—P4	2.436(6)	Ag14—S7	2.669(5)
Ag6—S2	2.666(6)	Ag14—S9	2.672(5)
Ag6—S4	2.648(6)	Ag14—P2	2.503(6)
Ag6—S8	2.651(5)	S9—Ag1—S1	119.12(16)
Ag6—P3	2.444(6)	S5—Ag2—S6	95.83(16)
Ag7—Ag8	2.8358(19)	S5—Ag2—S11	91.58(16)
Ag7—Ag10	3.276(2)	S6—Ag2—S11	93.93(16)
Ag7—Ag11	2.8523(18)	P5—Ag2—S5	124.4(2)
Ag7—Ag12	2.834(2)	P5—Ag2—S6	121.40(18)
Ag7—Ag14	3.301(2)	P5—Ag2—S11	121.8(2)
Ag7—S3	2.522(5)	S3—Ag3—S9	91.99(17)

Ag7—S6	2.545(5)	S3—Ag3—S12	94.40(16)
Ag8-Ag9	2.844(2)	S12—Ag3—S9	91.75(16)
P6—Ag3—S3	122.8(2)	S12—Ag8—S4	118.30(18)
P6—Ag3—S9	123.3(2)	S8—Ag9—S10	119.57(18)
P6—Ag3—S12	123.8(2)	S3—Ag10—S4	108.06(16)
S2—Ag4—S10	109.45(17)	S3—Ag10—S5	107.79(17)
S11—Ag4—S2	108.23(17)	S5—Ag10—S4	108.29(17)
S11—Ag4—S10	107.50(16)	P7—Ag10—S3	109.2(2)
P8—Ag4—S2	107.4(2)	P7—Ag10—S4	111.50(19)
P8—Ag4—S10	113.75(19)	P7—Ag10—S5	111.89(19)
P8—Ag4—S11	110.43(19)	S5—Ag11—S2	119.68(17)
S1—Ag5—S10	91.78(16)	S11—Ag12—S7	120.03(17)
S7—Ag5—S1	95.16(15)	S1—Ag13—S8	109.27(15)
S7—Ag5—S10	91.99(16)	S1—Ag13—S12	107.25(15)
P4—Ag5—S1	120.71(18)	S8—Ag13—S12	108.46(16)
P4—Ag5—S7	124.7(2)	P1—Ag13—S1	110.57(16)
P4—Ag5—S10	123.9(2)	P1—Ag13—S8	109.86(16)
S4—Ag6—S2	94.28(17)	P1—Ag13—S12	111.35(17)
S4—Ag6—S8	95.70(16)	S6—Ag14—S7	106.86(16)
S8—Ag6—S2	91.01(16)	S6—Ag14—S9	106.77(17)
P3—Ag6—S2	124.9(2)	S7—Ag14—S9	108.39(15)
P3—Ag6—S4	118.1(2)	P2—Ag14—S6	114.29(19)
P3—Ag6—S8	124.95(19)	P2—Ag14—S7	108.9(2)
S3—Ag7—S6	118.57(18)	P2—Ag14—S9	111.41(19)

SD/*L*-Ag14			
Ag1—Ag2	2.8068(19)	Ag9—P4	2.467(5)
Ag1—Ag3	2.7974(19)	Ag9—S6	2.626(5)
Ag1—Ag4	2.8063(19)	Ag9—S7	2.690(5)
Ag1—Ag5	2.8082(18)	Ag9—S9	2.665(5)
Ag1—Ag7	3.1841(19)	Ag10—Cl1	2.806(5)
Ag1—Ag11	3.0963(19)	Ag10—P2	2.410(6)
Ag1—Cl1	2.613(5)	Ag10—S5	2.615(5)
Ag1—S8	2.487(5)	Ag10—S7	2.530(5)
Ag2—Ag3	2.8529(18)	Ag11—P8	2.455(5)
Ag2—Ag5	2.8937(18)	Ag11—S1	2.666(5)
Ag2—Ag6	2.7884(19)	Ag11—S5	2.667(5)
Ag2—S1	2.537(5)	Ag11—S8	2.637(5)
Ag2—S4	2.503(5)	Ag12—P3	2.426(5)
Ag3—Ag4	2.8353(18)	Ag12—S2	2.693(5)
Ag3—Ag6	2.8132(18)	Ag12—S6	2.690(5)
Ag3—S5	2.498(5)	Ag12—S10	2.584(6)
Ag3—S9	2.510(5)	Ag13—Cl2	2.820(5)
Ag4—Ag5	2.8606(18)	Ag13—P1	2.54(3)
Ag4—Ag6	2.8174(19)	Ag13—S1	2.530(5)
Ag4—Ag7	3.281(2)	Ag13—S9	2.602(5)
Ag4—S7	2.548(5)	Ag14—P7	2.426(5)
Ag4—S10	2.522(5)	Ag14—S3	2.677(5)
Ag5—Ag6	2.7993(18)	Ag14—S4	2.586(5)
Ag5—Ag7	3.280(2)	Ag14—S8	2.701(5)
Ag5—Ag8	3.376(2)	S8—Ag1—Cl1	120.42(16)
Ag5—S2	2.526(5)	S4—Ag2—S1	119.88(16)
Ag5—S3	2.520(5)	S5—Ag3—S9	118.85(16)
Ag6—Ag8	3.221(2)	S10—Ag4—S7	118.09(16)
Ag6—Ag9	3.1182(19)	S3—Ag5—S2	116.64(16)
Ag6—Cl2	2.616(5)	S6—Ag6—Cl2	118.24(16)
Ag6—S6	2.510(5)	P5—Ag7—Cl1	109.90(17)
Ag7—Cl1	2.734(4)	P5—Ag7—S3	103.75(17)
Ag7—P5	2.464(5)	P5—Ag7—S10	119.49(18)
Ag7—S3	2.642(5)	S3—Ag7—Cl1	118.89(15)
Ag7—S10	2.688(5)	S3—Ag7—S10	105.92(16)
Ag8—Cl2	2.657(5)	S10—Ag7—Cl1	99.75(15)
Ag8—P6	2.447(6)	Cl2—Ag8—S4	96.60(15)

Ag8—S2	2.634(5)	P6—Ag8—Cl2	112.49(18)
Ag8—S4	2.689(5)	P6—Ag8—S2	107.40(17)
P6—Ag8—S4	119.01(17)	S8—Ag11—S5	117.61(15)
S2—Ag8—Cl2	117.75(15)	P3—Ag12—S2	118.18(17)
S2—Ag8—S4	103.55(15)	P3—Ag12—S6	117.49(18)
P4—Ag9—S6	116.50(17)	P3—Ag12—S10	126.37(19)
P4—Ag9—S7	116.30(18)	S6—Ag12—S2	86.35(15)
P4—Ag9—S9	106.17(18)	S10—Ag12—S2	98.19(16)
S6—Ag9—S7	101.51(15)	S10—Ag12—S6	101.84(15)
S6—Ag9—S9	116.11(15)	P1—Ag13—Cl2	97.3(7)
S9—Ag9—S7	99.25(16)	P1—Ag13—S9	122.7(8)
P2—Ag10—Cl1	110.68(18)	S1—Ag13—Cl2	97.05(15)
P2—Ag10—S5	113.02(17)	S1—Ag13—P1	130.7(8)
P2—Ag10—S7	131.90(18)	S1—Ag13—S9	105.32(17)
S5—Ag10—Cl1	84.03(14)	S9—Ag13—Cl2	85.25(15)
S7—Ag10—Cl1	99.07(15)	P7—Ag14—S3	118.76(19)
S7—Ag10—S5	106.76(16)	P7—Ag14—S4	126.63(18)
P8—Ag11—S1	118.90(19)	P7—Ag14—S8	117.6(2)
P8—Ag11—S5	101.32(17)	S3—Ag14—S8	88.89(15)
P8—Ag11—S8	116.15(18)	S4—Ag14—S3	97.90(16)
S1—Ag11—S5	101.75(16)	S4—Ag14—S8	99.08(15)
S8—Ag11—S1	101.05(15)		

**SD/*R*-Ag14**			
Ag1—Ag2	2.7987(19)	Ag9—P6	2.459(6)
Ag1—Ag3	2.8004(19)	Ag9—S5	2.660(5)
Ag1—Ag5	2.807(2)	Ag9—S7	2.631(5)
Ag1—Ag6	3.1784(19)	Ag9—S10	2.689(5)
Ag1—Ag7	2.805(2)	Ag10—Cl2	2.662(5)
Ag1—Ag8	3.1035(19)	Ag10—P2	2.448(6)
Ag1—Cl1	2.613(5)	Ag10—S1	2.636(5)
Ag1—S6	2.498(5)	Ag10—S3	2.695(5)
Ag2—Ag4	2.8121(19)	Ag11—Cl1	2.815(5)
Ag2—Ag5	2.8519(19)	Ag11—P1	2.402(6)
Ag2—Ag7	2.837(2)	Ag11—S4	2.609(5)
Ag2—S4	2.514(5)	Ag11—S10	2.529(5)
Ag2—S5	2.506(5)	Ag12—Cl2	2.811(5)
Ag3—Ag4	2.7978(19)	Ag12—P7	2.394(7)
Ag3—Ag5	2.8953(18)	Ag12—S2	2.539(5)
Ag3—Ag6	3.274(2)	Ag12—S5	2.601(6)
Ag3—Ag7	2.8579(19)	Ag13—P5	2.422(6)
Ag3—Ag10	3.372(2)	Ag13—S1	2.685(5)
Ag3—S1	2.525(5)	Ag13—S7	2.688(5)
Ag3—S8	2.522(5)	Ag13—S9	2.582(6)
Ag4—Ag5	2.7902(19)	Ag14—P3	2.419(5)
Ag4—Ag7	2.814(2)	Ag14—S3	2.576(5)
Ag4—Ag9	3.119(2)	Ag14—S6	2.700(5)
Ag4—Ag10	3.221(2)	Ag14—S8	2.678(5)
Ag4—Cl2	2.633(5)	S6—Ag1—Cl1	120.43(17)
Ag4—S7	2.508(5)	S5—Ag2—S4	119.00(17)
Ag5—S2	2.537(5)	S8—Ag3—S1	116.74(16)
Ag5—S3	2.515(5)	S7—Ag4—Cl2	118.35(17)
Ag6—Ag7	3.279(2)	S3—Ag5—S2	119.93(16)
Ag6—Cl1	2.724(5)	P4—Ag6—Cl1	109.77(18)
Ag6—P4	2.468(5)	P4—Ag6—S8	103.68(17)
Ag6—S8	2.636(5)	P4—Ag6—S9	119.50(19)
Ag6—S9	2.696(6)	S8—Ag6—Cl1	118.95(16)
Ag7—S9	2.518(5)	S8—Ag6—S9	106.22(17)
Ag7—S10	2.546(5)	S9—Ag6—Cl1	99.59(17)
Ag8—P8	2.454(6)	S9—Ag7—S10	117.88(17)
Ag8—S2	2.651(5)	P8—Ag8—S2	118.5(2)

Ag8—S4	2.679(5)	P8—Ag8—S4	100.97(19)
Ag8—S6	2.636(5)	P8—Ag8—S6	116.50(19)
S6—Ag8—S2	101.04(16)	S10—Ag11—S4	107.05(17)
S6—Ag8—S4	117.74(16)	P7—Ag12—Cl2	101.2(3)
S2—Ag8—S4	102.05(17)	P7—Ag12—S2	131.4(2)
P6—Ag9—S5	106.52(18)	P7—Ag12—S5	120.9(2)
P6—Ag9—S7	116.27(18)	S2—Ag12—Cl2	96.84(16)
P6—Ag9—S10	116.39(19)	S2—Ag12—S5	105.17(17)
S5—Ag9—S10	99.27(17)	S5—Ag12—Cl2	85.45(15)
S7—Ag9—S5	115.83(17)	P5—Ag13—S1	118.19(19)
S7—Ag9—S10	101.58(16)	P5—Ag13—S7	117.49(18)
Cl2—Ag10—S3	96.55(16)	P5—Ag13—S9	126.2(2)
P2—Ag10—Cl2	112.39(19)	S1—Ag13—S7	86.33(16)
P2—Ag10—S1	107.41(18)	S9—Ag13—S1	98.31(17)
P2—Ag10—S3	118.68(19)	S9—Ag13—S7	102.01(17)
S1—Ag10—Cl2	118.02(16)	P3—Ag14—S3	126.26(19)
S1—Ag10—S3	103.77(16)	P3—Ag14—S6	117.6(2)
P1—Ag11—Cl1	110.73(18)	P3—Ag14—S8	118.78(18)
P1—Ag11—S4	112.45(18)	S3—Ag14—S6	99.26(16)
P1—Ag11—S10	132.14(19)	S3—Ag14—S8	98.10(16)
S4—Ag11—Cl1	84.12(15)	S8—Ag14—S6	89.05(16)
S10—Ag11—Cl1	98.95(16)		

Supplementary Table 3: SCXRD analysis of 40 crystals grown in the one beaker for one-time synthesis.

Analysis object	Number	Space group	R_1	Flack
	1(**SD/*L*-Ag14**)	$P2_12_12_1$	9.03%	0.013(6)
	2(**SD/*R*-Ag14**)	$P2_12_12_1$	11.23%	0.002(10)
	3(**SD/*L*-Ag14**)	$P2_12_12_1$	12.08%	-0.007(9)
	4(**SD/*R*-Ag14**)	$P2_12_12_1$	24.36%	0.05(3)
	5(**SD/*L*-Ag14**)	$P2_12_12_1$	16.87%	-0.01(2)
	6(**SD/*L*-Ag14**)	$P2_12_12_1$	21.04%	0.15(11)
	7(**SD/*L*-Ag14**)	$P2_12_12_1$	10.07%	0.007(11)
	8(**SD/*L*-Ag14**)	$P2_12_12_1$	12.25%	0.009(13)
	9(**SD/*R*-Ag14**)	$P2_12_12_1$	10.93%	-0.019(9)
	10(**SD/*R*-Ag14**)	$P2_12_12_1$	23.57%	0.07(2)
	11(**SD/*R*-Ag14**)	$P2_12_12_1$	9.54%	0.003(10)
	12(**SD/*R*-Ag14**)	$P2_12_12_1$	11.25%	0.011(11)
	13(**SD/*R*-Ag14**)	$P2_12_12_1$	10.57%	-0.015(9)
	14(**SD/*R*-Ag14**)	$P2_12_12_1$	8.80%	0.022(8)
	15(**SD/*L*-Ag14**)	$P2_12_12_1$	10.36%	0.007(9)
	16(**SD/*L*-Ag14**)	$P2_12_12_1$	11.25%	0.009(9)
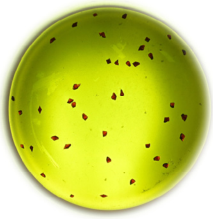	17(**SD/*L*-Ag14**)	$P2_12_12_1$	10.38%	0.026(10)
	18(**SD/*L*-Ag14**)	$P2_12_12_1$	9.34%	0.003(8)
	19(**SD/*L*-Ag14**)	$P2_12_12_1$	12.98%	-0.017(12)
	20(**SD/*R*-Ag14**)	$P2_12_12_1$	11.48%	-0.010(12)
	21(**SD/*L*-Ag14**)	$P2_12_12_1$	9.99%	0.012(10)
	22(**SD/*R*-Ag14**)	$P2_12_12_1$	10.48%	-0.020(10)
	23(**SD/*L*-Ag14**)	$P2_12_12_1$	8.61%	-0.007(9)
	24(**SD/*R*-Ag14**)	$P2_12_12_1$	8.60%	0.003(6)
	25(**SD/*R*-Ag14**)	$P2_12_12_1$	10.60%	-0.012(11)
	26(**SD/*R*-Ag14**)	$P2_12_12_1$	8.01%	0.001(8)
	27(**SD/*L*-Ag14**)	$P2_12_12_1$	10.29%	0.005(9)
	28(**SD/*L*-Ag14**)	$P2_12_12_1$	13.89%	0.02(2)
	29(**SD/*R*-Ag14**)	$P2_12_12_1$	9.76%	0.010(10)
	30(**SD/*L*-Ag14**)	$P2_12_12_1$	10.81%	0.013(13)
	31(**SD/*R*-Ag14**)	$P2_12_12_1$	10.00%	0.017(8)
	32(**SD/*L*-Ag14**)	$P2_12_12_1$	11.80%	0.007(13)
	33(**SD/*R*-Ag14**)	$P2_12_12_1$	24.61%	0.01(3)
	34(**SD/*L*-Ag14**)	$P2_12_12_1$	9.41%	0.002(9)
	35(**SD/*L*-Ag14**)	$P2_12_12_1$	12.99%	0.008(12)
	36(**SD/*R*-Ag14**)	$P2_12_12_1$	17.58%	0.02(2)
	37(**SD/*R*-Ag14**)	$P2_12_12_1$	10.18%	-0.015(12)
	38(**SD/*L*-Ag14**)	$P2_12_12_1$	17.36%	0.002(14)
	39(**SD/*L*-Ag14**)	$P2_12_12_1$	8.50%	0.002(8)
	40(**SD/*R*-Ag14**)	$P2_12_12_1$	10.04%	0.004(11)

Supplementary Table 4: Assignment of the key three species of racemic conglomerates **SD/*L*-Ag14** and **SD/*R*-Ag14** grown in the one beaker dissolved in MeOH/CH_2Cl_2 identified by ESI-MS.

**species**	**assignment**	**Exp.**	**Sim.**
A	$[Na_2Ag_{13}(pntp)_{10}(dpph)_3Cl_2]^+$	4424.2373	4424.2393
B	$[NaAg_{14}(pntp)_{10}(dpph)_4Cl_2]^+$	4964.3536	4964.3537
C	$[Na_2Ag_{14}(pntp)_{11}(dpph)_4Cl_2(CH_3CN)(CH_3OH)_5(H_2O)_2]^+$	5378.5601	5378.5189

Supplementary Table 5: Summary of reaction conditions tried in the synthesis of **SD/*rac*-Ag14a**.

CF_3COOAg /mg	dpph /mg	Hpntp /mg	Et_3N / μL	Solvent (6 mL)	Product	Single crystals size
11.0	2.3	7.8	40	Acetone:DCM = 4:2	No crystal	
	4.6				No crystal	
	6.9				No crystal	
	9.1				No crystal	
	11.4				**SD/*rac*-Ag14a**	large
	13.7				No crystal	
	16.0				No crystal	
	18.2				No crystal	
	20.5				No crystal	
	22.8				No crystal	
11.0	11.4	1.6	40	Acetone:DCM = 4:2	No crystal	
		3.2			No crystal	
		4.8			No crystal	
		6.4			No crystal	
		7.8			**SD/*rac*-Ag14a**	large
		9.6			No crystal	
		11.2			No crystal	
		12.8			No crystal	
		14.			No crystal	
		16			No crystal	
11.0	11.4	7.8	40	Acetone	**SD/*rac*-Ag14a**	large
				Acetone:DCM = 5:1	**SD/*rac*-Ag14a**	large
				Acetone:DCM = 4:2	**SD/*rac*-Ag14a**	large
				Acetone:DCM = 3:3	No crystal	
				Acetone:DCM = 2:4	No crystal	
				Acetone:DCM = 1:5	No crystal	
				DCM	No crystal	
11.0	11.4	7.8	0	Acetone:DCM = 4:2	No crystal	
			10		No crystal	
			20		**SD/*rac*-Ag14a**	small
			30		**SD/*rac*-Ag14a**	medium
			40		**SD/*rac*-Ag14a**	large
			50		**SD/*rac*-Ag14a**	medium
			60		**SD/*rac*-Ag14a**	small
			70		No crystal	
			80		No crystal	
			90		No crystal	
			100		No crystal	

Supplementary Table 6: The excited states, energies, oscillator strengths, and the most probable transitions of **SD/*L*-Ag14**, from TD-DFT calculations.

Excitation transition	Energy (nm)	Energy (ev)	Oscillator strength (a.u.)	Most probable transitions	Weight of transition	Nature of transition
1	422.60	2.9338	0.2058	HOMO→LUMO	0.937	M→L
2	413.15	3.0010	0.1288	HOMO→LUMO+1	0.857	M→L
3	406.90	3.0471	0.0599	HOMO→LUMO+2	0.621	M→L
7	398.76	3.1093	0.0512	HOMO-2→LUMO	0.299	L→L
				HOMO-2→LUMO+3	0.136	L→L
17	380.46	3.2588	0.1187	HOMO-2→LUMO+7	0.157	L→L
				HOMO-2→LUMO+2	0.136	L→L
				HOMO-2→LUMO+3	0.104	L→L
18	379.64	3.2658	0.1027	HOMO-4→LUMO	0.231	L→L
				HOMO-3→LUMO	0.221	L→L
33	362.93	3.4162	0.0965	HOMO-3→LUMO+4	0.263	L→L
34	362.17	3.4233	0.0900	HOMO-6→LUMO	0.242	L→L
				HOMO-4→LUMO+3	0.081	L→L
43	355.39	3.4886	0.0447	HOMO→LUMO+10	0.273	M→M
44	354.96	3.4929	0.0692	HOMO→LUMO+10	0.378	M→M
				HOMO-4→LUMO+7	0.087	L→L
54	348.45	3.5581	0.0428	HOMO→LUMO+11	0.063	M→M
55	347.81	3.5647	0.0578	HOMO-6→LUMO+4	0.104	L→L
				HOMO-6→LUMO+1	0.104	L→L
59	345.93	3.5841	0.0536	HOMO-8→LUMO	0.120	L→L
				HOMO-2→LUMO+9	0.106	L→L
				HOMO-6→LUMO+3	0.091	L→L
				HOMO→LUMO+12	0.135	M→M
68	339.36	3.6534	0.0196	HOMO→LUMO+12	0.096	M→M
72	337.54	3.6732	0.0678	HOMO-4→LUMO+8	0.227	L→L
				HOMO→LUMO+12	0.072	M→M
169	303.15	4.0899	0.0428	HOMO-12→LUMO+7	0.078	L→L
				HOMO-14→LUMO+4	0.072	L→L
174	301.79	4.1083	0.0358	HOMO-17→LUMO	0.104	L→L
				HOMO-15→LUMO+1	0.070	L→L

Supplementary References

1 Rigaku Oxford Diffraction. *CrysAlis^{Pro} Software system, version 1.171.40.25a,* Rigaku Corporation: Oxford, UK (2018).

2 Palatinus, L. & Chapuis, G. SUPERFLIP - a computer program for the solution of crystal structures by charge flipping in arbitrary dimensions. *J. Appl. Crystallogr.* **40**, 786-790 (2007).

3 Sheldrick, G.M. Crystal structure refinement with SHELXL. *Acta Crystallogr. Sect. C: Struct. Chem.* **71**, 3-8 (2015).

4 Dolomanov, O.V., Bourhis, L.J., Gildea, R.J., Howard, J.A.K. & Puschmann, H. OLEX2: a complete structure solution, refinement and analysis program. *J. Appl. Crystallogr.* **42**, 339-341 (2009).

5 Spek, A.L. Structure validation in chemical crystallography. *Acta Crystallogr D.* **65**, 148-155 (2009).

6 Frisch, M., Trucks, G., Schlegel, H., Scuseria, G., Robb, M. & Cheeseman, J. *et al. Gaussian 09 (Revision D.01)*, (2009).

7 Adamo, C. & Barone, V. Exchange Functionals with Improved Long-Range Behavior and Adiabatic Connection Methods without Adjustable Parameters: The *m*PW and *m*PW1PW Models. *J. Chem. Phys.* **108**, 664-675 (1998).

8 Li, Q. S., Xu, B., Xie, Y. M., King, R. B. & Schaefer, H. F. Unsaturated Trinuclear Osmium Carbonyls: Comparison with Their Iron Analogues. *Dalton Trans.* 4312-4322 (2007).

9 Lu, T. & Chen, F. W. Multiwfn: A Multifunctional Wavefunction Analyzer. *J. Comput. Chem.* **33**, 580-592 (2012).

10 Voss, N. R. & Gerstein, M. 3V: cavity, channel and cleft volume calculator and extractor. *Nucleic Acids Res.* **38**, W555-W562 (2010).
