# Supplementary material for: Revealing the chirality origin and homochirality crystallization of Ag14 nanocluster at the molecular level
Source: Nat Commun. 2021 Aug 17;12:4966. doi: 10.1038/s41467-021-25275-2 (PMC8371133; doi:10.1038/s41467-021-25275-2)
Supplement: Supplementary file 3 — Description of Additional Supplementary Files [file 41467_2021_25275_MOESM3_ESM.pdf]

Description of Additional Supplementary Files

Supplementary Data 1: Cartesian coordinates of optimized SD/L-Ag14
