# Supplementary material for: Revealing the chirality origin and homochirality crystallization of Ag14 nanocluster at the molecular level
Source: Nat Commun. 2021 Aug 17;12:4966. doi: 10.1038/s41467-021-25275-2 (PMC8371133; doi:10.1038/s41467-021-25275-2)
Supplement: Supplementary file 4 — Supplementary Data 1 [file 41467_2021_25275_MOESM4_ESM.pdf]

Cartesian coordinates of optimized SD/*L*-Ag14.

Ag	-0.61558100	0.27698700	1.88219200
Ag	-1.73816200	0.88818600	-0.70492700
Ag	0.88700300	1.91915900	0.08405900
Ag	1.79085500	-0.78558000	0.73201200
Ag	-0.82784300	-1.82773300	-0.04344500
Ag	0.66441400	-0.18033900	-1.84714500
Ag	0.27841600	-2.73806400	3.08352300
Ag	-2.16269400	-1.44026900	-3.24450700
Ag	3.81181500	0.94131100	-2.05202000
Ag	3.13921100	1.84794200	2.84683000
Ag	-1.81216600	3.29472900	2.16926100
Ag	1.99072500	-3.98402100	-1.59490300
Ag	-0.60425400	3.70698000	-2.71649800
Ag	-4.36602100	-1.22208800	1.42519400
C	-0.59473700	6.86216000	-4.60487900
C	-0.49434900	7.72204600	-5.70707100
H	-0.68687100	7.35003200	-6.70885100
C	-0.15404700	9.05832000	-5.52669600
H	-0.07726500	9.71545500	-6.38699300
C	0.09429700	9.55169300	-4.24644200
H	0.37070400	10.59204400	-4.10982100

C	-0.00789400	8.71036300	-3.14256100
H	0.19827400	9.08804000	-2.14594200
C	-0.35498100	7.37252800	-3.32392200
H	-0.42996300	6.71238200	-2.46347400
C	0.13188400	4.51761900	-6.16599200
H	-0.15418300	3.48099700	-6.37691600
H	-0.07199300	5.10083700	-7.07140300
C	1.60279200	4.58629800	-5.76506900
H	1.73151300	4.04136800	-4.82247900
H	1.87468000	5.62949700	-5.56068600
C	2.56677400	4.02816900	-6.81255900
H	2.27259400	3.00678000	-7.08861400
H	2.49670800	4.62772500	-7.72844600
C	4.01264400	4.02829100	-6.30454000
H	4.21844300	5.00423400	-5.84384600
H	4.70675600	3.93906900	-7.14821100
C	2.00348000	-0.64033800	-5.24896900
H	1.37115200	0.09976600	-4.77059800
C	3.44622300	-3.86794300	1.88407000
C	-5.12750100	0.09753900	-1.97122300
C	-4.89743800	1.09641200	-2.92968400
H	-3.88602800	1.44830500	-3.10466900

C	0.93762400	-3.80225200	6.64155200
C	2.96608400	4.36072800	-1.64403100
C	1.35673400	5.01018100	1.82130700
C	-4.76903300	3.29229000	0.07043800
H	-4.44953800	2.64122500	0.87804100
C	-2.39713800	-4.53807600	1.80571600
C	-2.21803400	-3.99617000	-5.90017400
H	-2.15366000	-4.68479000	-5.05074800
H	-2.79107800	-4.49255700	-6.69342000
C	-1.70345000	-4.41979600	5.69214000
H	-2.28979000	-4.80191500	4.84943600
H	-1.74345200	-5.16376200	6.49775100
C	5.26810600	1.58120000	5.85758700
C	3.93546400	-3.10270200	2.95428300
H	3.45338300	-2.16293900	3.20353400
C	-3.18229900	-4.39387600	-1.91268200
H	-3.51556600	-3.36547000	-1.81886700
C	-1.80965300	-4.67771500	-2.00142300
C	5.47986500	3.25631700	-4.37873900
H	5.21953400	4.13211100	-3.77343800
H	6.36971300	3.51033900	-4.96738600
C	-7.23714200	1.14343700	-3.44658900

C	0.41358700	5.82129100	1.16951200
H	-0.53586900	5.39647600	0.86415700
C	-0.82186400	-3.63551200	-6.41026900
H	-0.91256900	-2.86614300	-7.18464200
H	-0.23729300	-3.18587800	-5.59961100
C	3.53598300	-2.50412900	-5.10282300
H	4.11050600	-3.20341600	-4.50469600
C	-5.94842500	1.62202400	-3.66544300
H	-5.78098400	2.39102100	-4.40838900
C	-3.27783700	-1.49088900	-6.83648600
C	-3.81652200	3.80747300	-0.82382000
C	2.73829600	-1.54170800	-4.46269100
C	5.61263700	4.04618400	4.34287800
C	0.68083500	7.15394700	0.90427900
H	-0.05500200	7.78606300	0.42416500
C	3.01357300	-7.56489900	-1.68497400
C	4.08646100	-5.07499300	1.56288000
H	3.71110600	-5.67807400	0.74340700
C	5.86421300	5.20020400	5.09510900
H	5.33350200	5.37776800	6.02412400
C	6.31608100	3.85480800	3.14585200
H	6.11678500	2.97609600	2.53809500

C	6.86182400	0.72546700	-4.23855300
C	-6.44165100	-0.34270500	-1.74747900
H	-6.62792100	-1.11986200	-1.01384600
C	-6.11097100	3.60158000	-0.07303200
H	-6.84934400	3.21212900	0.61526000
C	3.60181100	-2.56860300	-6.48598400
H	4.21725900	-3.30405100	-6.98852800
C	-3.16138300	0.93413100	4.39385900
C	3.88177800	-8.61777100	-1.99596400
H	4.57705800	-8.52160400	-2.82404200
C	-1.40755300	-6.02144700	-2.07759400
H	-0.34972900	-6.25414500	-2.11026600
C	5.16925700	-5.52342900	2.30440400
H	5.66261800	-6.45949600	2.07419900
C	-6.50990500	4.42847700	-1.11843500
C	-7.93736700	0.51189700	2.03247300
H	-7.56551700	0.40315100	1.01722600
C	4.63466600	-5.85949200	-3.33753500
C	1.89016100	-6.60651200	-4.13374300
H	2.42238100	-7.38135800	-4.69898100
H	1.03442800	-7.11084200	-3.67053400
C	-7.59380500	-0.43566200	3.00764200

C	1.39953100	-5.47210300	-5.03151100
H	0.98148100	-4.67906100	-4.40042700
H	2.24447300	-5.02124100	-5.56395400
C	-2.90611000	6.80611100	2.86373500
C	5.61842400	-4.75648100	3.37490000
C	-2.24310700	0.77058400	6.63042300
H	-1.41576300	0.58840200	7.30475400
C	-5.59063600	4.93962900	-2.02870100
H	-5.93365100	5.56709000	-2.84147800
C	0.58631600	-6.00841300	4.84612300
C	2.86862800	6.90830800	1.92188300
H	3.81977900	7.34719900	2.19616900
C	2.58904500	5.57567700	2.18630400
H	3.33966100	4.95964800	2.66916700
C	-2.08038600	0.69094800	5.25519400
H	-1.10681000	0.44683200	4.84241800
C	2.86184500	-1.66378300	-7.23998300
C	1.15043400	-4.72011700	7.67749200
H	0.84015600	-5.75373700	7.55775600
C	5.36828200	-0.45785400	0.91519200
C	6.21886800	-0.34780900	2.02524700
H	5.99080000	0.36932100	2.80661900

C	5.01763000	-3.54401300	3.70025600
H	5.40324000	-2.96400100	4.52876900
C	7.62064700	-2.07471700	1.13413400
C	6.78123400	-2.21979100	0.03434500
H	7.01817100	-2.94889700	-0.72871800
C	7.34602300	-1.15021500	2.13730200
H	8.01048500	-1.07336300	2.98896100
C	3.25698000	3.68693200	5.99350900
H	3.83136600	4.01825700	6.86687700
H	2.92714200	4.58100100	5.45274100
C	7.09988600	0.93358700	7.29958200
H	8.03109200	1.18065300	7.79930700
C	1.90922800	7.68643900	1.28085200
C	2.05399000	2.85786000	6.44361500
H	2.41083300	1.97692600	6.98995000
H	1.52081900	2.47674700	5.56538400
C	1.10022200	3.64245700	7.35364200
H	0.34709700	2.94989500	7.74982200
H	1.66214000	4.00653200	8.22350900
C	0.38472400	4.82639400	6.69562200
H	1.11805200	5.51379400	6.25389800
H	-0.12460600	5.40355700	7.47830100

C	2.06417200	-0.69831100	-6.63307600
H	1.49605000	-0.01323900	-7.24959400
C	-0.63654100	4.42310900	5.63122000
H	-0.17180100	3.77316000	4.88259900
H	-1.43058900	3.83085800	6.10162500
C	6.46131100	1.89249300	6.51842900
H	6.89930400	2.87995900	6.41214000
C	7.25288500	4.78913600	2.71643800
H	7.78650900	4.62726000	1.78582100
C	-1.22310500	5.63923900	4.91073500
H	-0.44114800	6.13377400	4.32329700
H	-1.59483800	6.38216100	5.62672200
C	5.00424600	-6.11276400	-4.66191800
H	4.26645500	-6.43595700	-5.38846100
C	-7.49707400	0.17323400	-2.48339900
H	-8.51156400	-0.17454700	-2.33442300
C	-4.80394300	-3.58316300	5.90296500
H	-4.58717900	-4.53548900	5.40072300
H	-5.69512300	-3.76496500	6.51766500
C	-9.08006500	-2.88539300	1.70357100
H	-9.51528400	-2.22952600	2.45093600
C	5.61693800	-5.44129500	-2.42771200

H	5.34413800	-5.23003200	-1.39725700
C	-2.33915800	-7.04808200	-2.10934700
H	-2.03630400	-8.08588300	-2.17371800
C	-2.72561300	-7.30464200	1.83154600
C	-3.64821800	-3.19866300	6.83034900
H	-3.59170200	-3.92937500	7.64741300
H	-3.88376600	-2.23654400	7.30180400
C	-4.24715500	4.62576500	-1.87819500
H	-3.52321400	5.01193700	-2.58740900
C	0.35247400	-5.93123200	-6.04588900
H	0.75683300	-6.76889200	-6.62895100
H	-0.51985500	-6.33038000	-5.51172100
C	-3.69357300	-6.73026700	-2.06015600
C	6.33010400	-5.95589600	-5.06572900
H	6.60317900	-6.15702200	-6.09683600
C	-3.67049400	-5.11083600	1.96212300
H	-4.52943400	-4.46083100	2.07796100
C	7.29941800	-5.55793700	-4.15025600
H	8.33231900	-5.44694200	-4.46353900
C	6.42179000	-0.58909000	-4.40026000
H	5.50655700	-0.91054500	-3.91283300
C	-0.07430900	-4.82870000	-7.01919700

H	-0.70162500	-5.27112700	-7.80363800
H	0.82046900	-4.44873200	-7.52800200
C	-1.29684100	-5.39116900	1.62285000
H	-0.30742100	-4.97000900	1.47600300
C	5.65928500	-1.41574500	-0.07021400
H	4.99777300	-1.52507300	-0.92349600
C	-1.45729600	-6.76661400	1.64255100
H	-0.61038500	-7.42986300	1.53201400
C	-7.69976700	-2.86670100	1.47428800
C	2.75074000	5.61343800	-2.24139100
H	1.88244800	5.76203000	-2.87201900
C	2.13613700	-7.69914100	-0.60284800
H	1.47637600	-6.87610700	-0.33956600
C	-5.12760700	-2.52253900	4.85027400
H	-4.25330600	-2.35792400	4.20939200
H	-5.32731100	-1.56391500	5.34361500
C	4.30323200	2.91293600	-5.29148300
H	3.42482700	2.73317300	-4.66157100
H	4.49498500	1.97560300	-5.82735800
C	1.93749700	-6.23782700	4.55214900
H	2.63029800	-5.40323800	4.49683100
C	2.97514500	-9.91774900	-0.17557800

H	2.95992300	-10.83235200	0.40810900
C	3.86308700	-9.78593700	-1.24116500
H	4.54216900	-10.59600800	-1.48689200
C	-6.31186900	-2.92217300	3.97094500
H	-7.24659800	-2.99101500	4.54039200
H	-6.14339000	-3.92341400	3.55775500
C	-2.26360700	-3.08313300	6.18136800
H	-2.28587900	-2.37054300	5.34957700
H	-1.58100100	-2.66118500	6.92735300
C	-7.15835300	-3.70961100	0.49619900
H	-6.09032000	-3.68449000	0.29481000
C	4.97683000	5.23714900	-0.60845900
H	5.84379500	5.11481300	0.02637800
C	4.08210000	4.19778600	-0.80756500
H	4.25244400	3.24518000	-0.31686900
C	-2.64495300	4.99969100	-5.52380200
C	-3.49606800	6.10661300	-5.60366500
H	-3.15643000	7.07971600	-5.26452000
C	-4.08309400	-1.85933200	-7.92138200
H	-4.71432000	-2.74002200	-7.85293800
C	-3.10795700	3.75232700	-5.96524100
H	-2.47839800	2.87162800	-5.87514600

C	-3.84191500	-6.48641900	1.97790600
H	-4.81951100	-6.93429500	2.10665200
C	-2.48355400	-0.34383700	-6.93219000
H	-1.86773800	-0.04329000	-6.08880900
C	-3.28782200	0.04174200	-9.17596500
H	-3.29091000	0.63546700	-10.08438200
C	-2.48598000	0.41490300	-8.10153700
H	-1.86192900	1.30029400	-8.17173200
C	4.72646300	0.29579400	5.98967900
H	3.81184200	0.03781100	5.46111200
C	8.04909600	1.13104300	-4.86206600
H	8.41923600	2.14253500	-4.72226100
C	4.75716800	6.45507200	-1.24317900
C	8.77259700	0.23827900	-5.64261800
H	9.69188500	0.56117600	-6.12068600
C	6.55053500	-0.33876200	7.43633900
H	7.05362700	-1.08691500	8.03999900
C	7.15081600	-1.48526700	-5.18037700
H	6.80170500	-2.50591600	-5.29547800
C	7.48622300	5.93917600	3.46711200
H	8.19820600	6.67958400	3.11831200
C	-4.41684400	1.23891100	4.94539300

H	-5.25696800	1.43172500	4.28864600
C	2.40333800	-7.52909000	4.33419400
H	3.45454300	-7.68949700	4.11851600
C	-3.38879700	8.01508100	0.82017000
H	-3.53840600	8.01224400	-0.25486700
C	-3.08860900	6.82403800	1.47829100
H	-2.99777600	5.90217600	0.91154000
C	6.94159800	-5.30383600	-2.82623500
H	7.69353400	-5.00044700	-2.10422400
C	1.52282700	-8.60890000	4.38629200
H	1.88560900	-9.61682700	4.21199500
C	1.36092500	-2.47899900	6.80141100
H	1.21568600	-1.76442800	5.99579800
C	-3.03665500	8.00039100	3.58267900
H	-2.92649200	8.00404200	4.66256700
C	2.17944700	-2.99460300	9.01325900
H	2.66105500	-2.68174100	9.93417900
C	2.11070300	-8.87473600	0.14393400
H	1.42282500	-8.97372600	0.97718800
C	1.76979800	-4.31734500	8.85548200
H	1.93446200	-5.03690400	9.65111800
C	-3.49876500	9.19989000	1.54085600

H	-3.72590700	10.12950400	1.02924200
C	-0.28893600	-7.09773400	4.89597100
H	-1.34133900	-6.95008700	5.11005800
C	-4.58682900	1.32171300	6.31808900
H	-5.54446200	1.57904100	6.75063400
C	6.79206300	6.14183700	4.65605000
H	6.96752800	7.03724300	5.24356400
C	1.97397800	-2.07731900	7.98708300
H	2.29716900	-1.04779000	8.10366600
C	-3.49391500	1.09289400	7.14936900
C	-4.12058300	-5.41128600	-1.95024700
H	-5.17900100	-5.19207600	-1.91729400
C	-9.23754500	1.74047300	3.64864000
H	-9.88492700	2.57617300	3.89451800
C	-9.34928300	-4.58967800	0.01484500
H	-9.98993200	-5.26078000	-0.54783900
C	-9.89906900	-3.73927800	0.97154100
H	-10.96909700	-3.74386000	1.15287700
C	-8.06817900	-0.26637700	4.31154900
H	-7.81108800	-0.97756800	5.08901200
C	-8.76331300	1.58576500	2.34596700
H	-9.03542600	2.29988000	1.57457800

C	-8.88190900	0.82028200	4.62948900
H	-9.24451700	0.93808800	5.64570400
C	-7.97747900	-4.57731300	-0.22216400
H	-7.54654800	-5.24295200	-0.96317000
C	0.17693200	-8.39049700	4.66113700
H	-0.51662500	-9.22432800	4.68739600
C	5.36032800	-0.65494600	6.78580000
H	4.92815500	-1.64491100	6.89075900
C	8.32342000	-1.07212400	-5.80264600
H	8.89238200	-1.76928200	-6.40949600
C	9.25274700	3.51709000	-0.45863100
H	10.05522600	3.87091800	0.18107600
C	8.73170900	2.23545600	-0.28334400
H	9.12135700	1.58747400	0.49507700
C	7.70649100	1.78094300	-1.10523100
H	7.30223000	0.78438300	-0.95491900
C	7.19916000	2.59451200	-2.12668700
C	7.72360700	3.88031800	-2.29071400
H	7.34434600	4.53679500	-3.06559900
C	8.74086700	4.34080200	-1.45657200
H	9.12750300	5.34586900	-1.58858200
C	-4.08874600	-1.09494900	-9.08297000

H	-4.71893000	-1.38656800	-9.91705200
C	-4.06212400	4.96355400	4.69972400
C	-4.09703400	5.07688200	6.09205200
H	-3.19613000	5.30322500	6.65148900
C	-3.32284200	9.19039800	2.92346000
H	-3.41551500	10.11152200	3.48977200
C	-5.24720000	4.66495000	4.01527400
H	-5.23571900	4.56686600	2.93325200
C	3.64113600	6.65902800	-2.04941600
H	3.48627900	7.62328500	-2.51737800
C	-5.29244700	4.89824100	6.78591200
H	-5.30201700	4.97758400	7.86816900
C	-5.23008900	4.73038100	-6.57346100
H	-6.23461800	4.62154200	-6.96773100
C	-4.38482100	3.62399300	-6.50134500
H	-4.72235800	2.65487800	-6.85479500
C	-6.46641100	4.61624200	6.09561800
H	-7.39718700	4.48309800	6.63767000
C	-4.78313800	5.96799600	-6.12083300
H	-5.43590700	6.83363500	-6.17192100
C	-6.44170400	4.50258700	4.70651700
H	-7.35167400	4.27732500	4.16030300

C	-6.51542300	-4.93677400	-4.93439200
H	-6.73318800	-5.99876400	-4.88716400
C	-5.19867300	-4.51216400	-5.10509000
H	-4.41585100	-5.25615200	-5.19802600
C	-4.89250500	-3.14834800	-5.14732400
C	-5.93154200	-2.21749400	-5.01185300
H	-5.71296500	-1.15389300	-5.02963500
C	-7.24631900	-2.64237100	-4.85988600
H	-8.03963400	-1.90755000	-4.76864400
C	-7.54076900	-4.00425500	-4.81753800
H	-8.56644400	-4.33541900	-4.69115100
Cl	1.29415400	-0.21951000	3.71954100
Cl	-0.93277100	0.99121000	-3.68709700
N	8.80821000	-2.90808000	1.23050700
N	2.91875100	-1.73353900	-8.69072800
N	2.19703000	9.07969900	0.98804400
N	6.72817300	-5.23950000	4.18231000
N	-7.91748100	4.76446500	-1.25716200
N	-2.88223200	-8.74726900	1.89548800
N	-8.32960600	1.64338800	-4.26527100
N	5.71931600	7.53087800	-1.06871800
N	-3.65842700	1.21109700	8.58798600

N	-4.68213600	-7.79136800	-2.13729900
O	8.99165800	-3.74209800	0.35203800
O	2.31049000	-0.88415700	-9.32655700
O	1.37221500	9.70818600	0.33353300
O	3.24377600	9.54717000	1.40967300
O	7.20482600	-6.32912000	3.89803300
O	9.55675300	-2.72506900	2.17820400
O	7.11020400	-4.53225800	5.10401600
O	-9.42910800	1.12519600	-4.12284000
O	-8.08342000	2.53970300	-5.06038800
O	-8.23575700	5.52783100	-2.15559800
O	3.56991900	-2.64085600	-9.19145100
O	5.51646300	8.57863400	-1.66405100
O	6.68504200	7.31986700	-0.34507600
O	-2.67300600	1.03371900	9.29181900
O	-8.70441700	4.26632500	-0.46034600
O	-4.27910200	-8.94029100	-2.23783300
O	-1.87525800	-9.43557500	1.77935700
O	-5.86568900	-7.47240700	-2.10811500
O	-4.77199900	1.48748800	9.01467000
O	-4.00702300	-9.19108800	2.07365300
P	-0.97143000	5.08233100	-4.78627100

P	4.36912400	2.79029300	4.81771500
P	2.92731000	-6.04448000	-2.70484300
P	5.89247800	1.88978900	-3.20380700
P	0.05003100	-4.27696800	5.10886300
P	-3.18033900	-2.51969500	-5.32198400
P	-6.55550000	-1.84713700	2.48091500
P	-2.54621700	5.20996700	3.69510800
S	-2.09662300	3.47755300	-0.55615500
S	-0.61875700	-3.37445400	-2.11431200
S	-2.18982700	-2.78726300	1.94762800
S	-3.78564600	-0.67317600	-1.11240700
S	0.96162700	3.33766000	2.24825800
S	2.72142400	-1.47609100	-2.69294300
S	3.99030700	0.62788700	0.68323500
S	-2.98627600	0.90368400	2.63207600
S	1.91478600	2.99010100	-2.03880500
S	1.98908700	-3.37656200	1.00627500

ECD Spectrum of optimized SD/*L*-Ag14.

X-Axis: Wavelength (nm)

Y-Axis: Δε (10-40 esu2 cm2)

Y-Axis2: Rvel

Peak information

#	X	Y	Y2
#	422.6000000000	-2.7748000000	-35.1102348932
#	413.1500000000	-5.3685000000	-69.4827089662
#	406.9000000000	-34.4295000000	-452.4541414023
#	404.4500000000	38.1664000000	504.6007592973
#	402.9400000000	46.6004000000	618.4161237802
#	400.9900000000	14.2922000000	190.5886722857
#	398.7600000000	191.0778000000	2562.3012406585
#	397.3700000000	19.3266000000	260.0709976942
#	395.8600000000	8.3054000000	112.1890511830
#	392.2100000000	36.5241000000	497.9576429361
#	390.8400000000	-12.8788000000	-176.2008244548
#	387.8400000000	40.2685000000	555.1935297272
#	385.3500000000	-1.8463000000	-25.6199600519
#	384.0500000000	9.7670000000	135.9893694727
#	383.1900000000	3.2155000000	44.8710145177

#	382.8300000000	8.3489000000	116.6151130793
#	380.4600000000	-130.9381000000	-1840.2997749907
#	379.6400000000	38.0288000000	535.6390098891
#	377.5000000000	14.2790000000	202.2611068312
#	375.4500000000	-13.0424000000	-185.7534756970
#	373.9500000000	-26.0317000000	-372.2378532895
#	373.2100000000	10.9604000000	157.0379704841
#	371.3600000000	26.5589000000	382.4251999972
#	370.0700000000	-22.7243000000	-328.3508733980
#	369.3100000000	-36.2075000000	-524.2506936783
#	368.8900000000	-47.8706000000	-693.9106138139
#	368.1300000000	-26.0360000000	-378.1852564810
#	367.5400000000	-71.0923000000	-1034.3070758158
#	367.2400000000	-141.3601000000	-2058.2987337403
#	366.4000000000	16.3737000000	238.9587324084
#	364.5800000000	44.2012000000	648.2951330805
#	363.1500000000	-6.3482000000	-93.4751335064
#	362.9300000000	-70.0366000000	-1031.8907699467
#	362.1700000000	-181.8845000000	-2685.4357073752
#	361.9700000000	17.3286000000	255.9896702944
#	361.0700000000	0.9043000000	13.3922228271
#	360.4800000000	28.0540000000	416.1454591791

#	359.1100000000	0.4439000000	6.6098132290
#	358.0900000000	-9.3971000000	-140.3244010626
#	357.9500000000	2.2136000000	33.0680298696
#	357.0900000000	-10.8797000000	-162.9186391200
#	356.4700000000	-0.0029000000	-0.0435017325
#	355.3900000000	-21.1780000000	-318.6480626942
#	354.9600000000	69.6230000000	1048.8294150773
#	354.0200000000	-1.4719000000	-22.2321799706
#	353.6700000000	-4.0288000000	-60.9128651560
#	352.8200000000	31.8005000000	481.9614415581
#	352.3400000000	-18.7418000000	-284.4335953165
#	351.7500000000	33.4350000000	508.2750166916
#	350.7600000000	-17.9555000000	-273.7278484066
#	350.1400000000	-36.5229000000	-557.7698932439
#	349.3800000000	1.7724000000	27.1265924585
#	348.9200000000	11.2033000000	171.6926262116
#	348.4500000000	24.2760000000	372.5359611305
#	347.8100000000	-100.3089000000	-1542.1582636766
#	347.6600000000	-2.6024000000	-40.0267995492
#	347.2500000000	13.2560000000	204.1276251088
#	347.0900000000	-38.9057000000	-599.3805889116
#	345.9300000000	-10.4030000000	-160.8058757251

#	345.4200000000	4.9761000000	77.0323525657
#	344.3700000000	-1.0035000000	-15.5820146411
#	343.9400000000	5.5035000000	85.5633590428
#	343.5800000000	7.9898000000	124.3482205327
#	341.6300000000	-0.6393000000	-10.0064549699
#	340.6500000000	27.2146000000	427.1939603549
#	340.3100000000	25.7808000000	405.0915840279
#	339.7100000000	-30.4522000000	-479.3380270213
#	339.3600000000	54.6034000000	860.3805351182
#	338.9300000000	-44.1583000000	-696.6808545642
#	338.3000000000	-27.0747000000	-427.9501640750
#	337.7700000000	-11.1426000000	-176.3993613287
#	337.5400000000	39.2709000000	622.1242787193
#	336.8700000000	-45.0882000000	-715.7017896086
#	336.0500000000	-36.7346000000	-584.5248206796
#	335.3900000000	12.6336000000	201.4227655915
#	334.8700000000	3.6983000000	59.0551038042
#	334.6700000000	-24.9594000000	-398.7942605070
#	333.9600000000	-0.9759000000	-15.6258052588
#	333.7100000000	6.2121000000	99.5407157226
#	332.8200000000	5.0253000000	80.7391399584
#	332.6400000000	-5.4810000000	-88.1083101628

#	332.2800000000	17.5944000000	283.1403739366
#	332.1400000000	-1.3748000000	-22.1334892504
#	331.1200000000	-10.7668000000	-173.8732509513
#	330.7200000000	-18.4538000000	-298.3711938826
#	330.0900000000	8.0690000000	130.7130323160
#	329.5700000000	-1.9753000000	-32.0491808358
#	329.1200000000	-12.2409000000	-198.8797739023
#	328.3900000000	-6.5704000000	-106.9875957335
#	327.7700000000	4.0158000000	65.5140452183
#	327.1300000000	-0.7396000000	-12.0894925149
#	326.8300000000	28.7722000000	470.7417292901
#	326.4400000000	-6.4568000000	-105.7658604659
#	326.0400000000	-5.5963000000	-91.7828723839
#	325.3700000000	1.5654000000	25.7264205062
#	324.8800000000	16.6833000000	274.5936720246
#	324.6600000000	-11.1731000000	-184.0248376650
#	324.3900000000	7.6810000000	126.6140349829
#	324.0600000000	-7.0691000000	-116.6461044309
#	323.9000000000	2.4448000000	40.3611870594
#	323.4600000000	-9.6010000000	-158.7184557422
#	323.1500000000	-10.0396000000	-166.1283652463
#	322.5900000000	15.7312000000	260.7609135485

#	321.9300000000	9.5356000000	158.3864836973
#	321.7500000000	-6.4157000000	-106.6245065510
#	321.2800000000	5.6036000000	93.2642017295
#	321.2000000000	11.9610000000	199.1239485796
#	320.6800000000	-8.6614000000	-144.4267912425
#	320.1800000000	8.8958000000	148.5669993561
#	319.9200000000	-1.3027000000	-21.7738167670
#	319.6300000000	17.2578000000	288.7150603472
#	319.4300000000	-10.9350000000	-183.0520630033
#	318.6900000000	-15.0377000000	-252.3158437282
#	318.3700000000	1.1272000000	18.9321695208
#	318.2200000000	14.9476000000	251.1744951445
#	317.9300000000	15.5124000000	260.9029722442
#	317.6200000000	0.9499000000	15.9919560709
#	317.1800000000	-18.4801000000	-311.5516560211
#	317.0300000000	15.8813000000	267.8657769679
#	316.7100000000	4.1886000000	70.7194141043
#	316.5400000000	31.7197000000	535.8361564517
#	316.0500000000	14.6504000000	247.8707207489
#	315.9700000000	5.5478000000	93.8872217565
#	315.9000000000	20.9909000000	355.3145365646
#	315.7300000000	0.9276000000	15.7100089418

#	315.7000000000	2.9881000000	50.6118354627
#	315.2300000000	8.3478000000	141.6041675947
#	314.9900000000	2.9263000000	49.6768011827
#	314.8400000000	27.2677000000	463.1163837446
#	314.7000000000	30.5724000000	519.4746176275
#	314.5500000000	2.5144000000	42.7441042463
#	314.2000000000	6.9030000000	117.4798097516
#	314.0300000000	9.8309000000	167.3993167826
#	313.7200000000	-3.3688000000	-57.4201818980
#	313.4300000000	3.3877000000	57.7957525924
#	313.0700000000	28.2962000000	483.3015690235
#	312.9400000000	47.0108000000	803.2821295949
#	312.7500000000	10.4469000000	178.6165353592
#	312.4900000000	9.2421000000	158.1488656195
#	312.2700000000	28.8958000000	494.8071895666
#	312.0900000000	3.6627000000	62.7556796100
#	311.7500000000	21.6481000000	371.3170274872
#	311.3900000000	-12.4386000000	-213.5985904307
#	311.0400000000	1.0801000000	18.5686044394
#	310.7000000000	-39.8860000000	-686.4529465291
#	310.3600000000	4.7438000000	81.7320081108
#	309.7600000000	-21.6021000000	-372.9084104561

#	309.5800000000	-5.2975000000	-91.5017944929
#	308.8200000000	-8.5464000000	-147.9821519819
#	308.4400000000	-0.0378000000	-0.6553188301
#	308.4000000000	-17.5732000000	-304.6968919484
#	308.0900000000	5.0380000000	87.4403933934
#	307.9900000000	3.7788000000	65.6067965821
#	307.7500000000	-5.9596000000	-103.5501164776
#	307.2500000000	30.3760000000	528.6524319665
#	307.0700000000	-8.1475000000	-141.8791317279
#	306.9500000000	8.2728000000	144.1174035796
#	306.6700000000	1.7656000000	30.7859506473
#	306.4700000000	-5.1164000000	-89.2705198445
#	306.0600000000	18.3728000000	320.9965135626
#	305.7100000000	2.8288000000	49.4793675895
#	305.5100000000	6.4102000000	112.1960820807
#	305.2800000000	-10.5397000000	-184.6126422839
#	305.0400000000	4.6368000000	81.2817627209
#	304.7000000000	11.4251000000	200.5021793982
#	304.3800000000	-29.0855000000	-510.9659533142
#	303.9600000000	41.0204000000	721.6306934802
#	303.5500000000	15.5578000000	274.0624203725
#	303.1500000000	-4.8348000000	-85.2810389799

#	302.9800000000	7.5269000000	132.8414865196
#	302.6200000000	-0.3928000000	-6.9407338950
#	302.4800000000	19.5994000000	346.4795877072
#	302.4200000000	0.7179000000	12.6936051853
#	301.7900000000	-56.5924000000	-1002.7318480677
#	301.4500000000	10.8165000000	191.8682030990
#	301.2700000000	-12.8210000000	-227.5608538213
#	300.9500000000	0.6483000000	11.5189585579
#	300.5600000000	17.5043000000	311.4190013935
#	300.1900000000	-9.0625000000	-161.4296631785
#	300.0100000000	7.0007000000	124.7777867915
#	299.9200000000	-7.0199000000	-125.1575462262
#	299.5800000000	10.3654000000	185.0140847260
#	299.1500000000	-7.9474000000	-142.0586249743
#	298.9800000000	-18.3896000000	-328.8983442328
#	298.8100000000	-20.2674000000	-362.6890577559
#	298.3300000000	2.3387000000	41.9188277689
#	298.0000000000	10.9419000000	196.3396530187
#	297.7200000000	-6.7652000000	-121.5078039361
#	297.1800000000	-19.5462000000	-351.7015657182
#	297.0200000000	-26.4752000000	-476.6340864625
#	296.5700000000	-12.0419000000	-217.1197553945

#	296.3300000000	-1.8779000000	-33.8866301610
#	296.1900000000	-5.6593000000	-102.1701113428
#	295.8900000000	3.6109000000	65.2554389403
#	295.6000000000	3.2426000000	58.6570868718
#	295.3600000000	4.8184000000	87.2333824966
#	295.0500000000	-30.8012000000	-558.2176442399
#	294.7600000000	-8.3324000000	-151.1586846970
#	294.4200000000	-9.9568000000	-180.8356356773
#	294.1800000000	-6.8568000000	-124.6349604838
#	294.0500000000	2.7784000000	50.5248611206
#	293.9500000000	2.0541000000	37.3662624577
#	293.7200000000	5.6792000000	103.3915831797
#	293.4600000000	16.8847000000	307.6635007422
#	293.0900000000	-0.9083000000	-16.5714249822
#	292.7500000000	-30.3553000000	-554.4586554344
#	292.6900000000	20.4283000000	373.2122448958
#	292.5200000000	-9.4092000000	-172.0000911334
#	292.0400000000	1.0300000000	18.8593372499
#	291.8200000000	-47.6798000000	-873.6770219887
#	291.4300000000	18.9309000000	347.3509947495
#	291.3500000000	-19.1336000000	-351.1666058080
#	291.2500000000	2.7109000000	49.7713138631

#	291.0600000000	19.7796000000	363.3846027464
#	290.9000000000	-1.0661000000	-19.5968272374
#	290.4000000000	-0.1868000000	-3.4396305827
#	290.3200000000	-2.7117000000	-49.9454842001
#	290.1600000000	-18.8243000000	-346.9068175081
#	289.9800000000	9.3560000000	172.5256647658
#	289.7100000000	-8.1991000000	-151.3332082030
#	289.4000000000	23.2805000000	430.1553399366
#	289.2700000000	-5.3909000000	-99.6527807921
#	289.0600000000	-2.2884000000	-42.3326526027
#	288.8400000000	-20.4342000000	-378.2960836797
#	288.5900000000	-4.1120000000	-76.1909466652
#	288.5400000000	17.5998000000	326.1619140211
#	288.3800000000	6.6261000000	122.8639264508
#	288.0400000000	2.4978000000	46.3699257933
#	287.8400000000	3.3629000000	62.4732859004
#	287.8100000000	8.0837000000	150.1882130667
#	287.5300000000	-21.8375000000	-406.1171179496
#	287.3100000000	22.0485000000	410.3551126273
#	287.2100000000	2.3386000000	43.5399504266
#	287.1000000000	-36.1281000000	-672.8890665384
#	286.8700000000	-2.5871000000	-48.2235995676

#	286.6700000000	6.4671000000	120.6309844037
#	286.5000000000	-15.6742000000	-292.5447438492
#	286.2000000000	0.1981000000	3.7012326789
#	286.0000000000	5.4475000000	101.8504020397
#	285.9100000000	4.9061000000	91.7568703995
#	285.7800000000	13.3715000000	250.1956984082
#	285.6000000000	11.3677000000	212.8364186577
#	285.4400000000	-9.0967000000	-170.4121601136
#	285.3000000000	-33.6895000000	-631.4287260603
#	285.1200000000	0.0216000000	0.4050956789
#	285.1100000000	-1.7630000000	-33.0652190429
#	284.9100000000	9.6412000000	180.9484808025
#	284.7200000000	7.9244000000	148.8263929929
#	284.3400000000	-27.5960000000	-518.9669715427
#	284.2700000000	-40.9876000000	-770.9975564255
#	284.0600000000	-13.5806000000	-255.6468329803
#	283.9500000000	8.6677000000	163.2275794059
#	283.7700000000	-11.1312000000	-209.7524526138
#	283.6300000000	2.0971000000	39.5365270133
#	283.5100000000	4.5581000000	85.9700162438
#	283.3000000000	-3.7206000000	-70.2260027354
#	283.0800000000	-6.9977000000	-132.1836301626

#	283.0200000000	0.6360000000	12.0163212620
#	282.6900000000	23.7941000000	450.0806878019
#	282.6700000000	-11.5807000000	-219.0718795042
#	282.5300000000	7.1591000000	135.4956651273
#	282.2900000000	-5.4559000000	-103.3480888173
#	282.2200000000	-21.8547000000	-414.0841465771
#	282.1300000000	-5.9098000000	-112.0095510952
#	282.0600000000	-24.6032000000	-466.4248035771
#	281.8300000000	-17.8225000000	-338.1527663244
#	281.7000000000	1.2707000000	24.1205787906
#	281.5100000000	-3.2347000000	-61.4429025680
#	281.4400000000	10.6137000000	201.6566404761
#	281.3100000000	4.1235000000	78.3812833606
#	281.1300000000	-4.6577000000	-88.5922765136
#	281.0000000000	18.7268000000	356.3598719907
#	280.9600000000	14.3860000000	273.7960028675
#	280.8500000000	-2.0411000000	-38.8616643437
#	280.6900000000	10.0457000000	191.3748246585
#	280.4600000000	-1.0899000000	-20.7800823713
#	280.3400000000	11.5119000000	219.5803541689
#	280.1800000000	2.9446000000	56.1979817144
#	280.0400000000	12.3095000000	235.0454700253

#	279.9300000000	17.2854000000	330.1881870344
#	279.9000000000	29.3320000000	560.3641200064
#	279.7400000000	-33.0619000000	-631.9821023461
#	279.6400000000	6.8862000000	131.6775933565
#	279.4600000000	-7.2963000000	-139.6093707391
#	279.3900000000	17.6099000000	337.0369949804
#	279.2700000000	13.7448000000	263.1756363185
#	279.1800000000	4.1821000000	80.1016850481
#	279.0800000000	26.9896000000	517.1294554556
#	279.0300000000	-16.9745000000	-325.2951838217
#	278.9600000000	21.9128000000	420.0369365682
#	278.8700000000	22.2037000000	425.7504307405
#	278.7400000000	-16.0592000000	-308.0747842183
#	278.6700000000	-36.8386000000	-706.8779608045
#	278.6000000000	46.9008000000	900.1827376513
#	278.5300000000	43.7741000000	840.3820902869
#	278.5200000000	-27.0273000000	-518.8930067578
#	278.4300000000	2.5060000000	48.1278633114
#	278.3900000000	-4.8667000000	-93.4786617883
#	278.2200000000	-2.9536000000	-56.7668603386
#	278.1800000000	2.3512000000	45.1955017446

Spectra

#	X	Y	DY/DX
	200.0000000000	0.0000000000	0.0000000000
	200.6000000000	0.0000000000	0.0000000000
	201.2000000000	0.0000000000	0.0000000000
	201.8000000000	0.0000000000	0.0000000000
	202.4000000000	0.0000000000	0.0000000000
	203.0000000000	0.0000000000	0.0000000000
	203.6000000000	0.0000000000	0.0000000000
	204.2000000000	0.0000000000	0.0000000000
	204.8000000000	0.0000000000	0.0000000000
	205.4000000000	0.0000000000	0.0000000000
	206.0000000000	0.0000000000	0.0000000000
	206.6000000000	0.0000000001	0.0000000000
	207.2000000000	0.0000000002	0.0000000000
	207.8000000000	0.0000000003	0.0000000000
	208.4000000000	0.0000000005	0.0000000000
	209.0000000000	0.0000000009	0.0000000000
	209.6000000000	0.0000000016	0.0000000000
	210.2000000000	0.0000000027	0.0000000000
	210.8000000000	0.0000000047	0.0000000000
	211.4000000000	0.0000000079	0.0000000000

212.0000000000	0.0000000133	0.0000000000
212.6000000000	0.0000000222	0.0000000000
213.2000000000	0.0000000367	0.0000000000
213.8000000000	0.0000000601	0.0000000000
214.4000000000	0.0000000975	0.0000000000
215.0000000000	0.0000001568	0.0000000000
215.6000000000	0.0000002501	0.0000000000
216.2000000000	0.0000003956	0.0000000000
216.8000000000	0.0000006207	0.0000000000
217.4000000000	0.0000009659	0.0000000000
218.0000000000	0.0000014912	0.0000000000
218.6000000000	0.0000022841	0.0000000000
219.2000000000	0.0000034716	0.0000000000
219.8000000000	0.0000052361	0.0000000000
220.4000000000	0.0000078378	0.0000000000
221.0000000000	0.0000116451	0.0000000000
221.6000000000	0.0000171748	0.0000000000
222.2000000000	0.0000251469	0.0000000000
222.8000000000	0.0000365562	0.0000000000
223.4000000000	0.0000527674	0.0000000000
224.0000000000	0.0000756375	0.0000000001
224.6000000000	0.0001076751	0.0000000001

225.2000000000	0.0001522439	0.0000000003
225.8000000000	0.0002138204	0.0000000005
226.4000000000	0.0002983193	0.0000000010
227.0000000000	0.0004134979	0.0000000020
227.6000000000	0.0005694583	0.0000000038
228.2000000000	0.0007792612	0.0000000071
228.8000000000	0.0010596737	0.0000000134
229.4000000000	0.0014320688	0.0000000247
230.0000000000	0.0019235010	0.0000000450
230.6000000000	0.0025679779	0.0000000811
231.2000000000	0.0034079513	0.0000001444
231.8000000000	0.0044960488	0.0000002539
232.4000000000	0.0058970652	0.0000004410
233.0000000000	0.0076902280	0.0000007571
233.6000000000	0.0099717507	0.0000012846
234.2000000000	0.0128576752	0.0000021544
234.8000000000	0.0164870009	0.0000035720
235.4000000000	0.0210250866	0.0000058559
236.0000000000	0.0266672970	0.0000094933
236.6000000000	0.0336428536	0.0000152212
237.2000000000	0.0422188340	0.0000241404
237.8000000000	0.0527042427	0.0000378759

238.4000000000	0.0654540613	0.0000587973
239.0000000000	0.0808731640	0.0000903197
239.6000000000	0.0994199627	0.0001373069
240.2000000000	0.1216096308	0.0002066038
240.8000000000	0.1480167300	0.0003077312
241.4000000000	0.1792770518	0.0004537765
242.0000000000	0.2160884712	0.0006625187
242.6000000000	0.2592105980	0.0009578279
243.2000000000	0.3094630077	0.0013713753
243.8000000000	0.3677218355	0.0019446865
244.4000000000	0.4349145223	0.0027315623
245.0000000000	0.5120125192	0.0038008731
245.6000000000	0.6000217772	0.0052397133
246.2000000000	0.6999708829	0.0071568743
246.8000000000	0.8128967401	0.0096865554
247.4000000000	0.9398277440	0.0129921907
248.0000000000	1.0817644554	0.0172702190
248.6000000000	1.2396578391	0.0227535699
249.2000000000	1.4143852090	0.0297145792
249.8000000000	1.6067240869	0.0384669968
250.4000000000	1.8173242685	0.0493666937
251.0000000000	2.0466784606	0.0628106425

251.6000000000	2.2950919350	0.0792337192
252.2000000000	2.5626517155	0.0991028784
252.8000000000	2.8491958834	0.1229082815
253.4000000000	3.1542836436	0.1511510214
254.0000000000	3.4771668429	0.1843271866
254.6000000000	3.8167636669	0.2229081454
255.2000000000	4.1716352622	0.2673171072
255.8000000000	4.5399660332	0.3179022252
256.4000000000	4.9195483502	0.3749067435
257.0000000000	5.3077723718	0.4384369417
257.6000000000	5.7016216329	0.5084288880
258.2000000000	6.0976749807	0.5846152562
258.8000000000	6.4921153505	0.6664936779
259.4000000000	6.8807457709	0.7532982724
260.0000000000	7.2590128670	0.8439761029
260.6000000000	7.6220379975	0.9371703352
261.2000000000	7.9646560209	1.0312118168
261.8000000000	8.2814615361	1.1241206294
262.4000000000	8.5668622886	1.2136189147
263.0000000000	8.8151392852	1.2971559103
263.6000000000	9.0205130039	1.3719456982
264.2000000000	9.1772149502	1.4350176510

264.8000000000	9.2795636735	1.4832790077
265.4000000000	9.3220442417	1.5135884299
266.0000000000	9.2993900678	1.5228388199
266.6000000000	9.2066659022	1.5080471550
267.2000000000	9.0393507377	1.4664486326
267.8000000000	8.7934193391	1.3955920644
268.4000000000	8.4654210912	1.2934332180
269.0000000000	8.0525548673	1.1584227113
269.6000000000	7.5527386550	0.9895851151
270.2000000000	6.9646727310	0.7865861265
270.8000000000	6.2878952554	0.5497850228
271.4000000000	5.5228292584	0.2802700881
272.0000000000	4.6708201077	-0.0201247087
272.6000000000	3.7341626837	-0.3488228354
273.2000000000	2.7161176379	-0.7025284525
273.8000000000	1.6209162686	-1.0772694057
274.4000000000	0.4537537203	-1.4684588487
275.0000000000	-0.7792296201	-1.8709737322
275.6000000000	-2.0709784764	-2.2792478217
276.2000000000	-3.4135645648	-2.6873764441
276.8000000000	-4.7982396129	-3.0892298177
277.4000000000	-6.2154989657	-3.4785716216

278.0000000000	-7.6551550797	-3.8491793929
278.6000000000	-9.1064200668	-4.1949634104
279.2000000000	-10.5579963304	-4.5100809226
279.8000000000	-11.9981742288	-4.7890428793
280.4000000000	-13.4149356112	-5.0268107222
281.0000000000	-14.7960620049	-5.2188812468
281.6000000000	-16.1292461788	-5.3613580484
282.2000000000	-17.4022057792	-5.4510085776
282.8000000000	-18.6027977206	-5.4853063372
283.4000000000	-19.7191320216	-5.4624582302
284.0000000000	-20.7396838007	-5.3814174975
284.6000000000	-21.6534021889	-5.2418830521
285.2000000000	-22.4498149744	-5.0442863187
285.8000000000	-23.1191278659	-4.7897669092
286.4000000000	-23.6523173460	-4.4801386146
287.0000000000	-24.0412161843	-4.1178472700
287.6000000000	-24.2785907827	-3.7059220584
288.2000000000	-24.3582096393	-3.2479217710
288.8000000000	-24.2749023369	-2.7478774454
289.4000000000	-24.0246085826	-2.2102326716
290.0000000000	-23.6044169491	-1.6397826998
290.6000000000	-23.0125930946	-1.0416133117

291.2000000000	-22.2485973581	-0.4210402476
291.8000000000	-21.3130917490	0.2164501869
292.4000000000	-20.2079364638	0.8652588653
293.0000000000	-18.9361761719	1.5197295983
293.6000000000	-17.5020164167	2.1742026815
294.2000000000	-15.9107905717	2.8230660959
294.8000000000	-14.1689178761	3.4608042857
295.4000000000	-12.2838531552	4.0820444762
296.0000000000	-10.2640288920	4.6816005113
296.6000000000	-8.1187903781	5.2545141885
297.2000000000	-5.8583247148	5.7960940512
297.8000000000	-3.4935844732	6.3019515721
298.4000000000	-1.0362068444	6.7680346269
299.0000000000	1.5015708699	7.1906581219
299.6000000000	4.1069985743	7.5665316035
300.2000000000	6.7669026561	7.8927836491
300.8000000000	9.4677763833	8.1669828161
301.4000000000	12.1958706126	8.3871549121
302.0000000000	14.9372840214	8.5517963510
302.6000000000	17.6780521152	8.6598833670
303.2000000000	20.4042343061	8.7108768844
303.8000000000	23.1019984029	8.7047228759

304.4000000000	25.7577019083	8.6418480852
305.0000000000	28.3579695746	8.5231510495
305.6000000000	30.8897667281	8.3499884181
306.2000000000	33.3404679339	8.1241566331
306.8000000000	35.6979206363	7.8478691122
307.4000000000	37.9505034734	7.5237291459
308.0000000000	40.0871790265	7.1546987973
308.6000000000	42.0975408303	6.7440641612
309.2000000000	43.9718545267	6.2953974040
309.8000000000	45.7010931093	5.8125160649
310.4000000000	47.2769662567	5.2994401469
311.0000000000	48.6919438106	4.7603475665
311.6000000000	49.9392735015	4.1995285557
312.2000000000	51.0129930734	3.6213396304
312.8000000000	51.9079369987	3.0301577395
313.4000000000	52.6197380135	2.4303352033
314.0000000000	53.1448237376	1.8261560287
314.6000000000	53.4804086698	1.2217941597
315.2000000000	53.6244818750	0.6212741787
315.8000000000	53.5757906997	0.0284349280
316.4000000000	53.3338208649	-0.5531035404
317.0000000000	52.8987732986	-1.1199693375

317.6000000000	52.2715380753	-1.6690641534
318.2000000000	51.4536658307	-2.1975845064
318.8000000000	50.4473370221	-2.7030386868
319.4000000000	49.2553293952	-3.1832593279
320.0000000000	47.8809840113	-3.6364116052
320.6000000000	46.3281701803	-4.0609971307
321.2000000000	44.6012496246	-4.4558536714
321.8000000000	42.7050401882	-4.8201508771
322.4000000000	40.6447793830	-5.1533822504
323.0000000000	38.4260880464	-5.4553536383
323.6000000000	36.0549343603	-5.7261685583
324.2000000000	33.5375984616	-5.9662107003
324.8000000000	30.8806378478	-6.1761239702
325.4000000000	28.0908537606	-6.3567904491
326.0000000000	25.1752587048	-6.5093066553
326.6000000000	22.1410452347	-6.6349584926
327.2000000000	18.9955561204	-6.7351952651
327.8000000000	15.7462559801	-6.8116031267
328.4000000000	12.4007044444	-6.8658783193
329.0000000000	8.9665308972	-6.8998005336
329.6000000000	5.4514108182	-6.9152067034
330.2000000000	1.8630437330	-6.9139655213

330.8000000000	-1.7908672389	-6.8979529333
331.4000000000	-5.5026342651	-6.8690288441
332.0000000000	-9.2646021498	-6.8290152357
332.6000000000	-13.0691642043	-6.7796758713
333.2000000000	-16.9087767010	-6.7226977287
333.8000000000	-20.7759719836	-6.6596742802
334.4000000000	-24.6633703113	-6.5920907054
335.0000000000	-28.5636905243	-6.5213111022
335.6000000000	-32.4697596183	-6.4485677311
336.2000000000	-36.3745213198	-6.3749523106
336.8000000000	-40.2710437580	-6.3014093542
337.4000000000	-44.1525263240	-6.2287315245
338.0000000000	-48.0123058123	-6.1575569587
338.6000000000	-51.8438619329	-6.0883685039
339.2000000000	-55.6408222820	-6.0214947864
339.8000000000	-59.3969668545	-5.9571130249
340.4000000000	-63.1062321745	-5.8952534849
341.0000000000	-66.7627151185	-5.8358054633
341.6000000000	-70.3606764951	-5.7785246798
342.2000000000	-73.8945444435	-5.7230419478
342.8000000000	-77.3589177011	-5.6688729860
343.4000000000	-80.7485687879	-5.6154292323

344.0000000000	-84.0584471450	-5.5620295121
344.6000000000	-87.2836822601	-5.5079124151
345.2000000000	-90.4195868023	-5.4522492295
345.8000000000	-93.4616597855	-5.3941572840
346.4000000000	-96.4055897692	-5.3327135486
347.0000000000	-99.2472581031	-5.2669683473
347.6000000000	-101.9827422108	-5.1959590386
348.2000000000	-104.6083189077	-5.1187235242
348.8000000000	-107.1204677390	-5.0343134529
349.4000000000	-109.5158743201	-4.9418069924
350.0000000000	-111.7914336591	-4.8403210482
350.6000000000	-113.9442534350	-4.7290228211
351.2000000000	-115.9716572035	-4.6071406007
351.8000000000	-117.8711875003	-4.4739737041
352.4000000000	-119.6406088077	-4.3289014810
353.0000000000	-121.2779103517	-4.1713913162
353.6000000000	-122.7813086942	-4.0010055738
354.2000000000	-124.1492500835	-3.8174074402
354.8000000000	-125.3804125300	-3.6203656336
355.4000000000	-126.4737075714	-3.4097579625
356.0000000000	-127.4282816931	-3.1855737269
356.6000000000	-128.2435173734	-2.9479149681

357.2000000000	-128.9190337226	-2.6969965851
357.8000000000	-129.4546866874	-2.4331453469
358.4000000000	-129.8505687970	-2.1567978400
359.0000000000	-130.1070084261	-1.8684974001
359.6000000000	-130.2245685574	-1.5688900874
360.2000000000	-130.2040450262	-1.2587197693
360.8000000000	-130.0464642336	-0.9388223868
361.4000000000	-129.7530803203	-0.6101194795
362.0000000000	-129.3253717938	-0.2736110560
362.6000000000	-128.7650376075	0.0696321057
363.2000000000	-128.0739926916	0.4184766366
363.8000000000	-127.2543629425	0.7717351444
364.4000000000	-126.3084796769	1.1281750788
365.0000000000	-125.2388735632	1.4865278036
365.6000000000	-124.0482680450	1.8454977883
366.2000000000	-122.7395722740	2.2037718312
366.8000000000	-121.3158735741	2.5600282329
367.4000000000	-119.7804294588	2.9129458410
368.0000000000	-118.1366592292	3.2612128914
368.6000000000	-116.3881351812	3.6035355789
369.2000000000	-114.5385734508	3.9386462932
369.8000000000	-112.5918245314	4.2653114632

370.4000000000	-110.5518634965	4.5823389609
371.0000000000	-108.4227799629	4.8885850174
371.6000000000	-106.2087678307	5.1829606169
372.2000000000	-103.9141148385	5.4644373356
372.8000000000	-101.5431919694	5.7320526008
373.4000000000	-99.1004427481	5.9849143525
374.0000000000	-96.5903724665	6.2222050956
374.6000000000	-94.0175373759	6.4431853341
375.2000000000	-91.3865338839	6.6471963890
375.8000000000	-88.7019877930	6.8336626019
376.4000000000	-85.9685436178	7.0020929341
377.0000000000	-83.1908540161	7.1520819744
377.6000000000	-80.3735693689	7.2833103729
378.2000000000	-77.5213275412	7.3955447211
378.8000000000	-74.6387438576	7.4886369033
379.4000000000	-71.7304013200	7.5625229447
380.0000000000	-68.8008410992	7.6172213865
380.6000000000	-65.8545533240	7.6528312177
381.2000000000	-62.8959681953	7.6695293977
381.8000000000	-59.9294474464	7.6675680016
382.4000000000	-56.9592761710	7.6472710237
383.0000000000	-53.9896550386	7.6090308750

383.6000000000	-51.0246929125	7.5533046085
384.2000000000	-48.0683998865	7.4806099085
384.8000000000	-45.1246807524	7.3915208790
385.4000000000	-42.1973289088	7.2866636654
386.0000000000	-39.2900207197	7.1667119433
386.6000000000	-36.4063103297	7.0323823074
387.2000000000	-33.5496249396	6.8844295925
387.8000000000	-30.7232605451	6.7236421570
388.4000000000	-27.9303781398	6.5508371582
389.0000000000	-25.1740003801	6.3668558476
389.6000000000	-22.4570087104	6.1725589125
390.2000000000	-19.7821409423	5.9688218885
390.8000000000	-17.1519892836	5.7565306662
391.4000000000	-14.5689988068	5.5365771142
392.0000000000	-12.0354663508	5.3098548368
392.6000000000	-9.5535398428	5.0772550859
393.2000000000	-7.1252180306	4.8396628429
393.8000000000	-4.7523506112	4.5979530839
394.4000000000	-2.4366387429	4.3529872437
395.0000000000	-0.1796359256	4.1056098867
395.6000000000	2.0172507661	3.8566455963
396.2000000000	4.1527591127	3.6068960887

396.8000000000	6.2257698605	3.3571375588
397.4000000000	8.2353045284	3.1081182616
398.0000000000	10.1805229312	2.8605563315
398.6000000000	12.0607204356	2.6151378427
399.2000000000	13.8753249668	2.3725151086
399.8000000000	15.6238937863	2.1333052199
400.4000000000	17.3061100573	1.8980888192
401.0000000000	18.9217792168	1.6674091061
401.6000000000	20.4708251732	1.4417710698
402.2000000000	21.9532863468	1.2216409427
402.8000000000	23.3693115723	1.0074458662
403.4000000000	24.7191558794	0.7995737629
404.0000000000	26.0031761706	0.5983734047
404.6000000000	27.2218268114	0.4041546680
405.2000000000	28.3756551510	0.2171889659
405.8000000000	29.4652969880	0.0377098455
406.4000000000	30.4914719981	-0.1340862588
407.0000000000	31.4549791365	-0.2980391303
407.6000000000	32.3566920322	-0.4540237418
408.2000000000	33.1975543853	-0.6019491102
408.8000000000	33.9785753813	-0.7417570416
409.4000000000	34.7008251353	-0.8734207834

410.0000000000	35.3654301769	-0.9969435917
410.6000000000	35.9735689872	-1.1123572286
411.2000000000	36.5264675981	-1.2197204001
411.8000000000	37.0253952635	-1.3191171458
412.4000000000	37.4716602118	-1.4106551921
413.0000000000	37.8666054869	-1.4944642800
413.6000000000	38.2116048858	-1.5706944764
414.2000000000	38.5080590003	-1.6395144812
414.8000000000	38.7573913678	-1.7011099365
415.4000000000	38.9610447372	-1.7556817498
416.0000000000	39.1204774546	-1.8034444376
416.6000000000	39.2371599745	-1.8446244969
417.2000000000	39.3125714969	-1.8794588135
417.8000000000	39.3481967372	-1.9081931110
418.4000000000	39.3455228285	-1.9310804491
419.0000000000	39.3060363596	-1.9483797734
419.6000000000	39.2312205494	-1.9603545238
420.2000000000	39.1225525600	-1.9672713043
420.8000000000	38.9815009469	-1.9693986172
421.4000000000	38.8095232477	-1.9670056655
422.0000000000	38.6080637088	-1.9603612244
422.6000000000	38.3785511488	-1.9497325847

423.2000000000	38.1223969575	-1.9353845686
423.8000000000	37.8409932293	-1.9175786185
424.4000000000	37.5357110283	-1.8965719587
425.0000000000	37.2078987844	-1.8726168308
425.6000000000	36.8588808163	-1.8459598003
426.2000000000	36.4899559797	-1.8168411351
426.8000000000	36.1023964376	-1.7854942527
427.4000000000	35.6974465493	-1.7521452365
428.0000000000	35.2763218753	-1.7170124163
428.6000000000	34.8402082941	-1.6803060139
429.2000000000	34.3902612271	-1.6422278486
429.8000000000	33.9276049693	-1.6029711017
430.4000000000	33.4533321203	-1.5627201359
431.0000000000	32.9685031118	-1.5216503680
431.6000000000	32.4741458297	-1.4799281904
432.2000000000	31.9712553237	-1.4377109396
432.8000000000	31.4607936028	-1.3951469069
433.4000000000	30.9436895116	-1.3523753908
434.0000000000	30.4208386835	-1.3095267845
434.6000000000	29.8931035667	-1.2667226982
435.2000000000	29.3613135186	-1.2240761117
435.8000000000	28.8262649663	-1.1816915547

436.4000000000	28.2887216265	-1.1396653110
437.0000000000	27.7494147831	-1.0980856454
437.6000000000	27.2090436186	-1.0570330490
438.2000000000	26.6682755935	-1.0165805000
438.8000000000	26.1277468730	-0.9767937396
439.4000000000	25.5880627947	-0.9377315580
440.0000000000	25.0497983757	-0.8994460895
440.6000000000	24.5134988553	-0.8619831145
441.2000000000	23.9796802685	-0.8253823660
441.8000000000	23.4488300498	-0.7896778390
442.4000000000	22.9214076621	-0.7548981007
443.0000000000	22.3978452482	-0.7210666004
443.6000000000	21.8785483034	-0.6882019768
444.2000000000	21.3638963639	-0.6563183622
444.8000000000	20.8542437110	-0.6254256821
445.4000000000	20.3499200872	-0.5955299484
446.0000000000	19.8512314220	-0.5666335467
446.6000000000	19.3584605654	-0.5387355151
447.2000000000	18.8718680265	-0.5118318156
447.8000000000	18.3916927162	-0.4859155955
448.4000000000	17.9181526910	-0.4609774403
449.0000000000	17.4514458960	-0.4370056156

449.6000000000	16.9917509070	-0.4139863002
450.2000000000	16.5392276685	-0.3919038069
450.8000000000	16.0940182261	-0.3707407943
451.4000000000	15.6562474538	-0.3504784663
452.0000000000	15.2260237726	-0.3310967624
452.6000000000	14.8034398608	-0.3125745355
453.2000000000	14.3885733541	-0.2948897206
453.8000000000	13.9814875352	-0.2780194916
454.4000000000	13.5822320117	-0.2619404086
455.0000000000	13.1908433805	-0.2466285547
455.6000000000	12.8073458806	-0.2320596630
456.2000000000	12.4317520302	-0.2182092337
456.8000000000	12.0640632508	-0.2050526422
457.4000000000	11.7042704752	-0.1925652381
458.0000000000	11.3523547409	-0.1807224355
458.6000000000	11.0082877673	-0.1694997951
459.2000000000	10.6720325171	-0.1588730982
459.8000000000	10.3435437409	-0.1488184136
460.4000000000	10.0227685059	-0.1393121568
461.0000000000	9.7096467079	-0.1303311426
461.6000000000	9.4041115663	-0.1218526318
462.2000000000	9.1060901023	-0.1138543710

462.8000000000	8.8155036011	-0.1063146276
463.4000000000	8.5322680562	-0.0992122188
464.0000000000	8.2562945987	-0.0925265366
464.6000000000	7.9874899085	-0.0862375669
465.2000000000	7.7257566102	-0.0803259061
465.8000000000	7.4709936525	-0.0747727721
466.4000000000	7.2230966720	-0.0695600129
467.0000000000	6.9819583407	-0.0646701114
467.6000000000	6.7474686986	-0.0600861874
468.2000000000	6.5195154706	-0.0557919969
468.8000000000	6.2979843694	-0.0517719285
469.4000000000	6.0827593828	-0.0480109988
470.0000000000	5.8737230473	-0.0444948442
470.6000000000	5.6707567080	-0.0412097124
471.2000000000	5.4737407641	-0.0381424513
471.8000000000	5.2825549024	-0.0352804979
472.4000000000	5.0970783166	-0.0326118640
473.0000000000	4.9171899155	-0.0301251235
473.6000000000	4.7427685176	-0.0278093964
474.2000000000	4.5736930355	-0.0256543339
474.8000000000	4.4098426478	-0.0236501021
475.4000000000	4.2510969605	-0.0217873658

476.0000000000	4.0973361575	-0.0200572712
476.6000000000	3.9484411417	-0.0184514292
477.2000000000	3.8042936649	-0.0169618986
477.8000000000	3.6647764495	-0.0155811684
478.4000000000	3.5297733004	-0.0143021412
479.0000000000	3.3991692082	-0.0131181162
479.6000000000	3.2728504446	-0.0120227723
480.2000000000	3.1507046484	-0.0110101516
480.8000000000	3.0326209059	-0.0100746434
481.4000000000	2.9184898219	-0.0092109680
482.0000000000	2.8082035850	-0.0084141616
482.6000000000	2.7016560257	-0.0076795605
483.2000000000	2.5987426684	-0.0070027870
483.8000000000	2.4993607770	-0.0063797346
484.4000000000	2.4034093953	-0.0058065545
485.0000000000	2.3107893815	-0.0052796418
485.6000000000	2.2214034374	-0.0047956230
486.2000000000	2.1351561338	-0.0043513432
486.8000000000	2.0519539298	-0.0039438543
487.4000000000	1.9717051887	-0.0035704031
488.0000000000	1.8943201900	-0.0032284209
488.6000000000	1.8197111365	-0.0029155123

489.2000000000	1.7477921593	-0.0026294454
489.8000000000	1.6784793179	-0.0023681423
490.4000000000	1.6116905982	-0.0021296693
491.0000000000	1.5473459072	-0.0019122287
491.6000000000	1.4853670650	-0.0017141502
492.2000000000	1.4256777941	-0.0015338830
492.8000000000	1.3682037067	-0.0013699882
493.4000000000	1.3128722897	-0.0012211320
494.0000000000	1.2596128871	-0.0010860782
494.6000000000	1.2083566819	-0.0009636828
495.2000000000	1.1590366745	-0.0008528870
495.8000000000	1.1115876617	-0.0007527121
496.4000000000	1.0659462121	-0.0006622540
497.0000000000	1.0220506421	-0.0005806780
497.6000000000	0.9798409895	-0.0005072139
498.2000000000	0.9392589871	-0.0004411520
498.8000000000	0.9002480341	-0.0003818383
499.4000000000	0.8627531684	-0.0003286710
500.0000000000	0.8267210366	-0.0002810964
500.6000000000	0.7920998647	-0.0002386058
501.2000000000	0.7588394273	-0.0002007319
501.8000000000	0.7268910171	-0.0001670458

502.4000000000	0.6962074137	-0.0001371544
503.0000000000	0.6667428526	-0.0001106974
503.6000000000	0.6384529931	-0.0000873451
504.2000000000	0.6112948873	-0.0000667958
504.8000000000	0.5852269481	-0.0000487738
505.4000000000	0.5602089175	-0.0000330274
506.0000000000	0.5362018353	-0.0000193271
506.6000000000	0.5131680073	-0.0000074634
507.2000000000	0.4910709745	0.0000027542
507.8000000000	0.4698754812	0.0000114991
508.4000000000	0.4495474451	0.0000189288
509.0000000000	0.4300539257	0.0000251864
509.6000000000	0.4113630949	0.0000304016
510.2000000000	0.3934442066	0.0000346920
510.8000000000	0.3762675673	0.0000381638
511.4000000000	0.3598045067	0.0000409132
512.0000000000	0.3440273492	0.0000430269
512.6000000000	0.3289093857	0.0000445830
513.2000000000	0.3144248453	0.0000456520
513.8000000000	0.3005488683	0.0000462971
514.4000000000	0.2872574790	0.0000465752
515.0000000000	0.2745275596	0.0000465369

515.6000000000	0.2623368240	0.0000462279
516.2000000000	0.2506637925	0.0000456888
516.8000000000	0.2394877670	0.0000449558
517.4000000000	0.2287888065	0.0000440610
518.0000000000	0.2185477035	0.0000430329
518.6000000000	0.2087459604	0.0000418969
519.2000000000	0.1993657669	0.0000406752
519.8000000000	0.1903899778	0.0000393875
520.4000000000	0.1818020912	0.0000380508
521.0000000000	0.1735862277	0.0000366803
521.6000000000	0.1657271089	0.0000352890
522.2000000000	0.1582100384	0.0000338882
522.8000000000	0.1510208814	0.0000324877
523.4000000000	0.1441460459	0.0000310958
524.0000000000	0.1375724640	0.0000297195
524.6000000000	0.1312875744	0.0000283650
525.2000000000	0.1252793039	0.0000270370
525.8000000000	0.1195360512	0.0000257399
526.4000000000	0.1140466699	0.0000244768
527.0000000000	0.1088004525	0.0000232504
527.6000000000	0.1037871147	0.0000220629
528.2000000000	0.0989967804	0.0000209156

528.8000000000	0.0944199668	0.0000198097
529.4000000000	0.0900475704	0.0000187457
530.0000000000	0.0858708528	0.0000177241
530.6000000000	0.0818814277	0.0000167447
531.2000000000	0.0780712477	0.0000158074
531.8000000000	0.0744325918	0.0000149117
532.4000000000	0.0709580533	0.0000140569
533.0000000000	0.0676405278	0.0000132422
533.6000000000	0.0644732020	0.0000124668
534.2000000000	0.0614495428	0.0000117295
534.8000000000	0.0585632865	0.0000110292
535.4000000000	0.0558084284	0.0000103648
536.0000000000	0.0531792132	0.0000097351
536.6000000000	0.0506701249	0.0000091388
537.2000000000	0.0482758780	0.0000085746
537.8000000000	0.0459914084	0.0000080413
538.4000000000	0.0438118646	0.0000075375
539.0000000000	0.0417325995	0.0000070620
539.6000000000	0.0397491622	0.0000066136
540.2000000000	0.0378572906	0.0000061910
540.8000000000	0.0360529035	0.0000057929
541.4000000000	0.0343320935	0.0000054182

542.0000000000	0.0326911202	0.0000050657
542.6000000000	0.0311264034	0.0000047344
543.2000000000	0.0296345165	0.0000044231
543.8000000000	0.0282121806	0.0000041307
544.4000000000	0.0268562582	0.0000038563
545.0000000000	0.0255637475	0.0000035990
545.6000000000	0.0243317771	0.0000033576
546.2000000000	0.0231576003	0.0000031315
546.8000000000	0.0220385901	0.0000029196
547.4000000000	0.0209722345	0.0000027213
548.0000000000	0.0199561312	0.0000025356
548.6000000000	0.0189879835	0.0000023620
549.2000000000	0.0180655959	0.0000021996
549.8000000000	0.0171868695	0.0000020478
550.4000000000	0.0163497982	0.0000019059
551.0000000000	0.0155524648	0.0000017735
551.6000000000	0.0147930372	0.0000016498
552.2000000000	0.0140697648	0.0000015343
552.8000000000	0.0133809750	0.0000014266
553.4000000000	0.0127250699	0.0000013261
554.0000000000	0.0121005233	0.0000012324
554.6000000000	0.0115058774	0.0000011451

555.2000000000	0.0109397396	0.0000010637
555.8000000000	0.0104007805	0.0000009878
556.4000000000	0.0098877305	0.0000009172
557.0000000000	0.0093993773	0.0000008515
557.6000000000	0.0089345636	0.0000007903
558.2000000000	0.0084921846	0.0000007333
558.8000000000	0.0080711858	0.0000006804
559.4000000000	0.0076705608	0.0000006311
560.0000000000	0.0072893490	0.0000005853
560.6000000000	0.0069266337	0.0000005427
561.2000000000	0.0065815404	0.0000005031
561.8000000000	0.0062532344	0.0000004663
562.4000000000	0.0059409198	0.0000004322
563.0000000000	0.0056438370	0.0000004004
563.6000000000	0.0053612615	0.0000003710
564.2000000000	0.0050925025	0.0000003436
564.8000000000	0.0048369008	0.0000003182
565.4000000000	0.0045938282	0.0000002947
566.0000000000	0.0043626853	0.0000002728
566.6000000000	0.0041429009	0.0000002526
567.2000000000	0.0039339300	0.0000002338
567.8000000000	0.0037352535	0.0000002163

568.4000000000	0.0035463763	0.0000002002
569.0000000000	0.0033668265	0.0000001852
569.6000000000	0.0031961543	0.0000001713
570.2000000000	0.0030339312	0.0000001584
570.8000000000	0.0028797486	0.0000001465
571.4000000000	0.0027332173	0.0000001355
572.0000000000	0.0025939666	0.0000001253
572.6000000000	0.0024616429	0.0000001158
573.2000000000	0.0023359097	0.0000001070
573.8000000000	0.0022164464	0.0000000989
574.4000000000	0.0021029474	0.0000000914
575.0000000000	0.0019951219	0.0000000845
575.6000000000	0.0018926926	0.0000000780
576.2000000000	0.0017953958	0.0000000721
576.8000000000	0.0017029801	0.0000000666
577.4000000000	0.0016152063	0.0000000615
578.0000000000	0.0015318464	0.0000000568
578.6000000000	0.0014526836	0.0000000524
579.2000000000	0.0013775114	0.0000000484
579.8000000000	0.0013061332	0.0000000447
580.4000000000	0.0012383618	0.0000000413
581.0000000000	0.0011740191	0.0000000381

581.6000000000	0.0011129356	0.0000000352
582.2000000000	0.0010549499	0.0000000324
582.8000000000	0.0009999086	0.0000000299
583.4000000000	0.0009476656	0.0000000276
584.0000000000	0.0008980817	0.0000000255
584.6000000000	0.0008510248	0.0000000235
585.2000000000	0.0008063690	0.0000000217
585.8000000000	0.0007639946	0.0000000200
586.4000000000	0.0007237878	0.0000000184
587.0000000000	0.0006856402	0.0000000170
587.6000000000	0.0006494487	0.0000000157
588.2000000000	0.0006151155	0.0000000145
588.8000000000	0.0005825473	0.0000000133
589.4000000000	0.0005516554	0.0000000123
590.0000000000	0.0005223557	0.0000000113
590.6000000000	0.0004945680	0.0000000105
591.2000000000	0.0004682162	0.0000000096
591.8000000000	0.0004432277	0.0000000089
592.4000000000	0.0004195337	0.0000000082
593.0000000000	0.0003970689	0.0000000075
593.6000000000	0.0003757709	0.0000000069
594.2000000000	0.0003555806	0.0000000064

594.8000000000	0.0003364418	0.0000000059
595.4000000000	0.0003183011	0.0000000054
596.0000000000	0.0003011078	0.0000000050
596.6000000000	0.0002848135	0.0000000046
597.2000000000	0.0002693725	0.0000000042
597.8000000000	0.0002547412	0.0000000039
598.4000000000	0.0002408782	0.0000000036
599.0000000000	0.0002277443	0.0000000033
599.6000000000	0.0002153020	0.0000000031
600.2000000000	0.0002035159	0.0000000028
600.8000000000	0.0001923522	0.0000000026
601.4000000000	0.0001817791	0.0000000024
602.0000000000	0.0001717661	0.0000000022
602.6000000000	0.0001622843	0.0000000020
603.2000000000	0.0001533063	0.0000000019
603.8000000000	0.0001448060	0.0000000017
604.4000000000	0.0001367589	0.0000000016
605.0000000000	0.0001291413	0.0000000015
605.6000000000	0.0001219311	0.0000000013
606.2000000000	0.0001151070	0.0000000012
606.8000000000	0.0001086490	0.0000000011
607.4000000000	0.0001025380	0.0000000010

608.0000000000	0.0000967560	0.0000000010
608.6000000000	0.0000912857	0.0000000009
609.2000000000	0.0000861109	0.0000000008
609.8000000000	0.0000812160	0.0000000007
610.4000000000	0.0000765866	0.0000000007
611.0000000000	0.0000722085	0.0000000006
611.6000000000	0.0000680686	0.0000000006
612.2000000000	0.0000641544	0.0000000005
612.8000000000	0.0000604539	0.0000000005
613.4000000000	0.0000569559	0.0000000005
614.0000000000	0.0000536497	0.0000000004
614.6000000000	0.0000505252	0.0000000004
615.2000000000	0.0000475726	0.0000000004
615.8000000000	0.0000447829	0.0000000003
616.4000000000	0.0000421475	0.0000000003
617.0000000000	0.0000396580	0.0000000003
617.6000000000	0.0000373068	0.0000000003
618.2000000000	0.0000350864	0.0000000002
618.8000000000	0.0000329899	0.0000000002
619.4000000000	0.0000310105	0.0000000002
620.0000000000	0.0000291421	0.0000000002
620.6000000000	0.0000273787	0.0000000002

621.2000000000	0.0000257146	0.0000000002
621.8000000000	0.0000241444	0.0000000001
622.4000000000	0.0000226631	0.0000000001
623.0000000000	0.0000212658	0.0000000001
623.6000000000	0.0000199481	0.0000000001
624.2000000000	0.0000187056	0.0000000001
624.8000000000	0.0000175342	0.0000000001
625.4000000000	0.0000164300	0.0000000001
626.0000000000	0.0000153894	0.0000000001
626.6000000000	0.0000144088	0.0000000001
627.2000000000	0.0000134851	0.0000000001
627.8000000000	0.0000126150	0.0000000001
628.4000000000	0.0000117957	0.0000000001
629.0000000000	0.0000110243	0.0000000001
629.6000000000	0.0000102981	0.0000000000
630.2000000000	0.0000096147	0.0000000000
630.8000000000	0.0000089717	0.0000000000
631.4000000000	0.0000083669	0.0000000000
632.0000000000	0.0000077981	0.0000000000
632.6000000000	0.0000072633	0.0000000000
633.2000000000	0.0000067606	0.0000000000
633.8000000000	0.0000062883	0.0000000000

634.4000000000	0.0000058445	0.0000000000
635.0000000000	0.0000054277	0.0000000000
635.6000000000	0.0000050364	0.0000000000
636.2000000000	0.0000046691	0.0000000000
636.8000000000	0.0000043244	0.0000000000
637.4000000000	0.0000040011	0.0000000000
638.0000000000	0.0000036980	0.0000000000
638.6000000000	0.0000034139	0.0000000000
639.2000000000	0.0000031476	0.0000000000
639.8000000000	0.0000028982	0.0000000000
640.4000000000	0.0000026648	0.0000000000
641.0000000000	0.0000024462	0.0000000000
641.6000000000	0.0000022418	0.0000000000
642.2000000000	0.0000020507	0.0000000000
642.8000000000	0.0000018721	0.0000000000
643.4000000000	0.0000017052	0.0000000000
644.0000000000	0.0000015494	0.0000000000
644.6000000000	0.0000014040	0.0000000000
645.2000000000	0.0000012684	0.0000000000
645.8000000000	0.0000011421	0.0000000000
646.4000000000	0.0000010244	0.0000000000
647.0000000000	0.0000009148	0.0000000000

647.6000000000	0.0000008129	0.0000000000
648.2000000000	0.0000007182	0.0000000000
648.8000000000	0.0000006302	0.0000000000
649.4000000000	0.0000005486	0.0000000000
650.0000000000	0.0000004729	0.0000000000
650.6000000000	0.0000004028	0.0000000000
651.2000000000	0.0000003379	0.0000000000
651.8000000000	0.0000002780	0.0000000000
652.4000000000	0.0000002226	0.0000000000
653.0000000000	0.0000001716	0.0000000000
653.6000000000	0.0000001246	0.0000000000
654.2000000000	0.0000000813	0.0000000000
654.8000000000	0.0000000416	0.0000000000
655.4000000000	0.0000000053	0.0000000000
656.0000000000	-0.0000000280	0.0000000000
656.6000000000	-0.0000000584	0.0000000000
657.2000000000	-0.0000000861	0.0000000000
657.8000000000	-0.0000001113	0.0000000000
658.4000000000	-0.0000001341	0.0000000000
659.0000000000	-0.0000001547	0.0000000000
659.6000000000	-0.0000001733	0.0000000000
660.2000000000	-0.0000001900	0.0000000000

660.8000000000	-0.0000002049	0.0000000000
661.4000000000	-0.0000002181	0.0000000000
662.0000000000	-0.0000002298	0.0000000000
662.6000000000	-0.0000002401	0.0000000000
663.2000000000	-0.0000002491	0.0000000000
663.8000000000	-0.0000002568	0.0000000000
664.4000000000	-0.0000002634	0.0000000000
665.0000000000	-0.0000002690	0.0000000000
665.6000000000	-0.0000002736	0.0000000000
666.2000000000	-0.0000002772	0.0000000000
666.8000000000	-0.0000002801	0.0000000000
667.4000000000	-0.0000002822	0.0000000000
668.0000000000	-0.0000002836	0.0000000000
668.6000000000	-0.0000002844	0.0000000000
669.2000000000	-0.0000002845	0.0000000000
669.8000000000	-0.0000002841	0.0000000000
670.4000000000	-0.0000002833	0.0000000000
671.0000000000	-0.0000002820	0.0000000000
671.6000000000	-0.0000002802	0.0000000000
672.2000000000	-0.0000002781	0.0000000000
672.8000000000	-0.0000002757	0.0000000000
673.4000000000	-0.0000002730	0.0000000000

674.0000000000	-0.0000002700	0.0000000000
674.6000000000	-0.0000002667	0.0000000000
675.2000000000	-0.0000002633	0.0000000000
675.8000000000	-0.0000002596	0.0000000000
676.4000000000	-0.0000002558	0.0000000000
677.0000000000	-0.0000002519	0.0000000000
677.6000000000	-0.0000002478	0.0000000000
678.2000000000	-0.0000002436	0.0000000000
678.8000000000	-0.0000002393	0.0000000000
679.4000000000	-0.0000002349	0.0000000000
680.0000000000	-0.0000002305	0.0000000000
680.6000000000	-0.0000002260	0.0000000000
681.2000000000	-0.0000002215	0.0000000000
681.8000000000	-0.0000002170	0.0000000000
682.4000000000	-0.0000002125	0.0000000000
683.0000000000	-0.0000002079	0.0000000000
683.6000000000	-0.0000002034	0.0000000000
684.2000000000	-0.0000001989	0.0000000000
684.8000000000	-0.0000001943	0.0000000000
685.4000000000	-0.0000001899	0.0000000000
686.0000000000	-0.0000001854	0.0000000000
686.6000000000	-0.0000001810	0.0000000000

687.2000000000	-0.0000001766	0.0000000000
687.8000000000	-0.0000001723	0.0000000000
688.4000000000	-0.0000001681	0.0000000000
689.0000000000	-0.0000001638	0.0000000000
689.6000000000	-0.0000001597	0.0000000000
690.2000000000	-0.0000001556	0.0000000000
690.8000000000	-0.0000001516	0.0000000000
691.4000000000	-0.0000001476	0.0000000000
692.0000000000	-0.0000001437	0.0000000000
692.6000000000	-0.0000001399	0.0000000000
693.2000000000	-0.0000001361	0.0000000000
693.8000000000	-0.0000001324	0.0000000000
694.4000000000	-0.0000001288	0.0000000000
695.0000000000	-0.0000001253	0.0000000000
695.6000000000	-0.0000001218	0.0000000000
696.2000000000	-0.0000001184	0.0000000000
696.8000000000	-0.0000001151	0.0000000000
697.4000000000	-0.0000001118	0.0000000000
698.0000000000	-0.0000001086	0.0000000000
698.6000000000	-0.0000001055	0.0000000000
699.2000000000	-0.0000001025	0.0000000000
699.8000000000	-0.0000000995	0.0000000000

700.4000000000	-0.0000000966	0.0000000000
701.0000000000	-0.0000000938	0.0000000000
701.6000000000	-0.0000000910	0.0000000000
702.2000000000	-0.0000000883	0.0000000000
702.8000000000	-0.0000000857	0.0000000000
703.4000000000	-0.0000000832	0.0000000000
704.0000000000	-0.0000000807	0.0000000000
704.6000000000	-0.0000000782	0.0000000000
705.2000000000	-0.0000000759	0.0000000000
705.8000000000	-0.0000000736	0.0000000000
706.4000000000	-0.0000000713	0.0000000000
707.0000000000	-0.0000000692	0.0000000000
707.6000000000	-0.0000000670	0.0000000000
708.2000000000	-0.0000000650	0.0000000000
708.8000000000	-0.0000000630	0.0000000000
709.4000000000	-0.0000000610	0.0000000000
710.0000000000	-0.0000000591	0.0000000000
710.6000000000	-0.0000000573	0.0000000000
711.2000000000	-0.0000000555	0.0000000000
711.8000000000	-0.0000000538	0.0000000000
712.4000000000	-0.0000000521	0.0000000000
713.0000000000	-0.0000000504	0.0000000000

713.6000000000	-0.0000000489	0.0000000000
714.2000000000	-0.0000000473	0.0000000000
714.8000000000	-0.0000000458	0.0000000000
715.4000000000	-0.0000000444	0.0000000000
716.0000000000	-0.0000000429	0.0000000000
716.6000000000	-0.0000000416	0.0000000000
717.2000000000	-0.0000000402	0.0000000000
717.8000000000	-0.0000000389	0.0000000000
718.4000000000	-0.0000000377	0.0000000000
719.0000000000	-0.0000000365	0.0000000000
719.6000000000	-0.0000000353	0.0000000000
720.2000000000	-0.0000000342	0.0000000000
720.8000000000	-0.0000000331	0.0000000000
721.4000000000	-0.0000000320	0.0000000000
722.0000000000	-0.0000000309	0.0000000000
722.6000000000	-0.0000000299	0.0000000000
723.2000000000	-0.0000000290	0.0000000000
723.8000000000	-0.0000000280	0.0000000000
724.4000000000	-0.0000000271	0.0000000000
725.0000000000	-0.0000000262	0.0000000000
725.6000000000	-0.0000000254	0.0000000000
726.2000000000	-0.0000000245	0.0000000000

726.8000000000	-0.0000000237	0.0000000000
727.4000000000	-0.0000000229	0.0000000000
728.0000000000	-0.0000000222	0.0000000000
728.6000000000	-0.0000000215	0.0000000000
729.2000000000	-0.0000000207	0.0000000000
729.8000000000	-0.0000000201	0.0000000000
730.4000000000	-0.0000000194	0.0000000000
731.0000000000	-0.0000000188	0.0000000000
731.6000000000	-0.0000000181	0.0000000000
732.2000000000	-0.0000000175	0.0000000000
732.8000000000	-0.0000000169	0.0000000000
733.4000000000	-0.0000000164	0.0000000000
734.0000000000	-0.0000000158	0.0000000000
734.6000000000	-0.0000000153	0.0000000000
735.2000000000	-0.0000000148	0.0000000000
735.8000000000	-0.0000000143	0.0000000000
736.4000000000	-0.0000000138	0.0000000000
737.0000000000	-0.0000000134	0.0000000000
737.6000000000	-0.0000000129	0.0000000000
738.2000000000	-0.0000000125	0.0000000000
738.8000000000	-0.0000000121	0.0000000000
739.4000000000	-0.0000000117	0.0000000000

740.0000000000	-0.0000000113	0.0000000000
740.6000000000	-0.0000000109	0.0000000000
741.2000000000	-0.0000000105	0.0000000000
741.8000000000	-0.0000000102	0.0000000000
742.4000000000	-0.0000000098	0.0000000000
743.0000000000	-0.0000000095	0.0000000000
743.6000000000	-0.0000000092	0.0000000000
744.2000000000	-0.0000000089	0.0000000000
744.8000000000	-0.0000000086	0.0000000000
745.4000000000	-0.0000000083	0.0000000000
746.0000000000	-0.0000000080	0.0000000000
746.6000000000	-0.0000000077	0.0000000000
747.2000000000	-0.0000000075	0.0000000000
747.8000000000	-0.0000000072	0.0000000000
748.4000000000	-0.0000000070	0.0000000000
749.0000000000	-0.0000000067	0.0000000000
749.6000000000	-0.0000000065	0.0000000000
750.2000000000	-0.0000000063	0.0000000000
750.8000000000	-0.0000000061	0.0000000000
751.4000000000	-0.0000000059	0.0000000000
752.0000000000	-0.0000000057	0.0000000000
752.6000000000	-0.0000000055	0.0000000000

753.2000000000	-0.0000000053	0.0000000000
753.8000000000	-0.0000000051	0.0000000000
754.4000000000	-0.0000000049	0.0000000000
755.0000000000	-0.0000000048	0.0000000000
755.6000000000	-0.0000000046	0.0000000000
756.2000000000	-0.0000000045	0.0000000000
756.8000000000	-0.0000000043	0.0000000000
757.4000000000	-0.0000000042	0.0000000000
758.0000000000	-0.0000000040	0.0000000000
758.6000000000	-0.0000000039	0.0000000000
759.2000000000	-0.0000000037	0.0000000000
759.8000000000	-0.0000000036	0.0000000000
760.4000000000	-0.0000000035	0.0000000000
761.0000000000	-0.0000000034	0.0000000000
761.6000000000	-0.0000000033	0.0000000000
762.2000000000	-0.0000000032	0.0000000000
762.8000000000	-0.0000000030	0.0000000000
763.4000000000	-0.0000000029	0.0000000000
764.0000000000	-0.0000000028	0.0000000000
764.6000000000	-0.0000000027	0.0000000000
765.2000000000	-0.0000000027	0.0000000000
765.8000000000	-0.0000000026	0.0000000000

766.4000000000	-0.0000000025	0.0000000000
767.0000000000	-0.0000000024	0.0000000000
767.6000000000	-0.0000000023	0.0000000000
768.2000000000	-0.0000000022	0.0000000000
768.8000000000	-0.0000000022	0.0000000000
769.4000000000	-0.0000000021	0.0000000000
770.0000000000	-0.0000000020	0.0000000000
770.6000000000	-0.0000000019	0.0000000000
771.2000000000	-0.0000000019	0.0000000000
771.8000000000	-0.0000000018	0.0000000000
772.4000000000	-0.0000000017	0.0000000000
773.0000000000	-0.0000000017	0.0000000000
773.6000000000	-0.0000000016	0.0000000000
774.2000000000	-0.0000000016	0.0000000000
774.8000000000	-0.0000000015	0.0000000000
775.4000000000	-0.0000000015	0.0000000000
776.0000000000	-0.0000000014	0.0000000000
776.6000000000	-0.0000000014	0.0000000000
777.2000000000	-0.0000000013	0.0000000000
777.8000000000	-0.0000000013	0.0000000000
778.4000000000	-0.0000000012	0.0000000000
779.0000000000	-0.0000000012	0.0000000000

779.6000000000	-0.0000000012	0.0000000000
780.2000000000	-0.0000000011	0.0000000000
780.8000000000	-0.0000000011	0.0000000000
781.4000000000	-0.0000000010	0.0000000000
782.0000000000	-0.0000000010	0.0000000000
782.6000000000	-0.0000000010	0.0000000000
783.2000000000	-0.0000000009	0.0000000000
783.8000000000	-0.0000000009	0.0000000000
784.4000000000	-0.0000000009	0.0000000000
785.0000000000	-0.0000000008	0.0000000000
785.6000000000	-0.0000000008	0.0000000000
786.2000000000	-0.0000000008	0.0000000000
786.8000000000	-0.0000000008	0.0000000000
787.4000000000	-0.0000000007	0.0000000000
788.0000000000	-0.0000000007	0.0000000000
788.6000000000	-0.0000000007	0.0000000000
789.2000000000	-0.0000000007	0.0000000000
789.8000000000	-0.0000000006	0.0000000000
790.4000000000	-0.0000000006	0.0000000000
791.0000000000	-0.0000000006	0.0000000000
791.6000000000	-0.0000000006	0.0000000000
792.2000000000	-0.0000000006	0.0000000000

792.8000000000	-0.0000000005	0.0000000000
793.4000000000	-0.0000000005	0.0000000000
794.0000000000	-0.0000000005	0.0000000000
794.6000000000	-0.0000000005	0.0000000000
795.2000000000	-0.0000000005	0.0000000000
795.8000000000	-0.0000000005	0.0000000000
796.4000000000	-0.0000000004	0.0000000000
797.0000000000	-0.0000000004	0.0000000000
797.6000000000	-0.0000000004	0.0000000000
798.2000000000	-0.0000000004	0.0000000000
798.8000000000	-0.0000000004	0.0000000000
799.4000000000	-0.0000000004	0.0000000000

Excitation energies and oscillator strengths for optimized SD/*L*-Ag14.

Part 1

Excited State 1: Singlet-A 2.9338 eV 422.60 nm f=0.2058

<S**2>=0.000

1029 ->1030 0.68453

1029 ->1041 -0.10248

This state for optimization and/or second-order correction.

Total Energy, E(TD-HF/TD-DFT) = -18684.6138966

Copying the excited state density for this state as the 1-particle RhoCI density.

Excited State 2: Singlet-A 3.0010 eV 413.15 nm f=0.1288

<S**2>=0.000

1029 ->1031 0.65489

1029 ->1032 -0.19271

Excited State 3: Singlet-A 3.0471 eV 406.90 nm f=0.0599

<S**2>=0.000

1029 ->1031 0.21942

1029 ->1032 0.55759

1029 ->1033 0.29515

1029 ->1034 0.11980

Excited State 4: Singlet-A 3.0655 eV 404.45 nm f=0.0186

<S**2>=0.000

1028 ->1030 -0.20699

1029 ->1032 -0.32943

1029 ->1033 0.54953

Excited State 5: Singlet-A 3.0770 eV 402.94 nm f=0.0094

<S**2>=0.000

1025 ->1030 -0.13045

1027 ->1030 -0.16073

1028 ->1030 0.51818

1028 ->1031 0.13062

1028 ->1032 0.15508

1028 ->1033 -0.10776

1029 ->1033 0.24845

Excited State 6: Singlet-A 3.0919 eV 400.99 nm f=0.0205

<S**2>=0.000

1029 ->1032 -0.10765

1029 ->1034 0.67542

Excited State 7: Singlet-A 3.1093 eV 398.76 nm f=0.0512

<S**2>=0.000

1025 ->1030 -0.11311

1026 ->1030 0.11224

1027 ->1030 0.38712

1027 ->1033 0.26102

1027 ->1034 -0.29601

1028 ->1030 0.14448

1029 ->1035 -0.22315

1029 ->1036 0.11798

1029 ->1037 0.10574

Excited State 8: Singlet-A 3.1201 eV 397.37 nm f=0.0059

<S**2>=0.000

1027 ->1030 0.18802

1027 ->1033 0.11213

1027 ->1034 -0.12660

1029 ->1035 0.62835

Excited State 9: Singlet-A 3.1320 eV 395.86 nm f=0.0023

<S**2>=0.000

1029 ->1035 0.11788

1029 ->1036 0.67450

Excited State 10: Singlet-A 3.1611 eV 392.21 nm f=0.0177

<S**2>=0.000

1029 ->1037 0.67609

Excited State 11: Singlet-A 3.1723 eV 390.84 nm f=0.0100

<S**2>=0.000

1026 ->1030 0.21414

1026 ->1032 0.16102

1026 ->1035 -0.24391

1027 ->1032 -0.13730

1027 ->1035 0.12179

1028 ->1030 -0.12853

1028 ->1031 0.33944

1028 ->1032 -0.22937

1028 ->1033 -0.14895

1028 ->1035 0.14706

1028 ->1036 0.14395

Excited State 12: Singlet-A 3.1968 eV 387.84 nm f=0.0249

<S**2>=0.000

1025 ->1030 0.11096

1025 ->1031 0.18467

1025 ->1033 -0.12659

1025 ->1035 -0.15214

1025 ->1036 -0.10011

1026 ->1030 -0.11669

1026 ->1031 0.29679

1026 ->1032 -0.19773

1026 ->1033 -0.20228

1028 ->1031 0.26085

1028 ->1035 -0.26297

1029 ->1038 0.10728

Excited State 13: Singlet-A 3.2174 eV 385.35 nm f=0.0042

<S**2>=0.000

1029 ->1038 0.67184

Excited State 14: Singlet-A 3.2283 eV 384.05 nm f=0.0460

<S**2>=0.000

1025 ->1031 0.16794

1027 ->1031 0.12775

1028 ->1031 -0.22575

1028 ->1033 -0.11471

1028 ->1036 -0.27128

1029 ->1039 0.45421

Excited State 15: Singlet-A 3.2356 eV 383.19 nm f=0.0091

<S**2>=0.000

1025 ->1031 -0.14008

1027 ->1031 -0.17795

1027 ->1032 0.12192

1028 ->1031 0.14103

1028 ->1033 0.10654

1028 ->1036 0.22435

1029 ->1038 -0.10491

1029 ->1039 0.49997

Excited State 16: Singlet-A 3.2386 eV 382.83 nm f=0.0672

<S**2>=0.000

1025 ->1030 -0.10319

1026 ->1030 0.39063

1026 ->1031 0.24286

1027 ->1030 -0.17704

1027 ->1031 -0.19004

1027 ->1032 0.14577

1027 ->1034 -0.10786

1028 ->1030 -0.18175

1028 ->1031 -0.12574

1028 ->1032 0.14969

1028 ->1033 -0.11716

Excited State 17: Singlet-A 3.2588 eV 380.46 nm f=0.1187

<S**2>=0.000

1025 ->1030 0.16162

1025 ->1031 0.11142

1026 ->1031 -0.11956

1027 ->1030 0.10036

1027 ->1031 -0.20255

1027 ->1032 0.26136

1027 ->1033 0.22895

1027 ->1034 0.15946

1027 ->1037 0.28030

1028 ->1030 0.10801

1028 ->1032 -0.17990

1029 ->1039 -0.13719

Excited State 18: Singlet-A 3.2658 eV 379.64 nm f=0.1027

<S**2>=0.000

1025 ->1030 0.34009

1026 ->1030 0.33309

1027 ->1033 -0.10285

1027 ->1037 -0.11630

1028 ->1030 0.19029

1028 ->1031 -0.14703

1028 ->1032 -0.24455

1028 ->1033 0.14923

1028 ->1035 -0.12062

1028 ->1036 0.13975

Excited State 19: Singlet-A 3.2843 eV 377.50 nm f=0.0181

<S**2>=0.000

1024 ->1030 -0.14201

1025 ->1030 0.20194

1026 ->1030 0.12260

1027 ->1030 0.26943

1027 ->1031 -0.25263

1027 ->1032 -0.13683

1027 ->1033 -0.13935

1027 ->1034 0.14483

1027 ->1037 -0.16993

1028 ->1031 0.14936

1028 ->1032 0.27916

1028 ->1033 -0.10461

1028 ->1035 0.14751

1028 ->1036 -0.11369

Excited State 20: Singlet-A 3.3022 eV 375.45 nm f=0.0681

<S**2>=0.000

1025 ->1030 0.23859

1026 ->1030 0.14948

1027 ->1030 -0.12926

1027 ->1031 0.33450

1027 ->1033 0.10317

1027 ->1037 0.23781

1028 ->1031 0.14444

1028 ->1032 0.25084

1028 ->1033 0.14444

Excited State 21: Singlet-A 3.3155 eV 373.95 nm f=0.0262

<S**2>=0.000

1024 ->1030 -0.11324

1025 ->1030 -0.22397

1026 ->1030 0.15463

1026 ->1033 -0.13145

1027 ->1031 -0.16874

1027 ->1033 0.11840

1027 ->1037 -0.12002

1028 ->1032 -0.15372

1028 ->1033 0.34587

1028 ->1034 0.17735

1028 ->1035 -0.11303

1028 ->1036 -0.22853

Excited State 22: Singlet-A 3.3221 eV 373.21 nm f=0.0191

<S**2>=0.000

1024 ->1030 0.27619

1025 ->1030 -0.13923

1025 ->1031 0.10814

1026 ->1030 0.10693

1026 ->1033 -0.16696

1027 ->1032 -0.17092

1027 ->1033 0.11373

1027 ->1034 0.30626

1027 ->1035 -0.16537

1027 ->1036 -0.10580

1028 ->1031 -0.18295

1028 ->1033 -0.11264

1028 ->1036 0.17131

Excited State 23: Singlet-A 3.3387 eV 371.36 nm f=0.0519

<S**2>=0.000

1025 ->1030 0.16185

1026 ->1031 -0.12783

1026 ->1033 -0.14568

1026 ->1035 0.11613

1027 ->1030 -0.21610

1027 ->1033 0.31800

1027 ->1035 0.27663

1027 ->1037 -0.23256

1028 ->1033 -0.15888

Excited State 24: Singlet-A 3.3503 eV 370.07 nm f=0.0481

<S**2>=0.000

1023 ->1030 -0.12128

1024 ->1030 0.36109

1024 ->1032 0.13283

1025 ->1031 0.23020

1026 ->1032 0.15145

1026 ->1033 0.10427

1027 ->1034 -0.13098

1027 ->1035 0.12896

1028 ->1032 0.18583

1028 ->1033 0.24974

1028 ->1036 -0.12905

Excited State 25: Singlet-A 3.3572 eV 369.31 nm f=0.0259

<S**2>=0.000

1026 ->1031 0.15216

1026 ->1032 0.10454

1026 ->1038 -0.18762

1027 ->1031 0.19818

1027 ->1032 0.28584

1027 ->1035 -0.16400

1027 ->1036 -0.16941

1027 ->1037 -0.23603

1028 ->1034 0.18588

1028 ->1035 0.11781

1028 ->1036 -0.10368

1028 ->1038 0.21865

Excited State 26: Singlet-A 3.3610 eV 368.89 nm f=0.0739

<S**2>=0.000

1024 ->1030 -0.23313

1024 ->1032 -0.10690

1025 ->1031 0.33536

1026 ->1032 0.19697

1027 ->1032 0.14530

1027 ->1037 -0.10092

1027 ->1038 -0.12476

1028 ->1031 -0.10612

1028 ->1032 0.15912

1028 ->1033 0.11742

1028 ->1036 0.28023

Excited State 27: Singlet-A 3.3680 eV 368.13 nm f=0.0418

<S**2>=0.000

1024 ->1030 0.11939

1025 ->1033 -0.12996

1026 ->1031 -0.15000

1026 ->1032 -0.17448

1026 ->1034 -0.19843

1026 ->1037 -0.14732

1027 ->1032 0.22878

1027 ->1033 -0.12230

1027 ->1038 -0.10889

1028 ->1033 -0.11028

1028 ->1034 0.34609

1028 ->1037 0.15953

1028 ->1038 -0.15825

Excited State 28: Singlet-A 3.3733 eV 367.54 nm f=0.0557

<S**2>=0.000

1025 ->1030 0.10184

1026 ->1031 0.22570

1026 ->1033 0.15211

1026 ->1036 0.17839

1027 ->1032 -0.16769

1027 ->1034 0.19449

1027 ->1035 0.30061

1027 ->1037 0.11477

1027 ->1038 0.12274

1028 ->1031 -0.12166

1028 ->1034 0.30046

Excited State 29: Singlet-A 3.3761 eV 367.24 nm f=0.0665

<S**2>=0.000

1025 ->1032 0.29499

1025 ->1033 0.10956

1026 ->1031 0.19751

1026 ->1033 0.13225

1026 ->1035 0.13512

1026 ->1038 0.10346

1027 ->1031 0.10701

1027 ->1033 0.12103

1027 ->1034 0.24519

1028 ->1031 0.10239

1028 ->1034 -0.17413

1028 ->1037 0.11948

1028 ->1038 -0.17501

Excited State 30: Singlet-A 3.3838 eV 366.40 nm f=0.0092

<S**2>=0.000

1025 ->1030 -0.11633

1025 ->1031 -0.10324

1025 ->1032 -0.11361

1025 ->1033 -0.16851

1026 ->1031 -0.17979

1026 ->1032 -0.16582

1026 ->1033 0.24835

1026 ->1035 -0.11942

1026 ->1036 -0.10506

1027 ->1031 0.15452

1027 ->1034 0.23758

1027 ->1035 0.13707

1028 ->1032 0.11466

1028 ->1034 -0.11301

1028 ->1035 -0.22078

1028 ->1038 0.15564

Excited State 31: Singlet-A 3.4007 eV 364.58 nm f=0.0264

<S**2>=0.000

1025 ->1031 -0.16245

1025 ->1032 -0.22676

1025 ->1033 -0.13253

1025 ->1034 -0.18086

1026 ->1032 -0.12744

1026 ->1034 0.22470

1026 ->1035 -0.15309

1027 ->1033 0.19564

1027 ->1036 -0.16663

1028 ->1033 0.15850

1028 ->1035 0.23480

1028 ->1038 -0.13725

Excited State 32: Singlet-A 3.4141 eV 363.15 nm f=0.0229

<S**2>=0.000

1023 ->1030 -0.12395

1024 ->1036 -0.13275

1025 ->1031 -0.17031

1025 ->1034 0.12824

1026 ->1032 0.17343

1026 ->1034 -0.19287

1026 ->1035 -0.18483

1026 ->1036 0.13123

1026 ->1039 -0.14482

1027 ->1036 -0.14098

1028 ->1034 -0.16705

1028 ->1035 -0.12915

1028 ->1037 0.21424

1028 ->1038 -0.12399

1028 ->1039 -0.18517

Excited State 33: Singlet-A 3.4162 eV 362.93 nm f=0.0965

<S**2>=0.000

1025 ->1039 -0.14537

1026 ->1031 -0.11611

1026 ->1034 0.36313

1026 ->1039 -0.22781

1027 ->1036 0.10669

1028 ->1034 0.11311

1028 ->1035 -0.15381

1028 ->1039 -0.28795

1029 ->1040 -0.10330

Excited State 34: Singlet-A 3.4233 eV 362.17 nm f=0.0900

<S**2>=0.000

1022 ->1030 -0.11555

1023 ->1030 0.34820

1023 ->1031 0.11403

1023 ->1033 0.15470

1023 ->1034 -0.15805

1024 ->1030 0.12064

1025 ->1033 0.20140

1025 ->1034 -0.15005

1026 ->1032 -0.13671

1026 ->1033 0.15162

1026 ->1034 -0.16021

1027 ->1037 -0.10436

1028 ->1037 0.12539

Excited State 35: Singlet-A 3.4252 eV 361.97 nm f=0.0063

<S**2>=0.000

1023 ->1036 -0.12263

1024 ->1031 0.35207

1024 ->1035 -0.12406

1024 ->1036 0.37110

1026 ->1031 0.11035

1028 ->1037 0.13388

1028 ->1038 -0.12345

Excited State 36: Singlet-A 3.4338 eV 361.07 nm f=0.0256

<S**2>=0.000

1023 ->1030 0.12383

1025 ->1031 -0.11689

1025 ->1033 -0.10485

1025 ->1035 0.13415

1025 ->1039 0.13777

1026 ->1032 0.22850

1026 ->1033 -0.14767

1026 ->1034 0.10989

1026 ->1037 -0.10118

1026 ->1038 0.10152

1026 ->1039 0.14357

1027 ->1032 0.11285

1027 ->1033 -0.12506

1027 ->1035 0.17460

1028 ->1034 0.11498

1028 ->1035 -0.18683

1028 ->1036 -0.14182

1028 ->1037 -0.12088

1028 ->1039 0.10737

Excited State 37: Singlet-A 3.4394 eV 360.48 nm f=0.0347

<S**2>=0.000

1024 ->1036 0.11221

1025 ->1030 -0.11715

1025 ->1034 0.10473

1025 ->1039 -0.10316

1026 ->1030 0.11826

1026 ->1032 -0.15397

1026 ->1033 -0.25181

1026 ->1036 0.26262

1027 ->1032 0.20650

1027 ->1033 -0.10955

1027 ->1035 0.22353

1028 ->1033 0.13767

1028 ->1034 -0.16341

1028 ->1035 0.19231

Excited State 38: Singlet-A 3.4526 eV 359.11 nm f=0.0088

<S**2>=0.000

1025 ->1031 0.12043

1025 ->1033 0.14863

1025 ->1036 0.22238

1026 ->1032 -0.19669

1026 ->1035 -0.17210

1026 ->1036 0.29256

1026 ->1037 -0.15488

1026 ->1038 0.16383

1027 ->1038 -0.20605

1028 ->1036 -0.12727

1029 ->1040 0.10510

Excited State 39: Singlet-A 3.4624 eV 358.09 nm f=0.0191

<S**2>=0.000

1025 ->1033 0.24765

1025 ->1034 -0.21478

1025 ->1037 0.18298

1025 ->1038 -0.10821

1026 ->1036 -0.16611

1026 ->1038 0.10673

1027 ->1035 0.12122

1027 ->1038 -0.20781

1028 ->1038 0.28054

1028 ->1039 -0.12052

Excited State 40: Singlet-A 3.4638 eV 357.95 nm f=0.0253

<S**2>=0.000

1025 ->1032 0.35638

1025 ->1035 0.12089

1025 ->1036 -0.26875

1026 ->1031 -0.15637

1027 ->1036 -0.31554

1027 ->1037 0.11190

Excited State 41: Singlet-A 3.4721 eV 357.09 nm f=0.0229

<S**2>=0.000

1023 ->1030 -0.22246

1023 ->1033 -0.12400

1025 ->1033 0.23793

1025 ->1034 -0.20205

1025 ->1039 0.16239

1026 ->1034 0.11723

1026 ->1036 0.10537

1026 ->1037 0.11878

1026 ->1039 0.13117

1027 ->1035 0.10932

1027 ->1038 0.16273

1027 ->1039 0.11947

1028 ->1037 0.14487

Excited State 42: Singlet-A 3.4781 eV 356.47 nm f=0.0036

<S**2>=0.000

1025 ->1034 0.12542

1025 ->1036 0.13249

1025 ->1039 0.11440

1026 ->1033 -0.18145

1026 ->1035 -0.14749

1026 ->1036 -0.14970

1026 ->1037 -0.21059

1026 ->1038 -0.13300

1027 ->1033 0.10229

1027 ->1036 0.18784

1028 ->1037 0.28329

1028 ->1038 0.21672

Excited State 43: Singlet-A 3.4886 eV 355.39 nm f=0.0447

<S**2>=0.000

1023 ->1031 0.13277

1023 ->1032 -0.18331

1023 ->1033 -0.10846

1023 ->1035 0.10311

1024 ->1031 0.12516

1025 ->1035 0.15141

1026 ->1035 0.16163

1026 ->1036 -0.13426

1026 ->1039 -0.10894

1027 ->1035 0.11237

1028 ->1035 0.12063

1028 ->1039 -0.14761

1029 ->1040 0.36965

Excited State 44: Singlet-A 3.4929 eV 354.96 nm f=0.0692

<S**2>=0.000

1025 ->1035 -0.20149

1025 ->1037 0.20903

1026 ->1034 0.13444

1026 ->1037 0.19690

1027 ->1039 -0.10171

1028 ->1039 0.14392

1029 ->1040 0.43494

Excited State 45: Singlet-A 3.5022 eV 354.02 nm f=0.0202

<S**2>=0.000

1021 ->1030 0.10949

1022 ->1030 0.12265

1023 ->1031 0.16332

1023 ->1032 -0.14474

1023 ->1035 0.12513

1023 ->1037 -0.13055

1025 ->1039 -0.12662

1027 ->1036 0.19943

1027 ->1038 -0.11081

1027 ->1039 -0.22771

1028 ->1039 0.27980

1029 ->1040 -0.15514

Excited State 46: Singlet-A 3.5057 eV 353.67 nm f=0.0047

<S**2>=0.000

1023 ->1031 0.11206

1025 ->1031 0.12053

1025 ->1032 -0.21328

1025 ->1033 0.18397

1025 ->1034 0.29543

1026 ->1034 0.20849

1026 ->1036 -0.19134

1026 ->1037 -0.16956

1027 ->1036 -0.19872

1027 ->1037 0.11291

1027 ->1038 0.10380

1028 ->1039 0.11075

1029 ->1040 -0.11692

Excited State 47: Singlet-A 3.5141 eV 352.82 nm f=0.0139

<S**2>=0.000

1022 ->1030 -0.17388

1023 ->1034 -0.10756

1025 ->1033 -0.16770

1025 ->1034 -0.18100

1025 ->1035 -0.11652

1026 ->1032 0.11933

1026 ->1035 0.28429

1026 ->1036 0.17991

1026 ->1037 -0.16981

1027 ->1036 -0.14802

1028 ->1037 0.25899

1028 ->1038 0.16301

Excited State 48: Singlet-A 3.5189 eV 352.34 nm f=0.0484

<S**2>=0.000

1022 ->1030 0.37145

1022 ->1031 -0.18685

1024 ->1032 0.15279

1025 ->1034 -0.15849

1025 ->1036 0.23317

1026 ->1037 -0.12427

1027 ->1036 -0.11447

1027 ->1038 0.17744

1029 ->1041 -0.13773

Excited State 49: Singlet-A 3.5248 eV 351.75 nm f=0.0411

<S**2>=0.000

1022 ->1030 0.32462

1025 ->1036 -0.24897

1025 ->1039 0.12966

1026 ->1035 0.16811

1026 ->1036 0.15686

1027 ->1036 0.10690

1027 ->1038 -0.18417

1028 ->1037 0.13285

1028 ->1039 -0.16041

Excited State 50: Singlet-A 3.5347 eV 350.76 nm f=0.0124

<S**2>=0.000

1023 ->1032 -0.11592

1023 ->1033 0.10330

1024 ->1030 -0.21685

1024 ->1031 0.34937

1024 ->1032 0.23106

1024 ->1033 -0.11284

1024 ->1035 0.12970

1024 ->1036 -0.12811

1025 ->1032 0.12123

1025 ->1036 -0.12612

Excited State 51: Singlet-A 3.5410 eV 350.14 nm f=0.0441

<S**2>=0.000

1023 ->1031 0.19543

1023 ->1032 -0.15639

1023 ->1035 0.11066

1024 ->1032 -0.13629

1025 ->1033 -0.16148

1025 ->1035 -0.19555

1025 ->1036 0.14396

1026 ->1037 0.19973

1026 ->1038 0.19985

1027 ->1038 -0.11902

1027 ->1039 0.26897

1028 ->1035 0.10865

Excited State 52: Singlet-A 3.5487 eV 349.38 nm f=0.0315

<S**2>=0.000

1022 ->1031 0.34600

1022 ->1034 -0.10065

1023 ->1030 0.22801

1023 ->1031 -0.13149

1023 ->1032 -0.18100

1023 ->1034 0.19066

1023 ->1037 -0.13074

1024 ->1033 -0.10819

1024 ->1034 0.10336

1026 ->1037 -0.13177

Excited State 53: Singlet-A 3.5534 eV 348.92 nm f=0.0083

<S**2>=0.000

1022 ->1032 0.15716

1023 ->1031 -0.19208

1023 ->1033 -0.13919

1023 ->1037 -0.13531

1025 ->1034 0.14237

1025 ->1035 0.14717

1026 ->1037 0.24826

1026 ->1038 -0.13001

1027 ->1038 -0.20267

1028 ->1037 0.16540

1028 ->1038 0.12789

1028 ->1039 0.14687

1029 ->1041 -0.18668

Excited State 54: Singlet-A 3.5581 eV 348.45 nm f=0.0428

<S**2>=0.000

1022 ->1031 0.21166

1022 ->1032 -0.13394

1022 ->1033 -0.13556

1023 ->1033 0.12142

1024 ->1035 0.12196

1025 ->1035 0.20095

1025 ->1036 0.27607

1026 ->1036 -0.11513

1026 ->1037 0.15969

1027 ->1036 -0.19088

1027 ->1038 -0.10984

1027 ->1039 -0.17269

1029 ->1041 0.17756

Excited State 55: Singlet-A 3.5647 eV 347.81 nm f=0.0578

<S**2>=0.000

1021 ->1034 0.13308

1023 ->1031 0.22878

1023 ->1032 -0.16595

1023 ->1034 0.22912

1023 ->1037 0.19160

1024 ->1032 0.13555

1024 ->1036 0.13308

1024 ->1038 0.10752

1025 ->1034 -0.13704

1025 ->1035 0.12653

1027 ->1038 -0.15782

1029 ->1041 -0.12259

Excited State 56: Singlet-A 3.5662 eV 347.66 nm f=0.0139

<S**2>=0.000

1024 ->1032 -0.16193

1025 ->1035 0.32020

1025 ->1037 0.22160

1026 ->1038 0.24710

1027 ->1036 0.15853

1027 ->1037 -0.12574

1027 ->1038 0.22980

Excited State 57: Singlet-A 3.5705 eV 347.25 nm f=0.0063

<S**2>=0.000

1021 ->1030 -0.10801

1023 ->1033 -0.11239

1023 ->1034 -0.12250

1025 ->1037 0.14626

1025 ->1039 -0.19252

1026 ->1038 -0.20063

1027 ->1039 0.41031

1028 ->1038 -0.13406

1029 ->1041 0.17639

Excited State 58: Singlet-A 3.5722 eV 347.09 nm f=0.0385

<S**2>=0.000

1023 ->1032 -0.13092

1023 ->1034 -0.14767

1024 ->1031 -0.18464

1024 ->1032 0.38237

1024 ->1036 0.21182

1024 ->1037 -0.12494

1025 ->1034 0.14180

1026 ->1038 0.18608

1027 ->1038 0.11243

Excited State 59: Singlet-A 3.5841 eV 345.93 nm f=0.0536

<S**2>=0.000

1020 ->1030 -0.10067

1021 ->1030 0.24568

1022 ->1030 -0.11060

1022 ->1032 -0.12281

1022 ->1035 -0.11815

1023 ->1030 -0.12176

1023 ->1033 0.21379

1023 ->1034 0.11046

1023 ->1037 -0.10737

1025 ->1039 -0.22000

1027 ->1039 0.23043

1028 ->1039 0.10180

1029 ->1040 0.11792

1029 ->1042 0.13531

Excited State 60: Singlet-A 3.5894 eV 345.42 nm f=0.0008

<S**2>=0.000

1022 ->1031 0.14721

1023 ->1032 -0.14203

1023 ->1035 0.12055

1024 ->1033 0.28931

1024 ->1036 -0.12404

1025 ->1034 -0.10308

1025 ->1037 -0.23568

1025 ->1038 -0.10041

1025 ->1039 -0.12759

1026 ->1038 0.11233

1028 ->1039 0.10995

1029 ->1041 -0.23812

Excited State 61: Singlet-A 3.6003 eV 344.37 nm f=0.0046

<S**2>=0.000

1022 ->1031 -0.16046

1022 ->1033 0.13334

1023 ->1033 -0.14937

1024 ->1033 0.38300

1024 ->1034 0.17588

1024 ->1035 -0.12385

1025 ->1035 0.10910

1025 ->1039 0.13981

1029 ->1041 0.23576

Excited State 62: Singlet-A 3.6048 eV 343.94 nm f=0.0033

<S**2>=0.000

1022 ->1031 -0.14138

1023 ->1038 0.16303

1024 ->1031 -0.10823

1024 ->1032 -0.13629

1024 ->1034 0.21107

1024 ->1035 0.37248

1024 ->1036 0.17105

1024 ->1038 -0.12559

1024 ->1039 0.10485

1025 ->1037 -0.15423

Excited State 63: Singlet-A 3.6086 eV 343.58 nm f=0.0055

<S**2>=0.000

1023 ->1034 0.13994

1024 ->1033 -0.20255

1024 ->1035 -0.14401

1025 ->1037 -0.32316

1025 ->1038 -0.10837

1029 ->1041 0.37193

Excited State 64: Singlet-A 3.6292 eV 341.63 nm f=0.0126

<S**2>=0.000

1018 ->1030 -0.10842

1020 ->1030 -0.21336

1021 ->1030 -0.14493

1021 ->1031 -0.15066

1021 ->1033 -0.17463

1022 ->1031 0.13401

1022 ->1033 0.19374

1023 ->1031 0.19131

1023 ->1032 0.12092

1023 ->1034 -0.16630

1023 ->1038 -0.12387

1024 ->1034 0.26559

1024 ->1035 0.10069

Excited State 65: Singlet-A 3.6397 eV 340.65 nm f=0.0289

<S**2>=0.000

1018 ->1030 0.10998

1020 ->1030 0.13995

1021 ->1033 0.12074

1022 ->1031 0.14291

1022 ->1032 0.28673

1023 ->1030 -0.15201

1023 ->1035 -0.11671

1024 ->1034 0.10622

1025 ->1039 -0.20577

1026 ->1039 0.29767

1029 ->1042 -0.13032

Excited State 66: Singlet-A 3.6432 eV 340.31 nm f=0.0360

<S**2>=0.000

1018 ->1030 -0.15496

1020 ->1030 0.14840

1020 ->1031 -0.12535

1021 ->1030 -0.15648

1022 ->1031 -0.10743

1022 ->1033 0.16464

1023 ->1031 -0.19292

1023 ->1032 -0.20038

1023 ->1033 0.21403

1023 ->1038 -0.11667

1024 ->1032 -0.14000

1024 ->1037 -0.14154

1024 ->1038 0.16808

1026 ->1039 0.18231

Excited State 67: Singlet-A 3.6497 eV 339.71 nm f=0.0228

<S**2>=0.000

1022 ->1032 -0.15032

1022 ->1033 -0.11415

1023 ->1030 0.12699

1023 ->1038 0.12115

1024 ->1036 0.12100

1024 ->1038 -0.15938

1025 ->1039 -0.26940

1026 ->1039 0.36213

1028 ->1039 -0.12307

Excited State 68: Singlet-A 3.6534 eV 339.36 nm f=0.0196

<S**2>=0.000

1018 ->1030 0.17101

1020 ->1030 0.10304

1022 ->1030 -0.12010

1022 ->1033 -0.19819

1022 ->1034 0.16330

1023 ->1032 0.12586

1023 ->1033 -0.15380

1023 ->1034 0.12649

1023 ->1038 -0.13847

1024 ->1035 0.18313

1024 ->1036 -0.10337

1024 ->1037 -0.18821

1024 ->1038 0.19016

1024 ->1039 0.13482

1029 ->1042 0.21876

Excited State 69: Singlet-A 3.6581 eV 338.93 nm f=0.0090

<S**2>=0.000

1020 ->1031 -0.12674

1021 ->1030 0.29515

1021 ->1034 -0.15507

1022 ->1034 -0.10547

1022 ->1035 0.10906

1023 ->1030 0.12431

1023 ->1033 -0.18785

1023 ->1036 0.17118

1023 ->1037 0.30544

Excited State 70: Singlet-A 3.6649 eV 338.30 nm f=0.0298

<S**2>=0.000

1020 ->1030 -0.21354

1022 ->1034 -0.11266

1023 ->1035 0.10995

1024 ->1034 -0.12661

1025 ->1038 0.42976

1026 ->1038 0.19725

1029 ->1042 -0.11830

Excited State 71: Singlet-A 3.6707 eV 337.77 nm f=0.0043

<S**2>=0.000

1021 ->1031 -0.11435

1022 ->1032 -0.10350

1022 ->1034 0.16021

1023 ->1031 -0.14194

1023 ->1035 0.18640

1024 ->1031 -0.12139

1024 ->1033 -0.12414

1024 ->1034 0.29214

1024 ->1035 -0.12936

1024 ->1038 0.10390

1024 ->1039 -0.26841

1025 ->1038 0.18180

1026 ->1038 0.11704

Excited State 72: Singlet-A 3.6732 eV 337.54 nm f=0.0678

<S**2>=0.000

1018 ->1030 -0.11697

1019 ->1030 0.10165

1020 ->1030 0.20163

1020 ->1032 -0.11357

1022 ->1035 -0.12892

1023 ->1031 0.14963

1023 ->1034 -0.10803

1023 ->1035 -0.22349

1023 ->1037 -0.10678

1024 ->1035 -0.13549

1024 ->1038 -0.12894

1025 ->1038 0.33743

1026 ->1038 0.14152

1029 ->1042 0.18988

Excited State 73: Singlet-A 3.6804 eV 336.87 nm f=0.0327

<S**2>=0.000

1020 ->1030 -0.12838

1021 ->1034 -0.16210

1022 ->1032 0.21303

1022 ->1033 0.11385

1022 ->1034 0.15136

1029 ->1042 0.43013

Excited State 74: Singlet-A 3.6895 eV 336.05 nm f=0.0198

<S**2>=0.000

1020 ->1030 -0.14224

1020 ->1031 0.13238

1021 ->1034 0.10898

1022 ->1037 -0.17472

1023 ->1034 -0.18277

1023 ->1036 -0.10610

1023 ->1038 0.29804

1024 ->1036 -0.10711

1024 ->1037 -0.13029

1024 ->1038 0.30575

1028 ->1040 -0.10225

Excited State 75: Singlet-A 3.6967 eV 335.39 nm f=0.0155

<S**2>=0.000

1019 ->1030 0.43595

1020 ->1034 -0.13338

1022 ->1034 0.16498

1023 ->1032 0.13014

1027 ->1040 0.11682

1028 ->1040 -0.20567

1029 ->1042 -0.10633

Excited State 76: Singlet-A 3.7025 eV 334.87 nm f=0.0052

<S**2>=0.000

1020 ->1034 -0.10506

1021 ->1033 -0.15603

1021 ->1034 -0.21229

1021 ->1035 0.12043

1021 ->1037 -0.10810

1022 ->1034 0.16404

1023 ->1034 0.17030

1023 ->1035 -0.11099

1023 ->1038 0.11843

1024 ->1038 0.11043

1027 ->1040 -0.13478

1028 ->1040 0.34002

1029 ->1042 -0.12511

Excited State 77: Singlet-A 3.7046 eV 334.67 nm f=0.0322

<S**2>=0.000

1019 ->1030 0.11804

1020 ->1030 -0.11427

1021 ->1031 0.20632

1021 ->1032 -0.17625

1021 ->1033 -0.11741

1021 ->1034 0.11458

1022 ->1031 -0.11906

1022 ->1032 0.20856

1022 ->1035 -0.16855

1022 ->1036 -0.17165

1023 ->1035 0.13746

1023 ->1038 -0.16196

1024 ->1034 0.13961

1024 ->1039 -0.16667

Excited State 78: Singlet-A 3.7126 eV 333.96 nm f=0.0023

<S**2>=0.000

1018 ->1030 -0.11691

1018 ->1036 -0.10604

1020 ->1030 0.18475

1020 ->1031 -0.16150

1022 ->1033 0.10795

1022 ->1034 -0.18895

1022 ->1035 0.18417

1022 ->1036 -0.22835

1023 ->1031 0.17513

1023 ->1032 0.23370

1023 ->1035 0.14095

1023 ->1036 -0.11490

1024 ->1032 0.11687

1024 ->1034 0.12741

Excited State 79: Singlet-A 3.7153 eV 333.71 nm f=0.0014

<S**2>=0.000

1018 ->1030 -0.11492

1018 ->1031 0.10360

1020 ->1033 0.10836

1021 ->1034 0.19801

1022 ->1033 0.12891

1022 ->1036 0.22842

1022 ->1039 -0.10333

1023 ->1032 0.15736

1023 ->1035 0.16790

1024 ->1033 0.13729

1024 ->1034 -0.12729

1024 ->1035 0.17170

1024 ->1038 0.12912

1024 ->1039 -0.16910

1029 ->1042 0.16346

Excited State 80: Singlet-A 3.7252 eV 332.82 nm f=0.0063

<S**2>=0.000

1019 ->1030 0.20388

1020 ->1030 -0.12118

1020 ->1031 -0.11792

1021 ->1034 0.17999

1022 ->1033 -0.19632

1022 ->1035 0.28110

1022 ->1037 -0.10882

1023 ->1034 -0.12185

1023 ->1035 -0.12878

1023 ->1037 -0.12265

1023 ->1038 -0.17873

1027 ->1040 -0.12348

1028 ->1040 0.18305

Excited State 81: Singlet-A 3.7273 eV 332.64 nm f=0.0039

<S**2>=0.000

1018 ->1030 -0.15034

1020 ->1031 0.15323

1020 ->1034 -0.10536

1021 ->1030 0.12560

1021 ->1031 -0.13311

1021 ->1032 0.15834

1022 ->1032 0.14800

1022 ->1039 0.16316

1024 ->1035 -0.12138

1024 ->1039 0.14203

1027 ->1040 0.22158

1028 ->1040 0.27054

Excited State 82: Singlet-A 3.7314 eV 332.28 nm f=0.0075

<S**2>=0.000

1018 ->1031 0.14723

1019 ->1031 -0.10099

1020 ->1030 -0.13317

1020 ->1031 -0.18834

1020 ->1034 0.15014

1022 ->1033 -0.11692

1022 ->1036 0.15019

1022 ->1037 -0.18687

1023 ->1033 0.10577

1023 ->1035 0.13611

1023 ->1036 0.28428

1023 ->1039 -0.11533

1024 ->1035 -0.13671

1024 ->1039 0.11621

Excited State 83: Singlet-A 3.7329 eV 332.14 nm f=0.0061

<S**2>=0.000

1018 ->1030 0.11302

1019 ->1030 0.10050

1019 ->1032 0.11513

1020 ->1031 0.14368

1020 ->1033 0.19025

1021 ->1032 -0.13462

1022 ->1033 0.21103

1022 ->1036 0.14851

1023 ->1035 0.14236

1024 ->1033 -0.11686

1024 ->1038 -0.16930

1024 ->1039 0.13811

1027 ->1040 -0.19394

1029 ->1042 0.11978

Excited State 84: Singlet-A 3.7444 eV 331.12 nm f=0.0053

<S**2>=0.000

1018 ->1030 0.32671

1019 ->1030 0.18119

1021 ->1030 -0.10425

1022 ->1032 -0.10350

1022 ->1034 -0.16987

1022 ->1035 -0.10618

1024 ->1039 -0.10734

1027 ->1040 0.24826

1028 ->1040 0.25830

Excited State 85: Singlet-A 3.7489 eV 330.72 nm f=0.0103

<S**2>=0.000

1020 ->1032 0.20805

1020 ->1033 0.12174

1021 ->1037 -0.10148

1022 ->1035 0.13132

1022 ->1037 -0.14253

1022 ->1039 0.13961

1023 ->1034 0.11734

1023 ->1035 -0.11309

1023 ->1036 0.27155

1023 ->1037 -0.21083

1024 ->1039 -0.20442

1027 ->1040 0.20018

1028 ->1040 -0.13328

Excited State 86: Singlet-A 3.7561 eV 330.09 nm f=0.0079

<S**2>=0.000

1018 ->1030 -0.13685

1019 ->1031 -0.15168

1020 ->1036 0.10498

1021 ->1032 -0.10415

1022 ->1037 -0.18496

1023 ->1036 -0.26812

1023 ->1038 -0.13117

1027 ->1040 0.33412

Excited State 87: Singlet-A 3.7620 eV 329.57 nm f=0.0039

<S**2>=0.000

1019 ->1031 -0.15014

1020 ->1031 0.17254

1020 ->1032 -0.14661

1021 ->1031 -0.17616

1021 ->1032 0.13109

1022 ->1034 -0.10379

1022 ->1035 -0.10975

1022 ->1037 -0.14518

1022 ->1039 0.28582

1023 ->1039 -0.10673

1024 ->1039 -0.20575

1027 ->1040 -0.24694

Excited State 88: Singlet-A 3.7672 eV 329.12 nm f=0.0171

<S**2>=0.000

1017 ->1030 -0.13318

1017 ->1035 0.10562

1018 ->1030 0.10262

1019 ->1031 0.21010

1019 ->1032 -0.17820

1019 ->1033 -0.14698

1019 ->1035 0.10608

1022 ->1034 0.13095

1022 ->1039 0.21506

1023 ->1036 -0.12125

1024 ->1037 0.23032

1024 ->1040 -0.10956

1027 ->1041 -0.11909

1028 ->1041 0.22621

Excited State 89: Singlet-A 3.7756 eV 328.39 nm f=0.0047

<S**2>=0.000

1019 ->1031 -0.20290

1021 ->1031 0.20824

1021 ->1034 0.12236

1021 ->1035 -0.16645

1021 ->1037 0.12010

1022 ->1032 -0.15997

1022 ->1033 0.13463

1022 ->1034 0.29752

1022 ->1037 0.12009

1022 ->1039 0.11482

1023 ->1036 0.11275

1024 ->1039 -0.11766

Excited State 90: Singlet-A 3.7827 eV 327.77 nm f=0.0010

<S**2>=0.000

1018 ->1030 -0.13724

1020 ->1037 -0.10254

1021 ->1030 -0.10367

1021 ->1031 0.11987

1021 ->1034 -0.10057

1022 ->1033 -0.14185

1022 ->1035 0.19786

1022 ->1036 0.22712

1022 ->1037 0.24136

1022 ->1038 0.13683

1022 ->1039 0.17931

1023 ->1037 0.16340

1024 ->1039 -0.14972

Excited State 91: Singlet-A 3.7901 eV 327.13 nm f=0.0002

<S**2>=0.000

1019 ->1031 0.14106

1020 ->1032 -0.10743

1020 ->1036 -0.10188

1021 ->1030 -0.15633

1021 ->1033 0.34315

1021 ->1035 0.14437

1022 ->1032 -0.12278

1022 ->1034 0.10451

1026 ->1040 -0.21515

1027 ->1041 0.12769

1028 ->1041 -0.12416

Excited State 92: Singlet-A 3.7935 eV 326.83 nm f=0.0126

<S**2>=0.000

1021 ->1037 -0.10185

1023 ->1037 0.10677

1024 ->1037 -0.14519

1026 ->1040 0.53053

1027 ->1041 0.14610

Excited State 93: Singlet-A 3.7981 eV 326.44 nm f=0.0057

<S**2>=0.000

1019 ->1031 0.14177

1020 ->1031 0.26263

1020 ->1032 -0.17421

1020 ->1033 -0.16898

1021 ->1031 0.10613

1021 ->1033 -0.13245

1022 ->1035 0.27189

1022 ->1037 -0.14395

1022 ->1039 -0.12369

1023 ->1036 0.14960

Excited State 94: Singlet-A 3.8027 eV 326.04 nm f=0.0015

<S**2>=0.000

1019 ->1031 -0.20190

1019 ->1032 0.15964

1020 ->1031 0.13173

1020 ->1032 -0.12967

1021 ->1030 -0.11563

1021 ->1035 0.15856

1022 ->1035 0.16791

1022 ->1037 0.24308

1023 ->1037 -0.11401

1024 ->1037 0.25067

1024 ->1038 0.17030

1024 ->1039 0.10353

1026 ->1040 0.14875

Excited State 95: Singlet-A 3.8106 eV 325.37 nm f=0.0049

<S**2>=0.000

1016 ->1030 -0.10218

1019 ->1033 0.18928

1019 ->1035 -0.11528

1021 ->1031 0.14956

1021 ->1033 0.12440

1021 ->1035 -0.18485

1021 ->1037 0.11881

1023 ->1037 -0.14812

1023 ->1038 -0.11519

1024 ->1037 0.29625

1024 ->1038 0.19669

1027 ->1041 0.21577

Excited State 96: Singlet-A 3.8163 eV 324.88 nm f=0.0185

<S**2>=0.000

1018 ->1031 0.24732

1019 ->1035 0.14758

1021 ->1033 -0.12958

1022 ->1036 -0.15199

1027 ->1041 0.43354

1028 ->1041 -0.14243

Excited State 97: Singlet-A 3.8189 eV 324.66 nm f=0.0068

<S**2>=0.000

1017 ->1030 0.13276

1018 ->1031 0.29962

1018 ->1032 0.16380

1019 ->1031 0.14101

1019 ->1032 0.14267

1019 ->1033 0.17190

1019 ->1035 -0.16262

1020 ->1033 -0.14560

1020 ->1035 0.10585

1021 ->1031 -0.10555

1021 ->1032 -0.18355

1027 ->1041 -0.21510

Excited State 98: Singlet-A 3.8220 eV 324.39 nm f=0.0046

<S**2>=0.000

1017 ->1031 -0.12510

1018 ->1031 0.16820

1018 ->1032 -0.11884

1019 ->1032 0.13291

1020 ->1032 0.10844

1020 ->1033 0.11665

1021 ->1033 0.13003

1021 ->1037 0.19009

1022 ->1036 -0.14738

1024 ->1037 -0.12159

1028 ->1041 0.30966

Excited State 99: Singlet-A 3.8260 eV 324.06 nm f=0.0011

<S**2>=0.000

1017 ->1031 -0.18465

1019 ->1031 0.15293

1019 ->1032 0.21013

1019 ->1033 -0.20241

1019 ->1036 0.13777

1020 ->1032 -0.11855

1020 ->1033 0.16380

1021 ->1030 -0.11523

1021 ->1032 0.13530

1021 ->1034 -0.13430

1021 ->1035 -0.15932

1023 ->1039 -0.12986

1027 ->1041 -0.10583

1028 ->1041 -0.16212

Excited State 100: Singlet-A 3.8279 eV 323.90 nm f=0.0026

<S**2>=0.000

1019 ->1033 0.10318

1020 ->1032 -0.14592

1020 ->1035 0.19522

1020 ->1037 0.13339

1021 ->1035 -0.22297

1021 ->1037 -0.16274

1022 ->1037 0.13537

1026 ->1040 -0.10536

1027 ->1041 0.12476

1028 ->1041 0.30668

Excited State 101: Singlet-A 3.8331 eV 323.46 nm f=0.0071

<S**2>=0.000

1018 ->1031 0.15570

1018 ->1033 -0.11537

1019 ->1033 0.11539

1019 ->1035 0.17796

1020 ->1033 0.11735

1020 ->1034 -0.10128

1020 ->1035 0.16802

1020 ->1036 0.14967

1021 ->1031 0.14145

1021 ->1033 0.10905

1021 ->1037 0.14785

1022 ->1036 -0.10726

1025 ->1040 0.21391

1027 ->1041 -0.15446

Excited State 102: Singlet-A 3.8367 eV 323.15 nm f=0.0107

<S**2>=0.000

1016 ->1030 0.12672

1019 ->1032 0.17406

1019 ->1036 0.19961

1019 ->1037 -0.11155

1020 ->1030 0.10184

1020 ->1035 0.22269

1020 ->1036 -0.15328

1021 ->1036 0.15790

1022 ->1036 0.13562

1022 ->1037 -0.12195

1023 ->1039 0.11888

1025 ->1040 0.26183

1027 ->1041 0.10734

Excited State 103: Singlet-A 3.8434 eV 322.59 nm f=0.0089

<S**2>=0.000

1017 ->1032 0.12235

1017 ->1033 0.11442

1018 ->1032 -0.27486

1018 ->1035 -0.11165

1019 ->1032 -0.13572

1019 ->1035 -0.18288

1020 ->1033 -0.10812

1021 ->1034 0.10388

1021 ->1037 -0.17188

1025 ->1040 0.30394

1028 ->1041 -0.14913

Excited State 104: Singlet-A 3.8513 eV 321.93 nm f=0.0035

<S**2>=0.000

1016 ->1031 -0.10342

1018 ->1032 0.17302

1019 ->1033 -0.10928

1019 ->1035 0.12061

1020 ->1035 -0.12536

1021 ->1031 -0.14858

1021 ->1032 -0.16688

1021 ->1034 -0.11295

1021 ->1035 -0.15026

1021 ->1036 -0.14394

1025 ->1040 0.37824

Excited State 105: Singlet-A 3.8534 eV 321.75 nm f=0.0094

<S**2>=0.000

1016 ->1030 0.14164

1017 ->1030 0.34833

1018 ->1032 0.10253

1019 ->1031 0.10673

1019 ->1032 0.10838

1020 ->1035 -0.12581

1021 ->1031 0.17943

1021 ->1032 0.18424

1021 ->1037 -0.17947

1025 ->1040 0.11264

1028 ->1041 0.12953

Excited State 106: Singlet-A 3.8591 eV 321.28 nm f=0.0136

<S**2>=0.000

1016 ->1030 -0.12428

1017 ->1030 0.28896

1018 ->1030 0.10613

1018 ->1032 -0.16535

1019 ->1034 -0.17415

1020 ->1031 -0.10498

1020 ->1032 -0.10253

1020 ->1033 0.15814

1020 ->1037 -0.19723

1021 ->1036 -0.10210

1022 ->1038 -0.10473

1023 ->1039 0.19364

Excited State 107: Singlet-A 3.8600 eV 321.20 nm f=0.0029

<S**2>=0.000

1017 ->1030 -0.14080

1019 ->1034 0.10462

1019 ->1036 0.12611

1021 ->1030 -0.11115

1021 ->1035 -0.11596

1022 ->1039 0.15941

1023 ->1039 0.44465

1028 ->1042 -0.11890

Excited State 108: Singlet-A 3.8663 eV 320.68 nm f=0.0055

<S**2>=0.000

1016 ->1030 0.25293

1016 ->1031 -0.12893

1018 ->1032 -0.16613

1019 ->1030 0.11724

1020 ->1030 0.12055

1020 ->1034 0.26137

1020 ->1035 0.15892

1021 ->1035 -0.14022

1021 ->1036 -0.14858

1022 ->1038 0.10796

1025 ->1040 -0.14650

Excited State 109: Singlet-A 3.8724 eV 320.18 nm f=0.0027

<S**2>=0.000

1019 ->1036 0.10600

1020 ->1034 -0.10166

1020 ->1037 -0.14447

1021 ->1032 -0.13273

1021 ->1035 -0.11108

1021 ->1038 -0.14320

1022 ->1038 0.40732

1022 ->1039 -0.11947

1023 ->1038 -0.19312

Excited State 110: Singlet-A 3.8755 eV 319.92 nm f=0.0060

<S**2>=0.000

1016 ->1030 -0.18173

1017 ->1030 0.12375

1017 ->1031 0.19609

1019 ->1036 0.24662

1020 ->1031 0.14882

1020 ->1034 0.22431

1020 ->1037 0.19354

1023 ->1039 -0.12286

Excited State 111: Singlet-A 3.8790 eV 319.63 nm f=0.0060

<S**2>=0.000

1016 ->1031 -0.10997

1017 ->1030 0.17215

1017 ->1031 -0.10721

1019 ->1035 -0.10240

1020 ->1037 0.18131

1021 ->1035 0.17525

1021 ->1037 0.11253

1022 ->1038 0.29688

1022 ->1039 0.10230

1023 ->1038 -0.10848

1023 ->1039 0.17249

Excited State 112: Singlet-A 3.8815 eV 319.43 nm f=0.0028

<S**2>=0.000

1014 ->1031 -0.10340

1015 ->1031 0.10051

1016 ->1030 -0.10797

1016 ->1031 0.13196

1019 ->1032 -0.11336

1019 ->1037 -0.11502

1019 ->1038 0.17288

1020 ->1031 0.13387

1020 ->1032 0.15186

1020 ->1038 -0.13966

1021 ->1035 -0.11058

1021 ->1037 -0.12284

1028 ->1041 -0.12387

1028 ->1042 0.18713

Excited State 113: Singlet-A 3.8904 eV 318.69 nm f=0.0055

<S**2>=0.000

1018 ->1030 0.10029

1018 ->1033 0.15288

1018 ->1036 0.18102

1019 ->1031 0.15908

1019 ->1033 0.13174

1019 ->1035 -0.13501

1020 ->1032 -0.13353

1020 ->1036 0.10131

1021 ->1036 -0.17571

1021 ->1038 0.10421

1022 ->1036 -0.13193

1026 ->1041 -0.13697

1028 ->1042 0.12285

Excited State 114: Singlet-A 3.8943 eV 318.37 nm f=0.0021

<S**2>=0.000

1016 ->1030 -0.23771

1016 ->1032 0.12192

1019 ->1032 0.16213

1019 ->1034 0.19177

1019 ->1035 0.11857

1020 ->1035 0.15207

1021 ->1036 -0.10477

1026 ->1041 0.33567

1027 ->1042 0.11140

1028 ->1042 -0.11872

Excited State 115: Singlet-A 3.8962 eV 318.22 nm f=0.0018

<S**2>=0.000

1019 ->1034 0.16219

1020 ->1032 -0.17269

1020 ->1033 0.15790

1021 ->1032 -0.17310

1021 ->1035 -0.11772

1021 ->1037 0.13289

1026 ->1041 0.32414

1027 ->1042 0.16010

1028 ->1042 0.14452

Excited State 116: Singlet-A 3.8997 eV 317.93 nm f=0.0104

<S**2>=0.000

1015 ->1030 0.15016

1016 ->1031 -0.11257

1017 ->1030 0.12802

1018 ->1036 0.10743

1019 ->1031 -0.12988

1019 ->1032 -0.12242

1019 ->1036 0.18803

1019 ->1037 -0.12691

1019 ->1038 0.17548

1020 ->1034 -0.17729

1020 ->1035 -0.11951

1026 ->1041 0.16652

Excited State 117: Singlet-A 3.9035 eV 317.62 nm f=0.0009

<S**2>=0.000

1014 ->1030 0.11702

1018 ->1031 -0.11330

1018 ->1036 0.16910

1019 ->1033 -0.16534

1019 ->1034 -0.11422

1020 ->1032 0.14171

1020 ->1033 -0.10998

1020 ->1035 0.10945

1020 ->1038 0.17172

1021 ->1036 -0.12702

1021 ->1037 0.12438

1022 ->1038 -0.13570

1026 ->1041 0.13020

Excited State 118: Singlet-A 3.9090 eV 317.18 nm f=0.0187

<S**2>=0.000

948 ->1031 -0.10813

948 ->1032 -0.10187

1015 ->1030 -0.14274

1016 ->1031 0.11962

1016 ->1033 0.11789

1018 ->1033 -0.16396

1019 ->1034 0.18466

1019 ->1036 0.10872

1019 ->1038 0.10777

1020 ->1034 -0.14572

1020 ->1036 -0.10766

1026 ->1041 -0.11732

1028 ->1042 -0.13553

Excited State 119: Singlet-A 3.9108 eV 317.03 nm f=0.0021

<S**2>=0.000

1016 ->1032 0.10236

1016 ->1033 0.11443

1018 ->1033 0.12653

1020 ->1037 -0.11229

1028 ->1042 0.39584

Excited State 120: Singlet-A 3.9148 eV 316.71 nm f=0.0027

<S**2>=0.000

950 ->1032 -0.10158

950 ->1033 0.13355

950 ->1034 0.14960

1018 ->1032 0.21260

1019 ->1034 -0.19459

1020 ->1033 0.10158

1020 ->1034 0.16376

1021 ->1035 0.10351

1027 ->1042 0.14492

1028 ->1042 -0.19856

Excited State 121: Singlet-A 3.9169 eV 316.54 nm f=0.0078

<S**2>=0.000

1015 ->1030 -0.15339

1019 ->1034 -0.10464

1021 ->1032 0.11435

1027 ->1042 0.46202

Excited State 122: Singlet-A 3.9230 eV 316.05 nm f=0.0041

<S**2>=0.000

950 ->1033 0.11758

950 ->1034 0.12153

953 ->1035 0.12691

954 ->1034 -0.11257

960 ->1039 0.13831

1016 ->1035 -0.10587

1019 ->1032 0.13409

1020 ->1035 -0.12162

1021 ->1035 -0.11781

1021 ->1036 0.12414

1021 ->1037 -0.10366

Excited State 123: Singlet-A 3.9240 eV 315.97 nm f=0.0057

<S**2>=0.000

949 ->1030 -0.16788

949 ->1032 -0.12090

949 ->1033 0.10245

949 ->1036 0.10103

953 ->1035 0.11344

1017 ->1033 0.10216

1020 ->1034 -0.10377

1020 ->1035 -0.14268

1025 ->1041 0.13949

1027 ->1042 0.28491

Excited State 124: Singlet-A 3.9247 eV 315.90 nm f=0.0113

<S**2>=0.000

949 ->1030 -0.15808

949 ->1032 -0.10418

953 ->1035 -0.12911

1020 ->1035 0.17213

1020 ->1036 0.15821

1021 ->1036 0.26252

1025 ->1041 0.11132

Excited State 125: Singlet-A 3.9269 eV 315.73 nm f=0.0002

<S**2>=0.000

951 ->1036 -0.10049

953 ->1035 0.10554

958 ->1039 0.10459

960 ->1039 -0.16145

1015 ->1030 -0.10005

1020 ->1036 0.13415

1021 ->1035 -0.10565

1021 ->1036 0.26951

1028 ->1042 -0.15656

Excited State 126: Singlet-A 3.9273 eV 315.70 nm f=0.0004

<S**2>=0.000

958 ->1039 0.13243

960 ->1039 -0.21273

1018 ->1032 -0.12519

1018 ->1033 -0.11176

1019 ->1032 0.13933

1019 ->1035 0.11654

1020 ->1033 -0.12738

1026 ->1041 -0.12007

1027 ->1042 0.16929

1028 ->1042 0.22628

Excited State 127: Singlet-A 3.9331 eV 315.23 nm f=0.0046

<S**2>=0.000

949 ->1030 0.22106

949 ->1032 0.14535

949 ->1033 -0.12437

949 ->1035 0.10865

949 ->1036 -0.11319

951 ->1036 0.16908

952 ->1036 0.14119

1017 ->1036 -0.10397

1018 ->1033 0.11927

1018 ->1036 0.11577

1019 ->1038 0.12551

1020 ->1036 0.14943

1025 ->1041 0.15394

Excited State 128: Singlet-A 3.9361 eV 314.99 nm f=0.0032

<S**2>=0.000

948 ->1031 0.10855

948 ->1032 0.10953

955 ->1037 -0.13027

956 ->1038 -0.11016

959 ->1038 0.12007

1016 ->1030 -0.13354

1017 ->1031 -0.12000

1019 ->1034 0.13413

1019 ->1038 0.16213

1020 ->1037 0.10189

1021 ->1036 -0.14352

Excited State 129: Singlet-A 3.9381 eV 314.84 nm f=0.0033

<S**2>=0.000

954 ->1037 -0.15336

955 ->1033 -0.12102

955 ->1034 0.12882

955 ->1037 -0.16955

956 ->1033 -0.14977

956 ->1034 0.12536

956 ->1037 -0.17988

956 ->1038 -0.10946

957 ->1037 -0.10827

1016 ->1030 0.16150

1018 ->1033 -0.12529

1020 ->1037 0.10212

Excited State 130: Singlet-A 3.9398 eV 314.70 nm f=0.0048

<S**2>=0.000

950 ->1033 0.10782

950 ->1034 0.12146

956 ->1037 -0.10832

1019 ->1038 -0.10700

1020 ->1034 -0.11647

1025 ->1041 0.38984

Excited State 131: Singlet-A 3.9417 eV 314.55 nm f=0.0050

<S**2>=0.000

951 ->1036 -0.14296

952 ->1036 -0.14532

954 ->1034 -0.13712

954 ->1037 -0.11773

1017 ->1033 0.15158

1018 ->1036 -0.14204

1019 ->1034 0.18277

1020 ->1033 0.15340

1020 ->1037 -0.10233

1025 ->1041 0.22786

Excited State 132: Singlet-A 3.9461 eV 314.20 nm f=0.0030

<S**2>=0.000

954 ->1034 0.16328

954 ->1037 0.14029

1019 ->1033 0.18947

1019 ->1034 -0.16590

1019 ->1035 0.12594

1020 ->1034 0.13173

1020 ->1035 -0.12629

1025 ->1041 0.20627

1026 ->1041 0.14731

Excited State 133: Singlet-A 3.9482 eV 314.03 nm f=0.0183

<S**2>=0.000

950 ->1034 0.10130

960 ->1039 0.12638

1015 ->1030 0.20860

1016 ->1031 -0.13489

1017 ->1031 -0.20009

1017 ->1032 -0.11896

1018 ->1033 0.17951

1019 ->1031 -0.10455

1019 ->1037 0.11691

1020 ->1034 -0.12349

1026 ->1041 -0.12362

1027 ->1042 0.13827

Excited State 134: Singlet-A 3.9521 eV 313.72 nm f=0.0132

<S**2>=0.000

1017 ->1031 0.13998

1018 ->1032 -0.13194

1018 ->1034 0.16428

1018 ->1035 0.32578

1018 ->1038 -0.13460

1019 ->1035 -0.15381

1019 ->1036 -0.19551

1019 ->1037 -0.13328

1020 ->1038 -0.21686

Excited State 135: Singlet-A 3.9557 eV 313.43 nm f=0.0018

<S**2>=0.000

953 ->1035 -0.13860

1014 ->1030 0.17974

1017 ->1031 0.23957

1018 ->1031 0.10641

1018 ->1033 0.23640

1019 ->1036 -0.10140

1019 ->1038 0.11453

1020 ->1036 -0.15364

1021 ->1038 -0.12244

1021 ->1039 -0.16234

Excited State 136: Singlet-A 3.9602 eV 313.07 nm f=0.0033

<S**2>=0.000

951 ->1035 0.10238

1015 ->1030 0.22449

1015 ->1031 0.11167

1016 ->1031 0.16396

1017 ->1031 0.17612

1017 ->1033 0.11891

1017 ->1034 -0.11285

1018 ->1034 -0.13152

1018 ->1036 -0.15108

1021 ->1036 -0.11518

1021 ->1037 0.10663

1025 ->1041 -0.12006

Excited State 137: Singlet-A 3.9619 eV 312.94 nm f=0.0213

<S**2>=0.000

1014 ->1030 0.27665

1016 ->1031 0.11758

1016 ->1032 -0.10265

1017 ->1033 -0.19311

1018 ->1033 0.11804

1018 ->1034 -0.14541

1018 ->1037 0.15043

1018 ->1038 -0.13417

1020 ->1038 -0.26180

1021 ->1038 0.11598

1025 ->1041 0.12921

Excited State 138: Singlet-A 3.9643 eV 312.75 nm f=0.0078

<S**2>=0.000

1014 ->1030 -0.15885

1015 ->1030 -0.21074

1020 ->1037 0.10800

1024 ->1040 -0.15889

1026 ->1042 0.44727

Excited State 139: Singlet-A 3.9676 eV 312.49 nm f=0.0061

<S**2>=0.000

1017 ->1031 0.15429

1017 ->1032 0.20652

1017 ->1033 0.14839

1017 ->1039 -0.11085

1018 ->1035 -0.17821

1019 ->1039 0.11632

1020 ->1036 0.12141

1020 ->1037 0.14538

1020 ->1038 -0.12482

1020 ->1039 -0.10636

1021 ->1039 0.17416

1026 ->1042 -0.19231

Excited State 140: Singlet-A 3.9704 eV 312.27 nm f=0.0083

<S**2>=0.000

959 ->1038 0.10647

1014 ->1030 0.18743

1015 ->1030 0.14394

1016 ->1031 -0.14545

1016 ->1032 0.11480

1017 ->1031 0.11376

1019 ->1035 -0.13850

1021 ->1038 0.23426

1021 ->1039 0.15454

1026 ->1042 0.13514

Excited State 141: Singlet-A 3.9728 eV 312.09 nm f=0.0076

<S**2>=0.000

1014 ->1030 -0.12827

1016 ->1034 -0.12283

1017 ->1032 -0.10043

1018 ->1033 0.17234

1018 ->1035 -0.12857

1019 ->1033 -0.11954

1019 ->1037 0.10436

1021 ->1037 -0.12179

1021 ->1038 0.33986

1021 ->1039 0.10911

1026 ->1042 -0.12281

Excited State 142: Singlet-A 3.9770 eV 311.75 nm f=0.0038

<S**2>=0.000

1014 ->1030 0.13255

1015 ->1030 0.10525

1018 ->1034 0.26501

1018 ->1035 -0.10023

1018 ->1039 -0.13061

1019 ->1036 -0.14976

1019 ->1038 0.10832

1020 ->1036 -0.17709

1021 ->1038 -0.13152

1021 ->1039 0.30072

1026 ->1042 0.12095

Excited State 143: Singlet-A 3.9816 eV 311.39 nm f=0.0052

<S**2>=0.000

960 ->1039 0.10313

1016 ->1031 0.16984

1016 ->1033 0.11089

1017 ->1032 0.19788

1017 ->1033 0.11401

1018 ->1035 0.11955

1019 ->1035 0.17338

1020 ->1038 0.12467

1021 ->1038 0.33202

1021 ->1039 -0.18026

Excited State 144: Singlet-A 3.9862 eV 311.04 nm f=0.0102

<S**2>=0.000

948 ->1030 -0.10802

948 ->1031 0.14139

948 ->1032 0.12140

1014 ->1031 -0.16985

1016 ->1031 0.20281

1016 ->1032 0.21717

1016 ->1033 0.19266

1017 ->1031 -0.14802

1017 ->1032 -0.18213

1017 ->1034 -0.12291

1021 ->1039 0.14696

Excited State 145: Singlet-A 3.9905 eV 310.70 nm f=0.0086

<S**2>=0.000

1014 ->1031 0.12719

1014 ->1032 -0.11101

1015 ->1031 0.15773

1015 ->1032 -0.13538

1016 ->1037 0.10348

1017 ->1033 -0.11580

1017 ->1035 -0.16566

1018 ->1033 0.12281

1018 ->1034 -0.10223

1018 ->1035 0.13705

1018 ->1036 -0.17216

1020 ->1038 0.16504

1021 ->1039 0.19106

1026 ->1042 0.13838

Excited State 146: Singlet-A 3.9949 eV 310.36 nm f=0.0016

<S**2>=0.000

1016 ->1032 0.10458

1016 ->1033 -0.10545

1016 ->1034 -0.15642

1017 ->1034 0.13296

1018 ->1034 0.24130

1018 ->1036 -0.24059

1019 ->1036 0.17185

1020 ->1036 0.23767

Excited State 147: Singlet-A 4.0025 eV 309.76 nm f=0.0064

<S**2>=0.000

1017 ->1032 -0.14154

1017 ->1034 0.13726

1017 ->1035 -0.11693

1017 ->1039 -0.18380

1019 ->1037 -0.14791

1019 ->1038 -0.12296

1019 ->1039 0.32201

1020 ->1039 -0.10333

1026 ->1042 0.16875

Excited State 148: Singlet-A 4.0049 eV 309.58 nm f=0.0030

<S**2>=0.000

1015 ->1031 -0.18571

1016 ->1031 0.10022

1016 ->1032 -0.14260

1017 ->1032 -0.13706

1017 ->1035 0.12017

1018 ->1033 0.11946

1018 ->1034 -0.10873

1018 ->1035 0.24782

1019 ->1035 -0.12308

1019 ->1036 0.11597

1019 ->1038 0.11503

1020 ->1036 0.15682

1020 ->1038 0.11438

1021 ->1039 0.19957

Excited State 149: Singlet-A 4.0147 eV 308.82 nm f=0.0200

<S**2>=0.000

1014 ->1030 -0.10692

1014 ->1031 -0.12251

1015 ->1031 0.13208

1016 ->1032 0.10935

1016 ->1033 -0.13967

1016 ->1035 -0.18808

1017 ->1032 0.19741

1017 ->1035 0.10400

1017 ->1037 0.10321

1018 ->1034 -0.11001

1018 ->1035 0.20417

1018 ->1036 0.17794

1019 ->1037 0.10618

1020 ->1036 -0.10750

1021 ->1039 0.15785

Excited State 150: Singlet-A 4.0198 eV 308.44 nm f=0.0068

<S**2>=0.000

1013 ->1030 -0.16632

1014 ->1032 -0.12623

1014 ->1034 -0.10264

1015 ->1033 -0.14659

1016 ->1034 0.13172

1017 ->1033 0.20417

1017 ->1034 0.26428

1017 ->1035 -0.18432

1019 ->1034 -0.12744

1019 ->1036 0.11680

1024 ->1040 0.12594

1025 ->1042 -0.11011

1026 ->1042 0.11849

Excited State 151: Singlet-A 4.0202 eV 308.40 nm f=0.0073

<S**2>=0.000

1013 ->1030 0.16721

1014 ->1030 0.13982

1015 ->1030 -0.16225

1016 ->1033 -0.19781

1016 ->1034 0.12645

1017 ->1032 -0.14024

1017 ->1033 0.26934

1017 ->1034 -0.13631

1017 ->1035 -0.10048

1019 ->1037 -0.14910

1020 ->1038 0.14068

1023 ->1040 -0.10300

1024 ->1040 -0.14715

1025 ->1042 -0.20198

Excited State 152: Singlet-A 4.0243 eV 308.09 nm f=0.0059

<S**2>=0.000

1015 ->1033 -0.10324

1016 ->1035 -0.11464

1017 ->1034 0.12473

1017 ->1036 -0.11807

1018 ->1038 -0.12640

1019 ->1037 -0.12002

1024 ->1040 -0.25182

1025 ->1042 0.41375

1026 ->1042 -0.10833

Excited State 153: Singlet-A 4.0256 eV 307.99 nm f=0.0074

<S**2>=0.000

1013 ->1030 0.16626

1014 ->1030 0.11789

1017 ->1032 -0.11980

1017 ->1036 -0.10577

1018 ->1037 -0.14309

1018 ->1038 0.10587

1019 ->1038 -0.11218

1020 ->1038 -0.13808

1020 ->1039 -0.10963

1024 ->1040 0.29370

1025 ->1041 -0.11297

1025 ->1042 0.27130

1026 ->1042 0.10337

Excited State 154: Singlet-A 4.0287 eV 307.75 nm f=0.0018

<S**2>=0.000

1014 ->1032 -0.11719

1016 ->1034 -0.15618

1016 ->1035 0.16972

1017 ->1035 -0.14413

1018 ->1037 -0.20760

1018 ->1038 0.14614

1020 ->1039 0.29709

1021 ->1039 0.12445

1023 ->1040 -0.11174

1024 ->1040 -0.23612

Excited State 155: Singlet-A 4.0353 eV 307.25 nm f=0.0209

<S**2>=0.000

1017 ->1034 -0.16110

1017 ->1039 -0.10565

1018 ->1037 0.16340

1018 ->1038 -0.11650

1018 ->1039 -0.10065

1019 ->1038 0.11497

1019 ->1039 0.26070

1020 ->1039 0.34942

1022 ->1039 -0.12227

1023 ->1040 -0.10719

1024 ->1040 0.17103

Excited State 156: Singlet-A 4.0377 eV 307.07 nm f=0.0078

<S**2>=0.000

1012 ->1030 -0.15103

1013 ->1031 -0.10226

1014 ->1033 0.12583

1015 ->1034 -0.13815

1016 ->1030 -0.11670

1016 ->1032 -0.18703

1016 ->1033 0.26162

1016 ->1035 -0.12037

1017 ->1037 0.20893

1018 ->1034 0.17508

1025 ->1042 -0.21585

Excited State 157: Singlet-A 4.0392 eV 306.95 nm f=0.0078

<S**2>=0.000

1015 ->1031 -0.13610

1016 ->1034 0.19945

1017 ->1032 0.10320

1017 ->1035 0.13458

1018 ->1034 0.16304

1018 ->1036 -0.11838

1018 ->1037 -0.12277

1019 ->1037 -0.17767

1019 ->1038 -0.12945

1020 ->1039 0.28405

1024 ->1040 0.16026

Excited State 158: Singlet-A 4.0429 eV 306.67 nm f=0.0057

<S**2>=0.000

1013 ->1030 0.11309

1014 ->1036 0.10812

1016 ->1034 -0.23033

1016 ->1036 0.10095

1016 ->1037 0.13072

1017 ->1034 0.12331

1017 ->1036 0.13224

1018 ->1034 -0.13731

1020 ->1039 0.19007

1023 ->1040 0.36442

Excited State 159: Singlet-A 4.0455 eV 306.47 nm f=0.0063

<S**2>=0.000

1013 ->1031 0.11292

1014 ->1030 -0.12915

1014 ->1031 0.12661

1014 ->1036 0.13138

1015 ->1036 -0.11376

1016 ->1034 0.10766

1016 ->1035 -0.15221

1017 ->1035 -0.11660

1017 ->1036 0.26797

1017 ->1038 0.10427

1018 ->1034 0.12190

1018 ->1038 0.16526

1019 ->1037 0.12688

1020 ->1038 -0.13767

1022 ->1040 -0.10398

1023 ->1040 -0.12015

1025 ->1042 0.15776

Excited State 160: Singlet-A 4.0509 eV 306.06 nm f=0.0079

<S**2>=0.000

1010 ->1030 -0.13381

1014 ->1030 0.15162

1015 ->1033 -0.15017

1016 ->1030 -0.10829

1016 ->1031 -0.13225

1016 ->1032 -0.16621

1016 ->1033 0.13547

1016 ->1034 -0.16461

1016 ->1035 -0.12524

1017 ->1033 0.12115

1017 ->1037 -0.14852

1018 ->1039 0.17200

1023 ->1040 -0.15018

1023 ->1041 0.11053

1024 ->1040 0.11555

1024 ->1041 0.10069

Excited State 161: Singlet-A 4.0556 eV 305.71 nm f=0.0032

<S**2>=0.000

1014 ->1034 0.10181

1015 ->1031 0.12100

1015 ->1033 -0.15392

1016 ->1034 0.14834

1017 ->1033 0.11562

1017 ->1034 -0.13812

1017 ->1039 -0.10624

1019 ->1037 0.22974

1019 ->1039 0.16647

1020 ->1038 -0.13589

1023 ->1040 0.29796

1024 ->1040 -0.11852

Excited State 162: Singlet-A 4.0582 eV 305.51 nm f=0.0155

<S**2>=0.000

1016 ->1032 0.10140

1016 ->1034 0.11858

1017 ->1039 -0.11049

1018 ->1035 -0.13319

1018 ->1038 -0.20725

1018 ->1039 0.34607

1024 ->1041 0.22215

1029 ->1043 0.15700

Excited State 163: Singlet-A 4.0613 eV 305.28 nm f=0.0088

<S**2>=0.000

1014 ->1031 -0.13044

1014 ->1038 0.10046

1016 ->1036 -0.10192

1017 ->1034 -0.13215

1017 ->1035 -0.10358

1017 ->1036 0.22128

1017 ->1037 0.11380

1017 ->1038 -0.10154

1018 ->1038 -0.17369

1018 ->1039 0.15117

1019 ->1038 0.19570

1019 ->1039 -0.11308

1020 ->1038 0.12708

1023 ->1040 0.10174

1025 ->1042 0.11575

1029 ->1043 0.17017

Excited State 164: Singlet-A 4.0646 eV 305.04 nm f=0.0060

<S**2>=0.000

1018 ->1039 -0.13217

1023 ->1040 -0.11402

1029 ->1043 0.58027

1029 ->1047 -0.12872

Excited State 165: Singlet-A 4.0691 eV 304.70 nm f=0.0062

<S**2>=0.000

1013 ->1030 -0.20808

1014 ->1033 0.16445

1014 ->1037 0.13074

1015 ->1033 0.13021

1015 ->1037 0.17904

1016 ->1033 -0.10212

1016 ->1037 -0.21497

1017 ->1037 -0.10027

1018 ->1038 0.13662

1019 ->1037 -0.11993

1019 ->1038 -0.14186

1023 ->1040 0.24538

1029 ->1043 0.10030

Excited State 166: Singlet-A 4.0733 eV 304.38 nm f=0.0309

<S**2>=0.000

1015 ->1031 0.10110

1017 ->1036 0.18204

1018 ->1039 -0.23371

1024 ->1041 0.46266

Excited State 167: Singlet-A 4.0789 eV 303.96 nm f=0.0275

<S**2>=0.000

1013 ->1031 -0.12007

1015 ->1031 0.25927

1015 ->1032 0.19534

1015 ->1034 0.16449

1017 ->1036 0.21671

1018 ->1039 0.22613

1019 ->1037 -0.10218

1022 ->1040 -0.12706

1023 ->1041 0.11598

1024 ->1041 -0.19012

Excited State 168: Singlet-A 4.0845 eV 303.55 nm f=0.0050

<S**2>=0.000

1014 ->1031 0.10200

1015 ->1031 0.12332

1017 ->1037 -0.16979

1018 ->1037 -0.15424

1022 ->1040 0.36650

1023 ->1041 -0.16552

1024 ->1041 -0.17274

1029 ->1044 0.13875

Excited State 169: Singlet-A 4.0899 eV 303.15 nm f=0.0428

<S**2>=0.000

1013 ->1030 -0.12091

1014 ->1032 0.13034

1015 ->1031 -0.19485

1015 ->1032 0.17050

1015 ->1034 0.18973

1015 ->1037 0.12359

1016 ->1033 -0.11459

1016 ->1035 -0.13220

1016 ->1037 0.16427

1017 ->1035 -0.15136

1017 ->1037 0.19745

1018 ->1037 -0.16096

Excited State 170: Singlet-A 4.0922 eV 302.98 nm f=0.0043

<S**2>=0.000

1010 ->1030 -0.11352

1012 ->1030 -0.15805

1014 ->1031 0.28724

1014 ->1032 0.21726

1015 ->1031 -0.13564

1016 ->1031 0.15635

1016 ->1032 0.19382

1016 ->1037 -0.13416

1017 ->1036 -0.17188

1017 ->1038 -0.12737

Excited State 171: Singlet-A 4.0970 eV 302.62 nm f=0.0029

<S**2>=0.000

1013 ->1030 0.22537

1015 ->1034 -0.11510

1016 ->1036 -0.14784

1017 ->1035 -0.11779

1023 ->1041 0.21801

1029 ->1044 0.37739

Excited State 172: Singlet-A 4.0989 eV 302.48 nm f=0.0076

<S**2>=0.000

1015 ->1031 -0.10482

1015 ->1032 -0.19467

1016 ->1036 0.10633

1017 ->1039 -0.11296

1018 ->1037 0.25765

1018 ->1038 0.16869

1020 ->1037 0.10025

1023 ->1041 -0.25492

1029 ->1043 -0.10124

1029 ->1044 0.16020

Excited State 173: Singlet-A 4.0997 eV 302.42 nm f=0.0065

<S**2>=0.000

1013 ->1030 -0.17106

1014 ->1031 0.11932

1015 ->1030 -0.10761

1017 ->1035 0.18183

1017 ->1037 0.13980

1017 ->1039 0.12531

1023 ->1041 0.10025

1029 ->1044 0.39224

Excited State 174: Singlet-A 4.1083 eV 301.79 nm f=0.0358

<S**2>=0.000

1010 ->1030 0.14304

1012 ->1030 0.22817

1014 ->1031 0.18731

1015 ->1032 0.15166

1015 ->1033 -0.16125

1016 ->1034 -0.11129

1016 ->1036 -0.20442

1017 ->1037 0.14947

1017 ->1039 0.11623

1022 ->1040 0.13996

1029 ->1044 -0.11722

Excited State 175: Singlet-A 4.1130 eV 301.45 nm f=0.0043

<S**2>=0.000

1010 ->1030 0.13598

1014 ->1032 -0.13592

1014 ->1035 -0.14392

1015 ->1031 -0.12500

1015 ->1032 -0.20723

1015 ->1039 -0.10875

1016 ->1037 -0.23130

1016 ->1039 -0.12480

1018 ->1037 -0.13499

1023 ->1041 0.28320

Excited State 176: Singlet-A 4.1153 eV 301.27 nm f=0.0182

<S**2>=0.000

1013 ->1030 -0.10228

1015 ->1034 -0.12773

1016 ->1037 0.15251

1017 ->1035 0.12543

1018 ->1037 0.18628

1018 ->1038 0.19026

1022 ->1040 0.30669

1023 ->1041 0.23868

1029 ->1044 -0.17530

Excited State 177: Singlet-A 4.1198 eV 300.95 nm f=0.0027

<S**2>=0.000

1012 ->1030 0.13497

1015 ->1033 0.20865

1015 ->1034 0.16020

1016 ->1032 -0.13936

1016 ->1033 0.12227

1016 ->1034 0.18211

1016 ->1035 -0.14533

1016 ->1036 0.14304

1017 ->1035 -0.13073

1017 ->1038 -0.12637

1017 ->1039 0.13175

1018 ->1037 0.10387

1022 ->1040 0.17478

1023 ->1041 0.16709

Excited State 178: Singlet-A 4.1251 eV 300.56 nm f=0.0045

<S**2>=0.000

1010 ->1030 0.18208

1011 ->1030 0.20305

1012 ->1030 -0.13873

1014 ->1032 -0.13627

1014 ->1033 0.16220

1014 ->1037 -0.10416

1015 ->1034 0.13525

1015 ->1035 0.10390

1015 ->1037 -0.15703

1016 ->1037 0.14007

1017 ->1034 -0.15748

1018 ->1037 0.16253

1018 ->1038 0.10833

Excited State 179: Singlet-A 4.1301 eV 300.19 nm f=0.0063

<S**2>=0.000

1022 ->1040 -0.13234

1029 ->1043 -0.15470

1029 ->1044 -0.20656

1029 ->1045 0.26840

1029 ->1046 0.40084

1029 ->1047 -0.19101

1029 ->1049 -0.12211

Excited State 180: Singlet-A 4.1326 eV 300.01 nm f=0.0095

<S**2>=0.000

1012 ->1030 0.12011

1014 ->1037 0.10859

1014 ->1038 0.14445

1015 ->1034 -0.10383

1015 ->1035 -0.13221

1015 ->1037 0.16101

1016 ->1034 -0.10829

1016 ->1037 0.25023

1016 ->1038 0.17071

1017 ->1037 -0.24326

1017 ->1038 -0.19731

1022 ->1040 -0.11694

Excited State 181: Singlet-A 4.1339 eV 299.92 nm f=0.0073

<S**2>=0.000

1014 ->1032 -0.23856

1014 ->1036 0.14190

1015 ->1032 0.25258

1015 ->1035 0.10625

1015 ->1036 -0.14301

1016 ->1035 -0.18532

1016 ->1036 0.19886

1017 ->1036 -0.11621

1029 ->1046 -0.17714

Excited State 182: Singlet-A 4.1385 eV 299.58 nm f=0.0033

<S**2>=0.000

1011 ->1031 0.19941

1012 ->1031 -0.11656

1013 ->1030 -0.10860

1013 ->1031 0.24072

1014 ->1031 0.15561

1014 ->1032 -0.11033

1014 ->1033 -0.15520

1014 ->1034 0.10432

1015 ->1036 0.16504

1015 ->1037 0.11673

1016 ->1036 -0.10867

1017 ->1035 0.13018

1017 ->1037 0.12977

1017 ->1039 -0.12559

1019 ->1039 -0.12922

Excited State 183: Singlet-A 4.1445 eV 299.15 nm f=0.0041

<S**2>=0.000

1010 ->1030 0.12002

1011 ->1030 0.12529

1014 ->1033 -0.14978

1014 ->1034 -0.12983

1014 ->1036 0.10201

1015 ->1033 -0.10870

1015 ->1036 0.29671

1016 ->1036 0.30849

1017 ->1036 0.15629

1017 ->1039 0.10987

Excited State 184: Singlet-A 4.1469 eV 298.98 nm f=0.0035

<S**2>=0.000

1029 ->1045 0.54742

1029 ->1046 -0.32993

Excited State 185: Singlet-A 4.1492 eV 298.81 nm f=0.0065

<S**2>=0.000

1011 ->1030 -0.12088

1012 ->1030 0.25390

1014 ->1032 0.19022

1015 ->1033 0.10148

1015 ->1035 0.19846

1015 ->1037 -0.17295

1015 ->1039 -0.11010

1016 ->1039 -0.11204

1017 ->1039 -0.13568

1022 ->1041 -0.14766

1029 ->1045 -0.12936

1029 ->1046 0.12449

Excited State 186: Singlet-A 4.1559 eV 298.33 nm f=0.0051

<S**2>=0.000

1013 ->1031 0.26130

1013 ->1032 -0.12016

1014 ->1032 0.22253

1015 ->1033 -0.12871

1015 ->1036 -0.18093

1016 ->1035 0.23964

1016 ->1036 0.18896

1017 ->1037 0.12026

1024 ->1042 -0.11388

Excited State 187: Singlet-A 4.1606 eV 298.00 nm f=0.0144

<S**2>=0.000

1012 ->1030 0.18123

1014 ->1033 -0.13817

1014 ->1035 0.21277

1015 ->1035 0.24670

1016 ->1035 -0.11354

1017 ->1035 0.13074

1022 ->1041 0.28996

Excited State 188: Singlet-A 4.1645 eV 297.72 nm f=0.0146

<S**2>=0.000

1011 ->1030 -0.15853

1012 ->1031 0.13540

1013 ->1032 -0.14703

1014 ->1033 0.24143

1014 ->1034 0.21678

1014 ->1036 -0.13986

1015 ->1032 0.10455

1015 ->1033 -0.10299

1015 ->1034 -0.14279

1015 ->1036 0.22656

1016 ->1034 -0.12548

1016 ->1036 0.13248

1022 ->1041 0.11705

Excited State 189: Singlet-A 4.1720 eV 297.18 nm f=0.0179

<S**2>=0.000

1014 ->1033 -0.12301

1014 ->1035 -0.19405

1014 ->1036 0.13202

1014 ->1037 -0.10983

1015 ->1033 0.13310

1015 ->1035 -0.13064

1015 ->1037 -0.18724

1015 ->1038 -0.10421

1016 ->1036 -0.11895

1022 ->1041 0.33072

1023 ->1042 -0.11700

1029 ->1047 0.14567

Excited State 190: Singlet-A 4.1742 eV 297.02 nm f=0.0114

<S**2>=0.000

1009 ->1030 0.10588

1012 ->1031 0.20366

1012 ->1032 -0.16520

1012 ->1033 -0.14530

1013 ->1035 0.19764

1014 ->1034 0.10001

1014 ->1036 0.13971

1016 ->1036 -0.10696

1016 ->1038 0.13492

1022 ->1041 -0.10673

1029 ->1046 -0.14977

1029 ->1047 -0.17418

Excited State 191: Singlet-A 4.1807 eV 296.57 nm f=0.0028

<S**2>=0.000

1013 ->1035 -0.13405

1014 ->1033 0.10242

1015 ->1034 0.11778

1015 ->1035 0.11615

1016 ->1038 0.23355

1017 ->1038 0.16473

1022 ->1041 0.19930

1024 ->1042 -0.10772

1029 ->1046 -0.16142

1029 ->1047 -0.22756

Excited State 192: Singlet-A 4.1840 eV 296.33 nm f=0.0164

<S**2>=0.000

1009 ->1030 0.14534

1015 ->1035 0.13000

1016 ->1038 0.22228

1017 ->1038 0.13971

1029 ->1045 0.11131

1029 ->1046 0.21678

1029 ->1047 0.35981

1029 ->1049 0.12396

1029 ->1051 0.11611

Excited State 193: Singlet-A 4.1859 eV 296.19 nm f=0.0063

<S**2>=0.000

1009 ->1030 0.12342

1012 ->1031 -0.15052

1013 ->1031 -0.10427

1014 ->1034 0.21408

1014 ->1036 0.13637

1015 ->1035 0.16961

1016 ->1035 0.10139

1016 ->1038 -0.20728

1020 ->1040 -0.10199

1022 ->1041 0.10449

1024 ->1042 0.29082

Excited State 194: Singlet-A 4.1902 eV 295.89 nm f=0.0011

<S**2>=0.000

1011 ->1030 0.16946

1011 ->1032 0.11535

1012 ->1031 -0.12052

1013 ->1030 -0.12259

1013 ->1032 0.22342

1013 ->1033 0.14339

1014 ->1034 0.28001

1014 ->1036 -0.21400

1015 ->1033 0.11013

1015 ->1034 -0.11165

1016 ->1036 0.10811

1024 ->1042 -0.20288

Excited State 195: Singlet-A 4.1944 eV 295.60 nm f=0.0060

<S**2>=0.000

1013 ->1031 0.16073

1014 ->1035 0.10196

1015 ->1035 -0.17459

1016 ->1037 -0.13107

1016 ->1039 -0.17928

1017 ->1039 0.10017

1019 ->1039 0.11541

1021 ->1040 -0.18888

1022 ->1041 0.14924

1024 ->1042 0.36683

Excited State 196: Singlet-A 4.1978 eV 295.36 nm f=0.0026

<S**2>=0.000

1010 ->1030 -0.16307

1011 ->1030 0.10858

1011 ->1031 0.12011

1011 ->1036 0.12333

1012 ->1032 -0.12105

1012 ->1035 -0.10474

1013 ->1033 0.20779

1014 ->1033 0.17196

1014 ->1034 -0.11214

1014 ->1036 0.18040

1015 ->1033 -0.10411

1015 ->1035 0.15457

1016 ->1039 0.10959

1021 ->1040 -0.19628

Excited State 197: Singlet-A 4.2022 eV 295.05 nm f=0.0061

<S**2>=0.000

1010 ->1030 0.12224

1011 ->1030 0.13184

1014 ->1036 -0.12325

1014 ->1037 0.11864

1014 ->1039 -0.12431

1015 ->1039 0.14496

1016 ->1038 0.14170

1016 ->1039 0.30079

1017 ->1039 -0.17846

1019 ->1039 -0.16948

1024 ->1042 0.19298

Excited State 198: Singlet-A 4.2063 eV 294.76 nm f=0.0037

<S**2>=0.000

1011 ->1032 0.10095

1012 ->1032 -0.11431

1013 ->1034 0.12842

1014 ->1035 0.24985

1015 ->1035 -0.15598

1016 ->1039 0.12528

1021 ->1040 0.38087

1024 ->1042 0.10671

Excited State 199: Singlet-A 4.2112 eV 294.42 nm f=0.0104

<S**2>=0.000

1009 ->1030 -0.16639

1010 ->1031 0.10582

1011 ->1030 0.13560

1011 ->1031 0.10345

1012 ->1034 -0.13929

1013 ->1033 -0.15477

1013 ->1034 -0.10779

1013 ->1036 0.12216

1015 ->1036 -0.10887

1016 ->1039 -0.13990

1021 ->1040 0.10923

1022 ->1042 -0.11358

1023 ->1042 -0.12713

1026 ->1043 0.14436

1028 ->1043 0.20728

Excited State 200: Singlet-A 4.2146 eV 294.18 nm f=0.0075

<S**2>=0.000

1009 ->1030 0.13483

1011 ->1030 -0.12033

1016 ->1038 -0.10681

1023 ->1042 -0.15044

1025 ->1043 0.14387

1026 ->1043 0.22165

1028 ->1043 0.33898

1028 ->1046 0.11488

1029 ->1049 0.10938

Excited State 201: Singlet-A 4.2164 eV 294.05 nm f=0.0028

<S**2>=0.000

1013 ->1033 -0.12197

1014 ->1034 0.11768

1014 ->1035 0.27416

1014 ->1036 0.24414

1015 ->1035 -0.10204

1021 ->1040 -0.16269

1022 ->1042 0.12789

1024 ->1042 -0.18188

1027 ->1044 -0.12594

1027 ->1048 -0.10685

1029 ->1048 -0.10161

Excited State 202: Singlet-A 4.2179 eV 293.95 nm f=0.0015

<S**2>=0.000

1009 ->1030 0.18616

1010 ->1030 -0.17817

1011 ->1030 0.15488

1012 ->1033 0.21025

1013 ->1032 -0.19920

1015 ->1033 0.11056

1015 ->1034 -0.18400

1015 ->1035 -0.13977

1023 ->1042 0.20412

1028 ->1043 0.12171

Excited State 203: Singlet-A 4.2212 eV 293.72 nm f=0.0020

<S**2>=0.000

1009 ->1030 -0.10536

1014 ->1035 -0.10443

1021 ->1040 0.27359

1022 ->1042 0.11920

1023 ->1042 0.25775

1027 ->1044 -0.20890

1027 ->1048 -0.13372

1027 ->1054 -0.10686

Excited State 204: Singlet-A 4.2250 eV 293.46 nm f=0.0035

<S**2>=0.000

1010 ->1030 0.12582

1011 ->1030 -0.14600

1012 ->1033 -0.10783

1023 ->1042 0.24388

1026 ->1043 0.12638

1027 ->1044 0.11651

1027 ->1048 0.12981

1028 ->1043 0.15285

1029 ->1048 0.26902

1029 ->1049 -0.17506

Excited State 205: Singlet-A 4.2303 eV 293.09 nm f=0.0114

<S**2>=0.000

1016 ->1039 -0.10045

1020 ->1040 0.18794

1026 ->1046 -0.14647

1028 ->1046 0.32111

1028 ->1049 -0.12868

1029 ->1048 0.14208

1029 ->1049 -0.10324

Excited State 206: Singlet-A 4.2352 eV 292.75 nm f=0.0058

<S**2>=0.000

1020 ->1040 0.18490

1021 ->1041 -0.13783

1023 ->1042 -0.26562

1027 ->1044 -0.18871

1029 ->1048 0.30060

1029 ->1053 0.16499

1029 ->1054 0.10542

Excited State 207: Singlet-A 4.2361 eV 292.69 nm f=0.0068

<S**2>=0.000

1010 ->1031 0.15587

1011 ->1036 0.11669

1012 ->1031 0.10536

1012 ->1032 0.16764

1012 ->1033 0.10051

1012 ->1034 0.19083

1013 ->1030 -0.12867

1013 ->1032 -0.10995

1013 ->1033 0.18898

1013 ->1034 0.15596

1014 ->1033 -0.11456

1014 ->1034 0.13998

1015 ->1034 -0.11759

1028 ->1046 0.15353

Excited State 208: Singlet-A 4.2385 eV 292.52 nm f=0.0070

<S**2>=0.000

1009 ->1030 0.10299

1010 ->1031 0.10645

1011 ->1032 -0.10872

1012 ->1031 0.12501

1013 ->1035 -0.15718

1016 ->1038 -0.10845

1020 ->1040 0.29426

1022 ->1042 0.19832

1027 ->1044 0.18725

1029 ->1048 -0.13582

Excited State 209: Singlet-A 4.2454 eV 292.04 nm f=0.0040

<S**2>=0.000

1022 ->1042 -0.10211

1023 ->1042 0.12570

1025 ->1043 -0.14832

1026 ->1043 -0.15733

1026 ->1046 -0.18548

1027 ->1046 0.10654

1028 ->1046 0.20012

1029 ->1047 -0.10725

1029 ->1048 0.12549

1029 ->1049 0.24837

Excited State 210: Singlet-A 4.2487 eV 291.82 nm f=0.0183

<S**2>=0.000

1010 ->1031 -0.11558

1013 ->1034 0.17776

1015 ->1039 0.10626

1017 ->1038 0.16122

1021 ->1041 0.29821

1022 ->1041 -0.12423

1022 ->1042 0.17727

1023 ->1042 -0.12631

Excited State 211: Singlet-A 4.2543 eV 291.43 nm f=0.0094

<S**2>=0.000

1009 ->1030 -0.23400

1010 ->1031 -0.11198

1012 ->1031 -0.12927

1012 ->1034 0.11466

1013 ->1032 -0.17340

1014 ->1034 0.10626

1017 ->1039 0.10024

1019 ->1040 0.14208

1020 ->1040 0.26751

1029 ->1049 0.13274

Excited State 212: Singlet-A 4.2555 eV 291.35 nm f=0.0239

<S**2>=0.000

1012 ->1031 0.10675

1013 ->1031 0.10103

1013 ->1032 0.12347

1014 ->1037 -0.11651

1022 ->1042 0.11637

1029 ->1047 -0.13869

1029 ->1048 0.21006

1029 ->1049 0.33079

Excited State 213: Singlet-A 4.2570 eV 291.25 nm f=0.0010

<S**2>=0.000

1011 ->1031 -0.11437

1012 ->1030 -0.11478

1013 ->1038 -0.13382

1013 ->1039 -0.12458

1014 ->1037 0.28290

1015 ->1037 -0.17646

1015 ->1039 0.20624

1016 ->1039 -0.24566

1020 ->1040 0.14834

Excited State 214: Singlet-A 4.2597 eV 291.06 nm f=0.0101

<S**2>=0.000

1013 ->1037 -0.11683

1014 ->1037 0.27305

1015 ->1039 -0.17663

1017 ->1039 0.12161

1019 ->1039 0.10319

1022 ->1041 -0.13053

1022 ->1042 0.32124

1023 ->1042 -0.14362

Excited State 215: Singlet-A 4.2622 eV 290.90 nm f=0.0035

<S**2>=0.000

1010 ->1031 -0.13484

1010 ->1032 -0.10795

1011 ->1031 0.23451

1011 ->1032 -0.10771

1012 ->1032 -0.20118

1013 ->1031 -0.15251

1013 ->1032 -0.10621

1027 ->1044 -0.10021

1029 ->1049 0.17489

Excited State 216: Singlet-A 4.2694 eV 290.40 nm f=0.0007

<S**2>=0.000

1009 ->1031 0.11950

1010 ->1030 -0.16425

1010 ->1031 0.24135

1010 ->1032 0.12484

1010 ->1033 -0.11449

1011 ->1031 0.10653

1013 ->1035 0.15818

1027 ->1044 0.19781

Excited State 217: Singlet-A 4.2707 eV 290.32 nm f=0.0016

<S**2>=0.000

1010 ->1031 -0.14925

1013 ->1034 -0.10524

1025 ->1044 -0.10553

1027 ->1044 0.38290

1027 ->1048 -0.13557

1029 ->1053 0.11566

Excited State 218: Singlet-A 4.2729 eV 290.16 nm f=0.0034

<S**2>=0.000

1013 ->1038 -0.11253

1014 ->1038 -0.16886

1015 ->1038 -0.16635

1017 ->1038 -0.18026

1019 ->1040 0.21260

1021 ->1041 0.26029

1022 ->1042 -0.12630

1027 ->1044 -0.13380

Excited State 219: Singlet-A 4.2756 eV 289.98 nm f=0.0040

<S**2>=0.000

1010 ->1032 0.11040

1012 ->1031 0.16559

1013 ->1034 -0.13047

1015 ->1037 -0.13300

1015 ->1038 0.23408

1015 ->1039 -0.14204

1017 ->1038 0.19297

1019 ->1040 0.13764

1021 ->1041 0.12036

1022 ->1042 -0.10667

1026 ->1046 -0.10800

Excited State 220: Singlet-A 4.2796 eV 289.71 nm f=0.0027

<S**2>=0.000

1012 ->1031 -0.15178

1013 ->1032 -0.12215

1013 ->1033 0.13077

1013 ->1034 -0.16644

1013 ->1035 0.11021

1013 ->1037 0.12539

1014 ->1037 -0.10540

1015 ->1038 0.25797

1022 ->1042 0.12695

1027 ->1044 -0.10662

Excited State 221: Singlet-A 4.2842 eV 289.40 nm f=0.0072

<S**2>=0.000

1010 ->1032 -0.12317

1012 ->1035 -0.13375

1014 ->1038 0.11473

1019 ->1040 -0.14332

1021 ->1041 0.10210

1027 ->1045 0.13231

1029 ->1048 -0.17435

1029 ->1050 0.22209

1029 ->1053 0.12132

1029 ->1055 0.10879

Excited State 222: Singlet-A 4.2861 eV 289.27 nm f=0.0024

<S**2>=0.000

1008 ->1030 -0.13689

1009 ->1030 -0.10126

1009 ->1031 0.12434

1010 ->1032 0.12811

1011 ->1031 0.10998

1011 ->1032 -0.10244

1011 ->1033 0.12228

1013 ->1033 0.11004

1020 ->1040 -0.13456

1021 ->1041 -0.13924

1027 ->1045 0.17141

1029 ->1048 -0.14414

1029 ->1050 0.15340

1029 ->1052 -0.10441

1029 ->1053 0.12247

Excited State 223: Singlet-A 4.2892 eV 289.06 nm f=0.0012

<S**2>=0.000

1010 ->1031 0.34003

1010 ->1032 -0.14299

1010 ->1034 -0.10578

1011 ->1032 -0.12944

1011 ->1033 0.11292

1011 ->1036 -0.13952

1012 ->1032 -0.12794

1025 ->1043 -0.15215

1028 ->1043 0.13267

1029 ->1050 -0.11628

Excited State 224: Singlet-A 4.2925 eV 288.84 nm f=0.0072

<S**2>=0.000

1010 ->1031 0.16741

1011 ->1031 -0.11095

1011 ->1032 -0.12664

1013 ->1033 0.14005

1015 ->1038 -0.13660

1019 ->1040 -0.11078

1021 ->1041 0.11287

1025 ->1043 0.13347

1027 ->1045 -0.18508

1028 ->1043 -0.10686

1029 ->1050 0.24261

Excited State 225: Singlet-A 4.2962 eV 288.59 nm f=0.0098

<S**2>=0.000

1011 ->1033 -0.18664

1012 ->1032 -0.10722

1013 ->1032 0.11628

1013 ->1033 -0.17569

1013 ->1034 0.13094

1014 ->1038 -0.13565

1015 ->1038 0.16614

1021 ->1041 -0.19113

1027 ->1045 -0.16050

1029 ->1050 0.25592

1029 ->1053 0.10100

1029 ->1055 0.10452

Excited State 226: Singlet-A 4.2970 eV 288.54 nm f=0.0035

<S**2>=0.000

1010 ->1030 0.12653

1010 ->1032 -0.15211

1012 ->1033 0.15936

1013 ->1033 -0.12057

1013 ->1034 0.14215

1014 ->1038 0.13521

1019 ->1040 0.12702

1021 ->1041 -0.18135

1025 ->1043 0.20685

1026 ->1046 -0.11675

1027 ->1045 0.23862

1028 ->1043 -0.14121

Excited State 227: Singlet-A 4.2993 eV 288.38 nm f=0.0062

<S**2>=0.000

1009 ->1030 0.11747

1010 ->1033 0.17254

1011 ->1032 -0.18832

1011 ->1033 0.33038

1011 ->1034 0.10482

1011 ->1035 -0.13103

1012 ->1035 -0.11188

1013 ->1034 0.10425

1015 ->1038 0.15859

1020 ->1041 -0.10275

1027 ->1045 -0.16181

Excited State 228: Singlet-A 4.3044 eV 288.04 nm f=0.0007

<S**2>=0.000

1008 ->1030 0.20770

1011 ->1036 -0.16192

1012 ->1030 0.10388

1012 ->1034 0.22394

1014 ->1038 -0.13166

1014 ->1039 -0.10217

1019 ->1040 -0.21470

1025 ->1043 0.11006

1027 ->1045 0.22546

1028 ->1043 -0.10778

Excited State 229: Singlet-A 4.3074 eV 287.84 nm f=0.0050

<S**2>=0.000

1009 ->1031 0.15366

1010 ->1033 0.18396

1011 ->1032 0.22526

1012 ->1033 -0.13222

1013 ->1032 -0.17635

1013 ->1035 -0.15882

1014 ->1039 -0.13002

1015 ->1038 -0.12347

1019 ->1040 0.13759

1025 ->1043 0.10958

1027 ->1045 -0.15874

Excited State 230: Singlet-A 4.3079 eV 287.81 nm f=0.0052

<S**2>=0.000

1008 ->1030 0.27975

1009 ->1030 -0.10299

1009 ->1031 0.20360

1009 ->1036 0.13145

1010 ->1030 0.14645

1010 ->1033 0.10607

1011 ->1032 0.12443

1012 ->1032 0.10645

1012 ->1034 -0.15384

1012 ->1036 -0.13792

1013 ->1031 0.11206

1013 ->1036 -0.10527

1025 ->1043 -0.12762

1028 ->1043 0.12807

Excited State 231: Singlet-A 4.3120 eV 287.53 nm f=0.0111

<S**2>=0.000

1011 ->1032 0.12868

1011 ->1036 -0.11827

1012 ->1033 0.25094

1019 ->1040 -0.17346

1020 ->1041 -0.13222

1025 ->1043 0.13030

1027 ->1045 -0.16653

1028 ->1043 -0.15012

1029 ->1050 -0.15932

1029 ->1054 0.11118

Excited State 232: Singlet-A 4.3153 eV 287.31 nm f=0.0084

<S**2>=0.000

1007 ->1030 -0.12002

1008 ->1030 0.14523

1009 ->1031 -0.12023

1012 ->1034 0.15380

1013 ->1036 -0.11136

1013 ->1039 -0.10952

1014 ->1039 0.18144

1020 ->1041 0.29084

1027 ->1045 -0.11769

1029 ->1048 -0.10251

1029 ->1053 0.10527

Excited State 233: Singlet-A 4.3168 eV 287.21 nm f=0.0020

<S**2>=0.000

1009 ->1032 0.11935

1011 ->1032 0.18561

1011 ->1033 0.14454

1011 ->1034 0.13591

1012 ->1033 0.18177

1019 ->1040 0.15079

1029 ->1050 0.24947

1029 ->1053 -0.17368

1029 ->1055 -0.10957

Excited State 234: Singlet-A 4.3185 eV 287.10 nm f=0.0102

<S**2>=0.000

1011 ->1032 0.12417

1012 ->1033 0.11567

1027 ->1045 0.14344

1029 ->1051 0.29346

1029 ->1053 0.17521

1029 ->1054 -0.15442

1029 ->1055 0.18824

1029 ->1056 -0.18593

Excited State 235: Singlet-A 4.3220 eV 286.87 nm f=0.0013

<S**2>=0.000

1008 ->1030 0.20716

1010 ->1032 0.23816

1010 ->1033 0.11842

1011 ->1031 -0.13385

1011 ->1032 -0.11201

1011 ->1036 0.13201

1012 ->1031 -0.10693

1012 ->1035 0.18218

1018 ->1040 0.13107

1029 ->1053 0.13502

Excited State 236: Singlet-A 4.3250 eV 286.67 nm f=0.0056

<S**2>=0.000

1007 ->1030 0.12421

1010 ->1035 0.10296

1011 ->1034 0.17250

1011 ->1035 0.13149

1012 ->1034 0.15838

1012 ->1035 0.10958

1012 ->1036 0.11734

1012 ->1037 0.17579

1013 ->1034 -0.13867

1014 ->1038 0.11201

1014 ->1039 -0.17023

1015 ->1039 0.10858

1020 ->1041 0.15312

Excited State 237: Singlet-A 4.3275 eV 286.50 nm f=0.0171

<S**2>=0.000

1008 ->1030 0.11556

1009 ->1031 -0.16452

1010 ->1033 0.10111

1011 ->1031 -0.12677

1012 ->1034 0.13049

1014 ->1039 0.10275

1018 ->1040 -0.17064

1020 ->1041 -0.13428

1029 ->1047 -0.12121

1029 ->1051 0.27812

1029 ->1052 -0.10827

Excited State 238: Singlet-A 4.3321 eV 286.20 nm f=0.0001

<S**2>=0.000

1007 ->1030 0.18223

1010 ->1034 0.11559

1011 ->1034 0.19045

1011 ->1036 -0.15363

1013 ->1032 -0.10981

1013 ->1038 -0.16421

1014 ->1038 0.12325

1014 ->1039 0.15168

1018 ->1040 0.14176

1019 ->1041 -0.11137

1020 ->1041 -0.14202

1021 ->1041 -0.12701

Excited State 239: Singlet-A 4.3352 eV 286.00 nm f=0.0083

<S**2>=0.000

1008 ->1030 -0.11322

1011 ->1036 0.13691

1012 ->1035 -0.18394

1013 ->1034 -0.12388

1013 ->1039 0.12337

1014 ->1038 -0.10595

1015 ->1039 0.16480

1018 ->1040 0.22173

1028 ->1043 0.13032

1029 ->1051 0.15977

Excited State 240: Singlet-A 4.3365 eV 285.91 nm f=0.0024

<S**2>=0.000

1012 ->1035 0.11210

1018 ->1040 0.10832

1027 ->1045 -0.12520

1029 ->1047 -0.10133

1029 ->1050 0.11265

1029 ->1051 0.33104

1029 ->1054 0.20375

1029 ->1055 -0.13838

Excited State 241: Singlet-A 4.3385 eV 285.78 nm f=0.0106

<S**2>=0.000

1010 ->1032 0.11675

1011 ->1034 0.10074

1012 ->1031 -0.11645

1012 ->1032 -0.10841

1012 ->1033 0.12785

1012 ->1034 -0.19984

1013 ->1034 0.14931

1013 ->1035 -0.12713

1013 ->1036 0.12386

1014 ->1038 -0.10098

1020 ->1041 0.25649

Excited State 242: Singlet-A 4.3413 eV 285.60 nm f=0.0011

<S**2>=0.000

1007 ->1030 -0.11231

1008 ->1031 -0.12139

1010 ->1036 0.11694

1011 ->1032 0.11481

1011 ->1034 -0.10986

1012 ->1035 0.24351

1012 ->1036 0.15022

1012 ->1038 -0.12163

1013 ->1036 0.16678

1013 ->1037 0.11214

1013 ->1038 -0.13285

1014 ->1038 0.13419

1014 ->1039 0.13589

1019 ->1040 -0.15226

Excited State 243: Singlet-A 4.3437 eV 285.44 nm f=0.0085

<S**2>=0.000

1010 ->1033 0.14736

1011 ->1036 0.11863

1013 ->1038 -0.10899

1014 ->1039 0.11146

1023 ->1044 0.10239

1026 ->1044 0.28067

1027 ->1044 -0.14400

1028 ->1044 -0.27301

1028 ->1046 0.18025

Excited State 244: Singlet-A 4.3457 eV 285.30 nm f=0.0090

<S**2>=0.000

1007 ->1031 -0.11558

1009 ->1033 -0.14914

1011 ->1033 -0.18631

1011 ->1036 0.13337

1012 ->1034 0.10803

1012 ->1036 -0.10905

1013 ->1034 -0.12778

1013 ->1036 0.20053

1014 ->1039 0.11604

1026 ->1044 -0.17238

1028 ->1044 0.22533

Excited State 245: Singlet-A 4.3485 eV 285.12 nm f=0.0018

<S**2>=0.000

1007 ->1030 0.11794

1009 ->1033 0.10354

1012 ->1035 -0.10737

1012 ->1036 0.10004

1012 ->1037 0.12275

1013 ->1034 0.10056

1013 ->1036 -0.20470

1013 ->1039 0.10974

1014 ->1039 0.11359

1020 ->1041 0.14490

1026 ->1044 -0.17273

1028 ->1044 0.24482

Excited State 246: Singlet-A 4.3487 eV 285.11 nm f=0.0028

<S**2>=0.000

1009 ->1031 0.15990

1010 ->1034 -0.10682

1011 ->1035 0.18634

1012 ->1036 0.13327

1013 ->1036 0.10636

1013 ->1037 -0.19700

1013 ->1038 0.15045

1014 ->1039 0.26625

1015 ->1039 0.11550

Excited State 247: Singlet-A 4.3518 eV 284.91 nm f=0.0070

<S**2>=0.000

1008 ->1031 -0.10164

1009 ->1036 0.13125

1010 ->1033 -0.18627

1011 ->1032 0.12935

1011 ->1033 0.11748

1011 ->1034 0.16613

1013 ->1035 -0.13647

1014 ->1038 -0.10842

1019 ->1040 0.10647

1025 ->1046 -0.12112

1026 ->1046 0.15073

1027 ->1046 -0.13271

1028 ->1046 0.20209

1028 ->1047 -0.10544

1029 ->1051 -0.10400

Excited State 248: Singlet-A 4.3546 eV 284.72 nm f=0.0030

<S**2>=0.000

1007 ->1030 0.10617

1008 ->1031 0.10841

1009 ->1031 0.20656

1009 ->1032 -0.16697

1010 ->1036 0.18652

1011 ->1034 0.11880

1011 ->1035 -0.15910

1012 ->1036 0.12244

1013 ->1038 0.12236

1013 ->1039 -0.15202

1015 ->1039 -0.12889

1018 ->1040 0.10610

1019 ->1041 -0.10916

Excited State 249: Singlet-A 4.3605 eV 284.34 nm f=0.0035

<S**2>=0.000

1008 ->1031 0.11289

1011 ->1035 -0.11490

1011 ->1036 -0.11299

1013 ->1036 0.11157

1014 ->1038 0.13712

1018 ->1040 0.30529

1018 ->1041 -0.12858

1019 ->1040 0.12328

1029 ->1052 -0.18751

Excited State 250: Singlet-A 4.3615 eV 284.27 nm f=0.0079

<S**2>=0.000

1010 ->1034 0.16484

1011 ->1034 0.11742

1013 ->1036 -0.10431

1018 ->1040 -0.11389

1019 ->1041 0.19381

1029 ->1048 -0.16133

1029 ->1053 0.12205

1029 ->1055 -0.13730

1029 ->1057 0.10494

Excited State 251: Singlet-A 4.3647 eV 284.06 nm f=0.0036

<S**2>=0.000

1007 ->1030 0.11063

1009 ->1032 0.10620

1010 ->1034 -0.11397

1011 ->1033 0.11425

1011 ->1035 -0.11880

1011 ->1036 0.13192

1012 ->1037 0.13888

1013 ->1035 -0.12324

1019 ->1041 0.17001

1026 ->1047 0.11300

1026 ->1049 0.12657

1027 ->1049 -0.10026

1028 ->1049 -0.11448

1029 ->1050 -0.12079

1029 ->1052 0.14783

1029 ->1053 0.13475

Excited State 252: Singlet-A 4.3665 eV 283.95 nm f=0.0049

<S**2>=0.000

1008 ->1030 -0.10570

1009 ->1033 -0.11665

1009 ->1035 0.10522

1010 ->1032 -0.18267

1010 ->1033 0.21949

1010 ->1034 0.18967

1012 ->1032 0.12887

1012 ->1034 -0.10649

1013 ->1036 -0.13598

1018 ->1040 0.14884

1019 ->1041 0.13077

Excited State 253: Singlet-A 4.3692 eV 283.77 nm f=0.0178

<S**2>=0.000

1008 ->1031 0.11853

1009 ->1033 0.11214

1010 ->1032 0.11249

1010 ->1034 -0.13960

1010 ->1035 -0.11958

1018 ->1040 -0.12615

1026 ->1049 -0.12740

1027 ->1047 0.14105

1027 ->1049 0.18187

1027 ->1050 0.10051

1028 ->1047 0.16822

1028 ->1049 0.16503

1029 ->1050 0.15306

Excited State 254: Singlet-A 4.3714 eV 283.63 nm f=0.0126

<S**2>=0.000

1007 ->1030 0.10171

1008 ->1031 0.27497

1009 ->1032 0.11268

1010 ->1032 -0.11801

1010 ->1035 -0.19288

1011 ->1035 -0.11124

1012 ->1031 0.12425

1012 ->1032 0.16749

1012 ->1035 0.16661

1012 ->1037 -0.21256

1013 ->1039 0.15689

Excited State 255: Singlet-A 4.3732 eV 283.51 nm f=0.0175

<S**2>=0.000

1007 ->1030 0.17391

1009 ->1032 0.13456

1009 ->1036 0.13386

1010 ->1033 0.16155

1012 ->1037 0.11483

1013 ->1038 0.14810

1028 ->1047 0.16437

Excited State 256: Singlet-A 4.3765 eV 283.30 nm f=0.0008

<S**2>=0.000

1004 ->1030 0.10392

1019 ->1041 -0.11554

1027 ->1047 0.12151

1028 ->1047 -0.20984

1029 ->1052 0.22297

1029 ->1053 0.20176

1029 ->1056 0.11888

1029 ->1057 0.12427

Excited State 257: Singlet-A 4.3799 eV 283.08 nm f=0.0097

<S**2>=0.000

1009 ->1033 0.10004

1010 ->1034 -0.13643

1011 ->1035 0.21411

1012 ->1037 -0.17800

1013 ->1038 -0.15743

1013 ->1039 -0.14216

1014 ->1037 -0.12746

1015 ->1039 -0.11592

1029 ->1052 -0.15408

1029 ->1054 -0.14075

Excited State 258: Singlet-A 4.3808 eV 283.02 nm f=0.0011

<S**2>=0.000

1007 ->1031 0.12384

1009 ->1033 0.11345

1009 ->1036 0.13271

1013 ->1037 -0.13267

1014 ->1039 -0.16973

1021 ->1042 0.19941

1029 ->1052 0.28110

Excited State 259: Singlet-A 4.3859 eV 282.69 nm f=0.0118

<S**2>=0.000

1010 ->1032 -0.12215

1010 ->1033 -0.10347

1010 ->1034 0.14187

1010 ->1035 0.21340

1011 ->1033 0.11317

1011 ->1034 -0.16966

1019 ->1041 0.14626

1027 ->1047 0.18443

1027 ->1049 0.14063

1027 ->1050 0.10644

1029 ->1052 -0.12305

1029 ->1056 0.11575

Excited State 260: Singlet-A 4.3862 eV 282.67 nm f=0.0111

<S**2>=0.000

1011 ->1034 0.11114

1012 ->1037 0.16491

1013 ->1037 0.11038

1019 ->1041 -0.14048

1026 ->1047 -0.15432

1028 ->1046 -0.15414

1028 ->1047 -0.15604

1029 ->1053 0.11559

1029 ->1054 -0.12384

1029 ->1055 -0.13819

1029 ->1058 0.15003

Excited State 261: Singlet-A 4.3884 eV 282.53 nm f=0.0030

<S**2>=0.000

1009 ->1036 -0.13744

1011 ->1033 0.13636

1011 ->1036 0.10788

1012 ->1035 0.10251

1013 ->1036 -0.11496

1018 ->1040 0.11863

1020 ->1041 -0.12329

1029 ->1050 0.10638

1029 ->1052 0.25971

1029 ->1054 0.16034

Excited State 262: Singlet-A 4.3922 eV 282.29 nm f=0.0025

<S**2>=0.000

1007 ->1031 0.22682

1007 ->1032 -0.14046

1009 ->1034 0.19971

1009 ->1035 0.24070

1010 ->1034 -0.12865

1010 ->1035 0.18762

1012 ->1036 -0.11544

1012 ->1037 -0.19110

1015 ->1037 -0.10871

Excited State 263: Singlet-A 4.3932 eV 282.22 nm f=0.0048

<S**2>=0.000

1007 ->1030 -0.13969

1009 ->1035 0.12424

1012 ->1037 0.12661

1013 ->1035 -0.11115

1013 ->1037 -0.13531

1016 ->1040 0.10028

1019 ->1041 -0.14017

1026 ->1047 0.11642

1027 ->1043 -0.10333

1027 ->1047 0.17005

1028 ->1049 -0.16617

1029 ->1056 0.16892

Excited State 264: Singlet-A 4.3946 eV 282.13 nm f=0.0014

<S**2>=0.000

1010 ->1034 -0.10715

1010 ->1036 0.13066

1011 ->1034 0.14882

1011 ->1035 0.11584

1013 ->1037 0.21613

1014 ->1038 0.10980

1026 ->1047 0.11114

1027 ->1049 0.10454

1028 ->1049 -0.10420

1029 ->1052 0.13641

1029 ->1057 -0.12874

Excited State 265: Singlet-A 4.3957 eV 282.06 nm f=0.0081

<S**2>=0.000

1019 ->1041 -0.15032

1021 ->1042 0.13417

1025 ->1045 0.15847

1026 ->1045 0.11354

1028 ->1045 0.13066

1028 ->1047 0.24367

1028 ->1048 -0.10986

1029 ->1052 0.14361

Excited State 266: Singlet-A 4.3993 eV 281.83 nm f=0.0100

<S**2>=0.000

1007 ->1030 -0.17419

1008 ->1032 0.11775

1009 ->1031 0.10539

1009 ->1032 0.23607

1010 ->1037 -0.10193

1011 ->1035 -0.15049

1011 ->1037 -0.10998

1013 ->1037 -0.14435

1025 ->1045 0.11645

Excited State 267: Singlet-A 4.4013 eV 281.70 nm f=0.0057

<S**2>=0.000

1001 ->1037 -0.10395

1009 ->1032 0.13494

1013 ->1037 0.11938

1027 ->1043 0.14286

1027 ->1048 0.16554

1027 ->1049 -0.10603

1029 ->1053 -0.13506

1029 ->1054 -0.14910

1029 ->1055 0.14762

Excited State 268: Singlet-A 4.4042 eV 281.51 nm f=0.0066

<S**2>=0.000

1009 ->1032 -0.16768

1010 ->1034 -0.13252

1011 ->1034 0.13474

1017 ->1040 0.13494

1018 ->1041 -0.14169

1019 ->1041 0.21729

1020 ->1041 -0.11374

1021 ->1042 -0.11619

1025 ->1045 0.12451

1028 ->1045 0.10697

1029 ->1057 0.10339

Excited State 269: Singlet-A 4.4053 eV 281.44 nm f=0.0037

<S**2>=0.000

1008 ->1031 -0.15098

1010 ->1034 -0.11854

1025 ->1045 0.24815

1026 ->1045 0.15648

1027 ->1047 0.11056

1028 ->1045 0.18879

1029 ->1054 0.17430

1029 ->1056 -0.11173

Excited State 270: Singlet-A 4.4074 eV 281.31 nm f=0.0007

<S**2>=0.000

1008 ->1031 0.13586

1009 ->1033 0.15406

1010 ->1037 0.10062

1011 ->1035 0.16477

1011 ->1037 0.19233

1013 ->1037 0.18072

1013 ->1038 0.14178

1017 ->1040 -0.15162

1025 ->1045 0.13772

1028 ->1045 0.11997

Excited State 271: Singlet-A 4.4101 eV 281.13 nm f=0.0028

<S**2>=0.000

1007 ->1030 -0.13691

1007 ->1031 -0.14437

1008 ->1033 0.10650

1008 ->1034 -0.13554

1009 ->1033 0.17122

1010 ->1035 0.14812

1011 ->1033 -0.14025

1011 ->1034 0.20499

1011 ->1035 -0.12254

1012 ->1038 -0.10721

1017 ->1040 0.14762

Excited State 272: Singlet-A 4.4122 eV 281.00 nm f=0.0031

<S**2>=0.000

1004 ->1030 0.10428

1011 ->1035 0.10054

1012 ->1038 0.16090

1013 ->1037 0.10831

1021 ->1042 0.22740

1027 ->1043 -0.17919

1028 ->1049 0.16176

1028 ->1050 -0.14664

1029 ->1055 -0.14830

1029 ->1057 -0.11315

Excited State 273: Singlet-A 4.4129 eV 280.96 nm f=0.0113

<S**2>=0.000

1009 ->1032 -0.10583

1010 ->1034 -0.10315

1010 ->1035 0.12512

1012 ->1038 0.17152

1021 ->1042 0.30588

1027 ->1043 0.24659

Excited State 274: Singlet-A 4.4146 eV 280.85 nm f=0.0013

<S**2>=0.000

1004 ->1030 -0.11395

1017 ->1040 0.14047

1021 ->1042 0.27920

1025 ->1045 -0.10079

1026 ->1045 -0.10236

1027 ->1043 -0.18590

1028 ->1045 -0.11518

1028 ->1049 -0.15267

1028 ->1050 0.16621

Excited State 275: Singlet-A 4.4172 eV 280.69 nm f=0.0121

<S**2>=0.000

1007 ->1030 -0.10411

1007 ->1031 0.13193

1007 ->1033 0.12715

1007 ->1034 0.11859

1008 ->1032 -0.16278

1008 ->1033 0.13476

1008 ->1034 0.11786

1009 ->1036 -0.10233

1016 ->1040 -0.15786

1017 ->1040 0.18722

1029 ->1054 0.13695

1029 ->1055 0.11161

Excited State 276: Singlet-A 4.4207 eV 280.46 nm f=0.0021

<S**2>=0.000

1007 ->1033 0.17464

1010 ->1036 -0.11185

1012 ->1036 0.10018

1017 ->1040 -0.17414

1027 ->1043 0.29957

1029 ->1057 -0.10623

Excited State 277: Singlet-A 4.4226 eV 280.34 nm f=0.0063

<S**2>=0.000

1009 ->1032 0.14015

1010 ->1035 -0.12617

1012 ->1038 -0.13406

1016 ->1040 0.14806

1017 ->1040 0.11054

1018 ->1041 -0.16558

1029 ->1056 0.19768

1029 ->1057 0.21737

1029 ->1060 -0.11399

Excited State 278: Singlet-A 4.4251 eV 280.18 nm f=0.0083

<S**2>=0.000

1010 ->1035 -0.10782

1011 ->1035 0.13812

1012 ->1036 -0.12257

1017 ->1040 0.17304

1018 ->1041 0.24706

1025 ->1043 -0.11364

1027 ->1043 0.23574

1029 ->1056 0.15748

Excited State 279: Singlet-A 4.4274 eV 280.04 nm f=0.0032

<S**2>=0.000

1007 ->1032 0.10300

1008 ->1031 0.20707

1008 ->1032 -0.12583

1008 ->1033 -0.14566

1009 ->1036 0.10772

1027 ->1043 0.11234

1029 ->1054 0.19292

1029 ->1058 0.15167

Excited State 280: Singlet-A 4.4292 eV 279.93 nm f=0.0057

<S**2>=0.000

1010 ->1036 -0.17562

1012 ->1036 0.20070

1012 ->1037 -0.12298

1017 ->1040 0.18630

1029 ->1052 -0.11033

1029 ->1054 -0.14937

1029 ->1055 -0.18574

1029 ->1058 -0.15324

Excited State 281: Singlet-A 4.4297 eV 279.90 nm f=0.0042

<S**2>=0.000

939 ->1039 -0.11674

1008 ->1032 -0.13967

1009 ->1036 0.14604

1010 ->1037 -0.12593

1010 ->1038 0.10456

1012 ->1036 0.20489

1017 ->1040 -0.15335

1018 ->1041 0.10465

1027 ->1043 -0.12922

1029 ->1053 -0.10028

1029 ->1055 0.23119

1029 ->1056 0.15343

Excited State 282: Singlet-A 4.4321 eV 279.74 nm f=0.0063

<S**2>=0.000

1016 ->1040 0.25191

1018 ->1041 0.22286

1029 ->1056 -0.12744

1029 ->1058 -0.19829

Excited State 283: Singlet-A 4.4338 eV 279.64 nm f=0.0086

<S**2>=0.000

1007 ->1032 0.11790

1008 ->1032 -0.15997

1008 ->1034 -0.12372

1009 ->1032 0.10354

1010 ->1036 0.22147

1012 ->1036 -0.12681

1016 ->1040 -0.14177

1018 ->1041 -0.13067

1027 ->1043 -0.12533

1029 ->1054 -0.11758

1029 ->1058 -0.17508

Excited State 284: Singlet-A 4.4366 eV 279.46 nm f=0.0064

<S**2>=0.000

1007 ->1033 0.11920

1009 ->1035 -0.10014

1018 ->1041 0.19447

1020 ->1042 -0.13581

1025 ->1047 -0.10639

1027 ->1048 0.11728

1029 ->1058 0.15267

Excited State 285: Singlet-A 4.4377 eV 279.39 nm f=0.0013

<S**2>=0.000

939 ->1031 0.12103

939 ->1035 -0.11909

939 ->1039 -0.40341

940 ->1039 0.13938

1010 ->1039 0.10648

Excited State 286: Singlet-A 4.4396 eV 279.27 nm f=0.0033

<S**2>=0.000

939 ->1039 0.20685

1016 ->1040 0.17980

1020 ->1042 -0.13852

Excited State 287: Singlet-A 4.4411 eV 279.18 nm f=0.0028

<S**2>=0.000

934 ->1033 -0.13287

934 ->1037 -0.18118

936 ->1038 0.11520

939 ->1039 -0.10757

1007 ->1030 -0.13252

1008 ->1031 0.14901

1029 ->1058 -0.14646

Excited State 288: Singlet-A 4.4427 eV 279.08 nm f=0.0008

<S**2>=0.000

934 ->1033 -0.15586

934 ->1034 0.11681

934 ->1037 -0.21818

936 ->1038 -0.10846

Excited State 289: Singlet-A 4.4433 eV 279.03 nm f=0.0036

<S**2>=0.000

920 ->1030 0.14964

920 ->1031 -0.22646

920 ->1032 -0.20923

1016 ->1040 0.10635

1020 ->1042 0.13563

1029 ->1058 0.16074

Excited State 290: Singlet-A 4.4446 eV 278.96 nm f=0.0096

<S**2>=0.000

936 ->1038 -0.13038

939 ->1039 -0.13475

1009 ->1034 0.15281

1010 ->1037 0.18079

1011 ->1037 0.10609

1012 ->1038 0.15708

1025 ->1047 -0.14241

1029 ->1058 -0.12736

Excited State 291: Singlet-A 4.4459 eV 278.87 nm f=0.0040

<S**2>=0.000

930 ->1032 0.10445

930 ->1033 0.10771

932 ->1031 -0.13980

932 ->1033 0.11910

932 ->1035 0.18588

932 ->1039 -0.10868

1009 ->1033 -0.11202

1011 ->1035 -0.11265

1011 ->1037 0.11085

1020 ->1042 0.18992

Excited State 292: Singlet-A 4.4480 eV 278.74 nm f=0.0034

<S**2>=0.000

920 ->1030 -0.10427

934 ->1037 0.12499

1007 ->1031 -0.10092

1008 ->1033 0.13458

1009 ->1034 0.10803

Excited State 293: Singlet-A 4.4492 eV 278.67 nm f=0.0050

<S**2>=0.000

936 ->1038 0.15011

1009 ->1033 0.10883

1018 ->1041 0.10652

1020 ->1042 0.31765

Excited State 294: Singlet-A 4.4502 eV 278.60 nm f=0.0096

<S**2>=0.000

921 ->1030 0.10175

928 ->1030 -0.10588

930 ->1036 -0.11665

933 ->1035 0.10216

936 ->1038 0.17622

1020 ->1042 -0.10073

1025 ->1047 0.16202

1029 ->1057 -0.13610

Excited State 295: Singlet-A 4.4514 eV 278.53 nm f=0.0041

<S**2>=0.000

920 ->1030 -0.10787

921 ->1030 0.18576

921 ->1032 0.12853

921 ->1036 -0.10695

928 ->1036 0.16260

930 ->1036 0.15240

936 ->1038 -0.10565

Excited State 296: Singlet-A 4.4515 eV 278.52 nm f=0.0047

<S**2>=0.000

922 ->1033 0.12311

922 ->1034 0.16359

923 ->1032 -0.10268

923 ->1033 0.13420

923 ->1034 0.12193

936 ->1038 -0.13900

1007 ->1032 0.10140

1025 ->1047 0.12562

Excited State 297: Singlet-A 4.4529 eV 278.43 nm f=0.0009

<S**2>=0.000

922 ->1032 -0.13314

922 ->1033 0.16910

922 ->1034 0.19166

922 ->1036 -0.13312

923 ->1032 -0.10433

923 ->1033 0.15977

923 ->1034 0.17891

936 ->1038 0.15693

1029 ->1056 -0.10080

1029 ->1058 0.11884

Excited State 298: Singlet-A 4.4537 eV 278.39 nm f=0.0014

<S**2>=0.000

921 ->1030 -0.12698

923 ->1036 0.11871

930 ->1035 -0.13472

1008 ->1033 0.10579

1016 ->1040 0.10988

1025 ->1047 0.11230

Excited State 299: Singlet-A 4.4563 eV 278.22 nm f=0.0037

<S**2>=0.000

928 ->1035 0.09798

930 ->1035 -0.10868

930 ->1036 -0.10727

933 ->1035 0.10190

936 ->1038 -0.10129

1010 ->1035 -0.09896

1029 ->1056 -0.10272

1029 ->1057 0.10850

Excited State 300: Singlet-A 4.4570 eV 278.18 nm f=0.0032

<S**2>=0.000

936 ->1038 0.20495

1007 ->1032 0.12957

1008 ->1034 -0.10029

1009 ->1031 0.10973

1010 ->1037 0.14306

1010 ->1039 -0.15548

1012 ->1038 0.16131

1012 ->1039 -0.13099

Part 2

Excited State 1: Singlet-A 4.4570 eV 278.18 nm f=0.0032

<S**2>=0.000

936 ->1038 0.20398

1007 ->1032 0.12960

1008 ->1034 -0.10056

1009 ->1031 0.10975

1010 ->1037 0.14321

1010 ->1039 -0.15546

1012 ->1038 0.16159

1012 ->1039 -0.13118

This state for optimization and/or second-order correction.

Total Energy, E(TD-HF/TD-DFT) = -18684.5579210

Copying the excited state density for this state as the 1-particle RhoCI density.

Excited State 2: Singlet-A 4.4599 eV 278.00 nm f=0.0023

<S**2>=0.000

1007 ->1031 0.10001

1007 ->1034 -0.12914

1008 ->1033 -0.10491

1009 ->1033 0.16211

1009 ->1034 -0.11766

1009 ->1036 -0.10851

1018 ->1041 0.10005

1028 ->1052 -0.11138

1029 ->1056 0.12634

Excited State 3: Singlet-A 4.4615 eV 277.90 nm f=0.0135

<S**2>=0.000

936 ->1038 0.12655

1007 ->1031 -0.12684

1008 ->1032 -0.11357

1009 ->1033 0.10666

1009 ->1034 0.17808

1010 ->1038 -0.10050

1010 ->1039 0.15887

1011 ->1039 0.12249

1012 ->1038 0.22668

1029 ->1057 -0.10306

Excited State 4: Singlet-A 4.4628 eV 277.82 nm f=0.0005

<S**2>=0.000

1009 ->1034 -0.10319

1026 ->1044 0.13470

1026 ->1047 -0.13005

1027 ->1050 -0.10330

1028 ->1044 0.20911

1028 ->1052 -0.16838

1029 ->1058 0.12746

1029 ->1059 -0.18114

Excited State 5: Singlet-A 4.4664 eV 277.59 nm f=0.0070

<S**2>=0.000

1008 ->1032 -0.16548

1008 ->1033 0.29704

1008 ->1034 -0.23351

1010 ->1035 -0.10935

1010 ->1036 0.10616

1011 ->1038 -0.11374

1020 ->1042 0.12378

1029 ->1056 0.11631

Excited State 6: Singlet-A 4.4669 eV 277.56 nm f=0.0028

<S**2>=0.000

934 ->1037 -0.12103

1009 ->1034 -0.15062

1010 ->1038 0.11179

1010 ->1039 0.10130

1011 ->1037 0.23425

1016 ->1040 0.11805

1019 ->1042 -0.15387

1020 ->1042 0.11921

1025 ->1047 0.14454

1026 ->1049 -0.11669

Excited State 7: Singlet-A 4.4709 eV 277.31 nm f=0.0035

<S**2>=0.000

1007 ->1031 0.15374

1008 ->1032 0.14006

1009 ->1033 -0.10939

1009 ->1034 -0.19222

1012 ->1039 0.14707

1025 ->1047 -0.12579

1026 ->1043 -0.10093

1029 ->1060 0.20028

Excited State 8: Singlet-A 4.4715 eV 277.27 nm f=0.0020

<S**2>=0.000

1009 ->1034 0.15140

1017 ->1041 -0.10967

1020 ->1042 -0.11180

1026 ->1049 -0.12691

1027 ->1050 -0.16367

1028 ->1044 0.13979

1029 ->1059 0.17078

1029 ->1060 0.17867

Excited State 9: Singlet-A 4.4729 eV 277.19 nm f=0.0114

<S**2>=0.000

1010 ->1037 0.10663

1011 ->1037 -0.12358

1017 ->1041 0.12133

1025 ->1044 -0.11860

1026 ->1044 0.24034

1026 ->1047 0.10367

1028 ->1044 0.30754

Excited State 10: Singlet-A 4.4751 eV 277.05 nm f=0.0057

<S**2>=0.000

1008 ->1034 -0.13736

1008 ->1037 0.10214

1008 ->1038 0.10180

1010 ->1037 0.27008

1011 ->1037 -0.21803

1011 ->1038 0.12509

1013 ->1038 0.10425

1015 ->1040 -0.10784

1026 ->1044 -0.10671

1027 ->1050 -0.10816

1028 ->1044 -0.12955

Excited State 11: Singlet-A 4.4773 eV 276.92 nm f=0.0066

<S**2>=0.000

1008 ->1033 0.12622

1010 ->1038 0.15970

1010 ->1039 0.18407

1019 ->1042 0.28929

1029 ->1057 0.15714

1029 ->1059 -0.19178

Excited State 12: Singlet-A 4.4778 eV 276.88 nm f=0.0026

<S**2>=0.000

1008 ->1034 0.10301

1009 ->1038 0.10849

1010 ->1038 0.16516

1011 ->1038 0.20180

1012 ->1038 -0.16673

1012 ->1039 -0.17002

1013 ->1039 -0.10662

1016 ->1040 -0.12080

1029 ->1059 0.26842

Excited State 13: Singlet-A 4.4795 eV 276.78 nm f=0.0007

<S**2>=0.000

1007 ->1032 0.11650

1008 ->1033 0.13423

1010 ->1038 0.10390

1012 ->1039 0.20824

1019 ->1042 -0.13031

1027 ->1050 0.10210

1028 ->1051 -0.13090

1029 ->1060 -0.17832

Excited State 14: Singlet-A 4.4832 eV 276.56 nm f=0.0094

<S**2>=0.000

1011 ->1038 -0.11911

1016 ->1041 -0.10419

1019 ->1042 0.23268

1026 ->1043 -0.10818

1026 ->1050 0.10891

1029 ->1059 0.24668

1029 ->1060 -0.13542

1029 ->1061 -0.11827

Excited State 15: Singlet-A 4.4849 eV 276.45 nm f=0.0113

<S**2>=0.000

1019 ->1042 0.10961

1025 ->1043 -0.17498

1026 ->1043 0.27984

1026 ->1048 0.14469

1026 ->1050 -0.10558

1027 ->1053 -0.14835

1028 ->1043 -0.13452

1028 ->1054 -0.12106

1029 ->1059 0.11439

Excited State 16: Singlet-A 4.4860 eV 276.38 nm f=0.0091

<S**2>=0.000

1009 ->1036 -0.10881

1009 ->1037 -0.10817

1010 ->1039 -0.10247

1012 ->1039 0.19739

1013 ->1039 0.14148

1017 ->1041 0.18372

1026 ->1043 -0.10646

1027 ->1053 -0.10965

1028 ->1051 0.10042

1029 ->1059 0.17805

Excited State 17: Singlet-A 4.4874 eV 276.30 nm f=0.0072

<S**2>=0.000

1019 ->1042 -0.18372

1024 ->1043 0.17338

1025 ->1043 -0.12970

1026 ->1043 0.22185

1026 ->1049 0.10246

1026 ->1050 0.12805

1027 ->1046 0.18826

1027 ->1049 0.10146

1027 ->1050 0.15147

1028 ->1043 -0.11718

1028 ->1050 -0.10408

Excited State 18: Singlet-A 4.4883 eV 276.24 nm f=0.0025

<S**2>=0.000

1010 ->1039 -0.18286

1011 ->1038 0.10094

1012 ->1039 0.35771

1013 ->1039 0.17613

1019 ->1042 0.18336

1026 ->1043 0.11756

1027 ->1053 0.13421

Excited State 19: Singlet-A 4.4908 eV 276.09 nm f=0.0042

<S**2>=0.000

1007 ->1033 0.12815

1007 ->1034 -0.21762

1009 ->1037 -0.10849

1010 ->1038 0.14313

1010 ->1039 0.10030

1011 ->1038 -0.10212

1012 ->1039 -0.11558

1017 ->1041 0.23414

1027 ->1046 -0.15257

1027 ->1048 -0.12102

Excited State 20: Singlet-A 4.4960 eV 275.77 nm f=0.0164

<S**2>=0.000

1007 ->1033 0.10418

1007 ->1034 -0.10882

1008 ->1036 -0.10067

1019 ->1042 0.10099

1024 ->1043 -0.15893

1025 ->1050 -0.11356

1026 ->1050 0.21112

1027 ->1046 0.25998

1027 ->1049 0.14086

1028 ->1050 -0.12244

Excited State 21: Singlet-A 4.4991 eV 275.58 nm f=0.0042

<S**2>=0.000

1007 ->1031 -0.10476

1007 ->1033 0.14978

1008 ->1032 -0.10390

1008 ->1034 0.11082

1008 ->1035 0.15108

1009 ->1034 -0.15197

1009 ->1035 0.18583

1009 ->1036 0.13091

1016 ->1041 0.26445

Excited State 22: Singlet-A 4.4995 eV 275.55 nm f=0.0110

<S**2>=0.000

1008 ->1032 -0.10656

1008 ->1034 0.10984

1008 ->1037 0.13443

1011 ->1037 -0.10222

1011 ->1038 -0.21852

1024 ->1043 -0.14934

1026 ->1043 0.12703

1028 ->1052 0.23953

1029 ->1060 -0.15728

Excited State 23: Singlet-A 4.5013 eV 275.44 nm f=0.0059

<S**2>=0.000

1007 ->1031 -0.12551

1008 ->1032 -0.15405

1008 ->1034 0.10087

1008 ->1035 -0.12901

1008 ->1037 0.12924

1011 ->1038 -0.12584

1016 ->1041 -0.14511

1025 ->1045 -0.13646

1028 ->1045 0.21556

1028 ->1052 -0.11866

1029 ->1061 0.11579

Excited State 24: Singlet-A 4.5035 eV 275.30 nm f=0.0006

<S**2>=0.000

1007 ->1032 0.11614

1007 ->1033 0.16089

1009 ->1035 0.23142

1009 ->1037 -0.12923

1011 ->1039 0.12936

1016 ->1041 -0.14471

1017 ->1041 -0.10595

1025 ->1048 -0.15855

1028 ->1045 0.12410

1028 ->1048 -0.12409

Excited State 25: Singlet-A 4.5052 eV 275.20 nm f=0.0010

<S**2>=0.000

1011 ->1038 0.13307

1015 ->1040 -0.12778

1016 ->1041 0.28045

1021 ->1045 -0.10835

1025 ->1045 -0.16426

1028 ->1045 0.25469

1028 ->1052 0.10891

1029 ->1060 -0.10870

Excited State 26: Singlet-A 4.5077 eV 275.05 nm f=0.0028

<S**2>=0.000

1008 ->1035 0.20261

1009 ->1035 -0.18571

1010 ->1037 0.11087

1010 ->1039 -0.17131

1011 ->1038 -0.13839

1011 ->1039 0.23699

1013 ->1039 -0.10293

1017 ->1041 -0.14115

1018 ->1042 0.11065

1025 ->1045 -0.13216

1028 ->1045 0.25454

Excited State 27: Singlet-A 4.5108 eV 274.86 nm f=0.0102

<S**2>=0.000

1007 ->1032 0.10532

1008 ->1035 0.21128

1015 ->1040 -0.19530

1018 ->1042 0.12708

1019 ->1042 0.12012

1026 ->1045 0.30123

1026 ->1046 -0.11018

1027 ->1045 -0.13390

1028 ->1045 -0.22179

Excited State 28: Singlet-A 4.5118 eV 274.80 nm f=0.0107

<S**2>=0.000

1006 ->1030 -0.12835

1007 ->1032 -0.14897

1007 ->1035 0.11816

1008 ->1035 -0.14004

1009 ->1035 -0.13391

1024 ->1043 0.18277

1026 ->1045 0.10247

1027 ->1046 -0.15180

1028 ->1045 -0.11332

1028 ->1051 -0.11769

1028 ->1052 0.11513

1029 ->1061 0.10854

Excited State 29: Singlet-A 4.5126 eV 274.75 nm f=0.0003

<S**2>=0.000

1009 ->1035 -0.10680

1016 ->1041 0.16243

1025 ->1048 -0.21246

1026 ->1046 0.17787

1026 ->1048 -0.14736

1026 ->1050 -0.10207

1027 ->1046 0.22877

1027 ->1047 0.11246

1027 ->1049 -0.13249

1028 ->1045 -0.13069

1028 ->1048 -0.10105

1028 ->1052 -0.10738

1029 ->1061 0.13842

Excited State 30: Singlet-A 4.5152 eV 274.59 nm f=0.0160

<S**2>=0.000

1008 ->1035 -0.11274

1015 ->1040 0.12818

1017 ->1041 -0.16380

1019 ->1042 -0.11705

1021 ->1045 -0.13297

1023 ->1045 -0.11131

1024 ->1043 -0.12703

1025 ->1045 -0.11750

1026 ->1045 0.37069

1027 ->1045 -0.14953

Excited State 31: Singlet-A 4.5165 eV 274.51 nm f=0.0079

<S**2>=0.000

1006 ->1031 0.11351

1006 ->1032 -0.11870

1007 ->1034 0.10140

1007 ->1035 0.25092

1008 ->1032 0.11657

1008 ->1035 -0.14799

1008 ->1036 0.13149

1010 ->1039 -0.12509

1017 ->1041 0.19071

1018 ->1042 0.17018

Excited State 32: Singlet-A 4.5179 eV 274.43 nm f=0.0002

<S**2>=0.000

1007 ->1034 -0.12123

1008 ->1033 0.13387

1008 ->1034 0.22889

1008 ->1035 0.11564

1008 ->1036 0.13991

1008 ->1037 -0.10186

1009 ->1038 -0.15193

1011 ->1037 0.11824

1011 ->1038 0.17510

1016 ->1041 -0.17212

1017 ->1041 0.14721

Excited State 33: Singlet-A 4.5200 eV 274.30 nm f=0.0110

<S**2>=0.000

1007 ->1033 0.11119

1010 ->1038 0.10675

1010 ->1039 -0.17839

1011 ->1039 0.18069

1018 ->1042 -0.14369

1024 ->1043 0.18723

1026 ->1046 0.11459

1026 ->1050 -0.10278

1027 ->1046 0.15710

1027 ->1051 0.12999

Excited State 34: Singlet-A 4.5219 eV 274.19 nm f=0.0072

<S**2>=0.000

1008 ->1035 0.18149

1009 ->1037 0.10430

1015 ->1040 0.17665

1017 ->1041 0.11183

1018 ->1042 -0.18805

1025 ->1044 0.19532

1026 ->1044 0.12972

1029 ->1061 0.14550

1029 ->1062 -0.10426

Excited State 35: Singlet-A 4.5247 eV 274.02 nm f=0.0014

<S**2>=0.000

1007 ->1032 -0.13228

1008 ->1033 -0.12948

1008 ->1034 -0.12638

1009 ->1037 0.17753

1009 ->1038 -0.10591

1010 ->1038 0.22139

1010 ->1039 -0.18412

1011 ->1039 0.12104

1024 ->1043 -0.16818

1025 ->1044 -0.10289

Excited State 36: Singlet-A 4.5261 eV 273.93 nm f=0.0018

<S**2>=0.000

1008 ->1034 -0.13628

1008 ->1035 0.25547

1008 ->1037 0.13594

1009 ->1037 0.12926

1010 ->1038 0.16761

1011 ->1039 -0.18911

1015 ->1040 0.16926

1018 ->1042 0.11141

1024 ->1043 0.16395

Excited State 37: Singlet-A 4.5290 eV 273.76 nm f=0.0125

<S**2>=0.000

1017 ->1041 -0.12699

1018 ->1042 0.13915

1019 ->1044 -0.10753

1020 ->1044 -0.14478

1022 ->1044 0.11361

1023 ->1044 -0.11848

1025 ->1044 0.30916

1026 ->1044 0.29831

Excited State 38: Singlet-A 4.5296 eV 273.72 nm f=0.0038

<S**2>=0.000

1004 ->1030 0.11206

1007 ->1033 0.10835

1007 ->1034 -0.11226

1008 ->1035 -0.19042

1015 ->1040 -0.10851

1017 ->1041 -0.16167

1018 ->1042 0.21164

1024 ->1043 0.13577

1026 ->1043 -0.11921

1029 ->1060 -0.10304

Excited State 39: Singlet-A 4.5320 eV 273.58 nm f=0.0049

<S**2>=0.000

1005 ->1030 0.16057

1007 ->1034 0.11009

1007 ->1035 -0.28554

1008 ->1036 0.30994

1008 ->1037 -0.13758

1009 ->1037 -0.14054

1015 ->1040 0.16405

1018 ->1042 0.12444

Excited State 40: Singlet-A 4.5348 eV 273.41 nm f=0.0032

<S**2>=0.000

1008 ->1036 -0.10259

1009 ->1037 0.11215

1014 ->1040 0.15281

1018 ->1042 0.24287

1024 ->1043 -0.12205

1025 ->1049 -0.11001

1029 ->1061 0.16423

1029 ->1063 0.11034

Excited State 41: Singlet-A 4.5379 eV 273.22 nm f=0.0059

<S**2>=0.000

1007 ->1033 0.12229

1008 ->1036 0.31990

1015 ->1040 -0.17914

1017 ->1041 -0.13529

1018 ->1042 -0.13900

1025 ->1048 0.10191

1029 ->1061 0.18186

Excited State 42: Singlet-A 4.5396 eV 273.12 nm f=0.0129

<S**2>=0.000

1004 ->1038 -0.11143

1007 ->1034 -0.10117

1018 ->1042 0.22430

1026 ->1051 0.12500

1028 ->1051 -0.11837

1028 ->1053 -0.12560

1028 ->1054 0.10477

1028 ->1058 0.14509

1029 ->1061 -0.12230

1029 ->1063 -0.13094

1029 ->1064 0.12455

1029 ->1066 0.12583

Excited State 43: Singlet-A 4.5456 eV 272.75 nm f=0.0052

<S**2>=0.000

1006 ->1030 0.18473

1007 ->1031 -0.10406

1007 ->1034 -0.17899

1007 ->1036 -0.10116

1007 ->1037 -0.17750

1008 ->1036 0.15946

1009 ->1039 -0.18090

1010 ->1038 -0.11527

1015 ->1040 0.11424

1023 ->1044 -0.10429

1025 ->1044 -0.15486

1029 ->1061 -0.10905

Excited State 44: Singlet-A 4.5472 eV 272.66 nm f=0.0095

<S**2>=0.000

1026 ->1053 0.11367

1027 ->1055 -0.10232

1028 ->1049 0.11133

1028 ->1059 0.21613

1029 ->1061 0.18602

1029 ->1063 -0.11582

1029 ->1066 0.17185

Excited State 45: Singlet-A 4.5503 eV 272.47 nm f=0.0060

<S**2>=0.000

1023 ->1044 0.15920

1024 ->1046 0.18777

1025 ->1044 0.21383

1027 ->1053 -0.11737

1029 ->1061 -0.11146

1029 ->1062 0.24267

1029 ->1064 -0.11228

Excited State 46: Singlet-A 4.5511 eV 272.43 nm f=0.0074

<S**2>=0.000

1002 ->1030 0.12191

1023 ->1044 0.15255

1025 ->1044 0.10509

1025 ->1048 -0.10285

1026 ->1048 -0.11005

1026 ->1053 -0.13149

1027 ->1048 0.10156

1027 ->1051 -0.10605

1028 ->1051 0.13347

1029 ->1063 -0.13278

1029 ->1066 0.11757

Excited State 47: Singlet-A 4.5525 eV 272.34 nm f=0.0017

<S**2>=0.000

1005 ->1030 -0.23405

1007 ->1035 -0.13933

1009 ->1038 -0.12452

1009 ->1039 0.14340

1014 ->1040 0.25725

1015 ->1041 -0.15779

1024 ->1046 -0.16010

Excited State 48: Singlet-A 4.5546 eV 272.22 nm f=0.0057

<S**2>=0.000

1005 ->1031 -0.10191

1009 ->1039 0.20259

1022 ->1043 0.17118

1024 ->1046 0.22957

1025 ->1044 -0.11425

1025 ->1046 0.11092

1028 ->1059 -0.10399

Excited State 49: Singlet-A 4.5568 eV 272.09 nm f=0.0038

<S**2>=0.000

1005 ->1030 -0.20084

1014 ->1040 -0.17037

1015 ->1040 0.12355

1015 ->1041 -0.12052

1022 ->1043 -0.11430

1023 ->1044 -0.11143

1025 ->1044 -0.10189

1029 ->1060 -0.10154

1029 ->1061 0.11973

1029 ->1062 0.26357

1029 ->1064 -0.17709

Excited State 50: Singlet-A 4.5573 eV 272.06 nm f=0.0085

<S**2>=0.000

1006 ->1030 -0.13375

1006 ->1034 0.10033

1007 ->1036 -0.10308

1007 ->1037 0.10849

1009 ->1039 -0.16619

1014 ->1040 0.24670

1023 ->1044 -0.17242

1025 ->1044 -0.12285

1029 ->1063 -0.19482

1029 ->1066 0.10543

Excited State 51: Singlet-A 4.5607 eV 271.86 nm f=0.0026

<S**2>=0.000

1005 ->1030 0.23115

1006 ->1030 0.16290

1014 ->1040 0.17382

1023 ->1044 -0.13432

1024 ->1046 -0.14032

1025 ->1044 -0.10530

1026 ->1051 -0.10596

1029 ->1062 0.15052

Excited State 52: Singlet-A 4.5626 eV 271.74 nm f=0.0098

<S**2>=0.000

1002 ->1030 0.12584

1005 ->1031 0.14996

1007 ->1036 -0.10787

1014 ->1040 0.11228

1022 ->1043 0.13825

1028 ->1053 -0.11158

1028 ->1058 0.10241

1028 ->1059 -0.10429

1029 ->1062 0.12376

Excited State 53: Singlet-A 4.5646 eV 271.62 nm f=0.0060

<S**2>=0.000

1005 ->1030 -0.14738

1005 ->1031 0.15054

1006 ->1032 -0.10888

1007 ->1036 -0.14263

1008 ->1037 0.22567

1009 ->1037 -0.14767

1009 ->1038 -0.10247

1009 ->1039 0.11273

1011 ->1038 0.10028

1026 ->1056 -0.10610

1029 ->1066 -0.10861

Excited State 54: Singlet-A 4.5673 eV 271.46 nm f=0.0239

<S**2>=0.000

1008 ->1037 -0.20090

1022 ->1043 0.29479

1029 ->1063 0.10544

Excited State 55: Singlet-A 4.5699 eV 271.31 nm f=0.0230

<S**2>=0.000

1007 ->1036 -0.10349

1008 ->1037 0.12766

1009 ->1037 -0.11297

1009 ->1038 -0.10224

1015 ->1040 0.12863

1023 ->1044 0.17517

1024 ->1046 -0.11360

1025 ->1044 0.11619

1027 ->1048 -0.12629

1028 ->1060 -0.12241

Excited State 56: Singlet-A 4.5725 eV 271.15 nm f=0.0031

<S**2>=0.000

1008 ->1037 -0.10580

1009 ->1039 0.10974

1022 ->1043 -0.11633

1024 ->1043 -0.11011

1024 ->1046 0.14266

1027 ->1054 -0.10491

1027 ->1055 0.21817

1028 ->1048 -0.14275

1028 ->1053 -0.14316

1028 ->1055 -0.11642

1029 ->1062 0.11602

1029 ->1065 0.12406

Excited State 57: Singlet-A 4.5767 eV 270.90 nm f=0.0014

<S**2>=0.000

1005 ->1031 -0.19154

1006 ->1030 -0.10869

1007 ->1035 -0.13790

1007 ->1036 0.12238

1008 ->1037 0.27038

1009 ->1039 -0.19138

1010 ->1038 -0.13125

1011 ->1037 0.10685

1014 ->1040 0.11115

1017 ->1042 -0.13973

Excited State 58: Singlet-A 4.5785 eV 270.79 nm f=0.0108

<S**2>=0.000

1005 ->1030 0.10910

1009 ->1037 0.22491

1009 ->1038 0.21707

1009 ->1039 0.21634

1016 ->1042 -0.13704

1029 ->1060 0.15204

1029 ->1064 -0.13336

1029 ->1065 -0.15142

Excited State 59: Singlet-A 4.5798 eV 270.72 nm f=0.0061

<S**2>=0.000

1008 ->1037 0.11144

1009 ->1037 0.13448

1009 ->1038 0.13287

1009 ->1039 0.15991

1017 ->1042 0.24773

1022 ->1043 0.22913

1025 ->1051 -0.12248

1029 ->1063 -0.10307

Excited State 60: Singlet-A 4.5850 eV 270.42 nm f=0.0008

<S**2>=0.000

1025 ->1046 -0.13664

1025 ->1049 0.10350

1025 ->1051 0.12704

1027 ->1047 -0.10053

1028 ->1053 0.15147

1028 ->1055 0.14821

1029 ->1060 -0.19974

1029 ->1063 0.21193

1029 ->1065 0.21849

1029 ->1067 0.15185

Excited State 61: Singlet-A 4.5860 eV 270.35 nm f=0.0070

<S**2>=0.000

1007 ->1036 -0.15459

1009 ->1037 0.13675

1009 ->1039 0.10513

1022 ->1043 0.10730

1024 ->1046 -0.10214

1025 ->1046 0.11441

1029 ->1060 -0.12836

1029 ->1063 0.12611

1029 ->1064 0.15633

1029 ->1065 0.17894

1029 ->1069 0.14285

Excited State 62: Singlet-A 4.5868 eV 270.31 nm f=0.0105

<S**2>=0.000

1009 ->1039 0.11262

1015 ->1041 -0.11255

1022 ->1043 -0.14230

1026 ->1051 -0.12749

1026 ->1053 -0.10129

1027 ->1053 -0.10280

1027 ->1055 -0.12139

1027 ->1056 0.25183

1029 ->1062 -0.14807

Excited State 63: Singlet-A 4.5888 eV 270.19 nm f=0.0223

<S**2>=0.000

1005 ->1031 0.11208

1007 ->1036 0.15904

1014 ->1040 0.13162

1022 ->1043 -0.12411

1026 ->1053 -0.11452

1027 ->1054 0.10384

1027 ->1055 -0.13804

1028 ->1048 0.11874

1029 ->1062 -0.12407

1029 ->1063 0.16188

Excited State 64: Singlet-A 4.5904 eV 270.10 nm f=0.0164

<S**2>=0.000

1006 ->1031 0.12328

1007 ->1036 0.17340

1017 ->1042 0.17949

1025 ->1051 0.14645

1025 ->1053 0.10211

1028 ->1048 -0.12503

1028 ->1051 0.10264

1028 ->1054 -0.12898

1029 ->1065 0.10456

1029 ->1067 -0.11577

1029 ->1069 0.10016

Excited State 65: Singlet-A 4.5913 eV 270.04 nm f=0.0142

<S**2>=0.000

1005 ->1030 -0.12077

1015 ->1041 0.20229

1016 ->1042 0.26685

1027 ->1055 -0.11571

1029 ->1066 0.10406

1029 ->1067 0.17401

1029 ->1068 0.15374

1029 ->1070 -0.14879

Excited State 66: Singlet-A 4.5953 eV 269.81 nm f=0.0014

<S**2>=0.000

1005 ->1031 0.13016

1007 ->1036 0.16633

1007 ->1037 0.10012

1015 ->1041 -0.10724

1016 ->1042 0.11631

1027 ->1056 0.13197

1029 ->1062 0.12397

1029 ->1064 0.23763

1029 ->1065 -0.14141

1029 ->1068 -0.14758

Excited State 67: Singlet-A 4.5983 eV 269.63 nm f=0.0133

<S**2>=0.000

1005 ->1031 -0.17184

1014 ->1040 0.13731

1015 ->1041 0.15749

1017 ->1042 0.18425

1022 ->1043 -0.13265

1023 ->1046 -0.13091

1025 ->1046 -0.15060

1027 ->1053 -0.10737

1029 ->1064 0.15713

1029 ->1065 -0.10027

Excited State 68: Singlet-A 4.6020 eV 269.41 nm f=0.0003

<S**2>=0.000

1017 ->1042 0.23575

1023 ->1049 -0.11118

1025 ->1048 -0.11342

1026 ->1048 -0.10556

1026 ->1051 0.10350

1026 ->1054 -0.10610

1028 ->1048 0.17568

1028 ->1050 0.13831

1028 ->1060 -0.10566

1029 ->1062 -0.11618

1029 ->1066 0.11259

Excited State 69: Singlet-A 4.6031 eV 269.35 nm f=0.0062

<S**2>=0.000

1005 ->1032 0.14115

1005 ->1033 0.11004

1006 ->1030 0.13105

1007 ->1037 0.18717

1008 ->1038 0.10494

1016 ->1042 0.11426

1027 ->1056 -0.14672

1029 ->1067 -0.17686

Excited State 70: Singlet-A 4.6055 eV 269.21 nm f=0.0045

<S**2>=0.000

1006 ->1030 -0.11191

1007 ->1037 -0.12551

1022 ->1046 0.11044

1023 ->1046 0.20403

1024 ->1047 0.20285

1025 ->1046 -0.13106

1027 ->1052 0.10738

1028 ->1048 0.16900

1029 ->1067 -0.15364

Excited State 71: Singlet-A 4.6075 eV 269.09 nm f=0.0060

<S**2>=0.000

1005 ->1031 0.10676

1005 ->1032 0.10103

1006 ->1031 -0.12060

1024 ->1046 -0.10032

1024 ->1047 0.13680

1025 ->1046 0.20023

1028 ->1047 -0.10077

Excited State 72: Singlet-A 4.6086 eV 269.03 nm f=0.0013

<S**2>=0.000

1007 ->1037 0.22395

1013 ->1040 0.10401

1015 ->1041 -0.10644

1016 ->1042 -0.13260

1025 ->1046 -0.11981

1025 ->1051 -0.13972

1025 ->1053 0.14339

1026 ->1051 -0.10478

1029 ->1067 0.18279

Excited State 73: Singlet-A 4.6091 eV 269.00 nm f=0.0031

<S**2>=0.000

1002 ->1036 0.14253

1006 ->1031 0.10836

1024 ->1046 -0.13365

1025 ->1046 0.10414

1027 ->1056 0.10079

Excited State 74: Singlet-A 4.6112 eV 268.88 nm f=0.0017

<S**2>=0.000

1007 ->1037 -0.11680

1014 ->1041 0.12121

1015 ->1041 0.10034

1023 ->1046 0.22632

1025 ->1046 0.19614

1026 ->1046 0.10608

1029 ->1064 0.10158

Excited State 75: Singlet-A 4.6124 eV 268.81 nm f=0.0015

<S**2>=0.000

1015 ->1041 0.14969

1016 ->1042 0.11890

1027 ->1056 0.23273

1028 ->1056 -0.10401

1029 ->1062 -0.13604

1029 ->1064 -0.18890

1029 ->1067 -0.16108

Excited State 76: Singlet-A 4.6157 eV 268.61 nm f=0.0074

<S**2>=0.000

1006 ->1030 -0.10382

1007 ->1037 -0.17122

1008 ->1038 0.24025

1009 ->1038 -0.12814

1010 ->1038 0.10191

1017 ->1042 0.11486

1023 ->1046 -0.11504

1025 ->1046 0.11245

1028 ->1059 -0.10142

Excited State 77: Singlet-A 4.6173 eV 268.52 nm f=0.0174

<S**2>=0.000

1005 ->1031 -0.11423

1007 ->1036 -0.13725

1007 ->1037 0.16001

1008 ->1038 0.19671

1013 ->1040 -0.15577

1014 ->1041 -0.10178

1015 ->1041 -0.13059

1023 ->1046 0.13982

1028 ->1059 0.10268

1029 ->1064 -0.11290

Excited State 78: Singlet-A 4.6191 eV 268.42 nm f=0.0051

<S**2>=0.000

1005 ->1031 -0.16124

1005 ->1032 -0.12214

1006 ->1031 0.16543

1006 ->1034 -0.12800

1006 ->1035 0.13421

1007 ->1036 -0.13349

1008 ->1038 -0.12798

1013 ->1040 0.10610

1025 ->1046 0.11626

Excited State 79: Singlet-A 4.6217 eV 268.27 nm f=0.0005

<S**2>=0.000

1005 ->1033 -0.10255

1005 ->1035 -0.11312

1008 ->1038 0.23659

1008 ->1039 0.10192

1014 ->1041 0.15057

1017 ->1042 -0.15197

1024 ->1047 -0.12019

1028 ->1048 -0.11327

Excited State 80: Singlet-A 4.6233 eV 268.17 nm f=0.0026

<S**2>=0.000

1003 ->1030 0.11373

1014 ->1041 0.11861

1023 ->1047 0.10582

1026 ->1054 0.10004

1027 ->1058 0.12208

Excited State 81: Singlet-A 4.6241 eV 268.13 nm f=0.0007

<S**2>=0.000

1008 ->1038 0.15588

1025 ->1048 -0.13054

1025 ->1051 0.13910

1026 ->1052 -0.18041

1027 ->1051 0.11026

1027 ->1052 0.11453

1027 ->1055 0.11504

1028 ->1047 0.10617

1028 ->1048 0.21576

1028 ->1049 -0.10346

1028 ->1060 0.15427

Excited State 82: Singlet-A 4.6256 eV 268.04 nm f=0.0075

<S**2>=0.000

1008 ->1038 0.14894

1016 ->1042 -0.13566

1021 ->1045 0.12368

1023 ->1045 0.23819

1025 ->1045 -0.17828

1029 ->1066 0.10844

Excited State 83: Singlet-A 4.6298 eV 267.80 nm f=0.0128

<S**2>=0.000

1016 ->1042 0.13260

1021 ->1045 0.16521

1022 ->1045 -0.11601

1023 ->1045 0.30919

1024 ->1045 0.11107

1025 ->1045 -0.22723

1025 ->1046 0.11337

1026 ->1045 0.10958

Excited State 84: Singlet-A 4.6322 eV 267.66 nm f=0.0007

<S**2>=0.000

1008 ->1038 -0.12005

1008 ->1039 -0.13785

1019 ->1044 -0.17591

1020 ->1044 -0.20834

1022 ->1044 0.21941

1023 ->1044 0.26125

1025 ->1044 -0.15539

Excited State 85: Singlet-A 4.6339 eV 267.56 nm f=0.0075

<S**2>=0.000

1002 ->1030 -0.12137

1005 ->1035 -0.12879

1006 ->1032 0.10861

1007 ->1035 0.11547

1008 ->1039 0.10013

1022 ->1044 0.11964

1023 ->1044 0.15267

1023 ->1046 -0.13271

1024 ->1047 0.10343

1027 ->1047 0.10245

Excited State 86: Singlet-A 4.6363 eV 267.42 nm f=0.0003

<S**2>=0.000

1005 ->1033 -0.10921

1013 ->1040 -0.10877

1028 ->1048 -0.12901

1029 ->1062 0.14980

1029 ->1063 0.23053

1029 ->1065 -0.12129

1029 ->1066 0.31386

1029 ->1067 -0.12387

Excited State 87: Singlet-A 4.6375 eV 267.35 nm f=0.0145

<S**2>=0.000

1006 ->1030 -0.10961

1007 ->1037 -0.15478

1008 ->1039 0.31314

1013 ->1040 0.15595

1015 ->1041 -0.14632

1016 ->1042 0.10176

1017 ->1042 0.12483

1029 ->1063 0.10219

1029 ->1066 0.14815

Excited State 88: Singlet-A 4.6398 eV 267.22 nm f=0.0071

<S**2>=0.000

1005 ->1033 0.19511

1006 ->1032 -0.10450

1007 ->1035 -0.12592

1007 ->1038 0.11681

1012 ->1040 -0.10032

1028 ->1049 0.11274

1028 ->1050 0.13512

1029 ->1069 0.10568

Excited State 89: Singlet-A 4.6422 eV 267.08 nm f=0.0071

<S**2>=0.000

1008 ->1039 -0.23475

1029 ->1063 0.14513

1029 ->1064 -0.12188

1029 ->1066 0.24331

1029 ->1068 -0.14595

Excited State 90: Singlet-A 4.6452 eV 266.91 nm f=0.0134

<S**2>=0.000

1008 ->1039 0.21507

1013 ->1040 -0.12111

1029 ->1064 -0.15712

1029 ->1065 -0.12500

1029 ->1067 0.28190

1029 ->1068 -0.14228

1029 ->1069 0.26768

Excited State 91: Singlet-A 4.6470 eV 266.80 nm f=0.0035

<S**2>=0.000

1003 ->1030 0.13522

1005 ->1034 -0.12657

1008 ->1038 -0.14326

1008 ->1039 -0.12125

1014 ->1041 0.14981

1015 ->1041 -0.12557

1016 ->1042 0.10100

1027 ->1047 0.15512

1027 ->1055 0.11171

1027 ->1057 -0.13983

Excited State 92: Singlet-A 4.6491 eV 266.68 nm f=0.0009

<S**2>=0.000

1014 ->1041 0.19977

1022 ->1047 0.12101

1026 ->1047 -0.11508

1027 ->1047 -0.15821

1027 ->1048 0.11306

1027 ->1051 0.14046

1027 ->1055 -0.13019

1027 ->1057 0.13667

Excited State 93: Singlet-A 4.6499 eV 266.64 nm f=0.0131

<S**2>=0.000

1012 ->1040 0.16029

1023 ->1043 -0.11932

1024 ->1047 0.12365

1024 ->1049 0.12370

1024 ->1050 -0.10662

1027 ->1047 -0.14299

1027 ->1048 0.10289

1027 ->1051 0.10493

1029 ->1068 0.16185

Excited State 94: Singlet-A 4.6526 eV 266.48 nm f=0.0053

<S**2>=0.000

1008 ->1039 -0.20587

1013 ->1040 0.26486

1025 ->1047 0.10247

1026 ->1049 0.12779

1029 ->1067 0.13390

1029 ->1068 -0.15033

Excited State 95: Singlet-A 4.6538 eV 266.41 nm f=0.0017

<S**2>=0.000

1007 ->1038 -0.12878

1012 ->1040 0.16243

1013 ->1040 0.10992

1023 ->1046 0.17685

1024 ->1046 -0.10859

1024 ->1047 -0.14336

1024 ->1050 0.11176

1029 ->1068 0.11300

1029 ->1069 0.16857

Excited State 96: Singlet-A 4.6555 eV 266.32 nm f=0.0017

<S**2>=0.000

1025 ->1046 -0.11348

1025 ->1049 -0.10246

1025 ->1053 -0.12873

1026 ->1050 0.13211

1026 ->1057 0.12789

1028 ->1054 -0.13295

1028 ->1055 -0.10441

1028 ->1057 0.16720

1029 ->1067 0.10302

1029 ->1068 -0.16043

Excited State 97: Singlet-A 4.6559 eV 266.29 nm f=0.0024

<S**2>=0.000

1003 ->1030 0.11324

1005 ->1030 -0.13213

1005 ->1031 0.12147

1005 ->1033 0.13113

1006 ->1031 0.20077

1006 ->1035 -0.19154

1013 ->1040 -0.13750

1014 ->1041 -0.11857

1015 ->1041 0.13756

1027 ->1049 0.10812

Excited State 98: Singlet-A 4.6586 eV 266.14 nm f=0.0015

<S**2>=0.000

1004 ->1031 -0.12590

1004 ->1038 0.13097

1005 ->1035 -0.10235

1007 ->1038 0.20886

1026 ->1047 0.11146

1026 ->1056 -0.13964

1027 ->1052 -0.10376

1028 ->1050 -0.10953

1029 ->1069 0.17287

1029 ->1070 -0.13216

Excited State 99: Singlet-A 4.6629 eV 265.90 nm f=0.0042

<S**2>=0.000

1005 ->1033 -0.11195

1005 ->1034 0.17131

1006 ->1030 0.12088

1006 ->1034 0.20316

1007 ->1038 0.13032

1014 ->1041 0.17362

1027 ->1049 0.12785

1027 ->1050 -0.11442

Excited State 100: Singlet-A 4.6642 eV 265.82 nm f=0.0214

<S**2>=0.000

1005 ->1032 0.10511

1006 ->1033 -0.13980

1006 ->1034 -0.15937

1007 ->1038 -0.12838

1012 ->1040 -0.10478

1014 ->1041 0.11662

1023 ->1046 -0.12087

1029 ->1068 0.20386

1029 ->1069 0.23018

Excited State 101: Singlet-A 4.6652 eV 265.76 nm f=0.0054

<S**2>=0.000

1005 ->1035 0.10526

1006 ->1034 0.13167

1014 ->1041 0.16338

1023 ->1049 0.10285

1025 ->1049 0.10451

1029 ->1068 0.11003

Excited State 102: Singlet-A 4.6654 eV 265.75 nm f=0.0024

<S**2>=0.000

1005 ->1032 -0.10761

1006 ->1031 -0.10732

1007 ->1038 0.15448

1014 ->1041 -0.15356

1029 ->1068 0.20838

1029 ->1069 0.22074

1029 ->1070 0.11152

Excited State 103: Singlet-A 4.6678 eV 265.62 nm f=0.0167

<S**2>=0.000

1006 ->1031 -0.11898

1013 ->1040 -0.12771

1021 ->1045 0.10220

1022 ->1045 0.10418

1023 ->1047 0.12867

1023 ->1049 0.21224

1024 ->1050 0.13565

1029 ->1070 -0.13476

Excited State 104: Singlet-A 4.6716 eV 265.40 nm f=0.0055

<S**2>=0.000

1015 ->1042 0.11828

1016 ->1042 0.10607

1026 ->1056 0.10719

1026 ->1063 -0.10950

1026 ->1066 0.13194

1027 ->1062 0.10145

1028 ->1063 0.10406

1028 ->1066 -0.14474

1029 ->1068 0.15067

1029 ->1070 0.18944

Excited State 105: Singlet-A 4.6732 eV 265.31 nm f=0.0125

<S**2>=0.000

1006 ->1034 0.15884

1007 ->1038 -0.12218

1013 ->1040 -0.12703

1021 ->1045 0.10546

1022 ->1047 -0.14310

1026 ->1058 0.10856

1028 ->1056 -0.12329

1028 ->1057 -0.14955

Excited State 106: Singlet-A 4.6756 eV 265.18 nm f=0.0063

<S**2>=0.000

1005 ->1032 -0.15661

1005 ->1034 -0.11007

1007 ->1038 0.15314

1007 ->1039 -0.10819

1026 ->1053 -0.10096

1029 ->1070 0.27167

Excited State 107: Singlet-A 4.6776 eV 265.06 nm f=0.0020

<S**2>=0.000

1003 ->1030 -0.16309

1005 ->1032 -0.15152

1005 ->1033 0.18729

1005 ->1034 -0.18245

1006 ->1033 0.14652

1012 ->1040 -0.12355

Excited State 108: Singlet-A 4.6787 eV 265.00 nm f=0.0061

<S**2>=0.000

1005 ->1032 0.17620

1005 ->1034 0.13489

1006 ->1033 0.11265

1006 ->1035 0.10149

1007 ->1039 0.16757

1024 ->1051 -0.10512

1029 ->1070 0.27861

Excited State 109: Singlet-A 4.6795 eV 264.95 nm f=0.0148

<S**2>=0.000

1005 ->1032 -0.15000

1015 ->1042 0.11822

1018 ->1043 0.15091

1023 ->1043 0.15773

1023 ->1046 -0.12592

1026 ->1060 0.13254

1028 ->1053 0.10298

Excited State 110: Singlet-A 4.6813 eV 264.85 nm f=0.0011

<S**2>=0.000

1003 ->1030 -0.12028

1004 ->1038 -0.10345

1005 ->1033 0.15940

1006 ->1030 -0.10872

1006 ->1033 0.23134

1007 ->1039 0.12522

1012 ->1040 -0.12420

Excited State 111: Singlet-A 4.6849 eV 264.65 nm f=0.0063

<S**2>=0.000

1007 ->1038 -0.13983

1015 ->1042 0.12485

1018 ->1043 0.18739

1022 ->1047 0.10624

1023 ->1043 0.16335

Excited State 112: Singlet-A 4.6865 eV 264.56 nm f=0.0010

<S**2>=0.000

1005 ->1032 0.11840

1006 ->1031 0.16225

1006 ->1032 0.12583

1006 ->1034 -0.15819

1007 ->1038 0.25875

1007 ->1039 0.14938

1012 ->1040 0.17628

1018 ->1043 0.10177

1026 ->1047 -0.10021

1027 ->1050 0.10541

Excited State 113: Singlet-A 4.6885 eV 264.44 nm f=0.0034

<S**2>=0.000

1005 ->1035 -0.12585

1013 ->1041 0.14486

1021 ->1045 0.13326

1022 ->1045 0.14850

1026 ->1053 0.10306

1029 ->1070 0.18030

Excited State 114: Singlet-A 4.6911 eV 264.30 nm f=0.0030

<S**2>=0.000

1005 ->1031 -0.10592

1005 ->1032 -0.11108

1005 ->1034 -0.10531

1006 ->1033 -0.10242

1007 ->1039 0.38405

1009 ->1039 0.11004

1015 ->1042 -0.15545

Excited State 115: Singlet-A 4.6929 eV 264.20 nm f=0.0091

<S**2>=0.000

1005 ->1035 0.14801

1006 ->1032 0.10353

1007 ->1039 0.20235

1024 ->1051 0.17425

1027 ->1054 0.11736

Excited State 116: Singlet-A 4.6953 eV 264.06 nm f=0.0004

<S**2>=0.000

1005 ->1031 -0.14980

1005 ->1034 -0.10702

1005 ->1035 -0.11743

1006 ->1032 -0.18189

1006 ->1033 -0.12714

1007 ->1039 0.19622

1013 ->1041 -0.14595

1015 ->1042 0.12645

1024 ->1044 0.12556

1027 ->1051 0.14512

Excited State 117: Singlet-A 4.6967 eV 263.98 nm f=0.0031

<S**2>=0.000

1013 ->1041 0.16329

1015 ->1042 -0.16153

1024 ->1044 0.20340

1027 ->1050 0.12367

1027 ->1051 0.16978

1027 ->1052 -0.12480

1028 ->1048 0.10582

Excited State 118: Singlet-A 4.6979 eV 263.92 nm f=0.0102

<S**2>=0.000

1012 ->1040 -0.13581

1019 ->1046 0.11982

1020 ->1046 -0.13440

1024 ->1044 0.16578

1026 ->1060 -0.11843

1028 ->1054 0.10227

1028 ->1057 -0.14217

Excited State 119: Singlet-A 4.6985 eV 263.88 nm f=0.0114

<S**2>=0.000

1015 ->1042 0.23204

1024 ->1044 0.19316

1027 ->1050 -0.11255

1027 ->1051 -0.12172

1027 ->1052 0.10629

1027 ->1054 0.11478

1027 ->1059 0.10527

Excited State 120: Singlet-A 4.7006 eV 263.76 nm f=0.0058

<S**2>=0.000

1005 ->1035 -0.10082

1015 ->1042 -0.12156

1024 ->1044 0.20477

Excited State 121: Singlet-A 4.7041 eV 263.56 nm f=0.0020

<S**2>=0.000

1002 ->1039 -0.10748

1004 ->1030 -0.10345

1023 ->1043 -0.10268

1025 ->1048 -0.13791

1026 ->1048 0.22935

1027 ->1051 0.14745

1027 ->1054 0.14109

1028 ->1054 0.15210

1028 ->1055 -0.10950

Excited State 122: Singlet-A 4.7043 eV 263.56 nm f=0.0069

<S**2>=0.000

1020 ->1046 0.11547

1023 ->1043 -0.13499

1023 ->1044 -0.10438

1024 ->1044 0.28565

1025 ->1048 0.11868

1026 ->1048 -0.14806

1027 ->1062 0.14357

Excited State 123: Singlet-A 4.7071 eV 263.40 nm f=0.0003

<S**2>=0.000

1013 ->1041 -0.12031

1015 ->1042 0.13885

1024 ->1044 0.17969

1025 ->1048 -0.15066

1026 ->1048 0.15773

1026 ->1051 -0.10490

1027 ->1052 -0.16149

1027 ->1062 -0.14393

Excited State 124: Singlet-A 4.7073 eV 263.39 nm f=0.0068

<S**2>=0.000

1013 ->1041 0.16656

1024 ->1044 0.19029

1025 ->1048 -0.11446

1026 ->1048 0.17643

Excited State 125: Singlet-A 4.7097 eV 263.25 nm f=0.0029

<S**2>=0.000

1005 ->1034 -0.14223

1006 ->1032 0.15098

1025 ->1049 -0.14614

1025 ->1054 0.11604

1026 ->1058 0.14307

1027 ->1051 -0.10020

1028 ->1053 0.12483

Excited State 126: Singlet-A 4.7110 eV 263.18 nm f=0.0095

<S**2>=0.000

1012 ->1041 0.10396

1013 ->1041 0.23436

1015 ->1042 0.10566

1025 ->1048 -0.11505

1026 ->1048 0.14060

1027 ->1053 0.10407

1027 ->1054 -0.11171

1028 ->1051 -0.12549

1029 ->1071 0.17138

1029 ->1073 -0.12109

Excited State 127: Singlet-A 4.7128 eV 263.08 nm f=0.0038

<S**2>=0.000

1002 ->1031 -0.11247

1002 ->1033 0.10019

1002 ->1039 0.14204

1003 ->1039 0.10792

1015 ->1042 -0.10397

1025 ->1054 -0.12113

1025 ->1057 0.13422

1028 ->1061 -0.13814

Excited State 128: Singlet-A 4.7151 eV 262.95 nm f=0.0056

<S**2>=0.000

1006 ->1032 0.15250

1013 ->1041 0.22769

Excited State 129: Singlet-A 4.7160 eV 262.90 nm f=0.0040

<S**2>=0.000

1005 ->1032 -0.16933

1005 ->1033 0.10981

1005 ->1034 0.19502

1005 ->1035 -0.15593

1005 ->1037 0.18870

1014 ->1042 -0.10593

1023 ->1043 -0.11427

1026 ->1051 0.12627

Excited State 130: Singlet-A 4.7190 eV 262.73 nm f=0.0160

<S**2>=0.000

1014 ->1042 0.10517

1021 ->1045 0.12879

1022 ->1045 0.16298

1023 ->1051 -0.12548

1026 ->1048 -0.11287

Excited State 131: Singlet-A 4.7208 eV 262.63 nm f=0.0078

<S**2>=0.000

1003 ->1030 -0.11365

1005 ->1034 0.15514

1005 ->1035 -0.13258

1005 ->1037 0.20498

1018 ->1043 -0.11915

1022 ->1047 -0.10108

1023 ->1043 0.12352

1029 ->1071 0.14082

Excited State 132: Singlet-A 4.7219 eV 262.57 nm f=0.0132

<S**2>=0.000

1004 ->1035 -0.10693

1021 ->1045 0.11463

1022 ->1045 0.14132

1022 ->1047 0.10683

1028 ->1060 0.15610

1029 ->1071 0.23761

1029 ->1072 -0.10215

1029 ->1073 -0.18182

Excited State 133: Singlet-A 4.7254 eV 262.38 nm f=0.0016

<S**2>=0.000

1003 ->1031 -0.11187

1004 ->1030 -0.17001

1004 ->1031 0.18104

1004 ->1032 0.27363

1005 ->1033 0.11756

1005 ->1034 0.12261

1005 ->1035 -0.10576

1006 ->1037 -0.12012

1029 ->1071 0.11228

Excited State 134: Singlet-A 4.7261 eV 262.34 nm f=0.0065

<S**2>=0.000

1018 ->1043 0.18472

1023 ->1043 -0.15326

1025 ->1049 -0.11398

1025 ->1051 0.15574

1029 ->1071 0.14065

Excited State 135: Singlet-A 4.7270 eV 262.29 nm f=0.0067

<S**2>=0.000

1006 ->1032 0.12829

1014 ->1042 0.19561

1025 ->1057 0.13733

1028 ->1056 0.10179

1028 ->1057 -0.12218

1028 ->1060 -0.12200

Excited State 136: Singlet-A 4.7281 eV 262.23 nm f=0.0031

<S**2>=0.000

1005 ->1037 0.13438

1014 ->1042 0.13151

1019 ->1046 -0.13440

1020 ->1046 0.11974

1024 ->1052 -0.12731

1025 ->1051 0.10423

1026 ->1051 -0.12524

1028 ->1054 0.12280

Excited State 137: Singlet-A 4.7287 eV 262.20 nm f=0.0165

<S**2>=0.000

1001 ->1030 0.10538

1005 ->1034 0.10066

1005 ->1037 0.11528

1012 ->1041 0.11584

1014 ->1042 0.18155

1020 ->1046 -0.10195

1028 ->1060 0.10412

Excited State 138: Singlet-A 4.7294 eV 262.16 nm f=0.0197

<S**2>=0.000

1014 ->1042 0.13442

1025 ->1057 0.10403

1025 ->1059 -0.11747

1026 ->1054 -0.16766

1026 ->1057 0.13224

1027 ->1052 -0.10662

1027 ->1055 0.10191

1027 ->1059 -0.13555

1028 ->1054 -0.10258

1028 ->1055 0.17020

1029 ->1071 -0.10606

1029 ->1073 0.10295

Excited State 139: Singlet-A 4.7311 eV 262.06 nm f=0.0018

<S**2>=0.000

1012 ->1041 0.15674

1013 ->1042 0.10245

1014 ->1042 0.15270

1015 ->1042 -0.12558

1018 ->1043 -0.14019

1023 ->1043 0.11457

1025 ->1055 -0.10056

Excited State 140: Singlet-A 4.7336 eV 261.93 nm f=0.0359

<S**2>=0.000

1014 ->1042 -0.15635

1019 ->1046 -0.11284

1024 ->1045 0.18563

1028 ->1056 -0.10187

Excited State 141: Singlet-A 4.7342 eV 261.89 nm f=0.0066

<S**2>=0.000

1022 ->1045 0.16504

1024 ->1051 -0.11721

1024 ->1053 0.12536

1026 ->1051 -0.10970

1028 ->1055 -0.10714

Excited State 142: Singlet-A 4.7361 eV 261.79 nm f=0.0101

<S**2>=0.000

1005 ->1037 -0.11009

1006 ->1037 -0.14993

1010 ->1040 0.11930

1011 ->1040 0.13015

1014 ->1042 0.14431

1018 ->1043 -0.10962

1023 ->1043 0.10147

1027 ->1052 0.19115

1027 ->1053 0.10998

1028 ->1056 -0.10932

Excited State 143: Singlet-A 4.7377 eV 261.70 nm f=0.0025

<S**2>=0.000

1006 ->1037 0.22521

1023 ->1043 -0.10192

1024 ->1045 0.21440

1026 ->1051 0.10128

1026 ->1052 0.13968

1027 ->1052 0.13757

Excited State 144: Singlet-A 4.7397 eV 261.59 nm f=0.0093

<S**2>=0.000

1000 ->1030 0.10530

1004 ->1031 -0.10580

1004 ->1032 -0.12827

1006 ->1037 -0.15560

1024 ->1045 0.17893

1026 ->1052 0.15178

1027 ->1054 0.10787

Excited State 145: Singlet-A 4.7413 eV 261.50 nm f=0.0021

<S**2>=0.000

1001 ->1031 -0.12138

1012 ->1041 -0.14870

1022 ->1048 0.10101

1026 ->1052 0.14863

1027 ->1054 0.15276

1027 ->1065 -0.11485

Excited State 146: Singlet-A 4.7428 eV 261.42 nm f=0.0047

<S**2>=0.000

1001 ->1031 0.14272

1005 ->1033 -0.12398

1005 ->1037 0.16065

1006 ->1037 -0.12480

1024 ->1045 -0.14389

1026 ->1052 0.19200

1026 ->1054 0.13383

1027 ->1052 0.21301

Excited State 147: Singlet-A 4.7448 eV 261.30 nm f=0.0141

<S**2>=0.000

1010 ->1040 0.14430

1011 ->1040 0.15292

1012 ->1041 0.20413

1014 ->1042 -0.12086

1015 ->1042 0.10778

1023 ->1043 0.10209

1028 ->1056 0.10228

1028 ->1065 -0.13349

Excited State 148: Singlet-A 4.7449 eV 261.30 nm f=0.0085

<S**2>=0.000

1005 ->1033 -0.10236

1005 ->1037 0.13434

1012 ->1041 -0.10797

1024 ->1045 0.35708

Excited State 149: Singlet-A 4.7493 eV 261.06 nm f=0.0014

<S**2>=0.000

1005 ->1035 0.10370

1006 ->1036 -0.10607

1006 ->1037 -0.13921

1024 ->1045 -0.13755

1025 ->1058 0.12474

1026 ->1054 -0.12574

1027 ->1058 -0.12423

1027 ->1061 0.15023

1028 ->1061 -0.12012

Excited State 150: Singlet-A 4.7497 eV 261.04 nm f=0.0081

<S**2>=0.000

1001 ->1031 -0.10326

1001 ->1035 0.10730

1003 ->1031 -0.10183

1005 ->1035 0.11263

1012 ->1041 0.12124

1022 ->1048 -0.12358

1024 ->1045 0.19337

1026 ->1054 0.13818

Excited State 151: Singlet-A 4.7513 eV 260.95 nm f=0.0211

<S**2>=0.000

1014 ->1042 -0.10037

1018 ->1043 0.10169

1025 ->1049 0.12253

1025 ->1054 0.12204

1025 ->1060 0.10087

Excited State 152: Singlet-A 4.7547 eV 260.76 nm f=0.0025

<S**2>=0.000

1005 ->1036 0.27060

1005 ->1037 0.14504

1005 ->1038 -0.13760

1006 ->1036 0.15846

1006 ->1038 -0.10276

Excited State 153: Singlet-A 4.7554 eV 260.72 nm f=0.0043

<S**2>=0.000

1003 ->1030 0.12143

1003 ->1031 -0.10404

1026 ->1055 0.15221

1028 ->1055 -0.10405

Excited State 154: Singlet-A 4.7571 eV 260.63 nm f=0.0169

<S**2>=0.000

1022 ->1048 -0.11293

1026 ->1052 0.11805

1026 ->1065 0.11042

1027 ->1058 0.10241

Excited State 155: Singlet-A 4.7593 eV 260.51 nm f=0.0031

<S**2>=0.000

1001 ->1031 0.10299

1024 ->1052 0.16090

1025 ->1050 -0.12889

1025 ->1059 -0.10108

1026 ->1052 -0.12892

1026 ->1055 0.15463

1026 ->1059 -0.13973

1027 ->1061 0.13004

1028 ->1058 -0.10488

Excited State 156: Singlet-A 4.7601 eV 260.47 nm f=0.0051

<S**2>=0.000

1005 ->1036 -0.12240

1006 ->1037 0.10357

1024 ->1052 0.13443

1025 ->1052 -0.11215

1026 ->1053 0.10882

1026 ->1054 -0.11232

1028 ->1059 -0.10049

Excited State 157: Singlet-A 4.7612 eV 260.40 nm f=0.0130

<S**2>=0.000

987 ->1034 -0.10246

991 ->1030 0.11717

991 ->1034 -0.10471

995 ->1037 0.11954

996 ->1037 -0.11382

997 ->1030 -0.11890

997 ->1034 0.15174

997 ->1037 0.10065

1025 ->1058 0.10133

Excited State 158: Singlet-A 4.7640 eV 260.25 nm f=0.0067

<S**2>=0.000

1005 ->1037 0.16958

1006 ->1036 -0.12853

1011 ->1040 0.11565

1024 ->1052 0.10763

1025 ->1049 0.13313

1025 ->1050 0.20597

Excited State 159: Singlet-A 4.7669 eV 260.10 nm f=0.0096

<S**2>=0.000

1003 ->1031 -0.11517

1005 ->1036 -0.24411

1005 ->1037 0.24567

1006 ->1036 -0.12818

1006 ->1037 0.13902

1010 ->1040 0.10029

Excited State 160: Singlet-A 4.7684 eV 260.01 nm f=0.0011

<S**2>=0.000

996 ->1030 -0.11369

999 ->1030 0.10090

1000 ->1031 -0.10946

1003 ->1032 -0.14015

1005 ->1036 0.19173

1006 ->1031 -0.11147

1006 ->1035 -0.12096

1022 ->1044 0.13031

1025 ->1050 -0.12275

Excited State 161: Singlet-A 4.7688 eV 259.99 nm f=0.0004

<S**2>=0.000

1003 ->1031 0.14881

1005 ->1038 0.12018

1006 ->1032 0.11911

1006 ->1036 -0.11146

1026 ->1059 0.10406

1028 ->1065 0.10395

Excited State 162: Singlet-A 4.7702 eV 259.91 nm f=0.0025

<S**2>=0.000

1012 ->1041 -0.13630

1022 ->1048 -0.11755

1025 ->1050 0.15736

1025 ->1052 -0.10260

1026 ->1054 -0.10240

1029 ->1073 -0.13639

1029 ->1074 0.15412

1029 ->1075 0.11963

Excited State 163: Singlet-A 4.7713 eV 259.86 nm f=0.0147

<S**2>=0.000

1025 ->1054 0.11984

1026 ->1055 0.10115

1026 ->1059 -0.10700

1028 ->1052 0.11235

1029 ->1073 -0.11646

1029 ->1074 0.16386

1029 ->1075 0.13162

Excited State 164: Singlet-A 4.7719 eV 259.82 nm f=0.0041

<S**2>=0.000

1012 ->1041 -0.10373

1022 ->1044 -0.13225

1025 ->1050 -0.14407

1026 ->1056 0.10990

1028 ->1056 0.22832

1028 ->1057 -0.12406

Excited State 165: Singlet-A 4.7744 eV 259.69 nm f=0.0011

<S**2>=0.000

1022 ->1044 0.23191

1024 ->1059 0.10473

1025 ->1052 -0.12856

1026 ->1059 -0.12243

1027 ->1059 0.11480

Excited State 166: Singlet-A 4.7758 eV 259.61 nm f=0.0023

<S**2>=0.000

1000 ->1031 -0.10124

1003 ->1033 0.11557

1003 ->1034 0.22144

1012 ->1041 0.10695

1027 ->1058 -0.10197

1027 ->1063 -0.11112

1029 ->1073 0.10308

1029 ->1074 -0.11655

Excited State 167: Singlet-A 4.7787 eV 259.45 nm f=0.0023

<S**2>=0.000

1001 ->1030 0.10047

1003 ->1034 0.12116

1003 ->1035 -0.10157

1005 ->1036 -0.11365

1006 ->1035 0.14668

1026 ->1053 0.11127

1026 ->1058 0.10098

1027 ->1058 0.11834

1027 ->1062 0.10801

1027 ->1063 0.22165

Excited State 168: Singlet-A 4.7792 eV 259.42 nm f=0.0038

<S**2>=0.000

1025 ->1050 0.15196

1025 ->1051 -0.10232

1026 ->1054 0.11151

1026 ->1055 -0.11132

1028 ->1057 0.10200

Excited State 169: Singlet-A 4.7814 eV 259.31 nm f=0.0023

<S**2>=0.000

1005 ->1038 0.17182

1012 ->1041 0.11077

1025 ->1055 -0.10961

1026 ->1055 0.11487

1026 ->1059 0.11339

1027 ->1053 -0.10863

Excited State 170: Singlet-A 4.7827 eV 259.23 nm f=0.0134

<S**2>=0.000

1021 ->1044 -0.11253

1021 ->1046 -0.21152

1022 ->1044 0.25570

1022 ->1046 0.11758

1022 ->1047 0.10832

1026 ->1056 0.15701

Excited State 171: Singlet-A 4.7832 eV 259.21 nm f=0.0004

<S**2>=0.000

1021 ->1046 0.11988

1022 ->1044 0.24704

1022 ->1046 -0.11997

1025 ->1050 0.14052

1025 ->1052 0.12162

1025 ->1054 0.10205

1026 ->1052 0.10335

1028 ->1052 0.10242

1028 ->1056 0.16283

Excited State 172: Singlet-A 4.7852 eV 259.10 nm f=0.0053

<S**2>=0.000

1003 ->1031 0.12127

1005 ->1036 -0.11956

1005 ->1038 -0.15180

1005 ->1039 0.10083

1006 ->1038 -0.10797

1025 ->1055 -0.12771

1026 ->1053 0.10833

1026 ->1055 0.17756

Excited State 173: Singlet-A 4.7861 eV 259.05 nm f=0.0010

<S**2>=0.000

1005 ->1038 -0.14190

1025 ->1053 0.11089

1026 ->1053 -0.10443

1026 ->1058 0.11163

1027 ->1058 0.11201

1027 ->1059 0.22435

1027 ->1061 0.10974

1027 ->1062 0.12653

1027 ->1063 0.11308

1027 ->1064 0.15710

Excited State 174: Singlet-A 4.7875 eV 258.98 nm f=0.0010

<S**2>=0.000

999 ->1030 0.10115

1005 ->1038 0.26772

1006 ->1038 0.14336

1013 ->1042 -0.10713

1025 ->1052 -0.13924

Excited State 175: Singlet-A 4.7895 eV 258.87 nm f=0.0036

<S**2>=0.000

999 ->1031 0.10474

1022 ->1047 -0.10634

1026 ->1053 -0.10419

1027 ->1060 -0.11302

1027 ->1062 0.13700

1027 ->1066 0.11642

1029 ->1074 -0.10829

Excited State 176: Singlet-A 4.7904 eV 258.82 nm f=0.0056

<S**2>=0.000

995 ->1030 -0.12617

1003 ->1031 0.11218

1022 ->1044 0.14306

1022 ->1045 -0.12280

Excited State 177: Singlet-A 4.7917 eV 258.75 nm f=0.0045

<S**2>=0.000

1012 ->1041 0.13651

1022 ->1049 0.11872

1027 ->1057 0.10457

1027 ->1064 -0.10598

Excited State 178: Singlet-A 4.7925 eV 258.71 nm f=0.0021

<S**2>=0.000

1013 ->1042 0.13538

1022 ->1045 0.17885

1022 ->1046 -0.13607

1023 ->1045 0.19517

1026 ->1056 0.11943

1026 ->1057 -0.12493

1029 ->1072 0.11056

Excited State 179: Singlet-A 4.7939 eV 258.63 nm f=0.0057

<S**2>=0.000

1013 ->1042 0.22668

1021 ->1046 -0.19265

1022 ->1045 0.10186

1022 ->1046 0.16369

1023 ->1046 -0.10778

1025 ->1052 0.11921

1029 ->1073 0.12201

Excited State 180: Singlet-A 4.7951 eV 258.56 nm f=0.0013

<S**2>=0.000

1029 ->1071 0.23167

1029 ->1072 0.39999

1029 ->1075 0.12402

Excited State 181: Singlet-A 4.7975 eV 258.43 nm f=0.0019

<S**2>=0.000

999 ->1031 -0.10532

1004 ->1032 0.10003

1008 ->1040 0.13017

1009 ->1040 0.12505

1010 ->1040 -0.12398

1013 ->1042 -0.10584

1022 ->1045 0.14672

1023 ->1045 0.12167

1029 ->1072 -0.11693

Excited State 182: Singlet-A 4.7980 eV 258.41 nm f=0.0010

<S**2>=0.000

993 ->1030 -0.10304

993 ->1031 0.10668

994 ->1030 0.12704

999 ->1030 0.10071

1003 ->1031 0.10926

1004 ->1032 0.13060

1006 ->1035 -0.10295

1013 ->1042 0.11022

1022 ->1046 -0.11543

1027 ->1057 0.10267

1029 ->1072 -0.10607

Excited State 183: Singlet-A 4.7991 eV 258.35 nm f=0.0038

<S**2>=0.000

993 ->1030 -0.13782

993 ->1031 0.14667

993 ->1032 0.10226

1005 ->1039 0.13353

1010 ->1040 0.10244

1025 ->1050 0.16022

1025 ->1052 0.21284

Excited State 184: Singlet-A 4.8000 eV 258.30 nm f=0.0091

<S**2>=0.000

997 ->1030 -0.13963

997 ->1032 -0.13419

997 ->1033 -0.11233

997 ->1035 0.20475

1006 ->1035 0.12015

1022 ->1045 0.14514

1023 ->1045 0.16518

Excited State 185: Singlet-A 4.8009 eV 258.25 nm f=0.0132

<S**2>=0.000

1005 ->1038 0.10591

1006 ->1036 0.12230

1023 ->1045 -0.12690

1025 ->1052 0.19348

1025 ->1054 0.12754

1026 ->1056 -0.10427

1028 ->1056 -0.13521

1029 ->1072 -0.12262

Excited State 186: Singlet-A 4.8021 eV 258.19 nm f=0.0022

<S**2>=0.000

1000 ->1033 0.11846

1003 ->1033 0.15059

1005 ->1039 0.12203

1025 ->1052 0.10898

1027 ->1063 0.11789

1027 ->1066 0.10873

1029 ->1073 0.10340

1029 ->1075 0.12949

Excited State 187: Singlet-A 4.8030 eV 258.14 nm f=0.0004

<S**2>=0.000

993 ->1030 0.10698

994 ->1030 -0.11396

997 ->1031 -0.10473

999 ->1031 0.10673

1002 ->1031 -0.13662

1002 ->1032 0.16031

1002 ->1033 -0.16214

1002 ->1034 -0.15598

1003 ->1033 -0.11833

1005 ->1039 0.18745

Excited State 188: Singlet-A 4.8036 eV 258.11 nm f=0.0077

<S**2>=0.000

1003 ->1033 0.10424

1005 ->1038 0.10935

1005 ->1039 0.21804

1006 ->1036 0.13298

1011 ->1040 0.12449

1027 ->1057 0.11734

1029 ->1072 0.13033

Excited State 189: Singlet-A 4.8046 eV 258.05 nm f=0.0239

<S**2>=0.000

1005 ->1039 0.19207

1013 ->1042 0.13861

1025 ->1052 -0.13052

1029 ->1073 0.11795

Excited State 190: Singlet-A 4.8056 eV 258.00 nm f=0.0011

<S**2>=0.000

1027 ->1057 0.15545

1027 ->1063 0.19882

1027 ->1066 0.13725

1029 ->1072 0.24850

Excited State 191: Singlet-A 4.8080 eV 257.87 nm f=0.0066

<S**2>=0.000

1010 ->1040 0.10200

1013 ->1042 0.11812

1025 ->1052 -0.13434

1026 ->1061 0.10319

1027 ->1057 -0.12745

1027 ->1065 -0.10712

1029 ->1072 0.22361

1029 ->1073 -0.14538

Excited State 192: Singlet-A 4.8094 eV 257.79 nm f=0.0048

<S**2>=0.000

1006 ->1036 0.16310

1008 ->1040 -0.13935

1009 ->1040 -0.12247

1013 ->1042 -0.11357

1025 ->1055 -0.10299

1027 ->1066 -0.12381

1029 ->1071 0.12308

1029 ->1073 0.11559

Excited State 193: Singlet-A 4.8112 eV 257.70 nm f=0.0039

<S**2>=0.000

1003 ->1033 -0.11444

1006 ->1036 0.11239

1025 ->1059 0.11468

1027 ->1057 0.11918

Excited State 194: Singlet-A 4.8125 eV 257.63 nm f=0.0008

<S**2>=0.000

1001 ->1030 -0.10483

1006 ->1036 -0.11360

1020 ->1043 -0.11507

1024 ->1055 -0.14546

1024 ->1057 -0.10985

1025 ->1052 -0.10795

1029 ->1075 0.15643

1029 ->1076 0.13020

Excited State 195: Singlet-A 4.8134 eV 257.58 nm f=0.0110

<S**2>=0.000

1006 ->1036 -0.19878

1013 ->1042 0.10105

1029 ->1071 0.15917

1029 ->1073 0.20343

1029 ->1074 0.27949

1029 ->1076 -0.20941

Excited State 196: Singlet-A 4.8150 eV 257.49 nm f=0.0024

<S**2>=0.000

992 ->1030 -0.11917

1005 ->1039 0.14408

1026 ->1056 -0.13961

1027 ->1057 -0.13162

1027 ->1066 0.13389

1029 ->1073 0.11938

Excited State 197: Singlet-A 4.8154 eV 257.47 nm f=0.0013

<S**2>=0.000

1001 ->1030 -0.11961

1006 ->1036 0.15222

1027 ->1064 0.11858

1027 ->1065 -0.10473

1028 ->1069 0.11035

1029 ->1074 0.12252

Excited State 198: Singlet-A 4.8160 eV 257.44 nm f=0.0036

<S**2>=0.000

1005 ->1036 -0.13069

1006 ->1036 0.20962

1029 ->1074 0.13976

1029 ->1076 -0.11405

Excited State 199: Singlet-A 4.8170 eV 257.39 nm f=0.0077

<S**2>=0.000

999 ->1031 0.09802

1001 ->1030 0.10304

1003 ->1031 -0.10174

1008 ->1040 0.10391

1023 ->1050 0.09701

1029 ->1073 0.10507

1029 ->1074 0.10444

Excited State 200: Singlet-A 4.8197 eV 257.25 nm f=0.0057

<S**2>=0.000

1003 ->1033 0.10831

1005 ->1039 -0.15357

1006 ->1036 0.10865

1023 ->1050 -0.11122

1027 ->1066 0.15585

1027 ->1068 -0.15136

Excited State 201: Singlet-A 4.8209 eV 257.18 nm f=0.0000

<S**2>=0.000

1005 ->1039 0.11596

1025 ->1054 0.14370

1025 ->1055 -0.10756

1027 ->1064 -0.12573

1027 ->1066 0.10855

1027 ->1068 -0.10386

Excited State 202: Singlet-A 4.8237 eV 257.03 nm f=0.0045

<S**2>=0.000

1001 ->1033 -0.10048

1027 ->1064 0.10663

1027 ->1066 -0.15729

1027 ->1068 0.20524

1028 ->1069 0.10451

Excited State 203: Singlet-A 4.8243 eV 257.00 nm f=0.0013

<S**2>=0.000

1022 ->1048 0.14103

1025 ->1057 -0.12118

1026 ->1064 -0.12004

1027 ->1058 0.10519

1027 ->1059 -0.10796

1027 ->1064 -0.14008

Excited State 204: Singlet-A 4.8256 eV 256.93 nm f=0.0021

<S**2>=0.000

999 ->1032 -0.10796

1003 ->1032 0.14548

1020 ->1043 0.17669

1021 ->1043 -0.11171

1024 ->1055 -0.13069

Excited State 205: Singlet-A 4.8283 eV 256.79 nm f=0.0010

<S**2>=0.000

999 ->1030 0.10182

1005 ->1039 -0.11664

1011 ->1041 -0.10050

1020 ->1043 0.17297

1021 ->1043 -0.10458

Excited State 206: Singlet-A 4.8289 eV 256.76 nm f=0.0084

<S**2>=0.000

994 ->1031 -0.11217

998 ->1032 0.13028

1003 ->1032 0.22093

1020 ->1043 -0.17428

1021 ->1043 0.13281

Excited State 207: Singlet-A 4.8308 eV 256.65 nm f=0.0060

<S**2>=0.000

1000 ->1035 0.11424

1019 ->1043 -0.12112

1020 ->1043 -0.10253

1026 ->1056 -0.10151

1027 ->1068 0.12243

Excited State 208: Singlet-A 4.8321 eV 256.59 nm f=0.0023

<S**2>=0.000

1019 ->1043 -0.10189

1023 ->1050 -0.12769

1025 ->1059 0.10624

1028 ->1061 -0.10682

1028 ->1062 0.13848

1028 ->1069 -0.11979

Excited State 209: Singlet-A 4.8337 eV 256.50 nm f=0.0051

<S**2>=0.000

1003 ->1032 0.11006

1019 ->1043 0.11686

1020 ->1043 0.16151

1021 ->1043 -0.12072

1028 ->1064 -0.10545

1028 ->1065 0.10269

Excited State 210: Singlet-A 4.8344 eV 256.46 nm f=0.0018

<S**2>=0.000

992 ->1030 0.12711

998 ->1031 -0.12103

1003 ->1031 -0.10441

1004 ->1036 0.10673

1008 ->1040 -0.11158

1012 ->1042 0.11074

Excited State 211: Singlet-A 4.8347 eV 256.45 nm f=0.0010

<S**2>=0.000

1011 ->1040 -0.10131

1025 ->1055 -0.10757

1027 ->1057 0.12343

1028 ->1061 0.13520

Excited State 212: Singlet-A 4.8364 eV 256.36 nm f=0.0059

<S**2>=0.000

1006 ->1038 -0.12275

1023 ->1050 0.11528

1024 ->1060 0.10001

1027 ->1064 0.11040

1029 ->1074 0.12877

Excited State 213: Singlet-A 4.8373 eV 256.31 nm f=0.0010

<S**2>=0.000

998 ->1031 0.14425

1009 ->1040 0.10603

1023 ->1050 0.10570

1027 ->1064 -0.13154

1028 ->1062 -0.12415

Excited State 214: Singlet-A 4.8383 eV 256.25 nm f=0.0131

<S**2>=0.000

1005 ->1039 -0.13457

1006 ->1039 0.18910

1025 ->1051 -0.10615

1025 ->1056 -0.13234

1027 ->1068 0.10205

Excited State 215: Singlet-A 4.8407 eV 256.13 nm f=0.0074

<S**2>=0.000

998 ->1031 -0.11779

1005 ->1039 -0.12121

1006 ->1038 0.15541

1006 ->1039 0.15132

1008 ->1040 0.10171

1025 ->1056 0.13669

1027 ->1060 -0.10611

Excited State 216: Singlet-A 4.8420 eV 256.06 nm f=0.0029

<S**2>=0.000

995 ->1031 0.10860

995 ->1032 -0.10416

998 ->1033 0.10718

1004 ->1031 0.14457

1004 ->1033 0.19125

1004 ->1036 -0.11383

1010 ->1040 -0.10345

1012 ->1042 0.10149

Excited State 217: Singlet-A 4.8439 eV 255.96 nm f=0.0012

<S**2>=0.000

1004 ->1031 0.10236

1004 ->1033 0.19367

1006 ->1038 0.16788

1027 ->1068 -0.10281

Excited State 218: Singlet-A 4.8449 eV 255.91 nm f=0.0059

<S**2>=0.000

1019 ->1043 0.12821

1021 ->1044 0.15718

1026 ->1064 -0.10601

1027 ->1058 -0.12068

1027 ->1059 0.13420

1027 ->1065 0.11433

1028 ->1062 0.10945

Excited State 219: Singlet-A 4.8464 eV 255.82 nm f=0.0019

<S**2>=0.000

1009 ->1040 0.15051

1010 ->1041 -0.11131

1025 ->1053 -0.12107

1025 ->1056 -0.10925

1028 ->1061 -0.10283

1028 ->1065 0.10838

1028 ->1070 0.11426

Excited State 220: Singlet-A 4.8483 eV 255.73 nm f=0.0027

<S**2>=0.000

990 ->1035 0.11919

992 ->1030 0.12068

996 ->1031 0.12761

996 ->1032 -0.13758

996 ->1035 0.11987

997 ->1032 -0.12049

1000 ->1035 -0.11122

1001 ->1032 0.14212

1003 ->1037 -0.10731

Excited State 221: Singlet-A 4.8493 eV 255.67 nm f=0.0116

<S**2>=0.000

986 ->1030 0.11834

1002 ->1030 -0.11297

1021 ->1044 0.14991

1023 ->1049 0.10904

1023 ->1050 -0.12511

1023 ->1051 -0.14919

1025 ->1056 -0.10779

1026 ->1059 -0.11600

1028 ->1062 -0.11689

Excited State 222: Singlet-A 4.8508 eV 255.60 nm f=0.0034

<S**2>=0.000

1020 ->1048 0.10024

1021 ->1044 0.16688

1024 ->1048 0.13677

1026 ->1057 -0.11004

1027 ->1068 0.11436

Excited State 223: Singlet-A 4.8510 eV 255.58 nm f=0.0010

<S**2>=0.000

986 ->1030 -0.12873

1006 ->1038 0.11326

1006 ->1039 0.12784

1019 ->1043 -0.13761

1021 ->1044 0.18144

1028 ->1063 -0.11269

1029 ->1075 0.10058

Excited State 224: Singlet-A 4.8519 eV 255.54 nm f=0.0280

<S**2>=0.000

990 ->1030 -0.11300

990 ->1032 -0.10145

990 ->1035 0.10748

1017 ->1043 -0.10089

1019 ->1043 0.12409

Excited State 225: Singlet-A 4.8529 eV 255.49 nm f=0.0068

<S**2>=0.000

1006 ->1039 0.11170

1021 ->1044 0.16610

1027 ->1066 -0.13798

1028 ->1063 -0.13300

Excited State 226: Singlet-A 4.8536 eV 255.45 nm f=0.0180

<S**2>=0.000

986 ->1030 -0.10628

990 ->1032 0.11043

990 ->1035 -0.14254

1006 ->1038 0.18626

1006 ->1039 0.11251

1012 ->1042 0.14726

1019 ->1043 0.12081

Excited State 227: Singlet-A 4.8539 eV 255.43 nm f=0.0021

<S**2>=0.000

1021 ->1044 0.17410

1021 ->1046 -0.13214

1022 ->1046 -0.10535

1025 ->1061 -0.10807

1027 ->1064 -0.10766

1029 ->1076 0.18777

Excited State 228: Singlet-A 4.8563 eV 255.31 nm f=0.0165

<S**2>=0.000

1019 ->1043 0.10530

1021 ->1044 -0.16795

1025 ->1058 0.13556

1028 ->1071 -0.10060

1029 ->1077 0.14978

1029 ->1078 0.20914

1029 ->1079 0.13983

1029 ->1082 0.10612

Excited State 229: Singlet-A 4.8571 eV 255.27 nm f=0.0025

<S**2>=0.000

1021 ->1044 -0.22393

1026 ->1057 -0.12270

1027 ->1060 0.10718

1029 ->1074 0.12004

1029 ->1075 -0.18075

1029 ->1076 0.22949

Excited State 230: Singlet-A 4.8583 eV 255.20 nm f=0.0097

<S**2>=0.000

1019 ->1043 0.13574

1021 ->1044 0.15445

1024 ->1048 0.12123

1028 ->1063 0.16215

Excited State 231: Singlet-A 4.8593 eV 255.15 nm f=0.0058

<S**2>=0.000

1006 ->1038 0.11280

1024 ->1048 0.10914

1028 ->1070 -0.10794

1029 ->1074 0.10435

1029 ->1075 -0.16437

1029 ->1076 0.20552

Excited State 232: Singlet-A 4.8603 eV 255.10 nm f=0.0007

<S**2>=0.000

1006 ->1039 0.19448

1008 ->1040 -0.15925

1010 ->1041 -0.14449

1027 ->1059 0.11033

1028 ->1063 0.12240

Excited State 233: Singlet-A 4.8614 eV 255.04 nm f=0.0075

<S**2>=0.000

1006 ->1039 0.17753

1021 ->1044 -0.11732

1024 ->1048 0.17748

1028 ->1063 -0.10997

1028 ->1070 -0.15472

1029 ->1074 -0.11294

1029 ->1075 0.10419

1029 ->1076 -0.14897

Excited State 234: Singlet-A 4.8633 eV 254.94 nm f=0.0074

<S**2>=0.000

1003 ->1036 0.11020

1009 ->1040 0.10641

1029 ->1078 0.14657

1029 ->1079 0.12205

Excited State 235: Singlet-A 4.8636 eV 254.93 nm f=0.0010

<S**2>=0.000

989 ->1032 -0.10465

1006 ->1038 0.10587

1006 ->1039 -0.18682

1029 ->1074 -0.11596

1029 ->1075 0.21694

1029 ->1076 -0.21538

Excited State 236: Singlet-A 4.8657 eV 254.81 nm f=0.0073

<S**2>=0.000

985 ->1030 -0.11885

989 ->1030 0.12594

1002 ->1030 0.11627

1003 ->1034 -0.11784

1008 ->1040 -0.10169

1009 ->1040 0.11219

1019 ->1043 0.12282

1021 ->1046 -0.10728

1029 ->1075 0.10827

Excited State 237: Singlet-A 4.8676 eV 254.71 nm f=0.0034

<S**2>=0.000

1004 ->1033 0.13552

1006 ->1039 0.10656

1023 ->1048 -0.11478

1024 ->1047 0.11317

1024 ->1048 0.16131

1025 ->1057 -0.11470

1026 ->1060 0.11356

Excited State 238: Singlet-A 4.8685 eV 254.66 nm f=0.0060

<S**2>=0.000

1017 ->1043 0.11977

1019 ->1043 -0.16631

1021 ->1046 0.12903

1023 ->1049 0.11745

1024 ->1048 0.17993

1024 ->1050 -0.10661

1028 ->1071 0.13697

Excited State 239: Singlet-A 4.8697 eV 254.60 nm f=0.0065

<S**2>=0.000

998 ->1031 0.14283

998 ->1033 -0.13528

1005 ->1038 -0.10296

1006 ->1038 0.14575

1006 ->1039 0.12632

1012 ->1042 0.14652

Excited State 240: Singlet-A 4.8703 eV 254.57 nm f=0.0012

<S**2>=0.000

1021 ->1046 0.11475

1023 ->1048 0.12039

Excited State 241: Singlet-A 4.8727 eV 254.45 nm f=0.0257

<S**2>=0.000

1002 ->1032 -0.10695

1004 ->1033 -0.12873

1008 ->1040 0.20211

1010 ->1040 0.14932

1012 ->1042 0.13032

Excited State 242: Singlet-A 4.8729 eV 254.44 nm f=0.0029

<S**2>=0.000

992 ->1031 -0.10544

1004 ->1032 0.10979

1004 ->1033 0.11273

1008 ->1040 0.10404

1022 ->1051 0.12631

1022 ->1053 0.10173

1028 ->1071 0.13978

Excited State 243: Singlet-A 4.8743 eV 254.36 nm f=0.0053

<S**2>=0.000

1020 ->1048 -0.10866

1023 ->1054 0.11163

1024 ->1048 0.12658

1028 ->1069 0.13749

1028 ->1070 0.10805

1028 ->1071 -0.10888

Excited State 244: Singlet-A 4.8761 eV 254.27 nm f=0.0016

<S**2>=0.000

1004 ->1034 0.13445

1011 ->1041 -0.11475

1023 ->1048 -0.11559

1024 ->1049 -0.11652

1024 ->1050 -0.12412

Excited State 245: Singlet-A 4.8773 eV 254.21 nm f=0.0026

<S**2>=0.000

1004 ->1034 0.12985

1025 ->1056 -0.10649

1025 ->1059 -0.14126

1027 ->1059 0.17752

1027 ->1060 -0.14398

1027 ->1064 -0.10963

1027 ->1068 0.10201

1028 ->1069 0.10371

Excited State 246: Singlet-A 4.8782 eV 254.16 nm f=0.0021

<S**2>=0.000

995 ->1031 -0.13146

999 ->1033 0.10317

1002 ->1031 0.14872

1003 ->1035 0.10379

1011 ->1041 -0.10029

1025 ->1059 -0.12270

Excited State 247: Singlet-A 4.8793 eV 254.10 nm f=0.0054

<S**2>=0.000

1003 ->1036 0.14592

1003 ->1037 -0.12396

1004 ->1032 -0.12255

1006 ->1039 0.11167

Excited State 248: Singlet-A 4.8807 eV 254.03 nm f=0.0001

<S**2>=0.000

1002 ->1033 0.10933

1004 ->1033 -0.12383

1004 ->1034 0.17099

1028 ->1071 0.10611

Excited State 249: Singlet-A 4.8816 eV 253.98 nm f=0.0031

<S**2>=0.000

1021 ->1046 -0.12368

1024 ->1048 0.16101

1025 ->1059 0.12600

1028 ->1062 0.11662

Excited State 250: Singlet-A 4.8825 eV 253.94 nm f=0.0028

<S**2>=0.000

1029 ->1075 -0.10828

1029 ->1077 0.19427

1029 ->1078 0.22280

1029 ->1079 -0.15148

1029 ->1084 0.10717

Excited State 251: Singlet-A 4.8837 eV 253.87 nm f=0.0075

<S**2>=0.000

999 ->1036 -0.10513

1003 ->1036 0.12029

1004 ->1034 0.24764

1029 ->1078 0.17172

1029 ->1082 -0.12128

Excited State 252: Singlet-A 4.8852 eV 253.79 nm f=0.0063

<S**2>=0.000

1021 ->1050 -0.10378

1022 ->1051 -0.11837

1023 ->1050 -0.10425

1023 ->1056 -0.14747

1024 ->1049 0.18679

1024 ->1050 0.24905

1024 ->1058 0.10244

Excited State 253: Singlet-A 4.8868 eV 253.71 nm f=0.0041

<S**2>=0.000

1004 ->1034 -0.13808

1012 ->1042 -0.11522

1025 ->1060 0.15839

1029 ->1078 0.11987

1029 ->1082 -0.14203

Excited State 254: Singlet-A 4.8878 eV 253.66 nm f=0.0020

<S**2>=0.000

1003 ->1035 0.20354

1009 ->1040 -0.15776

1010 ->1040 -0.10628

1010 ->1041 -0.13521

1011 ->1040 0.10002

1029 ->1077 0.10836

1029 ->1082 -0.12273

1029 ->1084 -0.10956

Excited State 255: Singlet-A 4.8885 eV 253.63 nm f=0.0188

<S**2>=0.000

1003 ->1035 0.10011

1004 ->1034 0.11873

1028 ->1072 -0.13002

1029 ->1079 -0.13492

1029 ->1082 0.18401

1029 ->1084 0.18433

Excited State 256: Singlet-A 4.8902 eV 253.54 nm f=0.0025

<S**2>=0.000

989 ->1030 0.10252

1002 ->1031 0.11693

1002 ->1032 0.22312

1004 ->1035 0.11254

1028 ->1073 0.10139

Excited State 257: Singlet-A 4.8919 eV 253.45 nm f=0.0083

<S**2>=0.000

1002 ->1032 -0.11217

1025 ->1059 0.12555

1028 ->1072 0.11162

1029 ->1077 -0.10765

Excited State 258: Singlet-A 4.8923 eV 253.43 nm f=0.0068

<S**2>=0.000

1002 ->1032 0.16486

1003 ->1035 -0.15025

1003 ->1037 -0.11341

1012 ->1042 0.10935

1029 ->1077 0.20971

1029 ->1078 -0.15282

Excited State 259: Singlet-A 4.8933 eV 253.37 nm f=0.0015

<S**2>=0.000

1003 ->1037 -0.11392

1027 ->1060 -0.12953

1029 ->1077 -0.22006

1029 ->1078 -0.11065

1029 ->1079 0.22805

1029 ->1082 0.15377

Excited State 260: Singlet-A 4.8943 eV 253.32 nm f=0.0014

<S**2>=0.000

983 ->1030 -0.11290

995 ->1031 0.10105

1001 ->1031 0.13159

1001 ->1034 0.11998

1003 ->1035 0.12947

1004 ->1034 0.11662

1008 ->1041 -0.11301

1025 ->1060 0.11993

1029 ->1078 -0.11264

Excited State 261: Singlet-A 4.8952 eV 253.28 nm f=0.0047

<S**2>=0.000

1018 ->1047 -0.12935

1021 ->1046 -0.10245

1026 ->1063 0.11553

1028 ->1066 -0.13472

1028 ->1072 0.12076

1029 ->1082 0.20165

1029 ->1084 0.13502

Excited State 262: Singlet-A 4.8965 eV 253.21 nm f=0.0031

<S**2>=0.000

1028 ->1072 0.13137

1029 ->1077 0.23997

1029 ->1078 -0.20041

Excited State 263: Singlet-A 4.8971 eV 253.18 nm f=0.0067

<S**2>=0.000

1025 ->1060 0.12544

1029 ->1077 0.33977

1029 ->1078 -0.26949

Excited State 264: Singlet-A 4.8994 eV 253.06 nm f=0.0028

<S**2>=0.000

1008 ->1041 -0.10285

1021 ->1043 -0.11557

1023 ->1058 -0.10960

1026 ->1063 -0.10899

1029 ->1079 0.12451

1029 ->1082 -0.11767

Excited State 265: Singlet-A 4.9003 eV 253.01 nm f=0.0049

<S**2>=0.000

1001 ->1031 0.15122

1001 ->1034 0.14878

1011 ->1041 0.14170

1026 ->1063 -0.11262

Excited State 266: Singlet-A 4.9008 eV 252.99 nm f=0.0048

<S**2>=0.000

1021 ->1043 -0.10172

1023 ->1052 -0.10292

1024 ->1049 -0.11427

1024 ->1050 -0.11155

1028 ->1063 -0.10738

1028 ->1066 0.12372

1028 ->1072 0.11412

1029 ->1082 0.12421

Excited State 267: Singlet-A 4.9025 eV 252.90 nm f=0.0038

<S**2>=0.000

1023 ->1056 0.10792

1026 ->1063 0.10737

1028 ->1067 0.10884

1028 ->1072 0.15202

1028 ->1073 -0.15022

Excited State 268: Singlet-A 4.9048 eV 252.78 nm f=0.0129

<S**2>=0.000

1022 ->1053 -0.14097

1025 ->1057 0.10797

1025 ->1062 0.15068

1025 ->1064 -0.10015

1027 ->1065 -0.10581

Excited State 269: Singlet-A 4.9058 eV 252.73 nm f=0.0017

<S**2>=0.000

995 ->1030 -0.10394

998 ->1030 -0.10890

1001 ->1034 -0.14189

1002 ->1031 0.15322

1022 ->1057 0.10339

Excited State 270: Singlet-A 4.9071 eV 252.66 nm f=0.0027

<S**2>=0.000

1002 ->1033 -0.14485

1004 ->1034 0.12425

1010 ->1041 -0.16390

1020 ->1043 0.16474

1021 ->1043 0.22731

Excited State 271: Singlet-A 4.9093 eV 252.55 nm f=0.0016

<S**2>=0.000

1004 ->1034 0.12053

1020 ->1043 0.10955

1020 ->1045 -0.12523

1021 ->1043 0.10869

1025 ->1056 0.13449

1025 ->1058 0.12582

1025 ->1060 0.11644

1025 ->1061 -0.10456

1025 ->1064 -0.11770

1025 ->1065 0.11998

1025 ->1068 -0.11574

1028 ->1068 -0.10754

Excited State 272: Singlet-A 4.9108 eV 252.47 nm f=0.0027

<S**2>=0.000

1010 ->1041 0.14365

1020 ->1043 0.16756

1021 ->1043 0.28466

Excited State 273: Singlet-A 4.9118 eV 252.42 nm f=0.0015

<S**2>=0.000

992 ->1031 0.12623

998 ->1030 -0.10039

1023 ->1047 -0.12199

1023 ->1052 0.10349

1029 ->1079 0.11615

Excited State 274: Singlet-A 4.9125 eV 252.38 nm f=0.0019

<S**2>=0.000

1002 ->1033 -0.19693

1002 ->1036 0.10134

1004 ->1034 0.27635

1004 ->1035 -0.11112

1020 ->1043 -0.14164

1021 ->1043 -0.18821

Excited State 275: Singlet-A 4.9132 eV 252.35 nm f=0.0023

<S**2>=0.000

993 ->1030 0.10951

998 ->1030 0.11965

1020 ->1045 0.13853

1023 ->1048 0.11945

1028 ->1064 -0.10186

1029 ->1079 -0.10212

Excited State 276: Singlet-A 4.9148 eV 252.27 nm f=0.0024

<S**2>=0.000

987 ->1031 -0.11353

994 ->1030 -0.10034

1019 ->1045 0.11402

1020 ->1045 0.17574

1023 ->1047 0.10661

Excited State 277: Singlet-A 4.9153 eV 252.24 nm f=0.0029

<S**2>=0.000

1008 ->1041 -0.10502

1027 ->1074 -0.11831

1027 ->1075 -0.11029

1029 ->1076 0.10218

1029 ->1079 -0.10388

1029 ->1081 -0.15457

1029 ->1083 0.16460

1029 ->1084 -0.12651

Excited State 278: Singlet-A 4.9171 eV 252.15 nm f=0.0063

<S**2>=0.000

1029 ->1079 0.15017

1029 ->1081 0.10431

1029 ->1083 -0.11026

1029 ->1084 0.12006

Excited State 279: Singlet-A 4.9181 eV 252.10 nm f=0.0018

<S**2>=0.000

983 ->1030 -0.10654

996 ->1034 -0.10293

1000 ->1030 0.10930

1004 ->1035 0.18779

1004 ->1036 0.10760

1020 ->1045 -0.10116

Excited State 280: Singlet-A 4.9196 eV 252.02 nm f=0.0025

<S**2>=0.000

993 ->1030 0.18950

993 ->1031 0.11880

993 ->1032 0.11491

993 ->1033 -0.10447

996 ->1030 -0.12751

996 ->1032 -0.11007

998 ->1030 0.14397

Excited State 281: Singlet-A 4.9207 eV 251.96 nm f=0.0027

<S**2>=0.000

989 ->1031 0.10130

992 ->1032 -0.11875

993 ->1032 0.10833

993 ->1038 0.11461

994 ->1031 0.12320

995 ->1038 -0.10822

Excited State 282: Singlet-A 4.9211 eV 251.94 nm f=0.0010

<S**2>=0.000

984 ->1031 -0.10237

993 ->1030 0.14378

1001 ->1032 0.10427

1001 ->1034 -0.10121

1004 ->1034 0.13881

1027 ->1060 -0.10701

Excited State 283: Singlet-A 4.9226 eV 251.87 nm f=0.0008

<S**2>=0.000

1020 ->1045 0.11237

1024 ->1054 -0.12900

1024 ->1057 0.13113

1027 ->1060 -0.11380

Excited State 284: Singlet-A 4.9238 eV 251.81 nm f=0.0012

<S**2>=0.000

990 ->1032 -0.10548

993 ->1030 -0.11284

993 ->1031 -0.10937

998 ->1033 0.10087

1001 ->1030 0.10850

1001 ->1032 0.17878

Excited State 285: Singlet-A 4.9244 eV 251.77 nm f=0.0010

<S**2>=0.000

986 ->1031 -0.11987

992 ->1030 -0.10061

992 ->1031 -0.10196

1000 ->1030 -0.10658

1002 ->1032 -0.13936

1002 ->1033 -0.11128

1002 ->1034 -0.10669

1002 ->1035 -0.12422

1004 ->1033 0.17028

1004 ->1035 0.23636

Excited State 286: Singlet-A 4.9257 eV 251.71 nm f=0.0056

<S**2>=0.000

1008 ->1041 0.10456

1010 ->1041 0.10432

1020 ->1044 -0.11125

1028 ->1072 0.12111

Excited State 287: Singlet-A 4.9262 eV 251.69 nm f=0.0061

<S**2>=0.000

977 ->1038 0.13600

1001 ->1032 -0.11893

1003 ->1038 -0.13739

1019 ->1045 0.10322

1020 ->1045 0.21629

Excited State 288: Singlet-A 4.9285 eV 251.57 nm f=0.0005

<S**2>=0.000

1003 ->1038 -0.11724

1018 ->1044 0.11513

1019 ->1044 0.20725

1020 ->1044 -0.19865

Excited State 289: Singlet-A 4.9290 eV 251.54 nm f=0.0015

<S**2>=0.000

1019 ->1044 0.16306

1020 ->1044 -0.15784

1020 ->1045 0.13094

Excited State 290: Singlet-A 4.9303 eV 251.47 nm f=0.0092

<S**2>=0.000

1024 ->1057 0.14214

1025 ->1070 0.10472

1027 ->1069 -0.13774

Excited State 291: Singlet-A 4.9307 eV 251.45 nm f=0.0015

<S**2>=0.000

1019 ->1044 -0.10524

1020 ->1044 0.11261

1020 ->1045 -0.11027

1027 ->1070 -0.10204

Excited State 292: Singlet-A 4.9321 eV 251.38 nm f=0.0071

<S**2>=0.000

990 ->1030 0.10017

993 ->1032 0.17848

995 ->1030 -0.12630

1001 ->1032 0.14038

1011 ->1041 -0.10740

Excited State 293: Singlet-A 4.9334 eV 251.31 nm f=0.0003

<S**2>=0.000

1020 ->1045 -0.15378

1023 ->1048 0.17701

1023 ->1054 -0.11382

1024 ->1048 0.12761

1025 ->1067 -0.12101

1026 ->1069 0.12265

1027 ->1060 -0.10685

1027 ->1065 0.11123

1028 ->1061 -0.10223

Excited State 294: Singlet-A 4.9343 eV 251.27 nm f=0.0038

<S**2>=0.000

992 ->1033 -0.10648

999 ->1030 -0.15199

1002 ->1032 -0.12222

1002 ->1034 0.17557

Excited State 295: Singlet-A 4.9353 eV 251.22 nm f=0.0092

<S**2>=0.000

1019 ->1044 -0.16223

1020 ->1044 0.16944

1020 ->1045 0.14050

1020 ->1047 0.10341

1028 ->1064 0.10118

1029 ->1080 -0.10249

Excited State 296: Singlet-A 4.9371 eV 251.13 nm f=0.0009

<S**2>=0.000

986 ->1031 0.10298

987 ->1031 -0.11977

991 ->1030 0.10102

1001 ->1032 0.13573

1022 ->1057 0.12112

Excited State 297: Singlet-A 4.9378 eV 251.09 nm f=0.0034

<S**2>=0.000

986 ->1031 -0.10333

1000 ->1030 0.10731

1029 ->1080 0.33541

1029 ->1083 -0.11361

1029 ->1088 -0.10937

Excited State 298: Singlet-A 4.9392 eV 251.02 nm f=0.0016

<S**2>=0.000

989 ->1032 -0.12141

992 ->1033 -0.11403

993 ->1031 0.10209

997 ->1034 -0.11920

1000 ->1031 -0.11312

1009 ->1041 0.12878

Excited State 299: Singlet-A 4.9404 eV 250.96 nm f=0.0036

<S**2>=0.000

1024 ->1051 0.14985

1024 ->1054 -0.14031

1024 ->1057 0.16291

1024 ->1060 0.11500

1028 ->1073 -0.10564

Excited State 300: Singlet-A 4.9407 eV 250.94 nm f=0.0081

<S**2>=0.000

1008 ->1041 -0.15840

1009 ->1041 -0.11575

1010 ->1041 -0.13779

1011 ->1041 0.11836

1029 ->1080 0.18884

Part 3

Excited State 1: Singlet-A 4.9422 eV 250.87 nm f=0.0028

<S**2>=0.000

991 ->1030 0.10149

997 ->1030 0.15267

997 ->1034 -0.13575

1001 ->1037 0.10715

1029 ->1080 0.12230

This state for optimization and/or second-order correction.

Total Energy, E(TD-HF/TD-DFT) = -18684.5400903

Copying the excited state density for this state as the 1-particle RhoCI density.

Excited State 2: Singlet-A 4.9437 eV 250.79 nm f=0.0078

<S**2>=0.000

1026 ->1073 0.10483

1029 ->1080 0.16646

Excited State 3: Singlet-A 4.9446 eV 250.75 nm f=0.0015

<S**2>=0.000

988 ->1031 0.11946

990 ->1038 0.15313

1009 ->1041 0.11147

1029 ->1080 -0.11227

Excited State 4: Singlet-A 4.9455 eV 250.70 nm f=0.0014

<S**2>=0.000

991 ->1030 -0.10910

1001 ->1032 -0.11405

1002 ->1036 -0.10158

1009 ->1041 0.13274

Excited State 5: Singlet-A 4.9464 eV 250.66 nm f=0.0013

<S**2>=0.000

1000 ->1030 -0.10781

1000 ->1031 0.12614

1002 ->1034 0.16813

Excited State 6: Singlet-A 4.9477 eV 250.59 nm f=0.0008

<S**2>=0.000

989 ->1030 -0.10466

1002 ->1034 -0.10421

1021 ->1047 -0.10620

1026 ->1067 -0.10511

1027 ->1067 0.11196

Excited State 7: Singlet-A 4.9481 eV 250.57 nm f=0.0041

<S**2>=0.000

990 ->1031 -0.11572

990 ->1038 0.21503

991 ->1030 -0.10043

993 ->1031 -0.11511

993 ->1035 -0.10206

993 ->1038 0.12383

Excited State 8: Singlet-A 4.9488 eV 250.53 nm f=0.0004

<S**2>=0.000

988 ->1030 0.10630

989 ->1030 -0.12854

999 ->1039 0.13514

Excited State 9: Singlet-A 4.9501 eV 250.47 nm f=0.0021

<S**2>=0.000

1007 ->1040 0.11814

1018 ->1046 0.11230

1023 ->1052 -0.11210

Excited State 10: Singlet-A 4.9509 eV 250.43 nm f=0.0047

<S**2>=0.000

984 ->1030 0.11059

989 ->1033 0.12350

992 ->1030 0.10081

993 ->1030 0.10412

1002 ->1034 -0.10118

1027 ->1067 -0.10272

Excited State 11: Singlet-A 4.9522 eV 250.36 nm f=0.0007

<S**2>=0.000

1017 ->1046 0.10093

1018 ->1046 0.12816

1019 ->1046 0.14108

1028 ->1063 -0.10395

Excited State 12: Singlet-A 4.9533 eV 250.31 nm f=0.0061

<S**2>=0.000

1024 ->1060 0.15015

Excited State 13: Singlet-A 4.9540 eV 250.27 nm f=0.0007

<S**2>=0.000

990 ->1038 -0.14797

991 ->1030 -0.10161

993 ->1032 -0.10680

994 ->1030 0.12676

1001 ->1033 0.10127

1002 ->1032 0.10788

1002 ->1033 0.16817

1002 ->1034 -0.12273

1004 ->1035 -0.10349

1023 ->1066 -0.10013

1026 ->1061 -0.10403

1029 ->1080 0.13022

Excited State 14: Singlet-A 4.9554 eV 250.20 nm f=0.0063

<S**2>=0.000

1024 ->1053 -0.11176

1025 ->1061 -0.10335

1026 ->1061 0.19047

1027 ->1065 0.11199

1027 ->1069 -0.11749

1028 ->1062 -0.10224

1028 ->1064 -0.13527

Excited State 15: Singlet-A 4.9559 eV 250.17 nm f=0.0044

<S**2>=0.000

987 ->1030 -0.11130

989 ->1032 0.11617

991 ->1030 -0.11292

999 ->1035 -0.10052

1002 ->1035 -0.10408

Excited State 16: Singlet-A 4.9585 eV 250.04 nm f=0.0165

<S**2>=0.000

1019 ->1049 -0.11933

1022 ->1049 -0.15478

1022 ->1051 0.18890

1028 ->1068 0.10501

Excited State 17: Singlet-A 4.9593 eV 250.00 nm f=0.0032

<S**2>=0.000

994 ->1030 -0.11739

1000 ->1033 0.16387

1000 ->1037 0.14087

Excited State 18: Singlet-A 4.9605 eV 249.94 nm f=0.0042

<S**2>=0.000

1003 ->1038 0.12065

1007 ->1040 0.13043

Excited State 19: Singlet-A 4.9616 eV 249.89 nm f=0.0029

<S**2>=0.000

1000 ->1037 0.13588

1016 ->1044 0.11005

1017 ->1043 -0.11122

Excited State 20: Singlet-A 4.9625 eV 249.84 nm f=0.0010

<S**2>=0.000

1001 ->1035 -0.10398

1003 ->1038 0.10125

1017 ->1043 0.13149

1026 ->1062 0.10285

Excited State 21: Singlet-A 4.9629 eV 249.82 nm f=0.0003

<S**2>=0.000

1007 ->1040 0.15712

1016 ->1044 -0.12238

1026 ->1062 0.10483

1027 ->1067 0.10402

Excited State 22: Singlet-A 4.9641 eV 249.76 nm f=0.0044

<S**2>=0.000

1007 ->1040 -0.10140

1017 ->1043 0.16966

1022 ->1051 -0.13790

1022 ->1052 0.12496

Excited State 23: Singlet-A 4.9647 eV 249.73 nm f=0.0020

<S**2>=0.000

1007 ->1040 -0.12510

1016 ->1044 -0.13299

1019 ->1044 -0.10479

1020 ->1047 -0.11133

1023 ->1051 0.14343

1023 ->1052 -0.10559

1023 ->1053 -0.11915

1024 ->1051 0.11463

Excited State 24: Singlet-A 4.9669 eV 249.62 nm f=0.0007

<S**2>=0.000

980 ->1030 0.16836

981 ->1030 -0.11736

983 ->1030 0.10212

1002 ->1035 0.10492

1007 ->1040 0.10130

1016 ->1044 0.14134

1019 ->1044 0.12168

Excited State 25: Singlet-A 4.9684 eV 249.54 nm f=0.0111

<S**2>=0.000

1007 ->1040 -0.13360

1016 ->1044 0.19999

1019 ->1044 0.16357

1023 ->1052 -0.15276

Excited State 26: Singlet-A 4.9689 eV 249.52 nm f=0.0020

<S**2>=0.000

1024 ->1054 -0.13582

1024 ->1055 0.13765

1027 ->1067 -0.10260

Excited State 27: Singlet-A 4.9697 eV 249.48 nm f=0.0061

<S**2>=0.000

1022 ->1051 -0.10192

1023 ->1051 -0.11032

1023 ->1052 0.12211

1023 ->1058 -0.11147

1024 ->1057 -0.11054

1026 ->1061 0.12250

Excited State 28: Singlet-A 4.9705 eV 249.44 nm f=0.0030

<S**2>=0.000

1003 ->1038 -0.10719

1007 ->1040 0.11331

1016 ->1043 0.11049

1016 ->1044 0.13410

1019 ->1044 0.11615

1023 ->1053 -0.11830

Excited State 29: Singlet-A 4.9716 eV 249.38 nm f=0.0009

<S**2>=0.000

992 ->1036 -0.11251

1002 ->1035 0.12944

1003 ->1038 0.10868

1003 ->1039 -0.10088

Excited State 30: Singlet-A 4.9726 eV 249.33 nm f=0.0035

<S**2>=0.000

1007 ->1040 -0.10576

1017 ->1043 -0.12744

1022 ->1050 0.16543

1023 ->1052 -0.10043

Excited State 31: Singlet-A 4.9763 eV 249.15 nm f=0.0118

<S**2>=0.000

990 ->1030 -0.12191

1001 ->1035 0.14046

1002 ->1035 0.11263

1029 ->1088 0.10034

Excited State 32: Singlet-A 4.9774 eV 249.10 nm f=0.0060

<S**2>=0.000

991 ->1030 0.11807

994 ->1030 -0.10189

996 ->1031 0.11782

1001 ->1034 0.12346

1001 ->1035 0.21222

1002 ->1035 -0.10134

Excited State 33: Singlet-A 4.9783 eV 249.05 nm f=0.0058

<S**2>=0.000

986 ->1031 0.10190

1011 ->1042 0.11271

Excited State 34: Singlet-A 4.9791 eV 249.01 nm f=0.0036

<S**2>=0.000

1000 ->1031 0.12225

1022 ->1050 0.10432

1027 ->1070 0.10160

Excited State 35: Singlet-A 4.9806 eV 248.93 nm f=0.0009

<S**2>=0.000

1000 ->1031 -0.11489

1007 ->1040 0.13950

1022 ->1050 0.13525

Excited State 36: Singlet-A 4.9812 eV 248.90 nm f=0.0025

<S**2>=0.000

978 ->1030 0.10752

990 ->1030 0.18350

1002 ->1035 -0.10506

1026 ->1064 0.16642

1028 ->1068 -0.10495

Excited State 37: Singlet-A 4.9821 eV 248.86 nm f=0.0078

<S**2>=0.000

988 ->1030 -0.13601

990 ->1030 0.18765

Excited State 38: Singlet-A 4.9837 eV 248.78 nm f=0.0016

<S**2>=0.000

1008 ->1041 -0.10299

1009 ->1041 0.14277

1023 ->1054 0.16139

1024 ->1052 0.11253

1024 ->1053 -0.15282

1024 ->1054 -0.10404

1024 ->1055 0.16610

Excited State 39: Singlet-A 4.9841 eV 248.76 nm f=0.0007

<S**2>=0.000

1001 ->1035 0.11224

1002 ->1035 0.15965

1009 ->1041 0.11374

1022 ->1052 0.12314

Excited State 40: Singlet-A 4.9850 eV 248.71 nm f=0.0052

<S**2>=0.000

989 ->1031 0.10049

994 ->1031 -0.09704

995 ->1031 0.09768

995 ->1034 0.10292

996 ->1031 -0.09881

1011 ->1042 0.10735

Excited State 41: Singlet-A 4.9859 eV 248.67 nm f=0.0045

<S**2>=0.000

999 ->1034 0.12993

1000 ->1033 -0.12042

1002 ->1035 -0.10218

1003 ->1038 0.12904

1022 ->1050 -0.11064

Excited State 42: Singlet-A 4.9868 eV 248.62 nm f=0.0052

<S**2>=0.000

1024 ->1061 -0.11077

1028 ->1074 0.11196

Excited State 43: Singlet-A 4.9870 eV 248.62 nm f=0.0075

<S**2>=0.000

1000 ->1037 0.11459

1022 ->1052 0.11923

1023 ->1056 0.10449

1024 ->1060 -0.10821

1028 ->1071 0.10659

1028 ->1074 0.11290

Excited State 44: Singlet-A 4.9874 eV 248.59 nm f=0.0094

<S**2>=0.000

1003 ->1039 -0.10663

1007 ->1041 -0.10208

1009 ->1042 0.14013

1011 ->1042 0.14039

1026 ->1064 0.12824

1029 ->1087 0.16271

Excited State 45: Singlet-A 4.9901 eV 248.46 nm f=0.0004

<S**2>=0.000

1009 ->1041 0.11539

1028 ->1071 -0.13350

1028 ->1074 -0.13759

1029 ->1081 0.12715

1029 ->1088 0.14927

Excited State 46: Singlet-A 4.9914 eV 248.40 nm f=0.0010

<S**2>=0.000

999 ->1037 0.09264

1003 ->1038 0.09770

1004 ->1037 0.10067

Excited State 47: Singlet-A 4.9917 eV 248.38 nm f=0.0055

<S**2>=0.000

992 ->1036 -0.11474

999 ->1036 -0.11962

1029 ->1088 0.12837

Excited State 48: Singlet-A 4.9928 eV 248.33 nm f=0.0007

<S**2>=0.000

1019 ->1045 0.13651

1019 ->1047 0.12320

1028 ->1074 0.10526

Excited State 49: Singlet-A 4.9933 eV 248.30 nm f=0.0005

<S**2>=0.000

986 ->1031 -0.10159

1024 ->1056 0.11266

Excited State 50: Singlet-A 4.9936 eV 248.28 nm f=0.0054

<S**2>=0.000

1003 ->1039 0.11473

1024 ->1054 0.15042

1028 ->1066 0.10283

1029 ->1087 0.10019

Excited State 51: Singlet-A 4.9951 eV 248.21 nm f=0.0003

<S**2>=0.000

981 ->1030 0.11865

1004 ->1037 0.19402

1019 ->1045 0.14770

1024 ->1053 -0.10554

Excited State 52: Singlet-A 4.9962 eV 248.16 nm f=0.0007

<S**2>=0.000

999 ->1037 0.10088

1004 ->1037 0.18282

1019 ->1045 -0.10057

Excited State 53: Singlet-A 4.9966 eV 248.14 nm f=0.0038

<S**2>=0.000

998 ->1036 0.10780

1022 ->1052 -0.10894

1023 ->1054 0.10427

1023 ->1055 0.13050

1029 ->1088 0.11360

Excited State 54: Singlet-A 4.9973 eV 248.10 nm f=0.0047

<S**2>=0.000

1023 ->1053 0.10308

1024 ->1056 -0.12013

1025 ->1064 -0.12406

1028 ->1064 0.11699

1028 ->1068 0.17307

1028 ->1075 -0.11669

Excited State 55: Singlet-A 4.9980 eV 248.07 nm f=0.0008

<S**2>=0.000

983 ->1031 0.10694

1001 ->1035 -0.13569

1004 ->1037 0.14254

1008 ->1041 -0.12482

Excited State 56: Singlet-A 4.9987 eV 248.04 nm f=0.0061

<S**2>=0.000

1003 ->1039 -0.10057

1018 ->1044 -0.13058

1019 ->1045 0.20065

1026 ->1063 0.10000

Excited State 57: Singlet-A 4.9997 eV 247.98 nm f=0.0024

<S**2>=0.000

999 ->1034 0.12524

1000 ->1031 0.10453

1004 ->1037 0.19240

1019 ->1045 0.11432

1028 ->1068 -0.10555

Excited State 58: Singlet-A 5.0002 eV 247.96 nm f=0.0012

<S**2>=0.000

1004 ->1037 -0.11669

1021 ->1048 -0.11750

1026 ->1067 -0.10539

1029 ->1081 0.22394

1029 ->1084 -0.11128

1029 ->1089 0.10203

Excited State 59: Singlet-A 5.0008 eV 247.93 nm f=0.0015

<S**2>=0.000

980 ->1030 -0.10308

1003 ->1038 0.13599

1004 ->1037 0.16292

1019 ->1045 0.12681

Excited State 60: Singlet-A 5.0010 eV 247.92 nm f=0.0009

<S**2>=0.000

1000 ->1037 0.11019

1004 ->1037 0.28032

Excited State 61: Singlet-A 5.0018 eV 247.88 nm f=0.0009

<S**2>=0.000

1019 ->1045 0.27264

1019 ->1046 -0.10656

1020 ->1045 -0.14307

1029 ->1081 -0.11027

Excited State 62: Singlet-A 5.0039 eV 247.78 nm f=0.0042

<S**2>=0.000

1004 ->1037 -0.10058

1011 ->1042 -0.10422

1027 ->1069 -0.12717

1027 ->1076 0.11909

1028 ->1068 0.12089

1029 ->1087 0.14350

Excited State 63: Singlet-A 5.0042 eV 247.76 nm f=0.0025

<S**2>=0.000

984 ->1030 0.12532

1019 ->1045 -0.10146

1019 ->1049 -0.10315

1028 ->1068 -0.11013

Excited State 64: Singlet-A 5.0047 eV 247.73 nm f=0.0061

<S**2>=0.000

1004 ->1037 -0.13283

1026 ->1067 0.13974

1027 ->1069 0.16407

1029 ->1084 -0.10757

Excited State 65: Singlet-A 5.0060 eV 247.67 nm f=0.0000

<S**2>=0.000

1002 ->1038 -0.11963

1003 ->1038 0.12270

1003 ->1039 -0.11149

1018 ->1044 0.17196

1022 ->1052 0.12025

Excited State 66: Singlet-A 5.0065 eV 247.65 nm f=0.0017

<S**2>=0.000

1022 ->1052 0.10689

1022 ->1058 0.10069

1024 ->1054 0.14797

1024 ->1055 -0.11495

1024 ->1058 0.11852

1029 ->1081 0.13501

1029 ->1084 -0.11361

Excited State 67: Singlet-A 5.0076 eV 247.59 nm f=0.0032

<S**2>=0.000

989 ->1032 -0.10669

999 ->1031 0.12102

1018 ->1044 0.12612

1029 ->1081 0.10155

Excited State 68: Singlet-A 5.0082 eV 247.56 nm f=0.0004

<S**2>=0.000

1028 ->1068 0.12218

Excited State 69: Singlet-A 5.0092 eV 247.51 nm f=0.0037

<S**2>=0.000

1018 ->1044 0.36586

1020 ->1044 0.15170

Excited State 70: Singlet-A 5.0103 eV 247.46 nm f=0.0030

<S**2>=0.000

980 ->1030 -0.10974

984 ->1030 -0.17630

996 ->1030 -0.12882

996 ->1033 0.18515

998 ->1037 -0.10911

Excited State 71: Singlet-A 5.0114 eV 247.40 nm f=0.0017

<S**2>=0.000

1010 ->1042 0.13727

Excited State 72: Singlet-A 5.0114 eV 247.40 nm f=0.0025

<S**2>=0.000

1018 ->1044 0.19234

1029 ->1081 0.17345

1029 ->1083 0.11474

Excited State 73: Singlet-A 5.0127 eV 247.34 nm f=0.0082

<S**2>=0.000

1010 ->1042 0.14278

1018 ->1046 -0.10303

1020 ->1046 0.11643

1023 ->1059 -0.10222

1026 ->1068 0.10216

Excited State 74: Singlet-A 5.0128 eV 247.34 nm f=0.0034

<S**2>=0.000

1011 ->1042 -0.12152

1022 ->1054 0.14249

1023 ->1052 -0.10782

1024 ->1056 0.11896

Excited State 75: Singlet-A 5.0137 eV 247.29 nm f=0.0021

<S**2>=0.000

1027 ->1079 0.12560

Excited State 76: Singlet-A 5.0146 eV 247.24 nm f=0.0100

<S**2>=0.000

1026 ->1068 0.12588

Excited State 77: Singlet-A 5.0157 eV 247.19 nm f=0.0040

<S**2>=0.000

1002 ->1037 -0.10522

1002 ->1038 0.14216

Excited State 78: Singlet-A 5.0165 eV 247.16 nm f=0.0049

<S**2>=0.000

988 ->1030 0.12975

990 ->1031 0.10666

Excited State 79: Singlet-A 5.0169 eV 247.13 nm f=0.0028

<S**2>=0.000

988 ->1030 0.12322

1020 ->1046 -0.10350

1021 ->1050 0.10969

1022 ->1054 0.11591

Excited State 80: Singlet-A 5.0178 eV 247.09 nm f=0.0057

<S**2>=0.000

1001 ->1036 0.11714

1002 ->1037 0.10361

1022 ->1058 0.14013

1027 ->1079 0.13055

Excited State 81: Singlet-A 5.0188 eV 247.04 nm f=0.0004

<S**2>=0.000

997 ->1031 0.12302

998 ->1032 0.12635

Excited State 82: Singlet-A 5.0196 eV 247.00 nm f=0.0060

<S**2>=0.000

1016 ->1046 0.11657

1017 ->1046 -0.10743

1020 ->1046 0.18477

1026 ->1067 0.12287

Excited State 83: Singlet-A 5.0200 eV 246.98 nm f=0.0102

<S**2>=0.000

997 ->1031 0.10800

1022 ->1055 0.11674

1022 ->1058 -0.11454

1023 ->1058 0.17318

Excited State 84: Singlet-A 5.0213 eV 246.92 nm f=0.0051

<S**2>=0.000

980 ->1031 -0.10085

986 ->1032 -0.10092

990 ->1031 0.12353

990 ->1032 0.12877

Excited State 85: Singlet-A 5.0217 eV 246.90 nm f=0.0223

<S**2>=0.000

978 ->1030 0.10094

999 ->1032 0.10720

1019 ->1049 0.13148

1021 ->1050 -0.10578

1027 ->1069 0.10870

Excited State 86: Singlet-A 5.0219 eV 246.89 nm f=0.0090

<S**2>=0.000

1007 ->1041 0.18013

1016 ->1045 -0.11128

1020 ->1046 -0.14177

1028 ->1077 -0.15032

Excited State 87: Singlet-A 5.0229 eV 246.84 nm f=0.0011

<S**2>=0.000

1020 ->1046 -0.11251

1024 ->1056 0.15106

1027 ->1069 -0.10302

Excited State 88: Singlet-A 5.0232 eV 246.82 nm f=0.0088

<S**2>=0.000

977 ->1030 0.11047

978 ->1030 0.16209

990 ->1031 0.15098

990 ->1032 0.13095

Excited State 89: Singlet-A 5.0241 eV 246.78 nm f=0.0063

<S**2>=0.000

999 ->1032 0.10952

1010 ->1042 0.11499

1019 ->1050 -0.11310

1022 ->1057 0.11189

Excited State 90: Singlet-A 5.0256 eV 246.70 nm f=0.0039

<S**2>=0.000

1010 ->1042 0.16165

1022 ->1058 -0.11242

1026 ->1068 0.11159

1029 ->1088 -0.10252

Excited State 91: Singlet-A 5.0264 eV 246.67 nm f=0.0132

<S**2>=0.000

1026 ->1068 0.12685

Excited State 92: Singlet-A 5.0282 eV 246.58 nm f=0.0051

<S**2>=0.000

1016 ->1045 0.11310

1017 ->1044 0.13455

1025 ->1063 -0.10892

1027 ->1079 -0.10958

Excited State 93: Singlet-A 5.0284 eV 246.57 nm f=0.0016

<S**2>=0.000

996 ->1034 -0.10791

997 ->1030 -0.17273

997 ->1031 0.18532

997 ->1033 0.13382

997 ->1034 -0.13499

1001 ->1036 0.13926

Excited State 94: Singlet-A 5.0291 eV 246.53 nm f=0.0034

<S**2>=0.000

1028 ->1077 0.17556

1029 ->1081 -0.11658

1029 ->1083 -0.10467

1029 ->1086 -0.10990

1029 ->1088 0.11111

Excited State 95: Singlet-A 5.0304 eV 246.47 nm f=0.0004

<S**2>=0.000

1010 ->1042 -0.11110

1014 ->1046 0.10378

1016 ->1045 0.17982

1017 ->1046 -0.11145

1018 ->1046 0.16590

1028 ->1077 0.10589

Excited State 96: Singlet-A 5.0307 eV 246.46 nm f=0.0036

<S**2>=0.000

982 ->1030 -0.11303

1017 ->1044 -0.11011

1025 ->1063 0.11718

1025 ->1071 0.10462

Excited State 97: Singlet-A 5.0312 eV 246.43 nm f=0.0011

<S**2>=0.000

980 ->1030 0.10416

999 ->1035 -0.11236

1001 ->1036 0.13543

1018 ->1046 0.10099

1020 ->1046 -0.11926

Excited State 98: Singlet-A 5.0316 eV 246.41 nm f=0.0003

<S**2>=0.000

980 ->1030 0.11037

991 ->1031 0.12506

Excited State 99: Singlet-A 5.0335 eV 246.32 nm f=0.0020

<S**2>=0.000

1021 ->1050 0.10368

1022 ->1051 -0.10049

1025 ->1071 0.12380

1026 ->1067 0.10737

Excited State 100: Singlet-A 5.0338 eV 246.30 nm f=0.0019

<S**2>=0.000

992 ->1034 0.10305

1000 ->1035 0.12696

1001 ->1036 -0.10241

1016 ->1045 0.18918

1021 ->1045 0.11106

Excited State 101: Singlet-A 5.0342 eV 246.28 nm f=0.0030

<S**2>=0.000

1016 ->1045 -0.12961

1016 ->1046 0.10802

1025 ->1063 0.18156

1026 ->1063 0.11383

Excited State 102: Singlet-A 5.0350 eV 246.24 nm f=0.0032

<S**2>=0.000

980 ->1031 0.12383

993 ->1033 -0.10400

1017 ->1044 -0.11307

1018 ->1046 0.10305

Excited State 103: Singlet-A 5.0358 eV 246.21 nm f=0.0024

<S**2>=0.000

980 ->1031 0.11133

999 ->1035 0.10013

1001 ->1038 -0.13090

1002 ->1037 0.22756

1003 ->1037 0.12164

Excited State 104: Singlet-A 5.0367 eV 246.16 nm f=0.0044

<S**2>=0.000

997 ->1037 0.11411

999 ->1035 0.15422

1017 ->1044 0.18329

Excited State 105: Singlet-A 5.0373 eV 246.13 nm f=0.0076

<S**2>=0.000

1001 ->1036 0.10219

1004 ->1039 0.11861

1017 ->1044 0.11691

1022 ->1054 0.11404

Excited State 106: Singlet-A 5.0377 eV 246.11 nm f=0.0025

<S**2>=0.000

992 ->1031 0.13509

Excited State 107: Singlet-A 5.0388 eV 246.06 nm f=0.0012

<S**2>=0.000

1017 ->1044 0.18246

1025 ->1063 -0.10299

1029 ->1087 0.12712

Excited State 108: Singlet-A 5.0402 eV 245.99 nm f=0.0015

<S**2>=0.000

1017 ->1044 0.18166

1025 ->1065 -0.10251

1025 ->1068 0.10467

1025 ->1071 -0.11022

Excited State 109: Singlet-A 5.0413 eV 245.94 nm f=0.0144

<S**2>=0.000

1023 ->1057 0.15266

1024 ->1056 0.12798

1025 ->1060 -0.11662

1025 ->1063 0.13366

1025 ->1065 0.13926

Excited State 110: Singlet-A 5.0418 eV 245.91 nm f=0.0040

<S**2>=0.000

1023 ->1059 0.10574

1029 ->1080 -0.11873

1029 ->1081 0.11234

1029 ->1083 0.11831

1029 ->1086 0.11997

1029 ->1088 -0.10815

Excited State 111: Singlet-A 5.0431 eV 245.85 nm f=0.0007

<S**2>=0.000

1004 ->1039 0.10283

1017 ->1044 0.12147

1028 ->1077 0.11146

Excited State 112: Singlet-A 5.0437 eV 245.82 nm f=0.0002

<S**2>=0.000

1002 ->1037 0.11762

1028 ->1077 0.10556

1029 ->1087 -0.10102

Excited State 113: Singlet-A 5.0442 eV 245.79 nm f=0.0048

<S**2>=0.000

982 ->1030 -0.10002

995 ->1033 0.10406

1002 ->1037 0.12533

1004 ->1039 0.21737

1017 ->1044 -0.12509

Excited State 114: Singlet-A 5.0461 eV 245.70 nm f=0.0069

<S**2>=0.000

1001 ->1039 0.10987

1018 ->1052 -0.10796

1020 ->1049 -0.10365

Excited State 115: Singlet-A 5.0468 eV 245.67 nm f=0.0010

<S**2>=0.000

1002 ->1037 0.13706

1002 ->1039 0.10844

1004 ->1039 -0.11557

Excited State 116: Singlet-A 5.0477 eV 245.63 nm f=0.0009

<S**2>=0.000

994 ->1032 0.10351

1025 ->1063 0.10617

1025 ->1068 0.12417

1026 ->1069 -0.11891

1027 ->1069 0.11197

1028 ->1068 -0.10060

Excited State 117: Singlet-A 5.0479 eV 245.61 nm f=0.0012

<S**2>=0.000

980 ->1031 -0.12818

997 ->1033 0.12159

999 ->1037 0.11670

1001 ->1039 0.15925

Excited State 118: Singlet-A 5.0493 eV 245.55 nm f=0.0018

<S**2>=0.000

1004 ->1039 0.12031

1020 ->1047 0.11301

1021 ->1047 0.10498

Excited State 119: Singlet-A 5.0500 eV 245.51 nm f=0.0008

<S**2>=0.000

982 ->1030 0.17452

997 ->1031 0.11248

1007 ->1041 0.10203

Excited State 120: Singlet-A 5.0509 eV 245.47 nm f=0.0061

<S**2>=0.000

997 ->1037 0.16141

999 ->1035 -0.11257

999 ->1039 -0.10562

Excited State 121: Singlet-A 5.0511 eV 245.46 nm f=0.0021

<S**2>=0.000

982 ->1030 -0.11843

993 ->1033 0.13856

997 ->1036 0.10035

997 ->1037 0.18187

1001 ->1039 -0.12003

Excited State 122: Singlet-A 5.0522 eV 245.41 nm f=0.0063

<S**2>=0.000

1023 ->1057 -0.12346

1023 ->1059 -0.11166

1025 ->1062 -0.11660

1025 ->1066 -0.13393

1025 ->1069 0.14256

1025 ->1074 0.11769

1026 ->1066 -0.10185

Excited State 123: Singlet-A 5.0530 eV 245.37 nm f=0.0042

<S**2>=0.000

994 ->1033 0.10533

997 ->1033 0.11354

1023 ->1055 -0.10091

Excited State 124: Singlet-A 5.0540 eV 245.32 nm f=0.0029

<S**2>=0.000

980 ->1031 0.14071

997 ->1033 0.11849

997 ->1037 0.14787

Excited State 125: Singlet-A 5.0545 eV 245.30 nm f=0.0013

<S**2>=0.000

1007 ->1041 -0.15135

1018 ->1045 0.26785

Excited State 126: Singlet-A 5.0553 eV 245.25 nm f=0.0009

<S**2>=0.000

1001 ->1039 0.10742

1007 ->1041 0.11458

1018 ->1045 0.13399

1027 ->1078 -0.10412

Excited State 127: Singlet-A 5.0564 eV 245.20 nm f=0.0015

<S**2>=0.000

1007 ->1041 0.11524

1015 ->1045 0.12379

1018 ->1045 0.34763

1018 ->1046 -0.12092

Excited State 128: Singlet-A 5.0573 eV 245.16 nm f=0.0049

<S**2>=0.000

1018 ->1045 0.12493

1018 ->1052 0.10343

1025 ->1064 0.12396

Excited State 129: Singlet-A 5.0577 eV 245.14 nm f=0.0025

<S**2>=0.000

1001 ->1039 0.26749

1002 ->1039 -0.11415

1010 ->1042 0.10724

1029 ->1087 -0.10438

Excited State 130: Singlet-A 5.0583 eV 245.11 nm f=0.0088

<S**2>=0.000

998 ->1034 0.11656

1020 ->1049 0.13959

1021 ->1048 -0.11004

1027 ->1070 0.11404

Excited State 131: Singlet-A 5.0586 eV 245.10 nm f=0.0065

<S**2>=0.000

1001 ->1039 0.14866

1019 ->1052 0.11103

1021 ->1050 0.12872

Excited State 132: Singlet-A 5.0591 eV 245.07 nm f=0.0006

<S**2>=0.000

994 ->1034 -0.11874

1004 ->1039 0.13536

Excited State 133: Singlet-A 5.0596 eV 245.05 nm f=0.0104

<S**2>=0.000

1023 ->1057 -0.09322

1023 ->1059 0.10229

Excited State 134: Singlet-A 5.0610 eV 244.98 nm f=0.0033

<S**2>=0.000

1020 ->1049 0.12091

1022 ->1056 0.11346

1023 ->1065 0.11431

Excited State 135: Singlet-A 5.0616 eV 244.95 nm f=0.0031

<S**2>=0.000

992 ->1033 0.14193

998 ->1034 -0.10028

1001 ->1039 -0.10653

1002 ->1039 0.10103

1007 ->1041 -0.11689

Excited State 136: Singlet-A 5.0628 eV 244.89 nm f=0.0048

<S**2>=0.000

982 ->1030 0.12162

985 ->1030 0.10897

Excited State 137: Singlet-A 5.0632 eV 244.87 nm f=0.0027

<S**2>=0.000

1021 ->1048 0.14729

1022 ->1057 -0.10088

1025 ->1069 0.10722

Excited State 138: Singlet-A 5.0640 eV 244.83 nm f=0.0069

<S**2>=0.000

998 ->1035 -0.11206

1007 ->1041 0.12758

Excited State 139: Singlet-A 5.0650 eV 244.78 nm f=0.0011

<S**2>=0.000

982 ->1030 0.12451

Excited State 140: Singlet-A 5.0651 eV 244.78 nm f=0.0124

<S**2>=0.000

979 ->1037 0.09816

1001 ->1039 0.09396

1007 ->1041 0.09164

Excited State 141: Singlet-A 5.0668 eV 244.70 nm f=0.0004

<S**2>=0.000

986 ->1033 -0.10274

988 ->1033 0.10031

991 ->1033 0.13872

995 ->1033 0.12949

998 ->1034 -0.10137

998 ->1035 0.11431

Excited State 142: Singlet-A 5.0674 eV 244.67 nm f=0.0031

<S**2>=0.000

982 ->1030 0.12294

982 ->1031 0.13593

985 ->1030 0.12298

985 ->1033 -0.10426

995 ->1034 -0.10474

1007 ->1041 -0.10655

Excited State 143: Singlet-A 5.0676 eV 244.66 nm f=0.0016

<S**2>=0.000

979 ->1037 0.11014

994 ->1033 0.11726

1002 ->1037 0.11273

Excited State 144: Singlet-A 5.0677 eV 244.66 nm f=0.0046

<S**2>=0.000

1001 ->1038 0.10776

1002 ->1037 0.11366

1002 ->1038 0.10422

1024 ->1061 -0.10778

Excited State 145: Singlet-A 5.0688 eV 244.60 nm f=0.0015

<S**2>=0.000

1019 ->1047 0.10559

Excited State 146: Singlet-A 5.0699 eV 244.55 nm f=0.0004

<S**2>=0.000

979 ->1037 -0.10676

997 ->1032 -0.10547

1000 ->1036 0.18328

1001 ->1038 -0.13462

Excited State 147: Singlet-A 5.0700 eV 244.54 nm f=0.0016

<S**2>=0.000

1000 ->1036 0.11457

Excited State 148: Singlet-A 5.0711 eV 244.49 nm f=0.0027

<S**2>=0.000

994 ->1033 0.11514

997 ->1032 0.10842

1002 ->1038 -0.10887

Excited State 149: Singlet-A 5.0720 eV 244.45 nm f=0.0035

<S**2>=0.000

1000 ->1036 0.12229

1018 ->1049 0.12354

1023 ->1062 -0.11416

1025 ->1067 0.10432

Excited State 150: Singlet-A 5.0730 eV 244.40 nm f=0.0011

<S**2>=0.000

994 ->1036 0.11811

1001 ->1036 -0.14500

1001 ->1038 0.17062

1001 ->1039 -0.15452

Excited State 151: Singlet-A 5.0736 eV 244.37 nm f=0.0012

<S**2>=0.000

1028 ->1076 0.14050

Excited State 152: Singlet-A 5.0747 eV 244.32 nm f=0.0026

<S**2>=0.000

1002 ->1038 -0.11586

1022 ->1056 -0.10268

1023 ->1063 0.12371

Excited State 153: Singlet-A 5.0755 eV 244.28 nm f=0.0048

<S**2>=0.000

979 ->1037 0.13578

996 ->1034 0.10587

1002 ->1038 -0.10873

Excited State 154: Singlet-A 5.0757 eV 244.27 nm f=0.0018

<S**2>=0.000

1029 ->1085 0.10905

1029 ->1089 0.15070

Excited State 155: Singlet-A 5.0763 eV 244.24 nm f=0.0012

<S**2>=0.000

994 ->1036 0.10671

1018 ->1045 0.10367

1018 ->1051 0.11258

1022 ->1058 -0.10220

Excited State 156: Singlet-A 5.0771 eV 244.21 nm f=0.0011

<S**2>=0.000

993 ->1031 0.11709

995 ->1034 0.10004

1000 ->1036 -0.11549

1000 ->1038 0.11695

Excited State 157: Singlet-A 5.0774 eV 244.19 nm f=0.0007

<S**2>=0.000

992 ->1033 -0.11181

994 ->1036 0.11965

Excited State 158: Singlet-A 5.0781 eV 244.15 nm f=0.0007

<S**2>=0.000

1021 ->1048 -0.11486

1023 ->1065 0.13864

Excited State 159: Singlet-A 5.0793 eV 244.10 nm f=0.0014

<S**2>=0.000

993 ->1031 0.11043

Excited State 160: Singlet-A 5.0794 eV 244.09 nm f=0.0033

<S**2>=0.000

989 ->1033 0.10097

998 ->1035 0.09275

1023 ->1062 -0.09674

Excited State 161: Singlet-A 5.0805 eV 244.04 nm f=0.0005

<S**2>=0.000

991 ->1031 0.12066

991 ->1033 0.12627

991 ->1036 -0.13740

995 ->1032 0.13658

998 ->1032 0.14652

Excited State 162: Singlet-A 5.0812 eV 244.01 nm f=0.0021

<S**2>=0.000

993 ->1032 -0.11436

995 ->1031 -0.13603

1000 ->1036 0.19879

Excited State 163: Singlet-A 5.0818 eV 243.98 nm f=0.0036

<S**2>=0.000

993 ->1031 0.11022

995 ->1034 0.12605

1027 ->1071 -0.11835

Excited State 164: Singlet-A 5.0824 eV 243.95 nm f=0.0006

<S**2>=0.000

993 ->1032 0.10095

995 ->1031 0.12558

997 ->1032 -0.11657

998 ->1036 -0.11552

Excited State 165: Singlet-A 5.0828 eV 243.93 nm f=0.0041

<S**2>=0.000

1000 ->1036 0.12892

1001 ->1039 0.10084

Excited State 166: Singlet-A 5.0844 eV 243.85 nm f=0.0024

<S**2>=0.000

977 ->1030 -0.10546

992 ->1034 0.10330

994 ->1034 0.11367

1000 ->1038 0.10585

Excited State 167: Singlet-A 5.0845 eV 243.85 nm f=0.0093

<S**2>=0.000

968 ->1031 0.11390

1016 ->1045 -0.14876

1017 ->1045 0.27421

1019 ->1045 0.10151

Excited State 168: Singlet-A 5.0852 eV 243.81 nm f=0.0056

<S**2>=0.000

1018 ->1049 -0.10408

1027 ->1078 0.12154

Excited State 169: Singlet-A 5.0865 eV 243.75 nm f=0.0020

<S**2>=0.000

1016 ->1045 -0.10857

1017 ->1045 0.28589

Excited State 170: Singlet-A 5.0870 eV 243.73 nm f=0.0000

<S**2>=0.000

991 ->1032 -0.12441

991 ->1033 0.14324

996 ->1031 -0.11027

996 ->1036 -0.11549

997 ->1038 -0.12497

1001 ->1038 -0.10871

Excited State 171: Singlet-A 5.0883 eV 243.67 nm f=0.0026

<S**2>=0.000

996 ->1034 -0.10500

1025 ->1075 0.10543

1029 ->1087 0.14016

1029 ->1089 -0.13409

Excited State 172: Singlet-A 5.0889 eV 243.64 nm f=0.0020

<S**2>=0.000

992 ->1034 0.10196

1026 ->1071 -0.11866

1026 ->1074 0.12230

1027 ->1073 0.13730

1027 ->1076 0.10743

Excited State 173: Singlet-A 5.0894 eV 243.61 nm f=0.0035

<S**2>=0.000

992 ->1034 0.12093

1025 ->1066 0.12832

1025 ->1069 0.14029

1026 ->1070 0.16513

1027 ->1071 0.10184

1027 ->1073 -0.13501

Excited State 174: Singlet-A 5.0900 eV 243.59 nm f=0.0031

<S**2>=0.000

991 ->1034 -0.10447

1024 ->1065 0.13602

1027 ->1078 0.13539

Excited State 175: Singlet-A 5.0912 eV 243.53 nm f=0.0019

<S**2>=0.000

992 ->1034 0.12177

1023 ->1063 -0.10938

1024 ->1066 -0.11201

Excited State 176: Singlet-A 5.0916 eV 243.51 nm f=0.0038

<S**2>=0.000

991 ->1034 -0.09664

1022 ->1060 0.09582

1023 ->1064 -0.09715

1024 ->1062 -0.09582

1025 ->1070 -0.09559

1026 ->1073 -0.09828

1027 ->1070 0.10061

Excited State 177: Singlet-A 5.0923 eV 243.47 nm f=0.0020

<S**2>=0.000

991 ->1033 -0.10891

991 ->1034 0.12204

Excited State 178: Singlet-A 5.0927 eV 243.46 nm f=0.0023

<S**2>=0.000

984 ->1033 -0.10124

987 ->1031 -0.11406

987 ->1033 -0.10541

988 ->1031 -0.10742

989 ->1034 0.10641

990 ->1030 0.11317

991 ->1034 0.11772

997 ->1035 -0.11534

1002 ->1038 0.11722

Excited State 179: Singlet-A 5.0934 eV 243.42 nm f=0.0046

<S**2>=0.000

1017 ->1045 0.16011

1023 ->1065 0.13153

1025 ->1068 -0.10764

1025 ->1078 -0.10856

1027 ->1078 -0.14625

Excited State 180: Singlet-A 5.0947 eV 243.36 nm f=0.0018

<S**2>=0.000

991 ->1034 0.15817

999 ->1039 0.10557

1000 ->1036 0.13828

Excited State 181: Singlet-A 5.0954 eV 243.33 nm f=0.0074

<S**2>=0.000

1023 ->1063 0.12998

1027 ->1071 -0.10244

Excited State 182: Singlet-A 5.0959 eV 243.30 nm f=0.0020

<S**2>=0.000

980 ->1032 -0.11144

988 ->1034 -0.10002

998 ->1035 0.15127

Excited State 183: Singlet-A 5.0966 eV 243.27 nm f=0.0005

<S**2>=0.000

986 ->1033 -0.10700

986 ->1039 0.12874

994 ->1034 0.15418

994 ->1036 -0.10679

996 ->1032 0.11450

1000 ->1038 -0.12414

Excited State 184: Singlet-A 5.0973 eV 243.23 nm f=0.0052

<S**2>=0.000

1025 ->1066 0.16614

1025 ->1079 0.10487

1026 ->1074 0.13271

1028 ->1075 0.10773

Excited State 185: Singlet-A 5.0977 eV 243.22 nm f=0.0039

<S**2>=0.000

1000 ->1036 0.12736

1000 ->1038 0.13212

1001 ->1038 0.12265

1025 ->1074 -0.10237

1028 ->1074 -0.10565

Excited State 186: Singlet-A 5.0984 eV 243.18 nm f=0.0029

<S**2>=0.000

997 ->1038 0.10849

998 ->1035 0.10109

1025 ->1070 0.10309

Excited State 187: Singlet-A 5.0984 eV 243.18 nm f=0.0061

<S**2>=0.000

1009 ->1042 0.10631

1023 ->1065 -0.13151

1025 ->1070 0.14366

1025 ->1074 -0.11105

Excited State 188: Singlet-A 5.0998 eV 243.12 nm f=0.0017

<S**2>=0.000

987 ->1034 0.12938

998 ->1035 -0.11492

1000 ->1038 0.15458

1001 ->1038 0.12107

Excited State 189: Singlet-A 5.1008 eV 243.07 nm f=0.0028

<S**2>=0.000

971 ->1033 0.10008

971 ->1034 -0.10967

972 ->1030 0.11025

987 ->1032 -0.11394

990 ->1031 0.12815

Excited State 190: Singlet-A 5.1010 eV 243.06 nm f=0.0019

<S**2>=0.000

1015 ->1043 0.13851

Excited State 191: Singlet-A 5.1021 eV 243.00 nm f=0.0017

<S**2>=0.000

990 ->1037 -0.10951

1015 ->1043 0.12844

Excited State 192: Singlet-A 5.1028 eV 242.97 nm f=0.0035

<S**2>=0.000

1022 ->1065 0.10524

1023 ->1064 0.10744

1024 ->1065 0.11577

Excited State 193: Singlet-A 5.1034 eV 242.94 nm f=0.0002

<S**2>=0.000

978 ->1031 0.10098

989 ->1031 0.13617

Excited State 194: Singlet-A 5.1044 eV 242.89 nm f=0.0025

<S**2>=0.000

1025 ->1070 0.17205

1026 ->1073 -0.14218

1027 ->1071 0.14443

Excited State 195: Singlet-A 5.1049 eV 242.87 nm f=0.0027

<S**2>=0.000

971 ->1030 0.11304

1022 ->1059 -0.11210

Excited State 196: Singlet-A 5.1052 eV 242.86 nm f=0.0014

<S**2>=0.000

1023 ->1060 0.18440

1025 ->1070 -0.10128

1027 ->1071 -0.15911

Excited State 197: Singlet-A 5.1060 eV 242.82 nm f=0.0005

<S**2>=0.000

977 ->1030 0.14312

980 ->1036 -0.11528

987 ->1032 0.11654

990 ->1033 0.10437

991 ->1034 -0.11836

992 ->1032 0.10831

Excited State 198: Singlet-A 5.1070 eV 242.77 nm f=0.0073

<S**2>=0.000

1022 ->1056 0.12824

1023 ->1060 0.15446

1023 ->1063 -0.10534

Excited State 199: Singlet-A 5.1075 eV 242.75 nm f=0.0023

<S**2>=0.000

1009 ->1042 -0.12851

1015 ->1043 -0.10656

1020 ->1050 0.17953

1020 ->1051 -0.10435

Excited State 200: Singlet-A 5.1086 eV 242.70 nm f=0.0097

<S**2>=0.000

986 ->1034 0.07612

998 ->1034 0.08440

998 ->1036 -0.08009

1019 ->1051 -0.07796

1021 ->1056 0.07953

1025 ->1069 -0.08000

1025 ->1078 -0.07614

1026 ->1077 -0.07659

1028 ->1074 0.08406

Excited State 201: Singlet-A 5.1095 eV 242.66 nm f=0.0065

<S**2>=0.000

1025 ->1071 0.11631

1026 ->1073 0.12919

1026 ->1077 -0.10279

1027 ->1071 -0.12566

1029 ->1085 0.11914

Excited State 202: Singlet-A 5.1106 eV 242.60 nm f=0.0024

<S**2>=0.000

1008 ->1042 -0.12683

1009 ->1042 0.14657

Excited State 203: Singlet-A 5.1108 eV 242.59 nm f=0.0036

<S**2>=0.000

988 ->1034 -0.10897

1002 ->1038 0.12322

1020 ->1050 0.11113

Excited State 204: Singlet-A 5.1116 eV 242.55 nm f=0.0016

<S**2>=0.000

981 ->1031 -0.10505

998 ->1039 0.11211

1000 ->1039 -0.11201

Excited State 205: Singlet-A 5.1126 eV 242.51 nm f=0.0011

<S**2>=0.000

985 ->1031 0.10245

993 ->1035 0.13738

998 ->1035 0.11741

Excited State 206: Singlet-A 5.1137 eV 242.45 nm f=0.0013

<S**2>=0.000

979 ->1031 0.16561

980 ->1036 -0.10454

997 ->1035 0.10706

1005 ->1040 0.12248

1015 ->1043 -0.11532

Excited State 207: Singlet-A 5.1140 eV 242.44 nm f=0.0032

<S**2>=0.000

997 ->1035 0.14912

997 ->1038 -0.13209

1022 ->1059 -0.11370

Excited State 208: Singlet-A 5.1150 eV 242.39 nm f=0.0015

<S**2>=0.000

1022 ->1059 0.12813

1025 ->1070 -0.12099

Excited State 209: Singlet-A 5.1160 eV 242.34 nm f=0.0017

<S**2>=0.000

1021 ->1047 -0.11984

1021 ->1051 0.22236

1025 ->1070 0.10201

Excited State 210: Singlet-A 5.1167 eV 242.31 nm f=0.0002

<S**2>=0.000

978 ->1032 -0.11341

987 ->1034 -0.10206

995 ->1035 -0.10878

1021 ->1051 0.11346

Excited State 211: Singlet-A 5.1179 eV 242.25 nm f=0.0001

<S**2>=0.000

980 ->1036 -0.10106

996 ->1035 -0.11636

1015 ->1046 -0.11011

1016 ->1046 0.13523

1017 ->1046 0.12333

1021 ->1053 -0.10496

Excited State 212: Singlet-A 5.1187 eV 242.22 nm f=0.0016

<S**2>=0.000

1009 ->1042 0.13918

1015 ->1044 0.10399

1024 ->1063 0.12245

1024 ->1065 -0.10122

Excited State 213: Singlet-A 5.1189 eV 242.21 nm f=0.0004

<S**2>=0.000

993 ->1034 -0.11561

993 ->1035 0.10255

993 ->1036 -0.10521

995 ->1034 0.10851

996 ->1035 -0.11426

997 ->1036 0.14956

997 ->1038 -0.10108

Excited State 214: Singlet-A 5.1198 eV 242.16 nm f=0.0007

<S**2>=0.000

986 ->1034 -0.10938

987 ->1034 0.16612

990 ->1034 -0.12388

998 ->1037 0.11952

1000 ->1039 -0.10432

Excited State 215: Singlet-A 5.1210 eV 242.11 nm f=0.0035

<S**2>=0.000

1016 ->1046 0.11089

1022 ->1059 0.18091

1022 ->1061 -0.11204

1024 ->1061 0.10425

1027 ->1072 0.10433

Excited State 216: Singlet-A 5.1219 eV 242.07 nm f=0.0019

<S**2>=0.000

977 ->1030 0.10944

992 ->1035 0.10605

1027 ->1081 -0.09933

Excited State 217: Singlet-A 5.1227 eV 242.03 nm f=0.0071

<S**2>=0.000

992 ->1035 0.10285

994 ->1035 -0.13245

994 ->1037 0.14041

1029 ->1085 -0.12872

1029 ->1086 -0.10032

Excited State 218: Singlet-A 5.1232 eV 242.00 nm f=0.0015

<S**2>=0.000

1019 ->1053 0.10516

1023 ->1062 0.13173

1024 ->1062 -0.12378

Excited State 219: Singlet-A 5.1235 eV 241.99 nm f=0.0010

<S**2>=0.000

1009 ->1042 -0.10403

1014 ->1044 0.12157

1015 ->1044 0.19473

1017 ->1044 0.11139

Excited State 220: Singlet-A 5.1241 eV 241.96 nm f=0.0027

<S**2>=0.000

1024 ->1061 0.10988

1024 ->1063 -0.10281

1024 ->1067 -0.10209

Excited State 221: Singlet-A 5.1247 eV 241.93 nm f=0.0004

<S**2>=0.000

993 ->1034 -0.13070

993 ->1035 0.13364

Excited State 222: Singlet-A 5.1252 eV 241.91 nm f=0.0020

<S**2>=0.000

977 ->1030 0.16893

981 ->1032 -0.10621

997 ->1038 0.13457

Excited State 223: Singlet-A 5.1258 eV 241.88 nm f=0.0024

<S**2>=0.000

973 ->1031 0.10487

982 ->1035 -0.10140

991 ->1034 0.10560

1005 ->1040 0.12257

Excited State 224: Singlet-A 5.1269 eV 241.83 nm f=0.0004

<S**2>=0.000

984 ->1034 -0.12393

993 ->1035 0.10921

994 ->1034 0.15020

994 ->1037 0.13002

996 ->1037 0.10770

997 ->1038 0.13761

Excited State 225: Singlet-A 5.1278 eV 241.79 nm f=0.0018

<S**2>=0.000

991 ->1035 0.10789

996 ->1033 0.11342

996 ->1035 -0.10107

998 ->1037 0.11583

1029 ->1085 0.13301

Excited State 226: Singlet-A 5.1279 eV 241.78 nm f=0.0011

<S**2>=0.000

987 ->1034 0.11442

989 ->1033 0.12545

1005 ->1040 -0.10900

1015 ->1044 0.10030

Excited State 227: Singlet-A 5.1282 eV 241.77 nm f=0.0014

<S**2>=0.000

1029 ->1085 0.19681

Excited State 228: Singlet-A 5.1292 eV 241.72 nm f=0.0043

<S**2>=0.000

973 ->1030 0.09909

986 ->1033 0.09539

996 ->1036 -0.09237

998 ->1039 0.08962

1021 ->1052 -0.09508

Excited State 229: Singlet-A 5.1300 eV 241.68 nm f=0.0020

<S**2>=0.000

994 ->1037 -0.08620

1000 ->1039 -0.08784

1014 ->1043 0.08555

1029 ->1090 0.09354

Excited State 230: Singlet-A 5.1315 eV 241.62 nm f=0.0012

<S**2>=0.000

987 ->1032 0.11308

993 ->1035 0.10276

996 ->1037 0.12970

997 ->1036 -0.10623

997 ->1037 0.13802

Excited State 231: Singlet-A 5.1319 eV 241.60 nm f=0.0036

<S**2>=0.000

1015 ->1044 0.13734

1017 ->1047 0.16447

1027 ->1081 0.14612

1029 ->1085 0.10203

Excited State 232: Singlet-A 5.1324 eV 241.57 nm f=0.0006

<S**2>=0.000

984 ->1036 0.12735

991 ->1032 -0.10749

997 ->1036 0.14362

Excited State 233: Singlet-A 5.1327 eV 241.56 nm f=0.0023

<S**2>=0.000

1018 ->1051 0.11065

1028 ->1078 -0.10487

Excited State 234: Singlet-A 5.1337 eV 241.51 nm f=0.0012

<S**2>=0.000

984 ->1034 0.10632

988 ->1033 0.10300

Excited State 235: Singlet-A 5.1341 eV 241.49 nm f=0.0009

<S**2>=0.000

968 ->1031 0.10484

1009 ->1042 0.09990

1028 ->1075 0.10558

Excited State 236: Singlet-A 5.1344 eV 241.48 nm f=0.0020

<S**2>=0.000

990 ->1034 0.13444

Excited State 237: Singlet-A 5.1354 eV 241.43 nm f=0.0039

<S**2>=0.000

1018 ->1048 0.10103

1023 ->1057 -0.11082

1027 ->1081 0.14575

1029 ->1086 0.10554

1029 ->1090 -0.12774

Excited State 238: Singlet-A 5.1368 eV 241.36 nm f=0.0023

<S**2>=0.000

968 ->1031 0.12761

987 ->1034 0.10037

990 ->1034 0.12374

1027 ->1081 -0.11137

Excited State 239: Singlet-A 5.1370 eV 241.36 nm f=0.0007

<S**2>=0.000

968 ->1031 0.16480

969 ->1030 -0.10061

980 ->1030 0.11306

980 ->1032 -0.12885

Excited State 240: Singlet-A 5.1380 eV 241.31 nm f=0.0011

<S**2>=0.000

969 ->1030 0.10338

983 ->1032 0.10755

987 ->1033 0.11774

993 ->1033 -0.10280

994 ->1034 0.12552

995 ->1035 -0.11889

996 ->1035 0.10791

996 ->1037 0.12216

Excited State 241: Singlet-A 5.1385 eV 241.29 nm f=0.0033

<S**2>=0.000

993 ->1035 0.10975

1019 ->1048 0.11463

1020 ->1052 0.12857

1022 ->1059 0.11243

Excited State 242: Singlet-A 5.1388 eV 241.27 nm f=0.0008

<S**2>=0.000

1007 ->1042 -0.10103

1020 ->1052 -0.11672

1029 ->1086 0.15042

1029 ->1090 -0.12060

Excited State 243: Singlet-A 5.1391 eV 241.26 nm f=0.0005

<S**2>=0.000

986 ->1034 0.10087

989 ->1034 0.17839

990 ->1033 0.13841

990 ->1034 -0.11272

999 ->1038 0.12424

Excited State 244: Singlet-A 5.1399 eV 241.22 nm f=0.0009

<S**2>=0.000

975 ->1030 0.16912

991 ->1035 0.15333

Excited State 245: Singlet-A 5.1409 eV 241.17 nm f=0.0048

<S**2>=0.000

1026 ->1072 -0.10161

1027 ->1072 -0.12009

1027 ->1073 -0.11348

1029 ->1085 0.14537

1029 ->1086 -0.11910

1029 ->1088 -0.11638

1029 ->1090 0.12891

Excited State 246: Singlet-A 5.1417 eV 241.14 nm f=0.0025

<S**2>=0.000

1019 ->1048 -0.12942

1020 ->1053 0.13911

1026 ->1072 0.11181

1027 ->1072 0.14951

Excited State 247: Singlet-A 5.1425 eV 241.10 nm f=0.0016

<S**2>=0.000

996 ->1036 -0.12995

999 ->1038 0.15331

1014 ->1043 -0.13652

Excited State 248: Singlet-A 5.1436 eV 241.04 nm f=0.0009

<S**2>=0.000

984 ->1034 -0.11614

1027 ->1072 0.13123

Excited State 249: Singlet-A 5.1443 eV 241.01 nm f=0.0068

<S**2>=0.000

1007 ->1042 0.11771

1027 ->1072 0.14351

Excited State 250: Singlet-A 5.1450 eV 240.98 nm f=0.0029

<S**2>=0.000

1023 ->1069 0.11138

Excited State 251: Singlet-A 5.1454 eV 240.96 nm f=0.0012

<S**2>=0.000

985 ->1032 0.11682

999 ->1038 0.10475

Excited State 252: Singlet-A 5.1459 eV 240.94 nm f=0.0011

<S**2>=0.000

988 ->1033 0.10020

990 ->1037 -0.11208

991 ->1035 0.10951

992 ->1037 0.15130

993 ->1034 0.14213

994 ->1037 0.10221

995 ->1033 -0.10132

995 ->1038 -0.11270

996 ->1035 -0.10680

Excited State 253: Singlet-A 5.1466 eV 240.90 nm f=0.0010

<S**2>=0.000

982 ->1032 0.13696

982 ->1034 0.11077

982 ->1035 -0.10266

982 ->1036 0.10906

985 ->1034 0.16563

996 ->1036 -0.15893

Excited State 254: Singlet-A 5.1469 eV 240.89 nm f=0.0099

<S**2>=0.000

1021 ->1052 0.14732

Excited State 255: Singlet-A 5.1479 eV 240.84 nm f=0.0052

<S**2>=0.000

1027 ->1073 0.16911

1029 ->1090 0.13719

Excited State 256: Singlet-A 5.1489 eV 240.80 nm f=0.0055

<S**2>=0.000

983 ->1034 -0.11548

985 ->1034 -0.11487

992 ->1037 -0.14013

993 ->1033 0.14774

993 ->1034 0.15950

993 ->1035 0.11774

Excited State 257: Singlet-A 5.1496 eV 240.77 nm f=0.0005

<S**2>=0.000

974 ->1031 0.11093

974 ->1032 -0.10236

982 ->1034 0.11231

983 ->1038 0.12972

985 ->1038 -0.12685

986 ->1035 0.11892

986 ->1038 0.13039

988 ->1034 0.12514

Excited State 258: Singlet-A 5.1505 eV 240.72 nm f=0.0040

<S**2>=0.000

1020 ->1054 0.12009

1022 ->1060 0.12207

Excited State 259: Singlet-A 5.1511 eV 240.70 nm f=0.0014

<S**2>=0.000

998 ->1037 -0.10211

1018 ->1057 0.10217

1022 ->1067 0.10770

Excited State 260: Singlet-A 5.1513 eV 240.68 nm f=0.0023

<S**2>=0.000

994 ->1035 0.11258

998 ->1037 0.20105

Excited State 261: Singlet-A 5.1522 eV 240.64 nm f=0.0052

<S**2>=0.000

1019 ->1054 -0.09990

1025 ->1073 0.11073

Excited State 262: Singlet-A 5.1527 eV 240.62 nm f=0.0022

<S**2>=0.000

982 ->1032 0.12649

982 ->1034 0.12261

985 ->1034 0.13455

997 ->1036 -0.11195

1020 ->1052 0.12817

Excited State 263: Singlet-A 5.1531 eV 240.60 nm f=0.0074

<S**2>=0.000

997 ->1036 0.12253

1027 ->1072 0.11389

1029 ->1085 0.11183

1029 ->1086 -0.10106

1029 ->1088 -0.10337

Excited State 264: Singlet-A 5.1536 eV 240.58 nm f=0.0091

<S**2>=0.000

981 ->1033 0.10415

997 ->1036 0.10090

1018 ->1048 -0.10292

1021 ->1055 0.10954

Excited State 265: Singlet-A 5.1542 eV 240.55 nm f=0.0023

<S**2>=0.000

980 ->1032 0.10560

1007 ->1042 -0.09818

Excited State 266: Singlet-A 5.1558 eV 240.47 nm f=0.0025

<S**2>=0.000

968 ->1030 0.11441

980 ->1032 0.17030

Excited State 267: Singlet-A 5.1561 eV 240.46 nm f=0.0006

<S**2>=0.000

998 ->1039 -0.10030

1029 ->1090 0.12563

1029 ->1091 0.10128

Excited State 268: Singlet-A 5.1568 eV 240.43 nm f=0.0009

<S**2>=0.000

981 ->1033 0.09676

986 ->1033 -0.10149

995 ->1038 0.10460

Excited State 269: Singlet-A 5.1572 eV 240.41 nm f=0.0053

<S**2>=0.000

1015 ->1045 0.13562

1018 ->1048 0.12395

1020 ->1052 0.12668

1027 ->1079 -0.10499

1029 ->1086 0.10273

1029 ->1091 0.13320

Excited State 270: Singlet-A 5.1576 eV 240.39 nm f=0.0003

<S**2>=0.000

993 ->1036 0.12583

1020 ->1052 0.15237

1021 ->1055 -0.11792

1027 ->1072 -0.11191

Excited State 271: Singlet-A 5.1586 eV 240.35 nm f=0.0010

<S**2>=0.000

989 ->1035 0.10501

990 ->1036 0.10734

992 ->1035 -0.09914

995 ->1036 -0.10512

Excited State 272: Singlet-A 5.1594 eV 240.31 nm f=0.0025

<S**2>=0.000

987 ->1032 0.10558

1007 ->1042 -0.10044

1020 ->1052 -0.09788

1021 ->1052 -0.09838

1026 ->1072 -0.10087

1027 ->1080 -0.10287

Excited State 273: Singlet-A 5.1605 eV 240.26 nm f=0.0041

<S**2>=0.000

984 ->1033 0.10412

993 ->1036 0.11796

1015 ->1045 0.11076

Excited State 274: Singlet-A 5.1606 eV 240.25 nm f=0.0027

<S**2>=0.000

993 ->1036 0.16770

999 ->1038 0.11429

1015 ->1045 -0.16163

1024 ->1070 0.10783

Excited State 275: Singlet-A 5.1616 eV 240.20 nm f=0.0028

<S**2>=0.000

1015 ->1045 0.21159

Excited State 276: Singlet-A 5.1622 eV 240.18 nm f=0.0019

<S**2>=0.000

1007 ->1042 -0.11383

1015 ->1045 0.20021

Excited State 277: Singlet-A 5.1628 eV 240.15 nm f=0.0017

<S**2>=0.000

993 ->1036 0.11220

Excited State 278: Singlet-A 5.1640 eV 240.09 nm f=0.0100

<S**2>=0.000

1007 ->1042 0.20358

1017 ->1047 0.10921

Excited State 279: Singlet-A 5.1642 eV 240.08 nm f=0.0021

<S**2>=0.000

995 ->1039 -0.12820

998 ->1039 0.12495

999 ->1039 -0.10941

1000 ->1039 0.18256

1015 ->1045 -0.14788

1024 ->1070 -0.10750

Excited State 280: Singlet-A 5.1645 eV 240.07 nm f=0.0009

<S**2>=0.000

975 ->1030 0.15629

993 ->1036 0.17011

Excited State 281: Singlet-A 5.1655 eV 240.02 nm f=0.0052

<S**2>=0.000

985 ->1033 -0.10520

989 ->1036 -0.10327

1025 ->1071 0.13034

Excited State 282: Singlet-A 5.1659 eV 240.01 nm f=0.0014

<S**2>=0.000

975 ->1030 0.13735

976 ->1030 -0.10077

978 ->1030 -0.11043

1000 ->1039 -0.10436

Excited State 283: Singlet-A 5.1674 eV 239.93 nm f=0.0050

<S**2>=0.000

982 ->1034 0.13256

985 ->1033 -0.11389

Excited State 284: Singlet-A 5.1677 eV 239.92 nm f=0.0013

<S**2>=0.000

1021 ->1066 -0.10326

1022 ->1066 0.12724

Excited State 285: Singlet-A 5.1679 eV 239.91 nm f=0.0012

<S**2>=0.000

988 ->1032 0.13303

Excited State 286: Singlet-A 5.1689 eV 239.87 nm f=0.0022

<S**2>=0.000

1015 ->1045 0.13788

1023 ->1061 0.11709

Excited State 287: Singlet-A 5.1695 eV 239.84 nm f=0.0024

<S**2>=0.000

988 ->1032 0.15600

992 ->1035 0.10412

Excited State 288: Singlet-A 5.1703 eV 239.80 nm f=0.0014

<S**2>=0.000

988 ->1032 0.10577

1007 ->1042 0.10645

1024 ->1070 -0.09601

1026 ->1073 0.09886

Excited State 289: Singlet-A 5.1711 eV 239.76 nm f=0.0050

<S**2>=0.000

982 ->1033 -0.14000

982 ->1034 0.11707

1007 ->1042 0.18739

Excited State 290: Singlet-A 5.1721 eV 239.72 nm f=0.0002

<S**2>=0.000

987 ->1035 -0.11152

988 ->1035 0.20776

Excited State 291: Singlet-A 5.1725 eV 239.70 nm f=0.0045

<S**2>=0.000

982 ->1033 0.11562

985 ->1034 -0.10953

992 ->1038 -0.10079

1007 ->1042 0.13914

1016 ->1048 0.11724

Excited State 292: Singlet-A 5.1730 eV 239.68 nm f=0.0011

<S**2>=0.000

1016 ->1048 0.12775

1019 ->1048 0.11519

Excited State 293: Singlet-A 5.1733 eV 239.66 nm f=0.0021

<S**2>=0.000

1015 ->1045 0.12777

Excited State 294: Singlet-A 5.1744 eV 239.61 nm f=0.0006

<S**2>=0.000

989 ->1037 0.14569

990 ->1035 -0.13813

991 ->1037 -0.10283

1027 ->1082 0.12784

Excited State 295: Singlet-A 5.1750 eV 239.58 nm f=0.0004

<S**2>=0.000

992 ->1039 0.13082

996 ->1039 0.14925

Excited State 296: Singlet-A 5.1759 eV 239.54 nm f=0.0024

<S**2>=0.000

966 ->1030 -0.10189

971 ->1030 -0.11121

991 ->1035 0.14903

994 ->1037 -0.10859

1028 ->1086 -0.10239

Excited State 297: Singlet-A 5.1763 eV 239.52 nm f=0.0092

<S**2>=0.000

1006 ->1040 -0.11990

1025 ->1073 0.12936

Excited State 298: Singlet-A 5.1773 eV 239.48 nm f=0.0006

<S**2>=0.000

1007 ->1042 0.11610

Excited State 299: Singlet-A 5.1784 eV 239.42 nm f=0.0035

<S**2>=0.000

1005 ->1040 0.09466

1017 ->1048 -0.09390

1025 ->1079 -0.08876

1026 ->1079 -0.09079

Excited State 300: Singlet-A 5.1787 eV 239.41 nm f=0.0005

<S**2>=0.000

984 ->1033 0.10923

991 ->1037 0.12079

992 ->1039 0.12495

1025 ->1073 -0.10608
